# Systematic evaluation of the DeepSeek large language model for clinical diagnostic reasoning

**DOI:** 10.1371/journal.pone.0346078

**Published:** 2026-05-08

**Authors:** Yang Wang, Yang He, Xuchang Qin, Yucai Hong, Lin Chen, Jing Zhang, Hongying Ni, Zhongheng Zhang

**Affiliations:** 1 Department of Emergency Medicine, Sir Run Run Shaw Hospital, Zhejiang University School of Medicine, Hangzhou, China; 2 School of Medicine, Shaoxing University, Shaoxing, Zhejiang, China; 3 Department of Neurosurgery, Affiliated Jinhua Hospital, Zhejiang University School of Medicine, Jinhua, China; 4 Department of Critical Care Medicine, Affiliated Jinhua Hospital, Zhejiang University School of Medicine, Jinhua, China; 5 Provincial Key Laboratory of Precise Diagnosis and Treatment of Abdominal Infection, School of Medicine, Sir Run Run Shaw Hospital, Zhejiang University, Zhejiang‌‌, China; 6 Longquan Industrial Innovation Research Institute, Lishui, China; The Affiliated Hospital of Zhejiang Chinese Medical University, CHINA

## Abstract

**Background:**

Artificial intelligence (AI) is undergoing an era of transformative advancement, particularly through the emergence of Transformer-based large language models (LLMs). While these systems demonstrate strong reasoning and generalization capabilities, their clinical applicability, particularly in emergency and critical care decision-making, remains underexplored.. In time-sensitive settings, diagnostic reasoning must align rigorously with evidence-based standards and ensure the relevance of timing to clinical decisions.

**Objective:**

This study aims to provide a preliminary evaluation of the decision-support performance of the DeepSeek model in acute medical scenarios. We systematically evaluate its diagnostic reasoning, temporal consistency of recommendations, and adherence to evidence-based critical care protocols using standardized case-based assessments.

**Methods:**

Twenty-nine representative clinical cases were extracted from the Merck Manual of Diagnosis and Therapy, a widely used medical reference providing standardized case descriptions. The model’s outputs were evaluated across four decision-making dimensions: differential diagnosis, diagnostic testing, final diagnosis, and management planning. Human raters scored each response for accuracy, and multivariable linear regression was applied to assess associations between performance and case parameters (age, gender, and Rapid Emergency Medicine Score [REMS]).

**Results:**

DeepSeek achieved an overall mean accuracy of 82.9% (95% CI: 80.2–85.6%) across all cases. Accuracy peaked in final diagnosis (97.7%), but declined in differential diagnosis (73.0%). Model performance showed no significant variation across demographic or severity strata.

**Conclusions:**

DeepSeek shows promising performance in structured case-based diagnostic tasks, particularly in confirmatory diagnostic reasoning. However, its early-stage reasoning and handling of ambiguous cases require enhancement. Future studies using larger and more diverse clinical datasets are needed to further evaluate the model’s robustness and potential clinical applicability.

## Introduction

Artificial intelligence (AI) continues to advance rapidly, offering transformative opportunities to reshape the ways we discover, diagnose, and treat diseases, as well as how we understand human health and pathology [1]. In medicine, AI technologies offer significant advantages, including clinical support and multilingual assistance. While AI cannot replace physicians’ expertise, it can assist decision-making by analyzing patient data quickly to help make faster and more accurate diagnoses and treatment decisions [2]. In emergency medicine, rapid onset, swift progression, and complex symptom presentations are common. These conditions are often atypical and can easily be confused with other diseases. AI’s capabilities in big data processing and deep learning may assist emergency physicians in promptly identifying life-threatening conditions, improving diagnostic accuracy, and enhancing clinical outcomes [3]. In future emergency department (ED) settings, AI has the potential to serve as a reliable and supportive diagnostic tool [4]. Integrating AI into clinical workflows can enhance decision-making and improve patient outcomes [5]. At the same time, this evolution requires emergency physicians to develop digital literacy, including learning how to effectively utilize AI tools to synthesize medical histories, vital signs, and laboratory data into structured inputs for analysis. Such integration promotes continuous optimization of AI-driven decision support within dynamic healthcare environments [6].

DeepSeek is a large-scale sparse Mixture of Experts (MoE) model with a dynamic gating mechanism that efficiently allocates computational resources: compared with dense Transformer-based models, it achieves a 30–50% improvement in domain-specific precision in applications like healthcare. Its Domain-Adaptive Pre-Training (DAPT) framework, supported by patented technologies such as Knowledge Hypergraph Embedding, encodes authoritative clinical knowledge bases (e.g., UpToDate guidelines) into interpretable vector representations. In financial report generation, DeepSeek demonstrates a factual error rate below 2%. However, in complex medical scenarios such as differential diagnosis of chest pain, whether DeepSeek can achieve near-perfect diagnostic accuracy and effective identification of critical values remains uncertain [7]. This study evaluates DeepSeek’s potential as a clinical decision-support system by utilizing its capabilities in text comprehension, data integration, and fast reasoning [8]. Specifically, standardized clinical case simulations were used to evaluate DeepSeek’s performance in differential diagnosis, diagnostic test recommendation, management planning, and final diagnosis generation based on structured case information.

## Methods

### Study design

**Fig 1:**
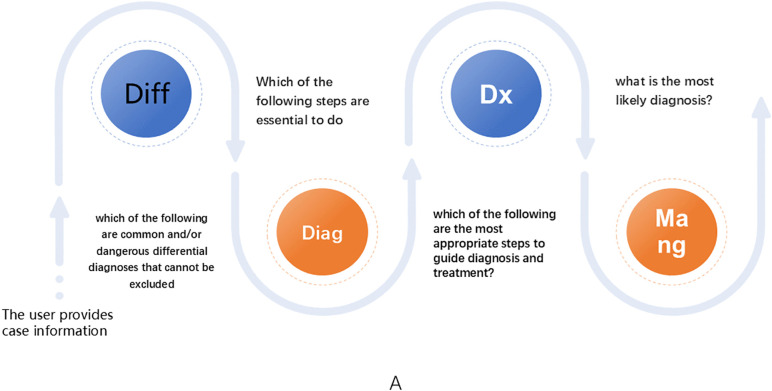
User operation process diagram. **Panel A:** Schematic diagram illustrating the operational workflow of DeepSeek in this experiment. The initial stage involves inputting patient medical history information into the model, followed by sequential prompts for differential‌‌ diagnosis, diagnostic testing, final diagnosis, and clinical management. All AI-generated responses were recorded for subsequent evaluation.

As shown in Fig 1, we evaluated the diagnostic accuracy of DeepSeek across multiple aspects of clinical decision-making and examined its sensitivity to patient age, gender, and clinical presentation. Each component of the clinical workflow: differential diagnosis, diagnostic testing, final diagnosis, and clinical management, was presented to the model as a sequence of structured prompts. The AI-generated responses were recorded for subsequent analysis.

All outputs were generated using the DeepSeek version released on February 9, 2025. To ensure consistency across experiments, each clinical case was evaluated in a new independent session. Patient history information was first entered into the model, followed by sequential prompts addressing differential diagnosis, diagnostic testing, final diagnosis, and management planning. All evaluators followed the same standardized prompting protocol. Model parameters such as temperature and top-p were maintained at the default system settings throughout the evaluation. The experiments were conducted using the publicly available DeepSeek interface under consistent computational conditions. Because the primary objective of this study was to evaluate diagnostic reasoning performance rather than real-time clinical deployment, system latency was not included as a study variable. No few-shot examples were used during the evaluation. All cases were evaluated using a zero-shot prompting strategy.

### Ethics Exemption and Study Workflow‌‌

This statement aims to clarify the ethics review exemption status of the current research project and confirm that the study does not involve human subjects.

DeepSeek is a large language model built on the Transformer architecture, featuring robust capabilities in contextual understanding and text integration. It can be customized for specific domains by incorporating specialized databases, thereby enhancing domain-specific reasoning, reducing hallucinations, and improving factual accuracy. In this study, all outputs were generated using the DeepSeek version released on February 9, 2025.

The Merck Manual is an authoritative and widely used reference source in the medical community. Published by Merck & Co., Inc., it provides comprehensive information on diseases, diagnostic methods, and treatment protocols. For this study, 29 representative clinical cases were selected from the Merck Manual, all publicly accessible as of the current date. These cases met the input standards compatible with DeepSeek and were used to evaluate the model’s diagnostic reasoning and decision-support performance.

Patient medical histories from each case were directly entered into DeepSeek, followed by sequential prompts addressing differential diagnosis, diagnostic testing, final diagnosis, and clinical management (see Fig 1). Because DeepSeek is a text-based model incapable of processing visual information, questions requiring image interpretation were handled by directly providing the correct findings as textual inputs‌‌.

DeepSeek generates responses through contextual reasoning based on the input prompt. To prevent interference between different cases, a new DeepSeek session was initiated for each clinical scenario. This design ensured that the information from one case did not influence subsequent analyses, allowing the model to focus exclusively on the medical history and data of the current patient. Such isolation improved the reliability and consistency of the AI’s diagnostic reasoning.

Three evaluators tested each clinical case using identical descriptions and prompts. AI-generated responses were recorded and compared to reference answers from the Merck Manual, with each correct response earning one point. The scoring process was conducted by a single independent rater, who assessed all outputs and documented the scores. The final score for each question was calculated as the mean accuracy across the three repetitions.

### Calculation of overall diagnostic accuracy

The overall diagnostic accuracy was determined through a structured multi-level averaging process designed to integrate independent evaluations and ensure consistency. For each of the 29 clinical cases, three independent users separately entered identical case information into the same DeepSeek model. The model generated diagnostic outputs across four diagnostic stages: differential diagnosis, initial diagnosis, intermediate reasoning, and final diagnosis.

Each user’s diagnostic outputs were independently evaluated by a clinical expert according to predefined criteria, including diagnostic correctness, logical coherence, and appropriateness of management recommendations. For each case, the scores of the three users were averaged within each diagnostic stage to obtain a final stage-specific accuracy. The four stage-level accuracies were then averaged to yield a composite diagnostic accuracy for that case. Finally, the mean of these composite accuracies across all 29 cases was reported as the overall diagnostic accuracy (82.9%).

**Fig 2:**
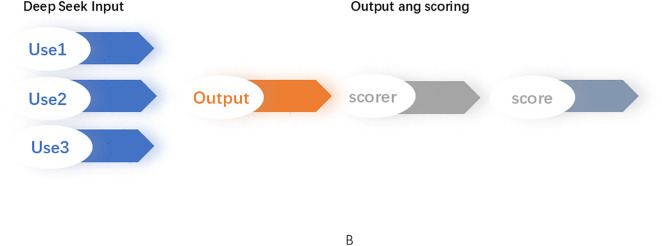
Study design. **Panel B:** Schematic diagram of the experimental workflow. Cases were selected from the Merck Manual clinical handbook and converted into text inputs compatible with DeepSeek. For questions requiring image interpretation, the final correct result was directly provided, and DeepSeek was tasked with analyzing it. Three independent users tested each prompt. A single independent scorer calculated scores for all outputs. Abbreviations: Diag: diagnostic questions; Diff: differential diagnosis; Dr: diagnostic testing questions; Mang: management questions.

For questions involving multiple correct responses (e.g., several differential diagnoses, diagnostic tests, or management steps), individual items were scored separately, and the mean value was used as the overall result. A schematic representation of the entire workflow is shown in Fig 2. All case-level and user-level diagnostic scores were recorded in a structured dataset (see S1 Data).

For each of the 29 clinical cases, three independent users entered identical case information into the DeepSeek model, and their outputs were scored independently by a clinical expert. The mean of the three user scores for each case was recorded as the DeepSeek_Average, representing the overall diagnostic accuracy for that case. A detailed description of all variables included in the dataset is provided in S1 Text.

### Participants and variables overview

The study utilized hypothetical cases based on Merck Manual descriptions, each including patient age, gender, and key clinical features. To assess performance variation with case severity, we applied the Rapid Emergency Medicine Score (REMS), stratified into four tiers: low risk (0–5), moderate (6–8), high (9–13), and very high (≥14). The accuracy of DeepSeek’s responses was measured across these strata.

As shown in Table 1, the baseline characteristics of the 29 clinical cases were summarized, with the majority of cases classified as low risk (86.2%), followed by very high risk (10.3%), and a small proportion classified as moderate risk (3.4%). No cases were classified as high risk.

**Table 1:** Baseline characteristics of the 29 clinical cases.

Variable	Value
Age (years), median (range)	49(2-80)
Sex (male: female)	18:11
REMS category	2(0-23)
Low (0–5)	25 (86.2%)
Moderate (6–8)	1 (3.4%)
High (9–13)	0 (0%)
Very high (≥14)	3 (10.3%)

This study employed a multiple linear regression model (implemented using SPSS Statistics version 25.0) to analyze the factors influencing DeepSeek’s performance in answering clinical questions. The dependent variable was the accuracy rate of responses to each question, which was confirmed to follow an approximately normal distribution via the Shapiro-Wilk test. The independent variable system encompassed three dimensions: (1) question characteristics (using “comprehensive knowledge” questions as the reference baseline), (2) patient characteristics (age and gender), and (3) clinical acuity indicators (Rapid Emergency Medicine Score, REMS).

As shown in Table 2, the multivariable linear regression analysis was conducted to examine the relationship between DeepSeek’s diagnostic accuracy and variables such as patient age, gender, Rapid Emergency Medicine Score (REMS), and question type.

**Table 2:** Multivariable linear regression analyzing the relationship between DeepSeek’s accuracy and patient age, gender, Rapid Emergency Medicine Score (REMS), as well as question type.

Variable	*P* value
Age	0.207
Sex	0.328
REMS	0.267
Diff	<0.001
Diag	<0.001
Dx	0.001
Mang	<0.001

## Results

### Clinical diagnostic model performance evaluation report

**Fig 3:**
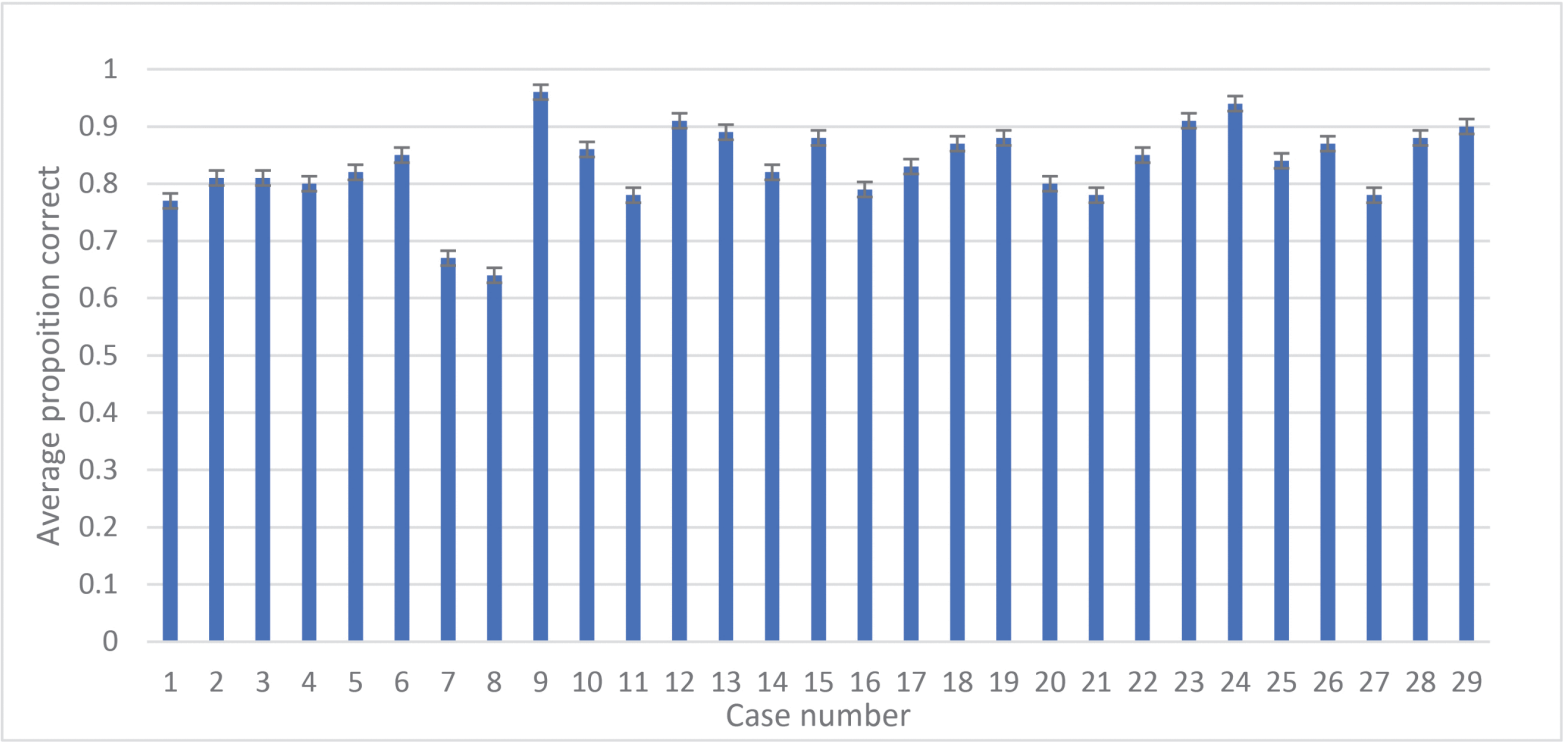
Overall performance of DeepSeek across 29 Merck Manual cases; error bars represent 1 standard error (SE) of the mean.

**Fig 4:**
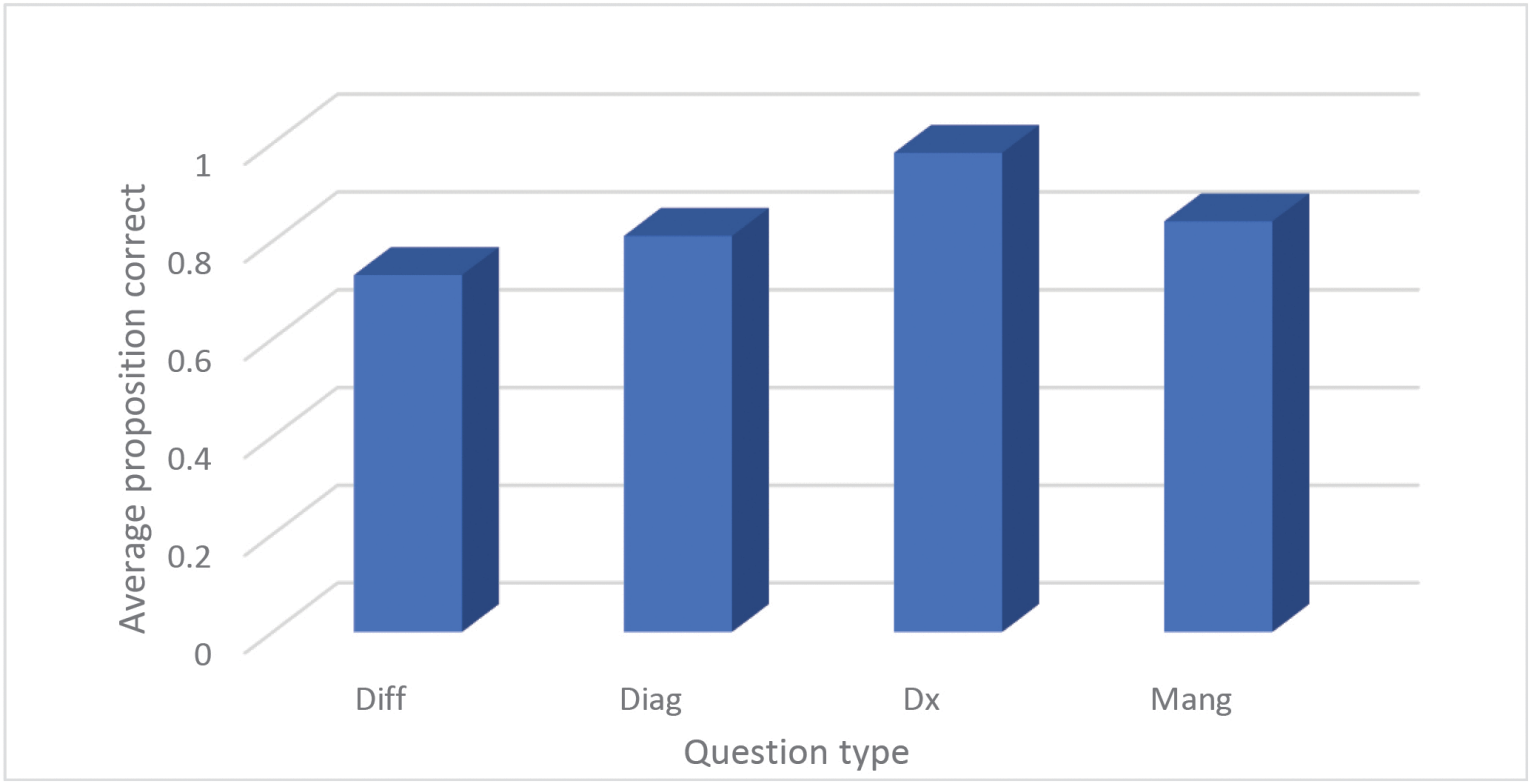
DeepSeek’s performance stratified by question type.

As shown in Fig 3, across the 29 clinical cases derived from the Merck Manual, DeepSeek achieved a mean diagnostic accuracy of 82.9% (range: 64–96%). As shown in Fig 4, performance was highest in final diagnostic reasoning (97.7%) and lowest in differential diagnosis (73.0%), indicating that DeepSeek excels at consolidating definitive diagnostic cues but demonstrates limitations in early-stage reasoning where diagnostic ambiguity is greater.

**Fig 5:**
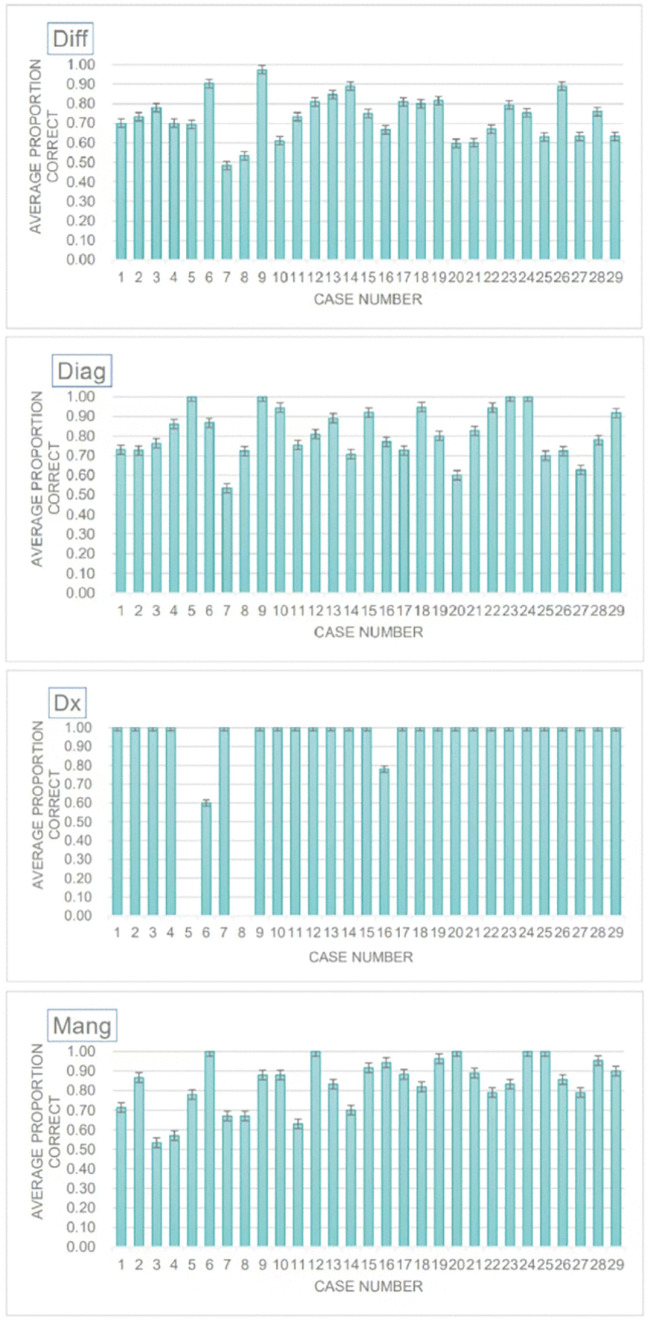
DeepSeek performance by question type. DeepSeek’s performance for each question type across the 29 Merck Manual cases; error bars represent 1 SE of the mean. Dx accuracy was not calculated for Cases 5 and 8 because the original case descriptions did not include a defined diagnostic endpoint.

The overall diagnostic accuracy of 82.9% was derived through a structured multi-level averaging process involving multiple independent evaluators. As shown in Fig 5 for each clinical case, three independent users separately entered the same case information into the DeepSeek model, and the model produced diagnostic outputs across four diagnostic stages: differential diagnosis, initial diagnosis, intermediate reasoning, and final diagnosis. Each user’s outputs were independently evaluated and scored by a clinical expert based on diagnostic correctness, reasoning consistency, and the appropriateness of subsequent management recommendations. For each diagnostic stage, the mean of the three user scores was calculated to obtain the final stage-specific accuracy for that case. The average of these four stage-level accuracies was then computed to yield a composite diagnostic accuracy score for that case. Finally, the mean of these composite scores across all 29 clinical cases was reported as the overall diagnostic accuracy (82.9%). Dx accuracy was not calculated for Cases 5 and 8 because the original case descriptions did not include a defined diagnostic endpoint.

DeepSeek achieved near-perfect performance in prototypical single-disease cases but showed reduced accuracy in multi-factorial or atypical presentations. For example, in a 24-year-old male presenting with dyspnea and back pain, accuracy was 64%, primarily due to difficulty in prioritizing among similar pathologies (e.g., pneumothorax, pulmonary embolism). Conversely, cases with well-defined disease patterns, such as uremic pulmonary edema, yielded accuracies as high as 96%. The model’s inability to dynamically request clarifying information remains a limiting factor for real-world clinical deployment.

Multivariable linear regression analysis revealed that question type was a significant predictor of performance (*p* < 0.001 for Diff, Diag, Dx, and Mang), whereas patient age, sex, and REMS did not reach statistical significance (see Table 2). This finding suggests that DeepSeek’s reasoning consistency is largely invariant across demographic characteristics and clinical severity. The model strata achieved an adjusted R-squared value of 0.91, indicating an excellent overall model fit and strong robustness in structured diagnostic reasoning tasks.

### Significant performance variations of the diagnostic system across specific clinical scenarios

The diagnostic model exhibited near-perfect performance in typical cases involving a single, well-defined pathology, achieving an accuracy of 1.00 in 25 out of 29 cases. However, its accuracy declined in cases involving extreme age groups (e.g., young children) and multidimensional or complex presentations (e.g., drug overdose combined with a mental health crisis), dropping to 0.60 and 0.78, respectively (see Fig 5).

In one case involving a 2-year-old male presenting with cough and dyspnea for one day, the final diagnostic accuracy was 0.60. The model correctly identified a foreign body in the right lung and highlighted that the right main bronchus, which is anatomically more vertical and wider, is the most common site for foreign body aspiration. However, it overlooked a critical detail in the medical history: prominent left-sided wheezing. This omission led to a partially incorrect conclusion, underscoring that AI-assisted diagnostic reasoning must integrate all available information: medical history, physical examination, and imaging findings, rather than relying on a single factor such as anatomical probability. When clinical data conflict with statistical likelihoods, dynamic reasoning based on objective evidence is essential to prevent bias in model decision-making.

In another case involving a 65-year-old male presenting with lethargy and confusion, the model achieved a final diagnostic accuracy of 0.83. DeepSeek’s responses consistently suggested calcium channel blocker overdose, whereas the correct diagnosis was beta-blocker overdose. This discrepancy indicates that the model may overemphasize psychological or behavioral factors (e.g., suicidal intent), thereby underweighting comprehensive pharmacologic assessment across all potential agents.

### Impact of different question types on DeepSeek’s performance

Task-specific variables demonstrated strong statistical significance. The complexity of differential diagnosis (Diff) (*p* < 0.001), completeness of diagnostic information (Diag) (*p* < 0.001), accuracy of the final diagnosis (Dx) (*p* = 0.001), and complexity of management decisions (Mang) (*p* < 0.001) were all positively correlated with model performance (see Table 2). Among these predictors, Diag exhibited the strongest effect, indicating that providing comprehensive diagnostic information substantially enhanced DeepSeek’s performance

### DeepSeek’s performance does not vary with demographic parameters or REMS

Demographic parameters (Age and Sex) and the Rapid Emergency Medicine Score (REMS), variables considered significant in human clinical experience, did not demonstrate a statistically significant impact (p > 0.05) (see Table 2). This suggests that these factors may lack predictive power within the model, indicating a potential divergence between the model’s decision-making framework and human clinical expertise. Alternatively, this could be due to an insufficient sample size, and further investigation is needed to clarify the source of these differences.

## Discussion

This study provides one of the first systematic evaluations of DeepSeek’s clinical reasoning capability in structured, case-based simulations. Overall, the model demonstrated strong alignment with evidence-based diagnostic reasoning, achieving an average accuracy of 82.9%. Its superior performance in final diagnostic conclusions (97.7%) highlights its ability to consolidate structured medical knowledge into high-confidence predictions, while reduced accuracy in differential diagnosis (73.0%) underscores challenges in uncertainty management and probabilistic reasoning under incomplete data conditions.

The results align with recent literature emphasizing both the promise and limitations of LLMs in clinical decision-making. For instance, GPT-4-based diagnostic tools have shown improved precision in emergency medicine, yet remain sensitive to contextual ambiguity and temporal misalignment. Similarly, DeepSeek’s limitations appear most pronounced in early decision stages where diagnostic pathways branch widely and multiple etiologies coexist. This finding parallels studies on model uncertainty propagation, suggesting that future architectures must integrate adaptive reasoning loops and multimodal signal processing.

Notably, DeepSeek’s diagnostic consistency was not significantly influenced by age, gender, or severity indicators such as REMS, implying that its reasoning process is invariant to basic demographic features. This differs from human clinicians, whose decision-making can be unconsciously biased by demographic cues. While this uniformity is advantageous for standardization, it may overlook clinically relevant contextual nuances, particularly in pediatric or geriatric cases.

From a methodological perspective, this work demonstrates how case-based benchmarking using authoritative medical resources like the Merck Manual can effectively quantify AI reasoning fidelity. Future efforts should expand this framework to include open-ended, multimodal inputs such as imaging data, dynamic vital signs, and natural-language physician-AI dialogue. Such integration could improve temporal coherence and decision interactivity, addressing one of the key weaknesses identified in this study [9].

The distribution of REMS scores was heavily skewed toward low-risk cases, which may limit the statistical power to detect severity-related performance variation. In addition, several limitations of this study should be acknowledged. First, the number of cases included in this evaluation was relatively small (n = 29), which may limit the statistical power of the analyses and the generalizability of the findings. Second, all cases were derived from the Merck Manual, which primarily contains well-structured textbook examples rather than the heterogeneous and often incomplete clinical information encountered in real-world emergency settings. Consequently, the present results should be interpreted as preliminary evidence of model performance in structured case-based scenarios rather than definitive evidence of clinical applicability.

In addition, because the Merck Manual is a publicly available medical reference, it is not possible to completely exclude the possibility that related content may have been included in the model’s training corpus. Consequently, the present study should not be interpreted as a strict out-of-sample evaluation. Future work may benefit from incorporating formal contamination detection approaches, such as text overlap analysis or perplexity-based methods, to better assess potential memorization effects.

Finally, this study did not include direct benchmarking against other large language models such as GPT-4 or Claude. Because LLMs evolve rapidly across successive releases, comparisons across models developed at different time points may introduce temporal bias. Therefore, the results should be interpreted as a preliminary evaluation, and future studies should incorporate standardized comparative evaluations across multiple models to provide a broader context for interpreting model performance in clinical decision-support tasks.

Although the multivariable regression model yielded a high adjusted R-squared, this finding should be interpreted cautiously given the limited sample size and the distribution of case characteristics. The regression analysis was performed as an exploratory approach to examine potential associations between case features and model performance, rather than to develop a robust predictive model. Future studies with larger and more heterogeneous datasets are required to validate these associations and to assess generalizability.

## Conclusion

The DeepSeek model demonstrated promising performance in structured clinical reasoning tasks, particularly in final diagnostic synthesis. However, its reduced accuracy in differential diagnosis highlights the need for enhanced probabilistic reasoning and multimodal data integration. Incorporating mechanisms for active inquiry, uncertainty calibration, and dynamic evidence weighting will be key to improving robustness in more complex clinical environments. Future studies should also explore longitudinal learning frameworks that allow continuous adaptation to evolving medical guidelines and patient diversity.

## Supporting information

S1 Data Dataset. DeepSeek diagnostic accuracy dataset. This dataset includes all case-level and user-level diagnostic scores across the 29 clinical cases.
(XLSX)

S1 Text Raw model outputs. This file contains the unprocessed outputs generated by DeepSeek for each clinical case, including the model’s responses across all diagnostic stages: differential diagnosis, diagnostic testing, final diagnosis, and management planning. The raw outputs are provided in their original format and serve as the foundation for the subsequent evaluation and scoring by the independent users and clinical experts.
(DOCX)
