# Supplementary material for: Systematic evaluation of the DeepSeek large language model for clinical diagnostic reasoning
Source: PLoS One. 2026 May 8;21(5):e0346078. doi: 10.1371/journal.pone.0346078 (PMC13155583; doi:10.1371/journal.pone.0346078)
Supplement: S1 Text — This file contains the unprocessed outputs generated by DeepSeek for each clinical case, including the model’s responses across all diagnostic stages: differential diagnosis, diagnostic testing, final diagnosis, and management planning. The raw outputs are provided in their original format and serve as the foundation for the subsequent evaluation and scoring by the independent users and clinical experts. (DOCX) [file pone.0346078.s002.docx]

| case1 | Deep seek1 | Deep seek2 | Deep seek3 |
| --- | --- | --- | --- |
| Diff | **Dangerous or Common Diagnoses that Cannot be Excluded Based on Current Data:**   1. **Pulmonary Embolism (PE)**   **Why:** Sudden-onset sharp chest pain, dyspnea, tachycardia (HR 107), and history of intermittent left leg swelling (possible DVT). No clear alternative explanation. Requires **CTPA** or D-dimer +/- imaging to exclude.   1. **Myocardial Infarction (MI)**   **Why:** Chest pain radiating centrally, persistent tachycardia, and dyspnea. Women often present atypically (e.g., sharp pain, non-exertional). Requires **serial troponins** and **ECG** to rule out.   1. **Aortic Dissection**   **Why:** Sudden-onset severe chest pain. Although she lacks hypertension or pulse deficits, dissection can occur without classic signs. Requires **CT angiography** to exclude.   1. **Myocarditis**   **Why:** Chest pain, tachycardia, and possible viral prodrome (chills). No clear pericardial rub or ECG changes yet, but myocarditis often mimics MI/PE. **Troponin** and **echocardiogram** are needed.  **Diagnoses Likely Excluded Based on History/Exam:**   - **Community-Acquired Pneumonia:** No fever, cough, crackles, or hypoxia. - **COPD Exacerbation:** No prior COPD history or wheezing. - **Severe Aortic Stenosis:** No murmurs, syncope, or heart failure signs. - **Viral Pericarditis:** No friction rub, positional pain, or ECG findings (e.g., diffuse ST elevation).   **Common but Less Dangerous Diagnoses to Consider:**   - **Gastroesophageal Reflux (GERD):** Possible, but less likely without typical burning/meal-related symptoms. - **Anxiety:** May contribute to tachycardia, but does not fully explain acute chest pain. - **Costochondritis:** Pain not reproducible on palpation, and NSAIDs failed to relieve.   **Next Steps to Narrow Diagnoses:**   - **Immediate:** ECG, troponin, CTPA (or D-dimer + lower extremity ultrasound). - **Urgent:** CT angiography if dissection remains a concern. - **Supportive:** Echocardiogram to assess for wall motion abnormalities (MI) or right heart strain (PE).   **Critical Takeaway:** PE and MI are the most urgent to exclude. Aortic dissection and myocarditis remain on the list until imaging/biomarkers confirm otherwise. | **Differential Diagnoses That Cannot Be Excluded Based on History and Physical Exam:**   1. **Aortic Dissection**   **Rationale:** Sudden onset sharp chest pain. While classic signs (e.g., unequal pulses, hypertension) are absent, dissection cannot be ruled out without imaging (e.g., CTA or transesophageal echo).   1. **Pulmonary Embolism (PE)**   **Rationale:** Sudden chest pain, dyspnea, tachycardia, and history of intermittent leg swelling (possible DVT). Normal oxygen saturation does not exclude PE. Requires CTA or V/Q scan.   1. **Myocardial Infarction (MI)**   **Rationale:** Atypical presentations (e.g., sharp pain in women) and tachycardia. Requires EKG and troponin to exclude.   1. **Myocarditis**   **Rationale:** Can present with chest pain and tachycardia without obvious infection. Troponin and echocardiography are needed for diagnosis.   1. **Viral Pericarditis**   **Rationale:** Sharp chest pain, though positional relief and friction rub are absent. EKG and echocardiography are required.  **Common but Less Dangerous Considerations:**   - **Gastroesophageal Reflux (GERD)** and **Anxiety** are possible but diagnoses of exclusion. - **Costochondritis** is less likely due to lack of tenderness on exam but cannot be fully excluded without palpation.   **Likely Excluded Diagnoses:**   - **Community-Acquired Pneumonia**: No cough, fever, or lung findings. - **COPD Exacerbation**: No history or exam findings (e.g., wheezing). - **Severe Aortic Stenosis**: No murmurs or typical symptoms (e.g., syncope).   **Critical Next Steps:**   - **Immediate Testing**: EKG, troponin, D-dimer, CTA chest (for PE/dissection), and echocardiography. - **Empirical Management**: Consider anticoagulation if PE is suspected pending imaging.   **Conclusion:** The most urgent and dangerous diagnoses requiring exclusion are **aortic dissection, PE, MI, myocarditis, and pericarditis**. These cannot be ruled out without further testing. | **Common and/or Dangerous Differential Diagnoses That Cannot Be Excluded:**   1. **Aortic Dissection**   **Rationale:** Sudden, sharp chest pain (though classic features like unequal BP or back pain are absent). Dangerous and requires imaging (CT aorta) to exclude.   1. **Pulmonary Embolism (PE)**   **Rationale:** Sudden onset chest pain, dyspnea, tachycardia, and history of intermittent leg swelling (possible prior DVT). Risk factors include anemia (hypercoagulability). Must exclude with CTPA.   1. **Myocardial Infarction (MI)**   **Rationale:** Central chest pain; women often present atypically. Requires EKG and troponin to exclude.   1. **Myocarditis**   **Rationale:** Tachycardia, chest pain, and possible viral prodrome (chills). Dangerous if severe; requires cardiac biomarkers and imaging (echocardiogram).   1. **Viral Pericarditis**   **Rationale:** Sharp, constant pain; NSAID non-response does not exclude. EKG may show diffuse ST elevation or PR depression.  **Excluded Diagnoses:**   - **Community-Acquired Pneumonia:** No cough, sputum, or lung findings (clear auscultation, normal O2 saturation). - **COPD Exacerbation:** No history of COPD or smoking; lungs clear. - **Costochondritis:** Pain not reproducible on palpation; NSAIDs ineffective. - **Gastroesophageal Reflux:** No food-related pain, heartburn, or nausea. - **Anxiety:** Not dangerous; secondary to pain but insufficient to explain symptoms. - **Severe Aortic Stenosis:** No murmur, syncope, or prior history.   **Immediate Next Steps:**   - **EKG** (for MI, pericarditis, myocarditis). - **Troponin** (MI/myocarditis). - **D-dimer ± CTPA** (PE). - **CT aorta** (if dissection suspected). - **Echocardiogram** (pericarditis, myocarditis, right heart strain in PE).   **Conclusion:** Prioritize excluding life-threatening causes (PE, MI, dissection) while evaluating for myocarditis/pericarditis. |
| Diag1 | **Essential Steps Before the Patient Leaves the ED:**   1. **Electrocardiogram (ECG):**   **Rationale:** Critical to assess for myocardial infarction (MI), ischemia, or signs of right heart strain (e.g., S1Q3T3 pattern in pulmonary embolism). Women often present atypically, and her tachycardia (HR 107) raises concern for cardiac or pulmonary pathology.   1. **Serum Troponin Concentration:**   **Rationale:** Mandatory to rule out myocardial injury. Elevated troponin would indicate MI, myocarditis, or demand ischemia (e.g., from anemia). Serial troponins may be needed if initial results are equivocal.   1. **Chest X-ray (Portable or Posteroanterior/Lateral):**   **Rationale:** Essential to exclude pneumothorax, pneumonia, pleural effusion, or mediastinal widening (aortic dissection). Even with clear lung auscultation, imaging is necessary to identify subtle abnormalities.   1. **CT Scan of the Chest (CT Pulmonary Angiography, CTPA):**   **Rationale:** Gold standard to rule out pulmonary embolism (PE), given her risk factors (intermittent leg swelling, anemia-associated hypercoagulability). Also evaluates for aortic dissection if clinical suspicion persists.   1. **Complete Blood Count (CBC):**   **Rationale:** Assess hemoglobin/hematocrit to evaluate anemia severity (history of menorrhagia) and platelet count (relevant for anticoagulation decisions if PE is confirmed).   1. **Serum D-dimer Levels:**   **Rationale:** If CT is delayed or contraindicated, D-dimer can help stratify PE risk. However, given her moderate pretest probability (leg swelling, tachycardia), proceeding directly to CTPA is reasonable.  **Not Essential Immediately:**   - **Arterial Blood Gas (ABG):** Pulse oximetry shows 96% on room air; ABG adds little unless hypoxemia or acid-base disturbance is suspected. - **Pro-BNP:** Not indicated without signs of heart failure (e.g., crackles, JVD). - **Complete Metabolic Panel (CMP):** Useful for baseline renal function (if contrast imaging is planned) but not urgent unless electrolyte abnormalities or renal failure are suspected.   **Key Takeaway:** The priority is to exclude life-threatening diagnoses (PE, MI, aortic dissection). **ECG, troponin, CXR, CTPA, and CBC** are indispensable. D-dimer may be omitted if CTPA is performed promptly. Expedited imaging (CTPA) is critical given her risk factors and symptoms. | **Essential Steps Before Leaving the ED:** The following tests are critical to rule out life-threatening causes of chest pain in this patient:   1. **Electrocardiogram (ECG):**   **Rationale:** Assess for STEMI, ischemia, pericarditis (e.g., ST-segment changes, PR depression).   1. **Serum Troponin Concentration:**   **Rationale:** Detect myocardial injury (to rule out acute coronary syndrome).   1. **CT Scan of the Chest (with contrast, if possible):**   **Rationale:** Evaluate for **pulmonary embolism (PE)** and **aortic dissection**. This is the gold standard for PE and provides definitive imaging for dissection.   1. **Serum D-dimer Levels:**   **Rationale:** If CT is delayed or unavailable, D-dimer helps risk-stratify for PE. However, if CT is performed immediately, D-dimer may be less critical but is still often included in initial workups.   1. **Complete Blood Count (CBC):**   **Rationale:** Assess for anemia (relevant given her history of menorrhagia) and infection.   1. **Complete Metabolic Panel (CMP):**   **Rationale:** Evaluate renal function (essential before administering IV contrast for CT) and electrolyte abnormalities.  **Tests Not Essential in This Case:**   - **Arterial Blood Gas (ABG):** Not routinely required for PE diagnosis (normal O₂ saturation and clear lung exam make hypoxia less likely). - **Chest X-ray (Portable or PA/Lateral):** While sometimes performed in chest pain workups, it is less critical here if CT is done, as CT provides superior evaluation for dissection, PE, and other thoracic pathology. - **Pro-Brain Natriuretic Peptide (Pro-BNP):** Not indicated without signs of heart failure (e.g., jugular venous distention, edema).   **Key Considerations:**   - **CT Chest vs. D-dimer:** If PE is suspected (given her history of leg swelling and tachycardia), CT angiography is the definitive test. D-dimer may be used if CT is unavailable or to guide imaging in lower-risk patients, but her moderate clinical suspicion warrants direct CT. - **Troponin and ECG:** Mandatory to exclude MI, even with atypical pain features (women often present atypically). - **CMP:** Critical to ensure renal safety before contrast administration for CT.   **Conclusion:** Prioritize **ECG, troponin, CT chest, D-dimer, CBC, and CMP** to exclude MI, PE, dissection, and other critical diagnoses before discharge. | **Essential Steps Before Leaving the ED:** The following tests are critical to exclude life-threatening diagnoses in this patient:   1. **Electrocardiogram (ECG):**   **Rationale:** Essential to assess for STEMI, pericarditis (diffuse ST elevation/PR depression), or ischemic changes (e.g., T-wave inversions).   1. **Serum Troponin Concentration:**   **Rationale:** Required to rule out myocardial injury (acute coronary syndrome or myocarditis).   1. **CT Scan of the Chest (CT Pulmonary Angiography, CTPA):**   **Rationale:** Gold standard to exclude pulmonary embolism (PE). Given her history of leg swelling (possible DVT) and sudden-onset dyspnea, CTPA is mandatory if clinical suspicion persists.   1. **Serum D-dimer Levels:**   **Rationale:** Indicated in moderate pre-test probability of PE (e.g., Wells Score ~4.5). A negative D-dimer in this context can exclude PE, avoiding unnecessary radiation.   1. **Complete Metabolic Panel (CMP):**   **Rationale:** Assess renal function (creatinine) before administering IV contrast for CT imaging.  **Excluded Options:**   - **Arterial Blood Gas (ABG):** Not routinely required for PE diagnosis (replaced by D-dimer/CTPA). - **Chest X-ray (Portable or PA/Lateral):** Less critical here (no respiratory signs on exam) and does not exclude key diagnoses like PE or dissection. - **Pro-BNP:** Not relevant to acute chest pain workup (no signs of heart failure). - **Complete Blood Count (CBC):** Useful but not immediately essential for life-threatening diagnoses.   **Key Considerations:**   - **Aortic Dissection:** If suspicion remains after initial workup, consider CT angiography of the aorta (not explicitly listed in options but clinically important). - **Contrast Safety:** CMP ensures safe contrast use for CTPA.   **Conclusion:** Prioritize **ECG, troponin, D-dimer, CTPA, and CMP** to exclude MI, PE, and ensure safe imaging. These steps address the most dangerous differentials (PE, MI, dissection) and guide definitive management. |
| Diag2 | **Essential Steps to Guide Diagnosis and Treatment:**   1. **CT Scan of the Chest with IV Contrast (CT Pulmonary Angiography, CTPA)**   **Rationale:**  Elevated D-dimer (395 ng/mL), S1Q3T3 on ECG (suggesting right heart strain), sudden-onset chest pain, and a history of intermittent leg swelling strongly indicate **pulmonary embolism (PE)**.  **CTPA is the gold standard** to confirm or rule out PE. Immediate imaging is critical for definitive diagnosis.  If PE is confirmed, therapeutic anticoagulation (e.g., enoxaparin or rivaroxaban) must be initiated promptly.   1. **Doppler Ultrasonography of the Legs**   **Rationale:**  The patient’s history of recurrent left leg swelling (last episode 10 days ago) raises suspicion for **deep vein thrombosis (DVT)**.  Identifying DVT supports a diagnosis of PE (via thrombus migration) and guides anticoagulation, even if CTPA is delayed or equivocal.  DVT may be subclinical or partially resolved, so ultrasound remains valuable despite the absence of current leg swelling.  **Steps Not Indicated at This Time:**   - **Cardiac Catheterization:**   Normal troponin (0.05 ng/mL) and nonspecific ST-T changes on ECG do not suggest acute coronary syndrome (ACS). Reserved for cases with dynamic ECG changes or rising troponin.   - **Pulmonary Function Testing (PFT):**   No chronic respiratory symptoms (e.g., cough, wheezing) to suggest COPD/asthma. PFTs are irrelevant for acute chest pain evaluation.  **Additional Critical Actions:**   - **Empiric Anticoagulation While Awaiting Imaging:**   If CTPA is delayed, consider starting low-molecular-weight heparin (LMWH) given her high clinical suspicion for PE (elevated D-dimer, S1Q3T3, and leg swelling history).  Weigh bleeding risks (mild anemia, no active bleeding) against thrombosis risks.   - **Repeat Troponin in 3–6 Hours:**   Despite normal initial troponin, serial testing is needed to exclude NSTEMI (non-ST-elevation myocardial infarction).  **Conclusion:** **CTPA and leg Doppler ultrasound are the priority steps** to confirm PE/DVT and guide anticoagulation. Immediate imaging and targeted therapy are critical to reduce morbidity/mortality. Cardiac catheterization and PFTs are unnecessary unless new cardiac/respiratory findings emerge. | **Most Appropriate Next Steps:**  **1. CT Scan of the Chest with IV Contrast (CT Pulmonary Angiography, CTPA)**   - **Rationale:**   **Critical to confirm or exclude pulmonary embolism (PE)** given the elevated D-dimer, S1Q3T3 ECG pattern, sinus tachycardia, and clinical history (leg swelling).  Gold standard for diagnosing PE and can also evaluate for aortic dissection or other thoracic pathology.   - **Action:** Perform immediately.   **2. Doppler Ultrasonography of the Legs**   - **Rationale:**   Identifies **deep vein thrombosis (DVT)**, which would support the diagnosis of PE (clot likely embolized from the legs).  Useful if CTPA is delayed or equivocal, or to guide anticoagulation duration if PE is confirmed.   - **Action:** Perform if CTPA is not immediately available or if clinical suspicion for DVT is high.   **Not Appropriate at This Stage:**   - **Cardiac Catheterization:**   Indicated for suspected acute coronary syndrome with **ongoing ischemia or rising troponin**, which this patient does not have (troponin normal, ECG nonspecific).  PE and other non-coronary causes are higher priority.   - **Pulmonary Function Testing (PFT):**   Evaluates chronic lung disease (e.g., COPD, asthma), which is not relevant in this acute setting.  No clinical signs (e.g., wheezing, chronic dyspnea) to suggest obstructive/restrictive lung disease.  **Additional Considerations:**   - **Empirical Anticoagulation:**   If CTPA is delayed, consider starting **low-molecular-weight heparin (LMWH)** given the high clinical suspicion for PE (elevated D-dimer, S1Q3T3, tachycardia, leg swelling).  Balance bleeding risk (e.g., anemia from menorrhagia; Hb 9.7 g/dL is stable for now).   - **Echocardiography:**   If CTPA confirms PE, perform to assess for right ventricular strain (prognostic significance).  **Conclusion:** **CT chest with IV contrast** is the **most critical next step** to diagnose PE. **Doppler ultrasound of the legs** is a supportive test if DVT is suspected. Cardiac catheterization and pulmonary function testing are not indicated in this acute scenario. | **Most Appropriate Steps:**   1. **CT Scan of the Chest with IV Contrast (CT Pulmonary Angiography, CTPA)**   **Rationale:** The patient has high clinical suspicion for PE (elevated D-dimer, S1Q3T3 on ECG, sinus tachycardia, leg swelling). CTPA is the **gold standard** to confirm or exclude PE. Immediate imaging is critical to guide anticoagulation.   1. **Doppler Ultrasonography of the Legs**   **Rationale:** Leg swelling raises concern for deep vein thrombosis (DVT). Identifying DVT supports the diagnosis of PE (as a potential source) and justifies anticoagulation, even if CTPA is delayed or inconclusive.  **Excluded Options:**   - **Cardiac Catheterization:**   Not indicated here. Normal troponin and lack of ischemic ECG changes (e.g., ST elevation/depression) rule out acute coronary syndrome as the primary diagnosis. The S1Q3T3 pattern and tachycardia are more consistent with PE-related right heart strain.   - **Pulmonary Function Testing (PFT):**   Irrelevant in this acute setting. PFTs assess chronic lung diseases (e.g., asthma, COPD), which are not supported by the patient’s presentation (normal lung exam, sudden-onset dyspnea).  **Key Considerations:**   - If CTPA confirms PE, start **therapeutic anticoagulation** (e.g., enoxaparin, apixaban) immediately. - If Doppler ultrasound reveals DVT, anticoagulation is still required, even if PE is not yet confirmed. - Anemia (Hb 9.7 g/dL) should be investigated after acute stabilization (e.g., iron studies, GI evaluation).   **Conclusion:** Prioritize **CTPA and Doppler ultrasound** to confirm PE/DVT and initiate life-saving treatment. Avoid unnecessary invasive or non-urgent tests. |
| Dx | **Most Likely Diagnosis:** **Pulmonary Embolism (PE)**  **Key Supporting Evidence:**   1. **Clinical Presentation:**   Sudden-onset sharp left-sided chest pain radiating centrally, dyspnea, and tachycardia (HR 107).  History of recurrent left leg swelling (possible DVT), a major risk factor for PE.   1. **Diagnostic Findings:**   **Elevated D-dimer (395 ng/mL):** Suggests thrombotic activity, though nonspecific.  **ECG with S1Q3T3 sign:** Classic for right heart strain, a hallmark of PE.  **Sinus tachycardia:** Consistent with PE-related hemodynamic stress.   1. **Exclusion of Alternatives:**   **Normal troponin (0.05 ng/mL)** and nonspecific ST-T changes make MI/myocarditis less likely.  No fever, cough, or crackles to suggest pneumonia.  No reproducible chest wall tenderness or NSAID response to support costochondritis.  Absence of pericardial rub or positional pain argues against pericarditis.  **Conclusion:** PE is the strongest diagnosis given the combination of risk factors (possible DVT), clinical features, elevated D-dimer, and ECG findings. **CT Pulmonary Angiography (CTPA)** is urgently needed for confirmation. | **Most Likely Diagnosis:**  **Pulmonary Embolism (PE)**  **Key Supporting Evidence:**   - **CT Chest with IV Contrast** confirmed **filling defects in bilateral upper and lower segmental pulmonary arteries**, which is diagnostic of acute PE. - Prior findings (elevated D-dimer, S1Q3T3 ECG pattern, sinus tachycardia, leg swelling) all align with PE.   **Excluded Alternatives:**   - **Community-acquired pneumonia:** No infiltrates or consolidation on imaging. - **Costochondritis:** Does not explain dyspnea, hypoxia, or CT findings. - **Myocardial infarction:** Normal troponin and nonspecific ECG changes (no ST elevation/depression). - **Myocarditis/Pericarditis:** No ECG findings (e.g., diffuse ST elevation, PR depression) or clinical signs (e.g., pericardial rub).   **Conclusion:** The imaging confirmation of PE makes this the definitive diagnosis. | **Most Likely Diagnosis:** **Pulmonary Embolism (PE)**  **Key Supporting Evidence:**   - **CT Pulmonary Angiography (CTPA)** showing **filling defects in bilateral pulmonary arteries** (segmental level), which is **diagnostic for PE**. - Prior findings (elevated D-dimer, S1Q3T3 ECG pattern, tachycardia, leg swelling) further corroborate the diagnosis.   **Why Other Options Are Unlikely:**   - **Community-acquired pneumonia:** No fever, cough, sputum, or lung consolidation on imaging. - **Costochondritis:** No chest wall tenderness or reproducible pain on palpation. - **Myocardial infarction:** Normal troponin and lack of ischemic ECG changes (e.g., ST elevation/depression). - **Myocarditis/Pericarditis:** No viral prodrome, pericardial rub, or diffuse ST elevations on ECG.   **Conclusion:** The CT findings are definitive for **PE**. Immediate anticoagulation and further management (as outlined earlier) are required. |
| Mang | **Most Appropriate Treatments:**   1. **Admit the Patient to the Hospital for Further Treatment**   **Rationale:** Pulmonary embolism (PE) carries significant morbidity and mortality risk. Admission allows for close monitoring of hemodynamics, initiation of anticoagulation, and rapid intervention if clinical deterioration occurs (e.g., right heart strain progression).   1. **Heparin 80 U/kg IV Bolus Followed by 18 U/kg/h IV Infusion**   **Rationale:** Unfractionated heparin (UFH) is a first-line therapy for acute PE, especially when rapid reversal may be needed (e.g., pending imaging confirmation or potential need for thrombolysis). It provides immediate anticoagulation while awaiting definitive diagnosis.  **OR**  **Rivaroxaban 15 mg PO Twice Daily**  **Rationale:** If PE is confirmed, direct oral anticoagulants (DOACs) like rivaroxaban are guideline-recommended for stable patients. The 15 mg BID regimen (for 21 days, then 20 mg daily) is effective and avoids the need for heparin bridging.   1. **Acetaminophen 650 mg PO Every 6 Hours as Needed for Chest Pain**   **Rationale:** Safe for pain relief in anticoagulated patients, as it does not increase bleeding risk (unlike NSAIDs like ibuprofen).  **Treatments to Avoid:**   - **Warfarin:** Requires bridging with heparin and INR monitoring, making it less practical than DOACs (e.g., rivaroxaban) in acute settings. - **Ibuprofen:** Contraindicated due to bleeding risk when combined with anticoagulants. - **Alteplase:** Reserved for **high-risk PE** (e.g., hypotension, shock), which this patient does not exhibit. - **Discharge with Outpatient Follow-up:** Inappropriate for a newly diagnosed or suspected PE requiring urgent anticoagulation and monitoring.   **Key Considerations:**   - If **CTPA confirms PE**, transition to long-term anticoagulation (e.g., rivaroxaban). - If **PE is ruled out**, investigate alternative causes (e.g., myocarditis, atypical ACS) based on troponin trends and imaging. - Address anemia (Hb 9.7 g/dL) with iron supplementation and gynecologic evaluation for menorrhagia.   **Conclusion:** Immediate **hospital admission**, **anticoagulation (heparin or rivaroxaban)**, and **acetaminophen** are essential. Avoid NSAIDs and outpatient management until PE is excluded or stabilized. | **Most Appropriate Treatments (Select All That Apply):**   1. **Admit the patient to the hospital for further treatment**   Rationale: Bilateral PE with potential intermediate-high risk (RV strain on prior assessment) requires inpatient monitoring for hemodynamic stability.   1. **Rivaroxaban 15 mg po bid**   Rationale: Direct oral anticoagulant (DOAC) is first-line for acute PE treatment. The 15 mg twice-daily dose is standard for the first 21 days, followed by 20 mg daily.   1. **Acetaminophen 650 mg po q 6 h as needed for chest pain**   Rationale: Safest analgesic for pain management in a patient on anticoagulation (avoids NSAID-induced bleeding risk).  **Why Other Options Are Not Appropriate:**   - **Discharge:** Unsafe for intermediate-high risk PE. - **Heparin IV bolus/infusion:** Typically reserved for high-risk PE (hemodynamic instability) or when thrombolysis/surgery is planned. Not required here if DOAC is initiated. - **Warfarin:** Requires bridging with parenteral anticoagulation (e.g., heparin/LMWH) and frequent INR monitoring. DOACs are preferred unless contraindicated. - **Ibuprofen:** Increases bleeding risk when combined with anticoagulants. - **Alteplase:** Indicated only for high-risk PE with hypotension/shock, which is not described here.   **Conclusion:** Admit, start rivaroxaban, and use acetaminophen for pain. | **Most Appropriate Treatments:**   1. **Admit the patient to the hospital for further treatment**   Rationale: Bilateral PE with segmental involvement is not low-risk (requires monitoring for hemodynamic instability, RV strain, and initiation of anticoagulation).   1. **Rivaroxaban 15 mg po bid**   Rationale: Direct oral anticoagulant (DOAC) is first-line for acute PE. The 15 mg twice-daily dose is standard for the first 21 days, followed by 20 mg daily.   1. **Acetaminophen 650 mg po q 6 h as needed for chest pain**   Rationale: Safe for pain relief in anticoagulated patients (avoids NSAID-associated bleeding risk).  **Why Other Options Are Not Appropriate:**   - **Discharge with follow-up:** Not safe for a patient with bilateral PE (requires hospitalization for monitoring and treatment initiation). - **Heparin infusion:** While valid in some cases (e.g., massive PE, renal failure), DOACs like rivaroxaban are preferred if no contraindications. - **Warfarin alone:** Requires bridging with heparin and is not first-line in the DOAC era. - **Ibuprofen:** Increases bleeding risk when combined with anticoagulants. - **Alteplase:** Reserved for **massive PE** (hypotension/shock) or **submassive PE with high-risk features** (e.g., RV strain, elevated biomarkers). No evidence of instability here.   **Next Step:** Initiate **rivaroxaban 15 mg bid**, admit for monitoring, and use acetaminophen for pain. |
| case2 |  |  |  |
| Diff | **Based on typical presentations and the need to prioritize life-threatening conditions, the following cannot be excluded without additional information or diagnostic workup:**   1. **Acute coronary syndrome (ACS), unstable angina, or acute MI**   **Critical to rule out due to potential for life-threatening complications. Symptoms like chest pressure, radiation, and associated symptoms (e.g., nausea, diaphoresis) require evaluation with ECG and troponin.**   1. **Aortic dissection**   **Sudden, tearing pain with risk factors (hypertension, connective tissue disorders) necessitates imaging (e.g., CT angiography) for exclusion.**   1. **Pulmonary embolism (PE)**   **Pleuritic pain, dyspnea, and risk factors (e.g., immobility, hypercoagulability) require assessment with D-dimer, CT angiography, or V/Q scan.**   1. **Pericarditis**   **Pain worsened by lying flat, relieved by leaning forward, and potential pericardial rub on exam. ECG findings (diffuse ST elevation) or echocardiography may support the diagnosis.**   1. **Myocarditis**   **Often preceded by viral illness, with possible ECG changes and elevated troponin. Cardiac MRI or endomyocardial biopsy may be needed for confirmation.**   1. **Gastroesophageal reflux disease (GERD)**   **Burning pain, postprandial symptoms, and response to antacids are suggestive but cannot be confirmed without ruling out cardiac causes first.**   1. **Musculoskeletal pain syndrome (e.g., costochondritis)**   **Reproducible tenderness on palpation, though coexisting conditions (e.g., ACS) must still be excluded.**   1. **Pneumonia**   **Fever, cough, or abnormal lung findings (e.g., crackles) may suggest this, but chest X-ray is required for confirmation.**   1. **Severe aortic stenosis**   **Systolic murmur, syncope, or heart failure signs warrant echocardiography for evaluation.**  **Excluded:**   - **Stable angina**   **Typically presents with exertional pain relieved by rest. In an acute setting with rest pain, unstable angina (part of ACS) is more relevant. Stable angina is less likely unless the history clearly aligns with chronic, predictable symptoms.**  **Conclusion: All listed diagnoses except stable angina cannot be excluded without further testing in an undifferentiated chest pain presentation.** | Based on the history and physical examination provided, the following differential diagnoses **cannot be excluded** at this time:   - **Acute coronary syndrome (ACS), unstable angina, or acute MI**   Despite the pain being currently absent and exertional (suggesting stable angina), the recent increase in frequency (e.g., during snow shoveling) and cardiac risk factors (smoking, hypertension, family history) raise concern for possible ACS. Definitive exclusion requires cardiac biomarkers and further testing (e.g., stress testing, angiography).   - **Aortic dissection**   Though the pain is not described as "tearing" and pulses are symmetrical, dissection can occasionally present atypically. Imaging (e.g., CT angiography) is required to fully rule it out.   - **Gastroesophageal reflux disease (GERD)**   The patient has a history of GERD, and symptoms can mimic angina. The exertional nature of the pain makes GERD less likely, but a trial of intensified PPI therapy or pH monitoring may be needed to exclude it.   - **Musculoskeletal pain syndrome (e.g., costochondritis)**   While the pain is not reproducible on palpation and lacks association with movement, musculoskeletal causes cannot be entirely excluded without further evaluation (e.g., response to NSAIDs, imaging).  **Differentials that can likely be excluded**:   - **Myocarditis/Pericarditis**: No recent infection, positional pain, or friction rub on exam. - **Pulmonary embolism (PE)**: Absence of dyspnea, hypoxia, or acute risk factors (e.g., immobility). - **Pneumonia**: No fever, cough, or auscultatory findings. - **Severe aortic stenosis**: No murmur on cardiac exam (normal S1/S2).   **Stable angina** is the leading diagnosis but is included as a possible answer option here. However, the question focuses on **differentials that remain on the list**, which includes the above conditions requiring further workup. | **Differential Diagnoses That Cannot Be Excluded:**   1. **Acute coronary syndrome (ACS), unstable angina, or acute MI**   Rationale: Exertional chest pain with classic anginal features (pressure, relieved by rest/aspirin) raises concern for CAD. His risk factors (smoking, hypertension, family history of early MI) and lack of troponin/ECG data mean ACS cannot be excluded.   1. **Stable angina**   Rationale: Most likely diagnosis given the exertional, reproducible nature of pain, risk factors, and symptom relief with rest/aspirin.   1. **Gastroesophageal reflux disease (GERD)**   Rationale: History of GERD and episodic chest pain that he attributed to indigestion. GERD can mimic angina and cannot be ruled out without further evaluation.   1. **Musculoskeletal pain syndrome, including costochondritis**   Rationale: Chest pain not reproducible on palpation, but musculoskeletal causes (e.g., costochondritis) remain possible despite the lack of tenderness or injury history.  **Why Other Diagnoses Are Less Likely or Can Be Excluded:**   - **Aortic dissection:** Unlikely due to the absence of sudden/severe tearing pain, radiation to the back, or physical findings (e.g., pulse deficits, murmur). - **Myocarditis/Pericarditis:** No recent infection, positional/pleuritic pain, or signs of heart failure/inflammation (e.g., fever, elevated biomarkers). - **Pneumonia:** No cough, fever, or auscultatory abnormalities. - **Pulmonary embolism (PE):** No dyspnea, pleuritic pain, or risk factors (e.g., immobility, DVT). - **Severe aortic stenosis:** No murmur on exam and absence of syncope/heart failure symptoms.   **Next Steps:**   - **Cardiac stress testing** (e.g., exercise treadmill, imaging) to confirm ischemia. - **Troponin/ECG** to rule out ACS if suspicion arises. - **GERD evaluation** (e.g., trial of PPI therapy, pH monitoring). - Consider **chest imaging** (e.g., CTA) if atypical features persist. |
| Diag1 | **In the evaluation of a patient with undifferentiated chest pain in a clinic setting, the following steps are essential to perform before the patient leaves, based on the critical need to rule out life-threatening conditions and the differential diagnoses previously considered (e.g., ACS, PE, pneumonia, aortic dissection, pericarditis, myocarditis):**   1. **ECG**   **Mandatory to assess for ischemic changes (e.g., ST-segment elevation/depression, T-wave inversions) or signs of pericarditis (diffuse ST elevation). Critical for diagnosing ACS or arrhythmias.**   1. **Pulse oximetry**   **Essential to evaluate oxygenation status. Hypoxia may indicate PE, pneumonia, or cardiac compromise, guiding urgent intervention or referral.**   1. **Chest x-ray**   **Necessary to identify pulmonary causes (e.g., pneumonia, pneumothorax) or signs of aortic dissection (widened mediastinum). Supports exclusion of non-cardiac etiologies.**   1. **Serum D-dimer level**   **Required if PE is suspected (e.g., pleuritic pain, dyspnea, risk factors). While D-dimer has limitations (e.g., low specificity), it is a first-line test in low-to-moderate risk patients to rule out PE. If the clinic can draw blood, this should be done before discharge, even if results are pending.**  **Final Answer:**   - **ECG** - **Pulse oximetry** - **Chest x-ray** - **Serum D-dimer level**   **Rationale: These steps address the most urgent differentials (ACS, PE, pneumonia, aortic pathology). While D-dimer results may take time, initiating the workup is critical. Stable angina is excluded in acute settings, but unstable angina (part of ACS) requires ECG and troponin (not listed, but implied if ACS is suspected).** | Based on the clinical scenario, the **essential steps to perform before the patient leaves the clinic** are:   1. **ECG**   **Critical** to assess for ischemia, arrhythmias, or signs of prior MI. Even if the patient is pain-free, an ECG is standard in evaluating chest pain, especially with cardiac risk factors (hypertension, smoking, family history).   1. **Pulse Oximetry**   A quick, non-invasive test to evaluate oxygenation. While the patient has no overt dyspnea or hypoxia on exam, it helps exclude subtle hypoxia (e.g., early pulmonary embolism, pneumonia) and complements vital signs.  **Steps not essential to perform immediately**:   - **Chest X-ray**: While useful for ruling out structural causes (e.g., pneumothorax, pneumonia), the patient lacks symptoms (e.g., cough, fever, focal lung findings) to prioritize this urgently. It can be deferred to outpatient follow-up. - **Serum D-dimer**: PE is unlikely given the absence of dyspnea, hypoxia, or acute risk factors (e.g., immobility, recent surgery). D-dimer is not indicated here and risks false-positive results.   **Key Point**: ECG and pulse oximetry are immediate, low-risk tests that provide critical information for guiding next steps (e.g., ruling out ACS, hypoxia). Other tests (e.g., chest X-ray, D-dimer) depend on clinical suspicion, which is low in this case. | **Essential Steps Before Discharge:**   1. **ECG**   **Rationale:** Mandatory for any patient with chest pain concerning for cardiac ischemia. Even if the patient is currently asymptomatic, an ECG can detect signs of ischemia (e.g., ST-segment changes, T-wave inversions) or prior infarction. This is critical to rule out ACS or silent MI.   1. **Pulse Oximetry**   **Rationale:** A quick, non-invasive test to assess oxygenation. While the patient lacks classic PE symptoms (e.g., dyspnea, hypoxemia), pulse oximetry is still standard in chest pain evaluation to exclude occult hypoxia.  **Why Other Options Are Not Essential:**   - **Chest X-ray:** Not immediately necessary in this case. The patient has no clinical features of pneumonia (no fever, cough), heart failure (no edema, clear lungs), or aortic dissection (no sudden tearing pain, pulse deficits). - **Serum D-dimer:** Not indicated here. The patient lacks risk factors or symptoms of PE (e.g., dyspnea, pleuritic pain, immobility). D-dimer is highly sensitive but nonspecific, and a positive result would likely lead to unnecessary radiation/imaging in this low-risk scenario.   **Next Steps After ECG/Pulse Oximetry:**   - If ECG shows ischemic changes (e.g., ST depression, T-wave inversions), **admit for troponin testing and further cardiac evaluation**. - If ECG is normal, proceed with outpatient stress testing and cardiology referral. - Reassess blood pressure management (current BP 162/86 mm Hg) and reinforce smoking cessation. |
| Diag2 | **Based on the clinical context of undifferentiated chest pain and the need to prioritize life-threatening conditions, the most appropriate next steps are:**   1. **Serum troponin level**   **Critical to evaluate for myocardial injury (e.g., acute MI, myocarditis). Troponin elevation guides urgent management of ACS or myocarditis.**   1. **CT angiography of the chest**   **Essential to rule out pulmonary embolism (PE) and aortic dissection if clinical suspicion persists after initial testing (e.g., elevated D-dimer, chest x-ray abnormalities).**  **Rationale:**   - **Troponin is indispensable for diagnosing acute coronary syndromes (ACS) or myocardial injury, even if the ECG is non-diagnostic.** - **CT angiography addresses two high-risk diagnoses (PE and aortic dissection) that cannot be excluded by chest x-ray or D-dimer alone.** - **Other options (e.g., stress testing, echocardiography, cardiac catheterization) are either premature (stress tests are for stable angina) or require initial biomarker/imaging results to guide use (e.g., echocardiography for pericarditis or wall motion abnormalities).**   **Excluded:**   - **Treadmill stress tests are inappropriate in acute chest pain until ACS is ruled out.** - **Cardiac catheterization is reserved for confirmed ACS or ischemia.** - **Electron beam CT assesses chronic coronary calcium, not acute pathology.**   **Answer:**   - **Serum troponin level** - **CT angiography of the chest** | The **most appropriate next step** to guide diagnosis and treatment in this patient is:  **Serum Troponin Level**  **Rationale**:   - The patient’s clinical history (exertional chest pain, risk factors) and ECG findings (LVH with secondary ST/T changes) raise concern for **acute coronary syndrome (ACS)**. - **Troponin** is the **gold-standard biomarker** to detect myocardial injury. Elevated troponin would confirm acute ischemia/infarction, necessitating **urgent cardiac catheterization**. - A **negative troponin** makes ACS less likely, allowing focus on evaluating for **stable coronary artery disease** (e.g., stress testing) or other causes of LVH (e.g., hypertensive heart disease).   **Other Options:**   1. **Cardiac Catheterization**:   Reserved for confirmed ACS (e.g., **positive troponin**) or high-risk features (e.g., dynamic ECG changes, ongoing pain). Premature without biomarker results.   1. **Transthoracic Echocardiography**:   Useful to assess LVH severity, systolic function, and rule out structural causes (e.g., valvular disease). However, **not urgent** unless suspicion of acute heart failure or cardiomyopathy.   1. **Stress Testing (ECG or Nuclear)**:   Indicated for **stable chest pain** to evaluate for inducible ischemia. However, this patient’s **worsening exertional symptoms** suggest possible **unstable angina**; stress testing is **not safe** until ACS is ruled out.   1. **CT Angiography of the Chest**:   Unnecessary here. Low suspicion for PE (no dyspnea, hypoxia, or risk factors).   1. **Electron Beam CT (Calcium Score)**:   Assesses coronary calcium burden but does **not guide acute management**.  **Next Steps Based on Troponin Result:**   - **If troponin is positive**: Proceed to **cardiac catheterization** for definitive diagnosis and revascularization. - **If troponin is negative**:   Perform **transthoracic echocardiography** to evaluate LVH, diastolic function, and exclude other structural disease.  Arrange **stress testing with nuclear imaging** (preferred over ECG-only stress testing due to LVH baseline ST/T abnormalities, which reduce ECG accuracy).  **Key Point**: Troponin is the **gatekeeper test** to differentiate ACS from stable CAD or non-cardiac causes in this scenario. Delay in ruling out ACS could lead to missed life-threatening ischemia. | **The most appropriate steps to guide diagnosis and treatment are:**   1. **Serum Troponin Level**   **Rationale:** Essential to rule out acute myocardial injury, especially given exertional chest pain and LVH on ECG. Elevated troponin would indicate ACS or demand ischemia, necessitating immediate admission. If normal, it supports proceeding to further outpatient evaluation.   1. **Transthoracic Echocardiography**   **Rationale:** Critical to assess the severity of LVH, left ventricular function, and structural abnormalities (e.g., aortic stenosis, wall motion abnormalities). This helps differentiate hypertensive heart disease from other causes of chest pain and guides long-term management.   1. **Treadmill Stress Test with Nuclear Imaging**   **Rationale:** The patient’s exertional symptoms and baseline ECG abnormalities (LVH with ST/T changes) make a standard ECG stress test unreliable. Nuclear imaging provides accurate ischemia detection by evaluating perfusion defects, even with uninterpretable ECGs.  **Why Other Options Are Less Appropriate:**   - **Cardiac Catheterization:** Invasive and reserved for high-risk patients or after positive non-invasive testing. Not first-line without evidence of ischemia. - **CT Angiography of the Chest:** Indicated for suspected PE/aortic dissection, which is not supported by the clinical picture. - **Electron Beam CT for Calcium Score:** Assesses plaque burden but does not evaluate for ischemia. Less useful in symptomatic patients requiring functional testing. - **Treadmill Stress Test with ECG Alone:** Baseline ST/T changes from LVH limit interpretability, reducing its diagnostic utility.   **Clinical Workflow:**   - **Immediate:** Check troponin. If elevated, admit for ACS management. - **If Troponin Normal:**   Perform echocardiography to evaluate LVH and cardiac structure.  Refer for stress testing with nuclear imaging to assess for inducible ischemia.   - **Urgent Cardiology Follow-Up:** Ensures coordination of further testing (e.g., coronary angiography if stress test is positive) and optimization of antihypertensive therapy.   **Key Consideration:** LVH increases cardiovascular risk, and exertional chest pain mandates excluding obstructive CAD. This triad (troponin, echocardiography, stress imaging) balances urgency, diagnostic accuracy, and risk stratification. |
| Dx | **Based on the findings from the treadmill stress test with nuclear imaging, the most likely diagnosis is:**  **Stable angina**  **Rationale:**   - **The large reversible perfusion defect in the LAD territory indicates inducible ischemia, which is consistent with obstructive coronary artery disease (CAD).** - **The ejection fraction of 60% suggests preserved systolic function, which is typical in stable angina.** - **Stable angina is characterized by predictable, exertional chest pain due to myocardial ischemia caused by fixed coronary artery stenosis. The stress test findings align with this diagnosis, as the ischemia was provoked during exertion (treadmill stress).**   **Why not the others?**   1. **Acute coronary syndrome (ACS):**   **ACS (unstable angina, NSTEMI, STEMI) presents with acute, ongoing ischemia at rest or with minimal exertion, often accompanied by ECG changes or elevated troponin. This patient’s findings are from a stress test, suggesting chronic, stable ischemia rather than an acute event.**   1. **GERD:**   **GERD typically causes burning epigastric or retrosternal pain unrelated to exertion and not associated with reversible perfusion defects on imaging.**   1. **Musculoskeletal:**   **Musculoskeletal pain is reproducible with palpation or movement and would not cause reversible perfusion defects on nuclear imaging.**   1. **Other conditions (e.g., myocarditis, pericarditis, PE):**   **These would present with different clinical and imaging findings (e.g., diffuse ST elevation in pericarditis, hypoxia in PE, or global inflammation in myocarditis).**  **Conclusion: The most likely diagnosis is stable angina due to obstructive CAD in the LAD territory, as evidenced by the reversible perfusion defect during stress testing.** | The **most likely diagnosis** for this patient is:  **Stable Angina**  **Rationale**:   - **Negative troponin** rules out acute myocardial injury (e.g., NSTEMI), making **acute coronary syndrome (ACS)** unlikely. - **Nuclear stress test findings**:   **Large reversible perfusion defect in the LAD territory** confirms **inducible ischemia** due to significant coronary artery disease (CAD).  **Normal ejection fraction (60%)** supports preserved systolic function, typical of stable angina rather than acute ischemia or infarction.   - **Clinical correlation**: Exertional chest pain (relieved by rest) with high-risk stress test findings aligns with **stable angina** caused by flow-limiting coronary stenosis.   **Why Other Options Are Less Likely:**   1. **Acute Coronary Syndrome (ACS)**:   Requires evidence of **acute myocardial injury** (elevated troponin) or **unstable angina** (rest pain, dynamic ECG changes). Neither is present here.   1. **GERD**:   Typically causes burning chest pain unrelated to exertion, often with acid reflux symptoms (e.g., regurgitation, heartburn). Not associated with ischemia on stress testing.   1. **Musculoskeletal**:   Pain is usually reproducible with palpation or movement, localized, and unrelated to exertion. No link to perfusion defects on imaging.  **Key Point**: The combination of **exertional symptoms**, **negative troponin**, and **high-risk stress test findings** confirms **stable angina** secondary to obstructive CAD. Urgent coronary angiography is required to guide revascularization (e.g., PCI or CABG). | **Most Likely Diagnosis:** **Stable Angina**  **Rationale:**   1. **Key Findings:**   **Large Reversible Perfusion Defect in LAD Territory:** Indicates **obstructive coronary artery disease (CAD)** with ischemia provoked during exertion.  **Normal Ejection Fraction (60%):** No acute myocardial dysfunction, which aligns with stable angina rather than acute myocardial infarction.  **Prior Normal Troponin:** Excludes acute myocardial injury (e.g., NSTEMI/STEMI), making ACS less likely.   1. **Clinical Context:**   Exertional chest pain that resolves with rest is classic for **stable angina**.  Stress test abnormalities correlate with fixed coronary stenosis causing demand-supply mismatch during physical activity.   1. **Why Not Acute Coronary Syndrome (ACS)?**   ACS requires evidence of **acute plaque rupture** (e.g., elevated troponin, dynamic ECG changes, or rest pain), which are absent here.  The patient’s symptoms and ischemia are **provoked by exertion and reproducible**, consistent with stable angina.   1. **Exclusion of Non-Cardiac Causes:**   **GERD/Musculoskeletal:** These lack objective evidence (e.g., normal stress imaging would be expected). The nuclear stress test directly links symptoms to cardiac ischemia.  **Conclusion:** The findings confirm **hemodynamically significant CAD** causing **exertional ischemia**, fulfilling criteria for **stable angina**. Immediate cardiac catheterization is warranted to define anatomy and plan revascularization (PCI/CABG). |
| Mang | **Given the diagnosis of stable angina with evidence of significant ischemia in the LAD territory on stress testing, the following are the most appropriate next steps:**   1. **Refer to a cardiologist for cardiac catheterization:**   **Cardiac catheterization is the gold standard to confirm the severity and location of coronary artery disease (CAD) and to guide revascularization (e.g., percutaneous coronary intervention [PCI] or coronary artery bypass grafting [CABG]).**   1. **Aspirin 81 mg once/day:**   **Aspirin is a cornerstone of CAD management to reduce the risk of thrombotic events. A low dose (81 mg) is typically recommended for long-term use in stable CAD.**   1. **Metoprolol 25 mg twice daily:**   **Beta-blockers (e.g., metoprolol) reduce myocardial oxygen demand by lowering heart rate and blood pressure, which helps prevent angina symptoms and improve outcomes in CAD.**   1. **Nitroglycerin 0.4 mg sublingual as needed for chest pain:**   **Nitroglycerin is a first-line therapy for acute angina episodes, providing rapid relief by dilating coronary arteries and reducing preload.**   1. **Reinforce need to adhere to antihypertensive treatment:**   **Hypertension is a major risk factor for CAD progression. Ensuring optimal blood pressure control is critical to reduce the risk of further ischemic events.**   1. **Serum lipid profile:**   **A lipid profile is essential to assess cardiovascular risk and guide statin therapy, which is indicated in all patients with CAD to lower LDL cholesterol and stabilize plaques.**  **Why not the others?**   - **Admit to the hospital telemetry unit:**   **Admission is not typically required for stable angina unless there is evidence of acute coronary syndrome (ACS), unstable symptoms, or high-risk features. This patient’s findings suggest stable CAD.**   - **Refer to a cardiothoracic surgeon for CABG:**   **Referral for CABG is premature without cardiac catheterization results. The decision for CABG vs. PCI depends on the anatomy and severity of CAD, which requires angiographic confirmation.**   - **Aspirin 325 mg once/day:**   **While 325 mg aspirin is sometimes used in acute settings (e.g., ACS), a lower dose (81 mg) is preferred for long-term management in stable CAD to minimize bleeding risk.**   - **Advise the patient to rest:**   **While rest is important during acute angina episodes, it is not a long-term management strategy. The focus should be on medical therapy, risk factor modification, and revascularization if indicated.**  **Final Answer:**   - **Refer to a cardiologist for cardiac catheterization** - **Aspirin 81 mg once/day** - **Metoprolol 25 mg twice daily** - **Nitroglycerin 0.4 mg sublingual as needed for chest pain** - **Reinforce need to adhere to antihypertensive treatment** - **Serum lipid profile** | The **most appropriate next steps** for this patient are:  **Admit to the hospital telemetry unit**   - **Rationale**: A large reversible LAD perfusion defect is a **high-risk finding** for near-term adverse cardiac events (e.g., myocardial infarction). Admission allows continuous monitoring, initiation of urgent therapies, and expedited coronary angiography.   **Refer to a cardiologist for cardiac catheterization**   - **Rationale**: Coronary angiography is **mandatory** to confirm the severity and location of coronary artery disease (CAD) and guide revascularization (PCI or CABG).   **Aspirin 81 mg once/day**   - **Rationale**: Aspirin is a cornerstone of CAD management to reduce thrombotic risk. The 81 mg dose is standard for chronic therapy (lower bleeding risk than 325 mg).   **Metoprolol 25 mg twice daily**   - **Rationale**: Beta-blockers reduce myocardial oxygen demand, improve angina symptoms, and are recommended in stable CAD.   **Nitroglycerin 0.4 mg sublingual as needed for chest pain**   - **Rationale**: Provides rapid symptom relief for angina episodes.   **Serum lipid profile**   - **Rationale**: To assess LDL cholesterol and guide statin therapy intensity (e.g., high-intensity statin if not already prescribed).   **Advise the patient to rest**   - **Rationale**: Avoid exertion until revascularization is performed to prevent triggering ischemia.   **Why Other Options Are Less Appropriate:**   - **Refer to a cardiothoracic surgeon for CABG**:   Premature without coronary angiography results. CABG is reserved for left main disease, multivessel CAD, or complex anatomy, which angiography must first confirm.   - **Aspirin 325 mg once/day**:   Higher doses (325 mg) are used in **acute coronary syndromes**, not stable angina. Chronic 81 mg is preferred for long-term safety.   - **Reinforce need to adhere to antihypertensive treatment**:   While important, this is not the **immediate priority** unless the patient has documented hypertension with poor control.  **Key Point**: This patient requires **urgent coronary angiography** and admission for stabilization. Medical therapy (aspirin, beta-blocker, nitroglycerin) and risk-factor modification (lipid profile) are critical while awaiting revascularization. | **Most Appropriate Next Steps:**   1. **Admit to the hospital telemetry unit**   Rationale: The large reversible LAD perfusion defect indicates **high-risk ischemia**, warranting inpatient monitoring for potential progression to acute coronary syndrome (ACS) or arrhythmias.   1. **Refer to a cardiologist for cardiac catheterization**   Rationale: Cardiac catheterization is **urgently needed** to define coronary anatomy, assess stenosis severity, and guide revascularization (PCI or CABG).   1. **Reinforce need to adhere to antihypertensive treatment**   Rationale: Hypertension exacerbates coronary disease and left ventricular hypertrophy (LVH). Tight blood pressure control (target <130/80 mmHg) is critical for reducing cardiac risk.   1. **Serum lipid profile**   Rationale: To assess LDL cholesterol and guide statin therapy (e.g., high-intensity statin to target LDL <70 mg/dL).   1. **Aspirin 81 mg once/day**   Rationale: Low-dose aspirin is standard for **secondary prevention** in stable angina. Avoid 325 mg (reserved for ACS or post-PCI).   1. **Metoprolol 25 mg twice daily**   Rationale: Beta-blockers reduce myocardial oxygen demand, improve symptoms, and are first-line therapy for angina.   1. **Nitroglycerin 0.4 mg sublingual as needed for chest pain**   Rationale: Provides immediate symptom relief for angina episodes.  **Why Other Options Are Less Appropriate:**   - **Advise the patient to rest**: While rest is important during angina episodes, this is part of general counseling, not a definitive next step. - **Refer to a cardiothoracic surgeon for CABG**: CABG referral occurs **after** angiography confirms surgical anatomy (e.g., left main disease, multivessel CAD). Premature without catheterization. - **Aspirin 325 mg once/day**: High-dose aspirin is unnecessary in stable angina and increases bleeding risk.   **Key Considerations:**   - The reversible LAD defect signifies **critical, flow-limiting stenosis** requiring prompt revascularization to prevent infarction. - Aggressive medical therapy (aspirin, beta-blocker, statin) and risk factor modification (hypertension, lipids) are essential alongside invasive evaluation. - Delaying catheterization risks destabilization of plaque and progression to ACS.   **Immediate Action Plan:**   1. **Inpatient admission** with telemetry monitoring. 2. **Urgent cardiac catheterization** within 24-48 hours. 3. **Initiate medical therapy** (aspirin 81 mg, metoprolol, high-intensity statin, nitroglycerin). 4. **Follow lipid profile results** to titrate statin dose. |
| Case3 |  |  |  |
| Diff | **Based on the patient’s presentation, the following differential diagnoses should not be excluded at this time:**   1. **Hypothyroidism:**   **The patient’s symptoms (fatigue, cold intolerance, weight gain, constipation, dry skin, coarse hair, periorbital edema, bradycardia, delayed reflexes, and depression) are classic for hypothyroidism. This is the most likely diagnosis.**   1. **Anemia:**   **Fatigue, pallor, and lethargy could be due to anemia, especially given her history of atrial fibrillation (potential for gastrointestinal bleeding due to anticoagulation with warfarin).**   1. **Chronic Kidney Disease (CKD):**   **Fatigue, edema, and constipation could be consistent with CKD. However, her blood pressure is controlled, and there is no mention of elevated creatinine or proteinuria.**   1. **Heart Failure:**   **Exertional dyspnea, fatigue, and peripheral edema could suggest heart failure, particularly given her history of atrial fibrillation and prior acute left ventricular failure.**   1. **Hyponatremia:**   **Fatigue, lethargy, and confusion can occur with hyponatremia, which may be secondary to hypothyroidism or other causes (e.g., diuretic use with furosemide).**  **Why Other Diagnoses Are Less Likely:**   1. **Alzheimer Disease:**   **While the patient has memory issues, her cognitive decline is relatively recent (4 months) and accompanied by systemic symptoms (e.g., fatigue, cold intolerance, weight gain), making hypothyroidism a more likely cause.**   1. **Chronic Bilateral Subdural Hematoma:**   **There is no history of trauma or focal neurologic deficits to suggest this diagnosis.**   1. **Parkinson Disease:**   **The patient has no tremor, rigidity, or bradykinesia (other than generalized slowing due to fatigue).**   1. **Stroke:**   **There are no focal neurologic deficits (e.g., weakness, sensory loss, or visual changes) to suggest a stroke.**  **Final Answer:**   - **Hypothyroidism** - **Anemia** - **Chronic Kidney Disease** - **Heart Failure** - **Hyponatremia**   **These diagnoses should be investigated further with appropriate tests (e.g., thyroid function tests, CBC, renal function tests, electrolytes, and echocardiography if heart failure is suspected).** | **The following differential diagnoses should not be excluded at this time:**   1. **Hypothyroidism**    - Strongly supported by symptoms (fatigue, weight gain, cold intolerance) and signs (bradycardia, hypothermia, delayed reflexes, hair/skin changes). 2. **Heart failure**    - History of AF with heart failure, current bibasilar crackles, leg edema, and exertional dyspnea suggest possible exacerbation. 3. **Hyponatremia**    - Hypothyroidism can cause SIADH-induced hyponatremia, which may contribute to lethargy/cognitive changes. 4. **Anemia**    - Fatigue, pallor, and hypothyroidism-associated anemia (e.g., normocytic or macrotic) are plausible without CBC results. 5. **Chronic kidney disease (CKD)**    - Edema, constipation, and hypertension could overlap with CKD; requires creatinine/eGFR evaluation.   **Rationale for Excluding Others:**   - **Alzheimer disease**: Rapid symptom onset (4 months) and systemic signs favor metabolic causes over neurodegenerative dementia. - **Chronic bilateral subdural hematoma**: No history of trauma/focal neurologic deficits, though anticoagulation with warfarin warrants caution. - **Parkinson disease**: Bradykinesia and slow speech are nonspecific; absence of rigidity/tremor makes this less likely. - **Stroke**: No focal deficits reported; global cognitive changes align better with hypothyroidism.   **Next Steps:**   - Confirm hypothyroidism with **TSH/free T4**, assess for anemia (CBC), hyponatremia (electrolytes), and CKD (creatinine, urinalysis). - Evaluate heart failure with **BNP**, chest X-ray, or echocardiography. - Consider neuroimaging (e.g., head CT) only if focal deficits emerge or cognitive decline persists despite treatment.   **Final Answer:** Hypothyroidism, Heart failure, Hyponatremia, Anemia, Chronic kidney disease. | **Differential Diagnoses That Should Not Be Excluded:**   1. **Hypothyroidism**   **Rationale:** This is the **most likely diagnosis** given the constellation of symptoms (lethargy, weight gain, cold intolerance, dry skin, constipation, bradycardia, delayed reflexes, proximal muscle weakness, periorbital edema) and physical findings (goiter, hoarse voice). Hypothyroidism explains nearly all systemic and neurologic manifestations.   1. **Chronic Kidney Disease (CKD)**   **Rationale:** Edema, fatigue, and constipation can occur in CKD. While no overt signs of renal dysfunction (e.g., hypertension, hematuria) are noted, a serum creatinine, BUN, and urinalysis are necessary to exclude renal pathology.   1. **Heart Failure**   **Rationale:** Lower extremity edema, exertional dyspnea, and fatigue overlap with heart failure. However, the absence of orthopnea, paroxysmal nocturnal dyspnea, or elevated jugular venous pressure makes this less likely. An echocardiogram or BNP level may help differentiate.   1. **Hyponatremia**   **Rationale:** Lethargy, confusion, and neurologic symptoms can result from hyponatremia, which is common in hypothyroidism due to impaired free water excretion. A basic metabolic panel should be checked.   1. **Anemia**   **Rationale:** Fatigue and cognitive slowing may be caused by anemia (e.g., iron deficiency or pernicious anemia, which can coexist with autoimmune thyroid disease). A CBC is warranted.  **Differential Diagnoses That Can Likely Be Excluded:**   - **Alzheimer Disease**: Rapid progression (4 months) and systemic symptoms (weight gain, cold intolerance) are atypical for Alzheimer’s. - **Parkinson Disease**: Absence of tremor, rigidity, or postural instability, along with systemic signs, makes this unlikely. - **Chronic Bilateral Subdural Hematoma**: No history of trauma or focal neurologic deficits. - **Stroke**: Gradual onset and lack of acute focal deficits argue against stroke.   **Key Workup:**   - **Thyroid Function Tests (TSH, free T4)** to confirm hypothyroidism. - **Electrolytes** (sodium), **CBC**, **renal function tests**, **BNP**, and **echocardiogram** to evaluate for hyponatremia, anemia, CKD, or heart failure. - **Imaging** (e.g., brain MRI) only if neurologic findings worsen or thyroid testing is normal.   **Conclusion:** While **hypothyroidism** is the leading diagnosis, CKD, heart failure, hyponatremia, and anemia should remain on the differential until ruled out with laboratory testing. Alzheimer’s, Parkinson’s, subdural hematoma, and stroke are less likely based on clinical presentation. |
| Diag1 | **Based on the patient’s presentation and the differential diagnoses under consideration, the most appropriate tests to order at this time are:**   1. **Thyroid Function Tests:**   **TSH, free T4, and anti-thyroid peroxidase (anti-TPO) antibodies to confirm hypothyroidism and assess for Hashimoto's thyroiditis.**   1. **Serum Electrolytes, BUN, and Creatinine:**   **To evaluate for hyponatremia (common in hypothyroidism) and assess renal function (to rule out chronic kidney disease).**   1. **Complete Blood Count (CBC):**   **To check for anemia, which could explain fatigue and pallor, especially given her use of warfarin (risk of gastrointestinal bleeding).**   1. **Electrocardiogram (ECG):**   **To assess for bradycardia, atrial fibrillation, and other potential cardiac abnormalities (e.g., low voltage, prolonged QT interval) that may occur in hypothyroidism.**   1. **Urinalysis:**   **To screen for proteinuria or other abnormalities that could suggest chronic kidney disease.**  **Why Not the Others?**   - **Pulmonary Function Tests:**   **Not indicated unless there is evidence of primary lung disease (e.g., obstructive or restrictive pattern). Her exertional dyspnea is more likely due to cardiac or systemic causes.**   - **Chest X-ray:**   **Not immediately necessary unless there are signs of heart failure (e.g., cardiomegaly, pulmonary edema) or pulmonary disease (e.g., infection, effusion).**   - **CT Scan of the Head:**   **Not indicated without focal neurologic deficits or signs of increased intracranial pressure.**   - **CT Scan of the Lungs with IV Contrast:**   **Not indicated unless there is suspicion of pulmonary embolism or other lung pathology (e.g., malignancy).**   - **MRI of the Head and Neck with IV Contrast:**   **Not indicated without focal neurologic findings or suspicion of structural abnormalities (e.g., tumor, stroke).**  **Final Answer:**   - **Thyroid Function Tests** - **Serum Electrolytes, BUN, and Creatinine** - **Complete Blood Count (CBC)** - **Electrocardiogram (ECG)** - **Urinalysis**   **These tests will help confirm the diagnosis of hypothyroidism, evaluate for anemia, assess renal function, and rule out other systemic causes of her symptoms.** | **The most appropriate tests to order at this time are:**   1. **Thyroid function tests (TSH and free T4)**    - **Critical** to confirm suspected hypothyroidism, given classic symptoms (fatigue, cold intolerance, weight gain) and signs (bradycardia, delayed reflexes, goiter). 2. **Serum electrolyte, BUN, and creatinine concentrations**    - Evaluate for **hyponatremia** (common in hypothyroidism) and assess renal function (to rule out CKD or prerenal azotemia from heart failure). 3. **Complete blood count (CBC)**    - Check for **anemia** (hypothyroidism-associated normocytic/macrocytic anemia) and infection. 4. **Electrocardiogram (ECG)**    - Confirm atrial fibrillation (known history), assess bradycardia, and rule out ischemia or conduction abnormalities (e.g., prolonged QT interval in hypothyroidism). 5. **Chest x-ray**    - Evaluate for pulmonary edema (heart failure exacerbation) or pleural effusions (hypothyroidism-associated). 6. **Urinalysis**    - Assess for proteinuria (CKD) or other renal abnormalities.   **Tests to Avoid at This Stage:**   - **CT scan of head**: No focal neurologic deficits or head trauma to justify immediate imaging. - **Pulmonary function tests/CT scan of lungs**: Dyspnea is more likely cardiac or metabolic (e.g., anemia, heart failure) than primary pulmonary. - **MRI of head/neck**: Unnecessary without focal neurologic signs or suspicion of structural pathology.   **Rationale:**   - **Hypothyroidism** is the leading diagnosis, but comorbidities (heart failure, anemia, CKD) must be evaluated. - **Hyponatremia** and **anemia** are common complications of hypothyroidism and contribute to cognitive/mood symptoms. - **ECG** and **chest x-ray** address cardiac risks (AF, heart failure) exacerbated by hypothyroidism.   **Next Steps:**   - Start levothyroxine if hypothyroidism is confirmed. - Address electrolyte abnormalities, anemia, or heart failure based on test results. - Reassess cognitive symptoms after thyroid normalization. | **Most Appropriate Tests to Order:**   1. **Thyroid function tests (TSH, free T4)**   **Rationale:** Critical to confirm suspected hypothyroidism, which aligns with symptoms (lethargy, cold intolerance, weight gain, bradycardia, delayed reflexes) and physical findings (goiter, periorbital edema).   1. **Serum electrolytes, BUN, and creatinine concentrations**   **Rationale:**  Hyponatremia is common in hypothyroidism due to impaired free water excretion.  Assess renal function to evaluate for chronic kidney disease (CKD), which can mimic or coexist with hypothyroidism (edema, fatigue).   1. **Complete blood count (CBC)**   **Rationale:** Fatigue and cognitive slowing may be caused by anemia (e.g., iron deficiency or pernicious anemia, which can occur with autoimmune thyroid disease).   1. **Urinalysis**   **Rationale:** Helps evaluate for proteinuria or other signs of renal dysfunction if CKD is suspected.   1. **Electrocardiogram (ECG)**   **Rationale:**  Bradycardia (56 bpm) and irregularly irregular rhythm (possible atrial fibrillation) require evaluation.  Hypothyroidism can cause pericardial effusion or arrhythmias.   1. **Chest x-ray**   **Rationale:**  Assess for cardiomegaly, pulmonary congestion, or pleural effusion to evaluate for heart failure, which can overlap with hypothyroidism symptoms (edema, dyspnea).  **Tests That Are Less Likely Necessary at This Stage:**   - **CT scan of the head**: No focal neurologic deficits or trauma history to suggest subdural hematoma/stroke. Gradual cognitive decline is more consistent with metabolic causes (e.g., hypothyroidism). - **MRI of the head and neck with IV contrast**: No indication for tumor or compressive lesion (e.g., hoarseness is more likely due to thyroid enlargement or myxedema). - **Pulmonary function tests**: Exertional dyspnea is more likely due to cardiac or systemic metabolic dysfunction rather than primary pulmonary disease. - **CT scan of the lungs with IV contrast**: No clinical suspicion for pulmonary embolism or malignancy.   **Key Priorities:**   1. Confirm hypothyroidism with **TSH/free T4**. 2. Rule out CKD, hyponatremia, and anemia with **electrolytes, BUN, creatinine, CBC, and urinalysis**. 3. Evaluate cardiac status with **ECG and chest x-ray** given bradycardia and edema.   **Next Steps if Initial Tests Are Negative:**   - Consider echocardiogram (if heart failure is still suspected). - Measure BNP (not listed but relevant for heart failure). - Check thyroid antibodies (anti-TPO) if autoimmune thyroiditis is confirmed. |
| Dx1 | **he most likely diagnosis for this patient is:**  **Hypothyroidism**  **Rationale:**   - **The patient’s severely elevated TSH (88 mIU/L) and low free T4 (5.15 pmol/L) and free T3 (1.2 pmol/L) confirm primary hypothyroidism.** - **Her symptoms (fatigue, cold intolerance, weight gain, constipation, dry skin, coarse hair, periorbital edema, bradycardia, delayed reflexes, and depression) are classic for hypothyroidism.** - **The normocytic anemia (Hb 9.8 g/dL) and mild hyponatremia (Na 135 mmol/L) are also consistent with hypothyroidism.**   **Why Not the Others?**   1. **Chronic Fatigue Syndrome:**   **A diagnosis of exclusion, and the patient has clear biochemical evidence of hypothyroidism.**   1. **Chronic Kidney Disease (CKD):**   **Renal function is normal (creatinine 0.9 mg/dL, BUN 8 mg/dL), and there is no proteinuria or other signs of CKD.**   1. **Chronic Subdural Hematoma:**   **The head CT is normal, and there is no history of trauma or focal neurologic deficits.**   1. **Hyponatremia:**   **While present, it is mild and secondary to hypothyroidism, not a primary diagnosis.**   1. **Iron Deficiency Anemia:**   **The anemia is normocytic (MCV 88 fL), not microcytic, making iron deficiency less likely.**   1. **Other Conditions (e.g., heart failure, Alzheimer disease):**   **These are either ruled out by testing (e.g., normal head CT) or explained by hypothyroidism (e.g., edema, cognitive slowing).**  **Final Answer:**   - **Hypothyroidism**   **This diagnosis explains all the patient’s symptoms and laboratory abnormalities, and it requires immediate treatment with levothyroxine.** | **Hypothyroidism**  **Rationale:**   - **Lab Confirmation**: Severely elevated TSH (88 mIU/L) and low free T4 (0.4 ng/dL) confirm **primary hypothyroidism** as the underlying diagnosis. - **Clinical Correlation**: Symptoms (fatigue, cold intolerance, weight gain, constipation, cognitive slowing) and signs (bradycardia, delayed reflexes, periorbital edema) are classic for hypothyroidism. - **Exclusion of Other Options**:   **Chronic fatigue syndrome**: Not a diagnosis of exclusion; lab abnormalities (e.g., TSH) explain symptoms.  **Chronic kidney disease**: Normal BUN/creatinine rule this out.  **Chronic subdural hematoma**: Normal head CT excludes this.  **Hyponatremia**: Secondary to hypothyroidism (SIADH).  **Iron deficiency anemia**: Normocytic anemia (MCV 88 fL) aligns with hypothyroidism, not iron deficiency (typically microcytic).  **Conclusion:** **Hypothyroidism** is the definitive diagnosis, supported by biochemical and clinical evidence. All other findings (anemia, hyponatremia, heart failure) are secondary complications. | **Most Likely Diagnosis:** **Hypothyroidism**  **Key Supporting Evidence:**   1. **Severe TSH Elevation (88 mIU/L)** with **Low Free T4 (0.4 ng/dL)** and **Low Free T3 (0.8 pg/mL)**:   Classic lab findings of **primary hypothyroidism** (thyroid gland failure).   1. **Clinical Symptoms**:   Lethargy, cold intolerance, weight gain, bradycardia (56 bpm), delayed reflexes, periorbital edema, and goiter—all consistent with hypothyroidism.   1. **Complications Linked to Hypothyroidism**:   **Atrial fibrillation** (ECG finding): Hypothyroidism predisposes to arrhythmias.  **Hyponatremia (Na 135 mmol/L)**: Due to impaired free water excretion.  **Normocytic anemia (Hb 9.8 g/dL)**: Anemia of chronic disease caused by hypothyroidism.  **Enlarged cardiac silhouette on chest x-ray**: Likely pericardial effusion, a known complication.  **Why Other Options Are Less Likely:**   - **Chronic fatigue syndrome**: Does not explain lab abnormalities (e.g., elevated TSH, hyponatremia). - **Chronic kidney disease (CKD)**: Normal BUN (8 mg/dL) and creatinine (0.9 mg/dL) rule this out. - **Chronic subdural hematoma**: Normal head CT scan excludes this. - **Hyponatremia**: A consequence of hypothyroidism, not the primary diagnosis. - **Iron deficiency anemia**: Normocytic anemia (MCV 88 fL) is inconsistent with iron deficiency (typically microcytic).   **Conclusion**: Hypothyroidism is the unifying diagnosis explaining all clinical and lab findings. |
| Diag2 | **The most appropriate next steps for this patient, who has been diagnosed with primary hypothyroidism, include:**   1. **Serum Thyroid Peroxidase (TPO) Antibodies:**   **To confirm autoimmune thyroiditis (Hashimoto's thyroiditis) as the underlying cause of hypothyroidism. This is the most common cause of primary hypothyroidism in iodine-sufficient regions.**   1. **Thyroid Ultrasound:**   **To evaluate the thyroid gland for structural abnormalities (e.g., diffuse enlargement, nodules, or heterogeneity) that are typical of Hashimoto's thyroiditis.**  **Why Not the Others?**   1. **Pituitary Hormone Profile (LH, FSH, Cortisol, Prolactin):**   **Not necessary in this case because the elevated TSH and low free T4 confirm primary hypothyroidism (thyroid gland failure), not secondary (pituitary) or tertiary (hypothalamic) hypothyroidism.**   1. **Serum C-Reactive Protein (CRP):**   **Not indicated unless there is suspicion of an acute inflammatory or infectious process, which is not suggested by the patient’s presentation.**  **Additional Considerations:**   - **Initiate Levothyroxine Therapy:**   **Start levothyroxine at a low dose (e.g., 25–50 mcg/day) and titrate based on TSH levels, especially given the patient’s age and history of atrial fibrillation.**   - **Monitor for Improvement:**   **Reassess symptoms (e.g., fatigue, cold intolerance, constipation) and repeat thyroid function tests (TSH, free T4) in 6–8 weeks.**   - **Address Anemia:**   **Investigate the cause of normocytic anemia (e.g., check ferritin, vitamin B12, and folate levels) and treat accordingly.**  **Final Answer:**   - **Serum Thyroid Peroxidase (TPO) Antibodies** - **Thyroid Ultrasound**   **These steps will help confirm the etiology of hypothyroidism and guide long-term management.** | The **most appropriate next steps** are:  **✅ Serum Thyroid Peroxidase (TPO) Antibodies**   - **Rationale**:   **Primary hypothyroidism** with elevated TSH and low T4/T3 is most commonly caused by **autoimmune thyroiditis (Hashimoto's disease)**.  TPO antibodies confirm autoimmune etiology, guiding long-term management (e.g., monitoring for other autoimmune conditions like celiac disease or Addison's).  **✅ Thyroid Ultrasound**   - **Rationale**:   Evaluates thyroid gland structure (e.g., **heterogeneity, atrophy, or goiter**) typical of Hashimoto’s.  Rules out structural abnormalities (e.g., nodules requiring biopsy for malignancy).  **❌ Pituitary Hormone Profile (LH, FSH, cortisol, prolactin)**   - **Rationale**:   **Not indicated** here. Elevated TSH confirms **primary hypothyroidism** (thyroid failure). Pituitary dysfunction (secondary hypothyroidism) would present with **low/normal TSH** and low T4.  No clinical signs of panhypopituitarism (e.g., hypotension, hypoglycemia, menstrual irregularities).  **❌ Serum C-reactive Protein (CRP)**   - **Rationale**:   No evidence of acute inflammation (normal leukocytes, no fever).  Anemia and thrombocytopenia are more likely due to **hypothyroidism** (reduced erythropoiesis) or chronic disease rather than inflammation.  **Key Takeaways:**   1. **Autoimmune Workup**: TPO antibodies are critical to confirm Hashimoto’s (accounts for >90% of hypothyroidism cases in iodine-sufficient areas). 2. **Thyroid Ultrasound**: Supports autoimmune thyroiditis (e.g., heterogeneous gland) and excludes nodules/malignancy. 3. **Avoid Unnecessary Tests**:   Pituitary testing is redundant with elevated TSH.  CRP adds no value without signs of infection/inflammation.  **Next Steps After Diagnosis**:   - Start **levothyroxine** replacement (titrate dose based on TSH). - Address anemia (check iron, B12, folate). - Monitor cardiac complications (echocardiogram for pericardial effusion, manage atrial fibrillation). | **he most appropriate next steps are:**   1. **Serum thyroid peroxidase (TPO) antibodies** 2. **Thyroid ultrasound**   **Rationale:**   1. **TPO Antibodies**:   **Purpose**: Confirm autoimmune thyroiditis (Hashimoto’s disease), the most common cause of primary hypothyroidism.  **Importance**: Guides long-term management (e.g., monitoring for other autoimmune conditions like celiac disease or type 1 diabetes).   1. **Thyroid Ultrasound**:   **Purpose**: Evaluate thyroid anatomy for features of Hashimoto’s (e.g., heterogeneous echotexture, goiter) and rule out nodules or malignancy.  **Relevance**: The patient has a goiter (clinical finding), and ultrasound helps assess structural abnormalities.  **Why Other Options Are Less Urgent:**   - **Pituitary hormone profile (LH, FSH, cortisol, prolactin)**:   Unnecessary here because **TSH is markedly elevated**, confirming **primary hypothyroidism** (thyroid gland failure). Secondary (pituitary) hypothyroidism would show low/normal TSH with low T4.  Exception: If adrenal insufficiency is suspected (e.g., hypotension, hyperpigmentation), cortisol could be checked, but no such symptoms are described.   - **Serum CRP**:   Not indicated unless infection or acute inflammation is suspected (e.g., subacute thyroiditis). Chronic autoimmune thyroiditis (Hashimoto’s) does not typically elevate CRP.  **Conclusion**: TPO antibodies and thyroid ultrasound are critical to confirm the autoimmune etiology and assess thyroid structure. Other tests are not immediately warranted based on the current data. |
| Dx2 | **The most likely diagnosis for this patient is:**  **Hashimoto Thyroiditis**  **Rationale:**   - **The patient has primary hypothyroidism (elevated TSH, low free T4, and low free T3).** - **The elevated serum thyroid peroxidase (TPO) antibodies (258 IU/mL) confirm autoimmune thyroiditis (Hashimoto's thyroiditis) as the underlying cause.** - **Her clinical presentation (fatigue, cold intolerance, weight gain, constipation, dry skin, coarse hair, periorbital edema, bradycardia, delayed reflexes, and depression) is classic for hypothyroidism due to Hashimoto's thyroiditis.**   **Why Not the Others?**   1. **Hypothyroidism Due to Iodine Deficiency:**   **Unlikely in iodine-sufficient regions (e.g., the United States). Additionally, iodine deficiency typically presents with a diffuse goiter and is not associated with elevated TPO antibodies.**   1. **Euthyroid Sick Syndrome:**   **This condition occurs in critically ill patients and is characterized by low T3, normal or low TSH, and normal or low T4. The patient’s elevated TSH and clinical presentation are inconsistent with this diagnosis.**   1. **Simple Nontoxic Goiter:**   **This condition is characterized by thyroid enlargement without thyroid dysfunction (normal TSH, T4, and T3) and is not associated with elevated TPO antibodies.**  **Final Answer:**   - **Hashimoto Thyroiditis**   **This diagnosis explains the patient’s hypothyroidism, elevated TPO antibodies, and clinical symptoms. Treatment with levothyroxine is the next step.** | **Most Likely Diagnosis:**  **✅ Hashimoto Thyroiditis**  **Rationale**:   1. **Positive TPO Antibodies (258 IU/mL)**:   **Strongly diagnostic** for autoimmune thyroiditis (levels >100 IU/mL are highly specific for Hashimoto’s).  Autoimmune destruction of the thyroid gland leads to hypothyroidism.   1. **Clinical and Lab Context**:   **Primary hypothyroidism** (elevated TSH, low T4/T3) aligns with Hashimoto’s progression.  Anemia and thrombocytopenia are common extra-thyroidal manifestations of autoimmune thyroiditis.   1. **Exclusion of Alternatives**:   **Hypothyroidism due to iodine deficiency**: Unlikely in iodine-sufficient regions (e.g., most developed countries). No history of dietary iodine restriction.  **Euthyroid sick syndrome**: Occurs in critically ill patients with normal/low TSH and low T3; inconsistent with elevated TSH.  **Simple nontoxic goiter**: Does not cause hypothyroidism or elevated TPO antibodies.  **Why Not the Other Options?**   - **Hypothyroidism due to iodine deficiency**:   Rare in areas with iodized salt; no goiter or iodine deficiency risk factors mentioned.  TPO antibodies are negative in iodine deficiency.   - **Euthyroid sick syndrome**:   Typically occurs during acute illness, surgery, or starvation.  TSH is normal/low (not elevated), and TPO antibodies are absent.   - **Simple nontoxic goiter**:   Presents with thyroid enlargement but normal thyroid function (euthyroid) and no autoantibodies.  **Conclusion**: The combination of **hypothyroidism**, **elevated TPO antibodies**, and **anemia** confirms **Hashimoto thyroiditis** as the definitive diagnosis. | **Most Likely Diagnosis:** **Hashimoto Thyroiditis**  **Key Supporting Evidence:**   1. **Elevated TPO Antibodies (258 IU/mL)**:   **>100 IU/mL** is strongly indicative of **autoimmune thyroiditis** (Hashimoto’s).  TPO antibodies are present in >95% of Hashimoto’s cases.   1. **Clinical Context**:   The patient has **primary hypothyroidism** (elevated TSH) and a **palpable goiter**, both classic features of Hashimoto’s.  **Why Other Diagnoses Are Unlikely:**   - **Hypothyroidism Due to Iodine Deficiency**:   Rare in iodine-sufficient regions (e.g., most developed countries).  TPO antibodies are **not elevated** in iodine deficiency.   - **Euthyroid Sick Syndrome**:   Occurs in critically ill patients with **normal or low TSH**, not elevated TSH.  No evidence of acute illness in this case.   - **Simple Nontoxic Goiter**:   Thyroid enlargement **without hypothyroidism** (TSH would be normal).  TPO antibodies are typically absent.  **Conclusion**: The combination of **hypothyroidism**, **goiter**, and **elevated TPO antibodies** confirms **Hashimoto thyroiditis** as the definitive diagnosis. |
| Mang | **The management of Hashimoto thyroiditis involves levothyroxine (L-thyroxine) replacement therapy to normalize thyroid function. Given the patient’s age, comorbidities (e.g., atrial fibrillation), and the need to avoid over-replacement, the following is the most appropriate approach:**  **Most Appropriate Management Options:**   1. **L-thyroxine 25 mcg po once/day:**   **This is a low starting dose, which is appropriate for an elderly patient with a history of atrial fibrillation. It minimizes the risk of cardiac complications (e.g., exacerbation of arrhythmias or ischemic symptoms) due to rapid correction of hypothyroidism.**  **The dose can be gradually titrated upward (e.g., by 25 mcg every 4–6 weeks) based on TSH levels and clinical response.**   1. **L-thyroxine 75 mcg po once/day:**   **This is a moderate dose that may be appropriate for younger patients or those without cardiac comorbidities. However, for this elderly patient with atrial fibrillation, starting at 75 mcg is too high and could precipitate cardiac complications.**  **Why Not the Others?**   1. **Liothyronine (T3) 25 mcg bid:**   **Liothyronine is not typically used for long-term management of hypothyroidism. It has a short half-life, leading to fluctuating T3 levels, and increases the risk of cardiac side effects (e.g., tachycardia, arrhythmias).**   1. **L-thyroxine 150 mcg po once/day:**   **This is a full replacement dose and is too high for initial therapy, especially in an elderly patient with cardiac comorbidities. Starting at this dose could lead to overtreatment and adverse effects (e.g., atrial fibrillation exacerbation, osteoporosis, or cardiac ischemia).**   1. **Thyroid Extract:**   **Thyroid extract (desiccated thyroid) contains both T4 and T3 in non-physiological ratios. It is not recommended as first-line therapy due to the risk of T3 excess and associated side effects (e.g., palpitations, arrhythmias).**  **Additional Management Considerations:**   - **Monitor TSH and Clinical Response:**   **Recheck TSH and free T4 in 6–8 weeks after starting levothyroxine. Adjust the dose gradually to achieve a TSH within the normal range (typically 0.5–4.0 mIU/L).**   - **Address Anemia:**   **Investigate the cause of normocytic anemia (e.g., check ferritin, vitamin B12, and folate levels) and treat accordingly.**   - **Educate the Patient:**   **Advise the patient to take levothyroxine on an empty stomach (30–60 minutes before breakfast) and avoid calcium or iron supplements within 4 hours of taking the medication.**  **Final Answer:**   - **L-thyroxine 25 mcg po once/day** - **L-thyroxine 75 mcg po once/day (if starting cautiously in a younger, healthier patient, but not for this elderly patient with atrial fibrillation).**   **For this patient, L-thyroxine 25 mcg po once/day is the most appropriate starting dose.** | **Management of Hashimoto Thyroiditis:**  ✅ **L-thyroxine 75 mcg po once/day** ✅ **L-thyroxine 150 mcg po once/day**  **Rationale**:   1. **Levothyroxine (L-T4) is first-line therapy**:   Standard replacement dose = **1.6 mcg/kg/day** (adjusted for age, weight, and comorbidities).  **75 mcg/day**: Appropriate for patients weighing **~47 kg** (e.g., smaller adults or cautious titration).  **150 mcg/day**: Appropriate for patients weighing **~94 kg** or those requiring full replacement.   1. **Excluded Options**:   **Liothyronine (T3) 25 mcg bid**: Not recommended as monotherapy due to short half-life and risk of cardiac/osteoporotic complications.  **L-thyroxine 25 mcg/day**: Too low for most adults (unless for gradual titration in frail/elderly patients, which is not specified here).  **Thyroid extract**: Contains non-physiological T3:T4 ratios; not guideline-recommended for Hashimoto’s.  **Key Considerations**:   - **Dose adjustment**: Follow TSH levels every 6–8 weeks. - **Avoid overtreatment**: Target TSH in the normal range (0.5–4.0 mIU/L).   **Final Answer**: **L-thyroxine 75 mcg and 150 mcg once daily** are both appropriate, depending on patient weight and clinical context. | **Management of Hashimoto Thyroiditis:** The following options are appropriate:   1. **L-thyroxine 75 mcg po once/day** 2. **L-thyroxine 150 mcg po once/day**   **Rationale:**   - **Levothyroxine (L-thyroxine)** is the **first-line treatment** for hypothyroidism caused by Hashimoto’s. The dose is **weight-based** (1.6 mcg/kg/day).   **75 mcg/day**: Appropriate for a ~**47 kg** patient.  **150 mcg/day**: Appropriate for a ~**94 kg** patient.   - Doses should be adjusted based on **TSH monitoring** (target TSH: 0.5–4.0 mIU/L).   **Why Other Options Are Not Recommended:**   - **Liothyronine (T3) 25 mcg bid**:   T3 monotherapy is **not standard** due to short half-life and risk of cardiac arrhythmias.   - **L-thyroxine 25 mcg/day**:   Too low for most adults (unless the patient is very small or has severe cardiac disease).   - **Thyroid Extract**:   Contains non-physiological ratios of T4/T3 and is **not recommended** as first-line therapy.  **Next Steps**: Start levothyroxine at a **weight-based dose** (e.g., 75 mcg or 150 mcg) and recheck TSH in 6–8 weeks. Avoid T3 monotherapy or thyroid extract. |
| Case4 |  |  |  |
| Diff | **Based on the patient’s presentation and the need to prioritize life-threatening or common conditions, the following differential diagnoses cannot be excluded at this time:**   1. **Seizures:**   **The patient’s episode of confusion, amnesia, tongue laceration, bilateral arm pain, and urinary incontinence are highly suggestive of a generalized tonic-clonic seizure.**   1. **Acute Stroke:**   **Stroke can present with confusion, focal neurological deficits, or altered mental status. Although the patient has no focal deficits on exam, imaging is necessary to rule out stroke.**   1. **Subarachnoid Hemorrhage (SAH):**   **SAH can cause sudden confusion, headache, and neurological deficits. While the patient has no severe headache or neck stiffness, SAH cannot be excluded without imaging.**   1. **Subdural Hematoma:**   **Subdural hematoma can present with confusion, especially in elderly patients, even without a history of trauma. Imaging is required to rule this out.**   1. **Metabolic Encephalopathy:**   **Conditions such as hypoglycemia, hyponatremia, or uremia can cause confusion and altered mental status. Laboratory tests are needed to evaluate for metabolic abnormalities.**   1. **Syncope:**   **Syncope can cause transient loss of consciousness and confusion. However, it does not typically explain the tongue laceration or urinary incontinence.**  **Why Not the Others?**   1. **Alcohol Intoxication:**   **The patient consumed only 2–3 glasses of wine, which is unlikely to cause significant intoxication or amnesia.**   1. **Alcohol Withdrawal:**   **The patient does not have a history of heavy alcohol use or other withdrawal symptoms (e.g., tremors, hallucinations).**   1. **Drug Encephalopathy:**   **There is no history of drug use or exposure to suggest this diagnosis.**   1. **Meningitis:**   **The patient has no fever, headache, or neck stiffness, making meningitis unlikely.**   1. **Dementia:**   **Dementia is a chronic condition and would not explain the acute episode of confusion and amnesia.**  **Final Answer:**   - **Seizures** - **Acute Stroke** - **Subarachnoid Hemorrhage (SAH)** - **Subdural Hematoma** - **Metabolic Encephalopathy** - **Syncope**   **These diagnoses require further evaluation with brain imaging (CT or MRI), EEG, and laboratory tests to confirm or exclude them.** | **Differential Diagnoses That Cannot Be Excluded:**  ✅ **Acute stroke** ✅ **Subarachnoid hemorrhage (SAH)** ✅ **Subdural hematoma** ✅ **Metabolic encephalopathy** ✅ **Syncope**  **Rationale**:   1. **Acute Stroke**:   **Why**: Subtle strokes (e.g., posterior circulation, non-dominant hemisphere) may lack focal deficits initially.  **Risk**: Elderly patients with vascular risk factors.  **Exclusion Requires**: **Neuroimaging (CT/MRI)**.   1. **Subarachnoid Hemorrhage (SAH)**:   **Why**: Can present with seizures, confusion, and headache (though headache is absent here).  **Risk**: Sudden-onset neurologic symptoms.  **Exclusion Requires**: **Non-contrast CT head** ± lumbar puncture if CT negative.   1. **Subdural Hematoma**:   **Why**: History of possible trauma (e.g., from "tossing around in bed" or a fall). Elderly patients are at high risk due to cerebral atrophy.  **Exclusion Requires**: **CT head**.   1. **Metabolic Encephalopathy**:   **Why**: Electrolyte imbalances (e.g., hyponatremia, hypercalcemia), hypoglycemia, or uremia can mimic seizures or confusion.  **Exclusion Requires**: **Basic metabolic panel, glucose, calcium, LFTs**.   1. **Syncope**:   **Why**: Convulsive syncope can mimic seizures with post-event confusion and injury (e.g., tongue laceration).  **Risk**: Cardiac arrhythmia or orthostatic hypotension in the elderly.  **Exclusion Requires**: **ECG, cardiac monitoring, orthostatic vitals**.  **Excluded or Less Likely Diagnoses:**   - **Alcohol Intoxication/Withdrawal**: Unlikely given her reported low alcohol intake and resolution of symptoms by ED arrival. - **Drug Encephalopathy**: No history of medication/drug use to support this. - **Meningitis**: No fever, neck stiffness, or systemic signs. - **Dementia**: Chronic condition; acute episode is inconsistent with baseline.   **Key Next Steps**:   - **Neuroimaging (CT head)** to rule out SAH, stroke, subdural. - **Metabolic labs** to exclude electrolyte/glucose abnormalities. - **EEG** if seizure is suspected despite normal imaging. - **Cardiac workup** (ECG, telemetry) for syncope evaluation.   **Final Answer**: **Acute stroke, SAH, subdural hematoma, metabolic encephalopathy, and syncope** remain critical differentials requiring further testing. | **Critical Differential Diagnoses That Cannot Be Excluded Without Further Testing:**   1. **Acute Stroke**   Posterior circulation stroke or transient ischemic attack (TIA) can present with acute confusion, amnesia, or seizures without focal deficits. Neuroimaging (CT/MRI) is required to exclude.   1. **Subarachnoid Hemorrhage (SAH)**   Though classic symptoms (thunderclap headache, neck stiffness) are absent, SAH can manifest as seizures or altered mental status. Non-contrast CT head is essential.   1. **Subdural Hematoma**   Elderly patients are at risk due to cerebral atrophy and potential trauma (e.g., from a fall during the episode). Subtle presentations include confusion and neurologic deficits. CT head is diagnostic.   1. **Metabolic Encephalopathy**   Hypoglycemia, hyponatremia, or hypercalcemia could provoke seizures or confusion. Labs (electrolytes, glucose, calcium) are needed to exclude.   1. **Drug Encephalopathy**   Accidental ingestion, toxicity from supplements, or undiagnosed drug interactions could contribute. Toxicology screening is necessary.   1. **Seizures**   The presentation strongly suggests a generalized seizure, but secondary causes (e.g., structural brain lesion, metabolic derangement) must be ruled out.  **Why Other Options Are Less Likely or Excludable:**   - **Alcohol Intoxication/Withdrawal**: Unlikely given her low alcohol intake, normal mental status on exam, and timeline (symptoms persisted after alcohol metabolism). - **Meningitis**: No fever, neck stiffness, or systemic signs. Low probability in the absence of infection markers. - **Syncope**: Less likely due to features specific to seizures (tongue laceration, postictal confusion). - **Dementia**: Acute confusion resolving to baseline is inconsistent with neurodegenerative dementia.   **Immediate Next Steps**:   - **Non-contrast CT head** to evaluate for stroke, SAH, subdural hematoma. - **Lab studies**: Electrolytes, glucose, calcium, CBC, toxicology screen. - **EEG** if seizure is suspected after initial workup.   **Conclusion**: While seizures are the leading diagnosis, life-threatening conditions like stroke, SAH, subdural hematoma, and metabolic/drug causes must be urgently excluded. |
| Diag1 | **Given the patient’s presentation and the differential diagnoses under consideration, the most appropriate steps to guide diagnosis and treatment are:**   1. **CT Scan of the Brain Without Contrast:**   **This is the first-line imaging study to rule out acute intracranial pathology, such as stroke, subarachnoid hemorrhage (SAH), subdural hematoma, or mass lesion.**   1. **Complete Metabolic Profile:**   **To evaluate for metabolic encephalopathy (e.g., hyponatremia, hypoglycemia, uremia) as a potential cause of confusion or seizure-like activity.**   1. **Electrocardiogram (ECG):**   **To assess for cardiac arrhythmias (e.g., atrial fibrillation, prolonged QT interval) that could cause syncope or seizure-like episodes.**   1. **Electroencephalography (EEG):**   **To evaluate for epileptiform activity and confirm the diagnosis of a seizure.**   1. **Complete Blood Count with Platelets and Differential:**   **To assess for infection, anemia, or other hematologic abnormalities that could contribute to the patient’s symptoms.**   1. **Serum Alcohol Level:**   **To rule out alcohol intoxication as a contributing factor, although the patient’s alcohol consumption was minimal.**   1. **Urine Toxicology Screen:**   **To rule out drug intoxication or exposure to substances that could cause confusion or seizures.**  **Why Not the Others?**   1. **CT Scan of the Brain with Contrast:**   **Contrast is not necessary for the initial evaluation of acute intracranial pathology (e.g., stroke, hemorrhage). It is typically reserved for evaluating tumors or infections, which are less likely in this case.**   1. **Erythrocyte Sedimentation Rate (ESR):**   **ESR is nonspecific and not helpful in the acute evaluation of confusion or seizures.**   1. **Serum Vitamin B12 Level:**   **While vitamin B12 deficiency can cause neurological symptoms, it is unlikely to present acutely with confusion and seizures. This can be checked later if initial workup is unrevealing.**   1. **Serum Calcium and Magnesium Levels:**   **These are included in the complete metabolic profile and do not need to be ordered separately unless there is a specific indication (e.g., prolonged QT interval on ECG).**  **Final Answer:**   - **CT Scan of the Brain Without Contrast** - **Complete Metabolic Profile** - **Electrocardiogram (ECG)** - **Electroencephalography (EEG)** - **Complete Blood Count with Platelets and Differential** - **Serum Alcohol Level** - **Urine Toxicology Screen**   **These steps will help identify the underlying cause of the patient’s symptoms and guide appropriate treatment.** | **Most Appropriate Diagnostic Steps:**  ✅ **CT scan of the brain without contrast** ✅ **Complete metabolic profile** ✅ **Electrocardiogram (ECG)** ✅ **Electroencephalography (EEG)** ✅ **Complete blood count (CBC) with platelets and differential** ✅ **Serum magnesium level** ✅ **Urine toxicology screen**  **Rationale**:   1. **CT scan of the brain without contrast**:   **Critical** to rule out acute stroke, subarachnoid hemorrhage (SAH), subdural hematoma, or other structural abnormalities. Contrast is unnecessary for detecting acute hemorrhage.   1. **Complete metabolic profile (CMP)**:   Evaluates electrolytes (sodium, potassium), glucose, renal/hepatic function, and calcium. Essential to identify metabolic encephalopathy (e.g., hyponatremia, hypercalcemia) or hypoglycemia.   1. **Electrocardiogram (ECG)**:   Assesses for arrhythmias (e.g., prolonged QT, atrial fibrillation) or ischemic changes that could explain syncope or seizure-like activity.   1. **Electroencephalography (EEG)**:   Required to evaluate for seizure activity, especially if initial imaging is normal. Helps distinguish epileptic seizures from non-epileptic mimics.   1. **Complete blood count (CBC)**:   Screens for infection, anemia, or thrombocytopenia, which may contribute to confusion or neurologic symptoms.   1. **Serum magnesium level**:   Hypomagnesemia can precipitate seizures and is not included in standard metabolic panels.   1. **Urine toxicology screen**:   Excludes drug-induced encephalopathy or seizures (e.g., cocaine, amphetamines) even if substance use is not reported.  **Excluded or Less Urgent Options:**   - **CT scan of the brain with contrast**: Not indicated for acute intracranial hemorrhage or stroke evaluation. - **ESR**: Non-specific and low yield without signs of inflammation or infection. - **Serum vitamin B12 level**: More relevant in chronic neurologic decline, not acute settings. - **Serum alcohol level**: Low suspicion based on history, but may still be considered if altered mentation persists. - **Serum calcium level**: Already included in the CMP; redundant as a separate test.   **Next Steps**:   - **Immediate neuroimaging** (non-contrast CT) followed by **EEG** if seizures are suspected. - **Metabolic labs** (CMP, magnesium) and **urine tox screen** to identify reversible causes. - **ECG** and **CBC** to assess cardiac and hematologic contributors.   **Final Answer**: **CT brain without contrast, CMP, ECG, EEG, CBC, serum magnesium, and urine toxicology screen** are essential to evaluate for stroke, hemorrhage, metabolic derangements, seizures, and toxicity. | **Most Appropriate Steps for Diagnosis and Treatment:**   1. **CT Scan of the Brain Without Contrast**   **Critical** to exclude acute intracranial pathology (e.g., stroke, subarachnoid hemorrhage, subdural hematoma). Non-contrast CT is the first-line imaging in acute settings.   1. **Complete Metabolic Profile**   Evaluates electrolytes (Na⁺, Ca²⁺, Mg²⁺), glucose, renal/hepatic function, and acid-base status to rule out metabolic encephalopathy or electrolyte imbalances as seizure triggers.   1. **Electrocardiogram (ECG)**   Assess for arrhythmias (e.g., atrial fibrillation, prolonged QT) that could cause syncope or seizure-like activity.   1. **Electroencephalography (EEG)**   Essential to detect epileptiform activity if seizure is suspected and to guide long-term management (e.g., need for antiseizure medication).   1. **Complete Blood Count (CBC) with Platelets and Differential**   Screens for infection, anemia, or thrombocytopenia, which may contribute to altered mental status or seizures.   1. **Urine Toxicology Screen**   Excludes drug-induced seizures (e.g., cocaine, amphetamines) or accidental ingestion of toxins.   1. **Serum Alcohol Level**   Confirms or excludes acute alcohol intoxication, which can lower the seizure threshold.   1. **Serum Calcium and Magnesium Levels**   Hypocalcemia or hypomagnesemia can provoke seizures; these are often included in metabolic panels but may need separate testing.  **Less Urgent or Lower Yield in Acute Setting:**   - **CT Scan of the Brain With Contrast**: Not first-line for acute evaluation; reserved for suspected tumors or infections after non-contrast CT. - **ESR**: Non-specific marker of inflammation; not urgent here without signs of infection/vasculitis. - **Serum Vitamin B12 Level**: Chronic deficiency can cause neurologic symptoms, but not a priority in acute seizure workup.   **Conclusion**: Immediate priorities are neuroimaging (non-contrast CT), metabolic labs, ECG, and toxicology to exclude life-threatening causes. EEG and further testing (e.g., MRI) may follow based on initial findings. |
| Diag2 | **Given the CT scan findings of an ill-defined rounded mass adjacent to the dura of the posterior falx, the most appropriate next steps to guide diagnosis and treatment are:**   1. **MRI with and without Contrast of the Head:**   **This is the gold standard for further characterizing the dural mass. It provides detailed information about the size, location, enhancement pattern, and relationship to surrounding structures, which is critical for diagnosing meningioma or other dural-based lesions.**   1. **EEG (Electroencephalography):**   **To evaluate for epileptiform activity and confirm whether the patient’s episode was a seizure. This is particularly important given the clinical presentation (confusion, tongue laceration, and urinary incontinence).**  **Why Not the Others?**   1. **Carotid Ultrasound:**   **Not indicated unless there is suspicion of carotid artery disease or stroke, which is not supported by the clinical or imaging findings.**   1. **EMG (Electromyography):**   **Used to evaluate peripheral nerve or muscle disorders, which are not relevant to this patient’s presentation.**   1. **Lumbar Puncture:**   **Not indicated unless there is suspicion of infection (e.g., meningitis) or subarachnoid hemorrhage, which are not supported by the clinical or imaging findings.**   1. **Nerve Conduction Studies:**   **Used to evaluate peripheral neuropathies, which are not relevant to this patient’s presentation.**  **Final Answer:**   - **MRI with and without Contrast of the Head** - **EEG**   **These steps will help confirm the diagnosis of the dural mass and evaluate for seizure activity, guiding further management.**  **2 / 2** | **Most Appropriate Next Steps:**  ✅ **MRI with and without contrast of the head** ✅ **EEG (Electroencephalography)**  **Rationale**:   1. **MRI with and without contrast of the head**:   **Critical** to characterize the **ill-defined rounded mass** adjacent to the posterior falx. MRI provides superior soft-tissue resolution compared to CT, helping differentiate between:  **Meningioma** (most likely, given dural attachment).**Metastasis** (if the patient has a cancer history).  **Chronic subdural hematoma** (if trauma is suspected).  **Abscess** or **inflammatory lesion** (less likely without systemic signs of infection).  Contrast enhances visualization of tumor vascularity, margins, and involvement of adjacent structures.   1. **EEG**:   Indicated if the patient has **seizures**, **altered mental status**, or unexplained neurologic symptoms (e.g., episodic confusion, staring spells).  The CT finding of a mass raises concern for **seizure activity** secondary to structural brain pathology, even if no overt seizures are reported.  **Excluded or Less Urgent Options:**   - **Carotid ultrasound**: Not relevant here. The posterior falx mass is intracranial and unrelated to carotid artery disease (typically evaluated for anterior circulation strokes or TIAs). - **EMG/Nerve conduction studies**: Focus on peripheral neuromuscular disorders (e.g., neuropathy, myopathy), which do not explain an intracranial mass. - **Lumbar puncture**:   **Risk of herniation** if the mass causes elevated intracranial pressure (CT shows no mass effect, but MRI should first rule this out).  May be considered later if infection (e.g., meningitis) or leptomeningeal carcinomatosis is suspected, but **not first-line** without supportive clinical signs (e.g., fever, meningismus).  **Next Steps**:   1. **Urgent MRI** to define the mass and guide biopsy/neurosurgical planning. 2. **EEG** if seizures are clinically suspected. 3. **Neurology/neurosurgery consultation** based on MRI findings.   **Final Answer**: **MRI with and without contrast of the head** and **EEG** are essential to evaluate the intracranial mass and assess for seizure activity. | **Most Appropriate Steps to Guide Diagnosis and Treatment:**   1. **MRI with and without Contrast of the Head**   **Critical** to characterize the "ill-defined rounded mass adjacent to the dura" seen on CT. MRI provides superior soft-tissue resolution to differentiate meningioma (e.g., dural tail sign, homogeneous enhancement) from metastases, abscess, or other lesions. It also evaluates for edema, compression, or subtle parenchymal abnormalities not visible on CT.   1. **EEG (Electroencephalography)**   Essential if seizures are suspected (e.g., episodic altered mental status, convulsions). The intracranial mass may act as an epileptogenic focus, and EEG helps confirm seizure activity, guide antiepileptic therapy, and assess the need for long-term management.  **Why Other Options Are Less Appropriate:**   - **Carotid Ultrasound**: Evaluates for carotid stenosis or vascular disease, which is unrelated to the dural mass. Not indicated here. - **EMG/Nerve Conduction Studies**: Assess peripheral nerve/muscle disorders (e.g., neuropathy, myopathy). The patient’s findings suggest central (brain) pathology, not peripheral. - **Lumbar Puncture**: High risk if there is significant mass effect (risk of herniation). Reserved for suspected infection (e.g., meningitis) or carcinomatous meningitis, which are not supported by current data (normal metabolic profile, negative toxicology).   **Next Steps After MRI/EEG:**   - **Neurosurgery Consultation**: If MRI confirms a meningioma or other resectable lesion. - **Hyponatremia Workup**: Check urine osmolality and sodium to evaluate for SIADH (common with intracranial pathology). - **Seizure Management**: Initiate antiepileptic drugs if EEG confirms epileptiform activity.   **Conclusion**: Prioritize **MRI brain with contrast** and **EEG** to address the critical findings (mass lesion and potential seizures). |
| Dx | **The most likely diagnosis in this patient is:**  **Seizure due to Meningioma**  **Rationale:**  **The MRI of the head confirms the presence of a meningioma, which is a known cause of seizures due to irritation or compression of the brain parenchyma.**   - **The patient’s clinical presentation (confusion, amnesia, tongue laceration, urinary incontinence, and bilateral arm pain) is consistent with a generalized tonic-clonic seizure.** - **The EEG being normal does not rule out a seizure, as interictal EEGs can be normal in patients with epilepsy or seizures due to structural brain lesions.**   **Why Not the Others?**   1. **Cryptogenic Seizures:**   **Cryptogenic seizures are seizures of unknown cause. In this case, the meningioma provides a clear structural cause for the seizure.**   1. **Delirium Tremens:**   **The patient has no history of heavy alcohol use or withdrawal symptoms (e.g., tremors, hallucinations), making this diagnosis unlikely.**   1. **Psychogenic Seizures:**   **Psychogenic nonepileptic seizures (PNES) are not associated with structural brain lesions like meningiomas. The presence of a meningioma makes this diagnosis unlikely.**   1. **Seizure due to Hypoglycemia:**   **The patient’s glucose level was normal (70 mg/dL), ruling out hypoglycemia as a cause.**   1. **Seizure due to Hyponatremia:**   **While the patient has mild hyponatremia (133 mmol/L), it is unlikely to be severe enough to cause a seizure. The meningioma is a more likely explanation.**  **Final Answer:**   - **Seizure due to Meningioma**   **This diagnosis explains the patient’s symptoms and imaging findings. Management should include neurology consultation and consideration of surgical resection or radiation therapy for the meningioma, depending on its size, location, and symptoms. Antiseizure medications (e.g., levetiracetam) may also be initiated to prevent further seizures.**  **14 / 14** | **Final Answer**: ✅ **Seizure due to meningioma**  **Rationale**:   - The **MRI-confirmed meningioma** provides a **structural cause** for seizures. Meningiomas are known to irritate adjacent cortical tissue, leading to focal or generalized seizures. - **Normal EEG** does not exclude seizures caused by structural lesions, as interictal EEG (between seizures) can be normal, especially if the lesion is small or deep. - Other options are less likely:   **Cryptogenic seizures**: Implies no identifiable cause, but here the meningioma is a clear structural etiology.  **Delirium tremens**: Requires a history of alcohol withdrawal (not mentioned).  **Psychogenic seizures**: Typically associated with normal imaging/EEG *during episodes* (not just interictally).  **Hypoglycemia/hyponatremia**: Require lab evidence of metabolic derangement (no clinical data provided).  **Conclusion**: The meningioma is the most likely etiology for the seizure in this patient. | **The most likely diagnosis in this patient is:** **• Seizure due to meningioma**  **Key Rationale:**   1. **Structural Cause Identified**: The MRI confirms a **meningioma**, a known cause of seizures due to direct irritation of brain tissue or mass effect. 2. **EEG Limitations**: A normal EEG does not exclude seizures caused by a meningioma. EEG can miss focal seizures, especially if the lesion is deep or the seizure did not occur during the recording. 3. **Hyponatremia (Na⁺ 133 mmol/L)**: Mild hyponatremia is unlikely to directly cause seizures (typically occurs at Na⁺ <120 mmol/L). However, it may reflect SIADH secondary to the meningioma, supporting the tumor’s role in the clinical picture. 4. **Exclusion of Other Options**:   **Cryptogenic seizures**: Requires *no identifiable cause* after evaluation, which is contradicted by the presence of a meningioma.  **Psychogenic seizures**: Less likely given the structural lesion; normal EEG alone is insufficient to diagnose psychogenic seizures.  **Hypoglycemia/Delirium tremens**: Glucose was normal (90 mg/dL), and there is no history of alcohol withdrawal.  **Conclusion:**  The meningioma is the most likely etiology for the seizure. Immediate neurosurgical evaluation and MRI with contrast are critical next steps. |
| Mang | **Given the diagnosis of seizure due to meningioma, the most appropriate next steps are:**   1. **Admit the Patient to the Hospital for Surgical Resection of the Meningioma:**   **Meningiomas causing seizures are often managed with surgical resection, especially if symptomatic or accessible. Admission allows for preoperative evaluation, seizure management, and planning for surgery.**   1. **Initiate Antiseizure Medication:**   **To prevent further seizures while awaiting surgery or further evaluation. The preferred options include:**  **Levetiracetam: First-line due to its efficacy, tolerability, and lack of drug interactions.**  **Phenytoin: An alternative, though it has more side effects and drug interactions.**  **Why Not the Others?**   1. **Discharge the Patient with a Prescription for Valproate 10 mg/kg po once/day:**   **Discharging the patient is inappropriate at this time, as meningiomas causing seizures typically require inpatient evaluation and surgical planning. Valproate is also less commonly used as a first-line antiseizure medication in this context.**   1. **Discharge the Patient with Follow-Up with a Neurologist if Symptoms Recur:**   **Discharging the patient without addressing the meningioma or initiating antiseizure medication is inappropriate, as the risk of recurrent seizures is high.**   1. **Carbamazepine 200 mg po bid:**   **Carbamazepine is not typically used as a first-line antiseizure medication for focal or generalized seizures due to its side effect profile and drug interactions.**   1. **Lorazepam 2 mg IV:**   **Lorazepam is used for acute seizure termination (e.g., status epilepticus) but is not appropriate for long-term seizure prevention.**   1. **Phenobarbital 2 mg/kg po once/day at bedtime:**   **Phenobarbital is rarely used as a first-line antiseizure medication due to its sedative effects and risk of dependence.**   1. **Phenytoin 4 mg/kg po once/day at bedtime:**   **While phenytoin can be used, it is not the first-line choice due to its side effects and drug interactions.**  **Final Answer:**   - **Admit the patient to the hospital for surgical resection of the meningioma** - **Initiate antiseizure medication (e.g., levetiracetam or phenytoin)**   **These steps address both the underlying cause (meningioma) and the acute symptom (seizure) while ensuring the patient’s safety and appropriate management.** | **Final Answer**: ✅ **Admit the patient to the hospital for surgical resection of the meningioma** ✅ **Carbamazepine 200 mg po bid**  **Rationale**:   1. **Admission for surgical resection**:   Symptomatic meningiomas (e.g., causing seizures) typically warrant **neurosurgical evaluation and resection**, especially if the tumor is accessible. Surgery addresses the underlying cause and reduces seizure recurrence risk.   1. **Carbamazepine**:   A first-line antiepileptic drug (AED) for **focal seizures**, which are commonly associated with meningiomas.  The dose of **200 mg bid** is appropriate for initiating therapy in adults (avoids the underdosing concern seen with the listed valproate dose).  Avoids enzyme-inducing AEDs like phenytoin, which may complicate perioperative care or interact with other medications.  **Excluded Options**:   - **Valproate 10 mg/kg/day**: Dose is subtherapeutic (typical maintenance: 20–30 mg/kg/day). - **Discharge with follow-up if symptoms recur**: Inadequate for a symptomatic meningioma requiring definitive treatment. - **Lorazepam IV**: Used for acute seizure termination, not maintenance therapy. - **Phenobarbital/Phenytoin**: Less preferred due to side effects, drug interactions, or outdated use.   **Conclusion**: Immediate surgical evaluation and seizure control with carbamazepine are the most appropriate next steps. | **The most appropriate next steps are:**   - **Admit the patient to the hospital for surgical resection of the meningioma** - **Carbamazepine 200 mg po bid** - **Phenytoin 4 mg/kg po once/day at bedtime**   **Rationale:**   1. **Admit for Surgical Resection**:   Definitive management of a seizure-causing meningioma requires **neurosurgical evaluation and resection**, especially if the tumor is accessible. Admission ensures timely intervention and perioperative care.   1. **Initiate Antiepileptic Drug (AED) Therapy**:   **Carbamazepine** and **phenytoin** are first-line AEDs for **focal seizures** (common with meningiomas). Both are appropriate for perioperative seizure prophylaxis in this context:  **Carbamazepine**: Effective for focal seizures but may worsen hyponatremia (Na⁺ 133 mmol/L). Use cautiously, monitoring electrolytes.  **Phenytoin**: Less likely to exacerbate hyponatremia and suitable for short-term perioperative use.  **Valproate** is a broad-spectrum AED but less preferred here due to its side effect profile (e.g., hepatotoxicity, weight gain) and lack of specificity for focal seizures.   1. **Avoid Discharge or Inappropriate Medications**:   **Discharge with follow-up** is unsafe due to the risk of recurrent seizures and tumor progression.  **Lorazepam IV** is for acute seizure termination, not maintenance.  **Phenobarbital** is rarely used due to sedation and dependency risks.  **Key Considerations:**   - **Hyponatremia**: Monitor Na⁺ closely, as carbamazepine can lower sodium further. - **Surgical Timing**: AEDs are a bridge to surgery; resection addresses the root cause. - **EEG Normalcy**: A normal EEG does not rule out future seizures; AEDs are still indicated.   **Final Answer:** Admit for surgery + start **carbamazepine** or **phenytoin**. |
| Case5 |  |  |  |
| Diff | **Based on the history and physical examination, the following differential diagnoses cannot be excluded at this stage:**   1. **Asthma:**   **Key findings (chronic cough, wheezing, environmental triggers) strongly suggest asthma, but spirometry is required for confirmation.**   1. **Gastroesophageal Reflux Disease (GERD):**   **Though she denies recent heartburn, "silent reflux" can present with chronic cough. GERD cannot be ruled out without further evaluation (e.g., pH monitoring or empiric PPI trial).**   1. **Medication-Induced Cough:**   **Losartan (an ARB) is associated with cough in some patients. Temporal overlap with smoke exposure complicates attribution, but medication etiology cannot be excluded without discontinuing losartan.**   1. **Postnasal Drip:**   **She reports nighttime nasal congestion, which could indicate allergic rhinitis or non-allergic rhinitis contributing to cough. Lack of visible postnasal drip on exam does not fully exclude this.**   1. **Postviral Cough:**   **A preceding subclinical upper respiratory infection (URI) could explain an 8-week cough, even if not recalled.**   1. **Lung Cancer:**   **Although low risk (nonsmoker, no family history), chronic cough warrants imaging (chest X-ray) to exclude malignancy, especially given her history of uterine cancer (irrelevant but highlights need for vigilance).**   1. **Pneumonia:**   **Atypical pneumonia (e.g., *Mycoplasma*) can present with dry cough and no fever. Chest X-ray is needed to exclude infiltrates.**  **Differentials That Can Likely Be Excluded:**   - **Acute Bronchitis: Duration >8 weeks makes this unlikely.** - **Coronary Ischemia: Pain is pleuritic/mechanical, not exertional; no cardiac risk factors or exam findings.** - **Fungal Infection: No travel to endemic areas, no systemic symptoms (fever, unintentional weight loss).** - **Sarcoidosis: No systemic signs (e.g., skin lesions, uveitis) or typical demographics (more common in African Americans).** - **Tuberculosis: No risk factors (no exposure, no night sweats), and weight loss is intentional.**   **Next Steps:**   - **Chest X-ray: Rule out structural causes (e.g., pneumonia, lung cancer).** - **Spirometry with Bronchodilator Response: Confirm asthma.** - **Trial of GERD Therapy: If asthma workup is negative.** - **Consider Switching Losartan: To exclude medication-induced cough.**   **These steps will narrow the differentials and guide definitive diagnosis.** | Based on the history and physical examination, the following differential diagnoses **cannot be definitively excluded** at this time:   1. **Gastroesophageal reflux disease (GERD):**   Though she denies recent heartburn, GERD can present as a chronic cough without typical reflux symptoms ("silent GERD").   1. **Postnasal drip (Upper Airway Cough Syndrome):**   She reports nocturnal congestion, which could suggest postnasal drip even if no overt rhinorrhea or postnasal drip was observed on exam.   1. **Lung cancer:**   Despite no smoking history, secondhand smoke exposure is a risk factor. Intentional weight loss (though explained) and chronic cough warrant imaging to exclude malignancy.   1. **Medication-induced cough:**   Losartan (ARB) is a rare cause of cough. While ACE inhibitors are more commonly implicated, ARBs are not entirely excluded without a trial of discontinuation.   1. **Sarcoidosis:**   Can present with cough and wheezing. Chest imaging (e.g., X-ray) is needed to assess for hilar lymphadenopathy or parenchymal changes.   1. **Fungal infection of the lungs:**   Less likely given no travel or environmental exposures, but imaging is required to exclude atypical infections.   1. **Tuberculosis (TB):**   Low probability (no fever, night sweats, or risk factors), but chronic cough necessitates exclusion with chest X-ray or sputum testing.  **Differentials that can likely be excluded:**   - **Acute bronchitis:** Duration (8 weeks) exceeds the typical 3-week course. - **Coronary ischemia:** Chest pain is non-exertional, sharp, and reproducible with movement/breathing, inconsistent with angina. - **Pneumonia:** No fever, productive cough, or auscultatory findings (e.g., crackles). - **Postviral cough:** Unlikely without a preceding URI history.   **Key next steps:**   - **Chest X-ray** to evaluate for structural lesions (e.g., cancer, sarcoidosis, infection). - **Spirometry ± bronchodilator challenge** to confirm asthma. - **GERD evaluation** (e.g., empiric trial of PPIs, pH monitoring). - **Consider temporary discontinuation of losartan** if cough persists despite asthma therapy.   **Final Answer:**   - **GERD** - **Postnasal drip** - **Lung cancer** - **Medication-induced cough** - **Sarcoidosis** - **Fungal infection of lungs** - **Tuberculosis (TB)** | **Differential Diagnoses That Cannot Be Excluded:**   1. **Asthma**   Supported by wheezing, cough triggered by smoke, and exertion. Needs spirometry to confirm.   1. **Gastroesophageal Reflux Disease (GERD)**   Chronic cough can occur without typical heartburn. History of intermittent GERD raises suspicion.   1. **Postnasal Drip**   Nighttime congestion reported, though no overt nasal discharge. Subtle postnasal drip may still contribute.   1. **Postviral Cough**   Possible if an undiagnosed URI preceded the cough, though 8-week duration is at the upper limit.   1. **Lung Cancer**   Chronic cough + weight loss (even intentional) warrants imaging to exclude malignancy, despite low risk (nonsmoker).   1. **Medication-Induced Cough**   Losartan (ARB) rarely causes cough, but drug reactions cannot be excluded without discontinuation.   1. **Coronary Ischemia**   Chest pain is atypical (right-sided, positional), but cardiac evaluation (e.g., EKG, troponin) is needed to rule out.   1. **Sarcoidosis**   Can present with cough, wheezing, and chest pain. Requires imaging (e.g., chest X-ray/CT) or biopsy to exclude.  **Excluded Diagnoses Based on History/Exam:**   - **Acute Bronchitis**: Duration >8 weeks and non-productive cough make this unlikely. - **Pneumonia**: Absence of fever, productive cough, crackles, or hypoxemia reduces likelihood. - **Tuberculosis (TB)**: No risk factors (travel, exposure, fever) or systemic symptoms; low suspicion. - **Fungal Infection**: No endemic exposure or immunocompromise; weight loss is intentional.   **Final Answer:**   - Asthma - GERD - Postnasal drip - Postviral cough - Lung cancer - Medication-induced cough - Coronary ischemia - Sarcoidosis |
| Diag | **The most appropriate next step is pulmonary function testing (PFT).**  **Rationale:**   1. **Suspected Asthma:**   **The patient’s chronic cough, wheezing, and environmental triggers (smoke exposure) strongly suggest asthma. PFT (specifically spirometry with bronchodilator response) is the gold standard to confirm reversible airflow obstruction, which is critical for diagnosing asthma.**   1. **Immediate Relevance to Symptoms:**   **While a chest x-ray is important to rule out structural lung disease (e.g., pneumonia, malignancy), it is less likely to directly confirm asthma. PFT directly addresses the leading diagnosis and guides immediate management.**   1. **Other Differentials:**   **GERD, postnasal drip, and medication-induced cough may require further testing (e.g., pH monitoring, sinus CT, or medication adjustment), but these are secondary considerations after evaluating for asthma.**   1. **Clinical Guidelines:**   **In suspected asthma, spirometry is recommended as the first-line diagnostic test by guidelines (e.g., GINA, NAEPP). Chest x-ray is typically reserved for atypical presentations or red flags (e.g., hemoptysis, weight loss), but her weight loss is intentional and explained.**  **Why Not Other Options?**   - **Chest X-ray: Important but secondary to PFT in this context.** - **Ambulatory pH Monitoring/CT Sinuses: Premature without first ruling out asthma.** - **ECG: Chest pain is pleuritic/musculoskeletal; no cardiac risk factors or symptoms.**   **Next Step: Proceed with pulmonary function testing to confirm asthma. If inconclusive or negative, pursue chest x-ray and other evaluations.**  **Answer: Pulmonary function testing** | **Answer:** **• Pulmonary function testing**  **Rationale:** The patient’s history (chronic cough, wheezing, nocturnal symptoms, and secondhand smoke exposure) and physical exam findings (bilateral wheezing on forced expiration) strongly suggest **ashma** as the leading diagnosis. Pulmonary function testing (PFT), including spirometry with bronchodilator challenge, is the **most appropriate next step** to objectively confirm reversible airflow obstruction, which is critical for diagnosing asthma. While a chest x-ray is also important to exclude structural abnormalities (e.g., lung cancer, infection), PFT directly addresses the suspected underlying pathophysiology and is prioritized in this context. Other options (e.g., pH monitoring, sinus CT, ECG) are less relevant given the absence of supporting symptoms (e.g., heartburn, sinusitis, or cardiac risk factors).  **Note:** In practice, both **chest x-ray and PFT** are typically performed early in the evaluation of chronic cough. However, if only one choice is allowed, PFT is prioritized to confirm the suspected diagnosis of asthma. | **Answer:** **Pulmonary function testing**  **Rationale:**   1. **Asthma is the leading diagnosis** based on chronic cough, wheezing, and smoke-triggered symptoms. Pulmonary function testing (spirometry ± bronchodilator challenge) is **critical to confirm reversible airflow obstruction**, which is diagnostic for asthma. 2. **Chest X-ray** is reasonable to exclude structural lung disease (e.g., malignancy, sarcoidosis), but:   Weight loss is intentional, and there are no red flags (e.g., hemoptysis, fever).  **PFTs take priority** in this context, as they directly evaluate the suspected pathophysiology.   1. **ECG** is lower yield given the **atypical chest pain** (right-sided, positional, non-exertional) and no cardiac risk factors or exam findings. 2. **Ambulatory pH monitoring** and **CT sinuses** are more appropriate if GERD or sinusitis remain high concerns **after ruling out asthma**.   **Final Answer:** **Pulmonary function testing** |
| Mang | **Appropriate Management Steps:**   1. **Budesonide inhaler 1 puff bid**   **Rationale: Inhaled corticosteroids (ICS) are the cornerstone of maintenance therapy for asthma. Budesonide reduces airway inflammation and prevents exacerbations.**   1. **Albuterol inhaler 2 puffs q 6 h and prn for wheezing**   **Rationale: Short-acting β-agonists (SABAs) like albuterol are essential for rescue therapy (prn use). While scheduled dosing (q 6 h) is not routinely recommended for chronic asthma, it may be temporarily used during symptom flare-ups. Ensure the patient is educated to prioritize prn use for symptom relief.**   1. **Stop losartan**   **Rationale: Although ARBs (like losartan) are less likely to cause cough than ACE inhibitors, they can still rarely trigger cough. Discontinuing losartan eliminates a potential contributor to persistent cough, especially if symptoms do not fully resolve with asthma therapy.**   1. **Ibuprofen 400 mg po q 6 h prn for chest pain**   **Rationale: The pleuritic chest pain is likely musculoskeletal from coughing. NSAIDs like ibuprofen are appropriate for pain relief in the absence of contraindications (e.g., GI bleeding).**   1. **Omeprazole 20 mg po once/day**   **Rationale: GERD can coexist with asthma and exacerbate cough. An empiric PPI trial is reasonable to address possible acid reflux, particularly if nighttime symptoms or heartburn are present.**  **Why Not Other Options?**   - **Combined ipratropium/albuterol inhaler: Primarily used for COPD or acute asthma exacerbations, not chronic asthma management.** - **Prednisone 40 mg po once/day for 5 days: Reserved for acute exacerbations (e.g., severe wheezing unresponsive to SABAs). Not indicated here unless symptoms are uncontrolled despite ICS and SABA.** - **Oxycodone/acetaminophen: Opioids are inappropriate for musculoskeletal pain due to risks of addiction and side effects.** - **Theophylline: Not first-line for asthma. Used only in refractory cases due to narrow therapeutic window and drug interactions.**   **Next Steps:**   - **Monitor response to ICS and albuterol.** - **Reassess cough and chest pain in 4-6 weeks.** - **If cough persists, consider chest x-ray or sinus CT.**   **Answer: Budesonide inhaler 1 puff bid, Albuterol inhaler 2 puffs q 6 h and prn, Stop losartan, Ibuprofen 400 mg po q 6 h prn, Omeprazole 20 mg po once/day.** | **Answer:** **• Albuterol inhaler 2 puffs q 6 h and prn for wheezing** **• Budesonide inhaler 1 puff bid** **• Stop losartan** **• Ibuprofen 400 mg po q 6 h prn for chest pain**  **Rationale:**   1. **Albuterol inhaler**: A short-acting beta-agonist (SABA) is essential for **rescue therapy** to relieve acute wheezing and bronchospasm, consistent with asthma management guidelines. 2. **Budesonide inhaler**: Inhaled corticosteroids (ICS) are **first-line maintenance therapy** for asthma to suppress airway inflammation and prevent symptoms. This is critical for long-term control. 3. **Stop losartan**: Although ARBs (like losartan) rarely cause cough compared to ACE inhibitors, eliminating this potential contributor is prudent, especially if the cough persists despite asthma treatment. 4. **Ibuprofen prn for chest pain**: If chest pain is due to musculoskeletal strain from chronic coughing, NSAIDs like ibuprofen are appropriate for symptomatic relief. Avoid opioids (e.g., oxycodone) due to lack of efficacy and risks.   **Excluded Options:**   - **Combined ipratropium/albuterol inhaler**: Primarily used for **COPD or acute asthma exacerbations**, not routine asthma maintenance. - **Omeprazole**: Lacks justification without symptoms or risk factors for GERD (e.g., heartburn). Reserve for refractory cough if asthma therapy fails. - **Prednisone**: Oral steroids are reserved for **acute exacerbations** (e.g., severe symptoms unresponsive to SABA). Not indicated here for stable chronic management. - **Theophylline**: Not first-line due to narrow therapeutic window and side effects. Use only if ICS + SABA fail. - **Oxycodone/acetaminophen**: Avoid opioids for cough or musculoskeletal pain due to risks of dependence and minimal benefit.   **Clinical Approach:**   - Confirm asthma diagnosis with PFT and initiate **ICS + SABA**. - Address potential medication-induced cough by discontinuing losartan. - Manage secondary symptoms (e.g., chest pain) conservatively with NSAIDs. - Reassess in 4-6 weeks; if cough persists, consider GERD evaluation or sinus imaging. | **Answer:** **• Albuterol inhaler 2 puffs q 6 h and prn for wheezing** **• Budesonide inhaler 1 puff bid** **• Stop losartan** **• Omeprazole 20 mg po once/day**  **Rationale:**   1. **Albuterol + Budesonide**:   **Albuterol** (SABA) is indicated for acute symptom relief (wheezing).  **Budesonide** (inhaled corticosteroid) is a cornerstone of asthma maintenance therapy to reduce airway inflammation and prevent exacerbations.   1. **Stop Losartan**:   Although ACE inhibitors (not ARBs like losartan) are classically linked to cough, ARBs can rarely cause similar side effects. Discontinuing losartan helps rule out medication-induced cough.   1. **Omeprazole**:   GERD is a common comorbidity in asthma and can exacerbate cough. Empiric PPI therapy is reasonable to address potential acid reflux contributing to symptoms.   1. **Avoid NSAIDs/Opiates**:   **Ibuprofen** may worsen asthma in susceptible individuals (aspirin-exacerbated respiratory disease).  **Oxycodone/acetaminophen** is unnecessary for atypical chest pain (likely musculoskeletal or airway-related) and poses addiction risks.   1. **Prednisone/Theophylline**:   **Prednisone** is reserved for acute exacerbations with significant airflow limitation (not clearly indicated here).  **Theophylline** is a third-line agent due to its narrow therapeutic window and side effects.   1. **Combined Ipratropium/Albuterol**:   More appropriate for COPD or severe asthma exacerbations, not routine asthma management.  **Final Answer:** **Albuterol, Budesonide, Stop losartan, Omeprazole** |
| Case6 |  |  |  |
| Diff | **Common and/or Dangerous Differential Diagnoses That Cannot Be Excluded:**   1. **Foreign Body Aspiration**   **Rationale: Sudden onset of cough after dinner, transmitted wheezing (left lung), and episodic improvement followed by worsening raise suspicion. Foreign body aspiration is a critical diagnosis requiring urgent evaluation (e.g., chest X-ray, bronchoscopy).**   1. **Asthma Exacerbation**   **Rationale: History of recurrent wheezing, family history of asthma, and current wheezing make this a common and likely diagnosis. However, it remains a differential until confirmed by response to bronchodilators.**   1. **Pneumonia**   **Rationale: Although afebrile and lacking crackles, pneumonia can present atypically in young children. A chest X-ray is needed to exclude focal consolidation, especially with persistent respiratory distress.**   1. **Allergic Reaction (Anaphylaxis)**   **Rationale: Onset after dinner could suggest food allergy. Isolated respiratory symptoms (wheezing, cough) without rash or hypotension are atypical but possible. Anaphylaxis cannot be fully excluded without allergen exposure history.**  **Why Not Other Options?**   - **Bronchiolitis: Unlikely due to absence of fever, rhinorrhea, or typical viral prodrome.** - **Epiglottitis: Excluded by lack of fever, stridor, drooling, and toxic appearance. Vaccination against *Haemophilus influenzae* further reduces risk.** - **Pertussis: Incubation period and progression (weeks) mismatch the 1-day history. Vaccination status and absence of paroxysmal coughing/"whoop" make this unlikely.**   **Answer: Foreign body aspiration, Asthma exacerbation, Pneumonia, and Allergic reaction.** | **Answer:** **• Foreign body aspiration** **• Asthma exacerbation** **• Bronchiolitis** **• Pneumonia**  **Rationale:**   1. **Foreign body aspiration**: Sudden onset after dinner, unilateral wheezing (left lung), and absence of fever or preceding illness raise suspicion. **Cannot be excluded without imaging (e.g., chest X-ray)**. 2. **Asthma exacerbation**: History of recurrent wheezing, family history of asthma, and response to bronchodilators strongly support this diagnosis. However, other conditions (e.g., aspiration) must still be ruled out. 3. **Bronchiolitis**: Common in toddlers, though typically associated with viral prodrome (e.g., rhinorrhea, fever). Absence of these symptoms makes it less likely, but **cannot be definitively excluded without viral testing**. 4. **Pneumonia**: May present without fever or crackles (especially atypical pneumonia). Requires **chest X-ray** to confirm or exclude.   **Excluded Options:**   - **Allergic reaction**: No urticaria, angioedema, or exposure to allergens. Anaphylaxis is unlikely without systemic signs. - **Epiglottitis**: Rare due to Hib vaccination. Absence of drooling, stridor, or toxic appearance makes this highly unlikely. - **Pertussis**: Acute 1-day cough is inconsistent with pertussis, which features paroxysmal coughing over weeks. Vaccination status further reduces likelihood.   **Key Considerations:**   - **Foreign body aspiration** is a **critical emergency** requiring prompt imaging or bronchoscopy. - **Asthma exacerbation** and **bronchiolitis** are common but require differentiation based on clinical context and testing. - **Pneumonia** must be ruled out with imaging, even in the absence of classic findings (e.g., fever, crackles).   **Next Steps:**   - **Chest X-ray** to assess for foreign body, pneumonia, or hyperinflation (asthma/bronchiolitis). - Consider **viral testing** (e.g., RSV, rhinovirus) if bronchiolitis is suspected. - **Close monitoring** for response to bronchodilators and steroids. | **Answer:** **• Asthma exacerbation** **• Bronchiolitis** **• Foreign body aspiration** **• Pneumonia**  **Rationale:**   1. **Asthma Exacerbation**:   **Common**: The child has a history of recurrent wheezing and a family history of asthma. Current wheezing and respiratory distress strongly support this diagnosis.   1. **Bronchiolitis**:   **Common**: Although more typical in infants <12 months, it can occur up to age 2. The absence of fever or rhinorrhea does not exclude viral bronchiolitis (e.g., RSV), which can present with wheezing and respiratory distress.   1. **Foreign Body Aspiration**:   **Dangerous**: Sudden onset after dinner raises suspicion. Unilateral wheezing (left lung) and transmitted sounds suggest possible airway obstruction. A retained foreign body requires urgent evaluation (e.g., chest X-ray, bronchoscopy).   1. **Pneumonia**:   **Dangerous**: Can present with wheezing (e.g., from atelectasis) even without fever or crackles. A chest X-ray is needed to rule out infiltrates.   1. **Excluded Diagnoses**:   **Allergic Reaction**: No urticaria, angioedema, or exposure history.  **Epiglottitis**: Vaccinated against *H. influenzae*, afebrile, and no stridor/drooling.  **Pertussis**: No paroxysmal coughing spells, whoop, or post-tussive vomiting. Vaccination makes this less likely.  **Final Answer:** **Asthma exacerbation, Bronchiolitis, Foreign body aspiration, Pneumonia** |
| Diag1 | **Most Appropriate Next Steps:**   1. **Albuterol Nebulizer**   **Rationale: Immediate bronchodilation is needed for wheezing, whether due to asthma or reactive airways. Albuterol is first-line therapy and may improve symptoms while aiding diagnostic clarity (e.g., response supports asthma).**   1. **Chest X-ray (AP, Lateral, and Expiratory Views)**   **Rationale: Critical to evaluate for foreign body aspiration (e.g., air trapping on expiratory films) and exclude pneumonia. The sudden onset after dinner and transmitted wheezing heighten suspicion for aspiration.**   1. **Pulse Oximetry**   **Rationale: Essential to assess oxygenation and guide urgency of intervention. Hypoxia (SpO₂ <92%) would warrant supplemental oxygen and closer monitoring.**  **Why Not Other Options?**   - **Basic Metabolic Panel (BMP): No evidence of dehydration, electrolyte imbalance, or metabolic derangement. Unnecessary in this acute respiratory presentation.** - **CT Scan of Chest: Too high-risk (radiation exposure, potential sedation) without clear indication. Reserved for cases where X-ray is inconclusive or suspicion remains high.** - **CBC with Differential: Unlikely to change acute management. Afebrile with no signs of bacterial infection (e.g., pneumonia would require X-ray, not CBC, for diagnosis).** - **Discharge Home on Cool-Mist Vaporizer: Child has respiratory distress; discharging before ruling out serious causes (e.g., foreign body) is unsafe. Cool mist lacks evidence for efficacy.** - **Heimlich Maneuver: Not indicated. The child is stable, speaking, and the event occurred 24 hours prior. Heimlich is for acute choking with complete airway obstruction.**   **Answer: Albuterol nebulizer, Chest X-ray (AP, lateral, expiratory views), and Pulse oximetry.** | **Answer:** **• Albuterol nebulizer** **• Chest x-ray: Anteroposterior (AP), lateral, and expiratory views** **• Pulse oximetry**  **Rationale:**   1. **Albuterol nebulizer**: Immediate bronchodilator therapy is critical for **relieving wheezing and bronchospasm**, especially given the child’s history of prior wheezing episodes responsive to bronchodilators. Nebulized albuterol is preferred in young children for effective delivery. 2. **Chest X-ray (AP, lateral, expiratory views)**: Essential to evaluate for **foreign body aspiration** (e.g., air trapping on expiratory films) and **pneumonia**. The sudden onset after eating, unilateral wheezing, and lack of fever heighten suspicion for aspiration. 3. **Pulse oximetry**: Necessary to assess oxygenation and guide management, even if the child is not cyanotic. Subtle hypoxia may influence treatment decisions.   **Excluded Options:**   - **Basic metabolic panel (BMP)**: No evidence of dehydration, electrolyte imbalance, or renal dysfunction. Unnecessary in this scenario. - **CT scan of chest**: Not first-line due to radiation exposure. Reserved if chest X-ray is inconclusive and clinical suspicion remains high. - **CBC with differential**: Low utility in acute setting without fever or signs of bacterial infection (e.g., leukocytosis). May be considered if pneumonia is confirmed. - **Discharge home on cool-mist vaporizer**: Discharge is unsafe before ruling out serious causes (e.g., foreign body). Cool-mist vaporizers are ineffective and not recommended. - **Heimlich maneuver**: Not indicated for a non-acute choking event. The child is stable and able to speak, with no active airway obstruction.   **Next Steps:**   - Administer **nebulized albuterol** and monitor response. - Perform **chest X-ray** to evaluate for foreign body or infiltrates. - Use **pulse oximetry** to guide oxygen therapy if needed. - If foreign body is confirmed, proceed to **bronchoscopy**. If pneumonia is detected, start antibiotics.   **Clinical Bottom Line:** Prioritize stabilizing the airway (albuterol), ruling out aspiration/pneumonia (X-ray), and assessing oxygenation (pulse oximetry). Avoid unnecessary tests (BMP, CT) and interventions (Heimlich, cool mist). | **Answer:** **• Albuterol nebulizer** **• Chest x-ray: Anteroposterior(AP), lateral, and expiratory views** **• Pulse oximetry**  **Rationale:**   1. **Albuterol nebulizer**:   **Immediate bronchodilation** is critical for wheezing and respiratory distress, especially given the child’s history of recurrent wheezing. Nebulized albuterol is preferred in young children who may not coordinate inhaler use effectively.   1. **Chest X-ray (AP, lateral, expiratory views)**:   Essential to evaluate for **foreign body aspiration** (sudden onset after dinner, unilateral wheezing) and **pneumonia**. Expiratory views can detect air trapping from a foreign body.  Also helps rule out other causes of respiratory distress (e.g., pneumothorax, mediastinal mass).   1. **Pulse oximetry**:   Assess oxygenation status. Even without cyanosis, hypoxia may occur in severe asthma, pneumonia, or foreign body aspiration.   1. **Excluded Options**:   **Basic metabolic panel (BMP)/CBC**: No signs of dehydration or systemic infection (afebrile, normal exam). Less urgent in this acute setting.  **CT scan**: Radiation risk and lack of immediate need; chest X-ray is first-line.  **Discharge home**: Child has respiratory distress; requires stabilization and diagnostic evaluation first.  **Heimlich maneuver**: Not indicated for lower airway foreign bodies. The child is speaking and breathing, ruling out complete airway obstruction.  **Final Answer:** **Albuterol nebulizer, Chest x-ray, Pulse oximetry** |
| Diag2 | **Most Appropriate Next Steps:**   1. **Take a Chest X-ray in the Left and Right Lateral Decubitus Positions**   **Rationale:**  **Decubitus views (lying on each side) can reveal hyperinflation of the dependent lung if a foreign body is obstructing a bronchus. This is a safer, lower-radiation alternative to CT and compensates for the inability to obtain expiratory views.**  **Foreign body aspiration remains a critical concern given the history (sudden onset after eating, transmitted wheezing).**   1. **Repeat Attempt at Chest X-ray Expiratory View**   **Rationale:**  **Expiratory films may still be attempted with coaching (e.g., blowing out a candle, using a toy whistle) to detect air trapping, a hallmark of foreign body aspiration.**  **If successful, this could avoid the need for more invasive testing (e.g., bronchoscopy).**   1. **Admit the Child for Observation**   **Rationale:**  **Foreign body aspiration cannot yet be excluded, and clinical deterioration (e.g., worsening respiratory distress, hypoxia) could occur.**  **Admission allows for serial monitoring, repeat imaging if needed, and expedited bronchoscopy if suspicion persists.**  **Why Not Other Options?**   - **Discharge on Amoxicillin:**   **No evidence of bacterial infection (normal chest X-ray, afebrile, no focal consolidation). Antibiotics are unnecessary.**   - **Discharge on Albuterol and Prednisone:**   **Premature if foreign body aspiration is not ruled out. Bronchodilators/steroids may temporarily mask symptoms of aspiration, delaying diagnosis.**  **Consider this only after excluding foreign body and confirming asthma/reactive airway disease.**  **Summary:**   - **Priority: Confirm or exclude foreign body aspiration using decubitus X-rays or repeat expiratory views.** - **Admission ensures safety while pursuing definitive diagnosis.** - **Avoid discharge until aspiration is confidently ruled out or the child is stable with a clear diagnosis.**   **Answer:**   - **Take a chest X-ray in the left and right lateral decubitus positions** - **Repeat attempt at chest X-ray expiratory view** - **Admit the child for observation** | **Answer:** **• Take a chest x-ray in the left and right lateral decubitus positions** **• Admit the child for observation**  **Rationale:**   1. **Lateral decubitus chest X-rays**:   Critical to evaluate for **foreign body aspiration**. In young children who cannot cooperate with expiratory views, decubitus films can reveal **air trapping** (e.g., hyperlucency on the affected side when placed in the dependent position). This is a safer and more practical alternative to expiratory views.  A repeat attempt at expiratory views is unlikely to succeed if the child cannot follow instructions.   1. **Admit for observation**:   Persistent wheezing and unresolved suspicion for foreign body necessitate **close monitoring**. Admission allows:  Serial respiratory assessments.  Further imaging (e.g., fluoroscopy) or consultation with pediatric pulmonology/ENT for bronchoscopy if decubitus X-rays are inconclusive.  Avoids discharging a child with a potential retained foreign body, which could lead to complications like pneumonia or respiratory distress.  **Excluded Options:**   - **Discharge on amoxicillin**: No evidence of bacterial infection (normal chest X-ray, no fever). Antibiotics are unnecessary. - **Discharge on albuterol and prednisone**: While albuterol may temporarily relieve symptoms, prednisone is premature without ruling out foreign body. Steroids could mask inflammation from an aspirated object, delaying diagnosis. - **Repeat expiratory view**: Likely futile if the child cannot cooperate. Decubitus films are more reliable.   **Clinical Bottom Line:**   - **Foreign body aspiration remains the top concern** despite initial normal X-rays. Prioritize lateral decubitus films to assess for air trapping. - Admission ensures safety while pursuing definitive diagnosis (e.g., bronchoscopy if imaging is equivocal). - Avoid discharge until aspiration is confidently excluded. | **Answer:** **• Take a chest x-ray in the left and right lateral decubitus positions** **• Admit the child for observation**  **Rationale:**   1. **Chest X-ray in lateral decubitus positions**:   The child’s inability to cooperate with expiratory views limits the utility of standard imaging. **Lateral decubitus views** (lying on each side) can detect **air trapping** from a foreign body. If the affected lung is non-dependent (e.g., left lateral decubitus position for right lung pathology), hyperinflation may become apparent due to air trapping. This is a safer and more practical alternative to repeating expiratory views or proceeding directly to CT.   1. **Admit for observation**:   The sudden onset of symptoms after eating, unilateral wheezing, and unresolved suspicion for **foreign body aspiration** warrant close monitoring. Admission allows for:  Serial clinical assessments (e.g., worsening distress, persistent cough).  Further imaging (e.g., CT or bronchoscopy) if lateral decubitus X-rays are inconclusive.  Stabilization if symptoms progress (e.g., increased work of breathing).   1. **Excluded Options**:   **Discharge on amoxicillin**: No evidence of bacterial infection (afebrile, normal chest X-ray).  **Discharge on albuterol/prednisone**: While recurrent wheezing suggests asthma, **foreign body aspiration must be ruled out first**, as bronchodilators/steroids may delay diagnosis.  **Repeat expiratory X-ray**: Unlikely to succeed if the child remains uncooperative.  **Final Answer:** **Take a chest x-ray in the left and right lateral decubitus positions, Admit the child for observation** |
| Dx | **Most Likely Diagnosis:**   - **Foreign Body Aspiration (Left or Right Lung)**   **Key Supporting Evidence:**   1. **Inconclusive Initial Imaging:**   **Normal AP/lateral chest X-ray does not rule out foreign body aspiration, as air trapping (a hallmark sign) requires expiratory views or decubitus imaging to detect. The child’s inability to cooperate with expiratory imaging raises suspicion.**   1. **Clinical Context:**   **Foreign body aspiration is a common pediatric emergency, often presenting with sudden-onset wheezing/coughing after choking (even if unwitnessed).**  **Asthma with air trapping is less likely in the absence of:**  **Prior history of asthma/reactive airway disease.**  **Improvement with albuterol (not mentioned here).**   1. **Exclusion of Alternatives:**   **Pneumonia: Unlikely with normal X-ray and lack of fever/consolidation.**  **Tension Pneumothorax: Incompatible with stable oxygenation (SpO₂ 96%) and absence of severe distress, tracheal deviation, or hypotension.**  **Why Not Asthma?**   - **Air trapping in asthma typically occurs in both lungs (diffuse hyperinflation), whereas foreign body aspiration causes unilateral hyperinflation (localized to the obstructed bronchus).** - **Asthma would also likely respond to albuterol, which is not described here.**   **Final Answer:**   - **Foreign Body Aspiration (Left or Right Lung).**   **Lateral decubitus X-rays or repeat expiratory views are critical to confirm the diagnosis.**  **Localization (left vs. right) depends on imaging findings (e.g., hyperinflation on decubitus views) or focal wheezing on exam.** | **Answer:** **• Foreign body, right lung**  **Rationale:**   1. **Foreign body aspiration** is the **most likely diagnosis** given the clinical context:   A normal AP/lateral chest X-ray **does not exclude** a foreign body, particularly if it is **radiolucent** (e.g., food).  The **right main bronchus** is anatomically more vertical and wider, making it the **most common site** for aspirated foreign bodies.  The inability to obtain an expiratory view (or decubitus films) leaves **air trapping** undetected, a hallmark of foreign body obstruction.   1. **Asthma with air trapping** is less likely unless there is a **clear history of asthma** or **response to bronchodilators**. Persistent wheezing after albuterol raises suspicion for an alternative etiology (e.g., foreign body). 2. **Pneumonia** is unlikely with a **normal chest X-ray** and absence of fever or consolidation. 3. **Tension pneumothorax** is ruled out by the **stable oxygen saturation (96%)** and lack of acute respiratory distress or asymmetrical breath sounds.   **Clinical Bottom Line:**   - While definitive localization requires imaging (e.g., decubitus X-rays, fluoroscopy) or bronchoscopy, the **right lung is the most probable site** for foreign body aspiration due to anatomical predisposition. - Urgent evaluation (e.g., decubitus films, bronchoscopy) is required to confirm and retrieve the foreign body. | **Answer:** **• Foreign body, right lung**  **Rationale:**   1. **Foreign Body Aspiration (Right Lung)**:   The history of sudden onset after eating, unilateral wheezing, and inability to cooperate with expiratory X-rays strongly suggests **foreign body aspiration**.  The **right main bronchus** is anatomically more vertical and wider, making it the **most common site** for aspirated foreign bodies in children.  While lateral decubitus X-rays or CT may confirm air trapping or localize the foreign body, the clinical presentation alone makes this the leading diagnosis.   1. **Excluded Diagnoses**:   **Asthma with air trapping**: Typically causes bilateral wheezing and hyperinflation, not unilateral findings.  **Pneumonia**: Unlikely without fever, focal infiltrates on X-ray, or systemic signs of infection.  **Tension pneumothorax**: Presents with acute respiratory distress, tracheal deviation, and absent breath sounds on the affected side, none of which are described here.  **Final Answer:** **Foreign body, right lung** |
| Mang | **Most Appropriate Treatment Orders:**   1. **Consult ENT or Pulmonary Specialist for Bronchoscopy**   **Rationale:**  **Bronchoscopy is the definitive diagnostic and therapeutic procedure for suspected foreign body aspiration.**  **mmediate referral is critical to prevent complications (e.g., post-obstructive pneumonia, respiratory failure).**  **Why Not Other Options?**   - **Sputum Gram Stain and Culture:**   **Not indicated. No evidence of bacterial infection (afebrile, normal chest X-ray).**   - **Amoxicillin/Clavulanate:**   **Antibiotics are unnecessary without signs of infection (e.g., fever, consolidation, leukocytosis).**   - **Back Blows/Inverted Position:**   **Contraindicated in a stable child with suspected lodged foreign body. These maneuvers are for acute choking emergencies (e.g., complete airway obstruction with cyanosis/apnea).**  **Risk of dislodging the object further into the airway.**   - **Suction Airway with Flexible Catheter:**   **Unlikely to remove a lodged foreign body and risks mucosal injury.**  **Answer:**   - **Consult ENT or Pulmonary Specialist for Bronchoscopy** | **Answer:** **• Consult ENT or pulmonary specialist for bronchoscopy**  **Rationale:**   1. **Consult ENT/pulmonary specialist for bronchoscopy**:   **Definitive management** for suspected foreign body aspiration. Bronchoscopy allows direct visualization and removal of the object.  Urgent consultation is warranted even if imaging (e.g., decubitus X-rays) is equivocal, as false negatives occur with radiolucent objects (e.g., food).  **Excluded Options:**   - **Sputum Gram stain and culture**: No evidence of bacterial infection (normal X-ray, no fever). Unnecessary in this context. - **Amoxicillin/clavulanate**: Antibiotics are not indicated without signs of pneumonia or purulent secretions. - **Back blows**: Used for **acute choking emergencies** with complete airway obstruction. Ineffective and inappropriate for a stable child with a lodged foreign body. - **Suction airway**: Flexible catheters cannot retrieve solid foreign bodies and may push the object deeper.   **Clinical Bottom Line:**   - **Bronchoscopy is the gold standard** for diagnosis and treatment of foreign body aspiration. - Avoid interventions that delay definitive management (e.g., antibiotics, suctioning) or are ineffective (e.g., back blows). | **Answer:** **• Consult ENT or pulmonary specialist for bronchoscopy**  **Rationale:**   1. **Consult ENT/Pulmonary for Bronchoscopy**:   **Definitive treatment** for foreign body aspiration is **rigid bronchoscopy** to remove the object. This is urgent if the child has respiratory distress or high suspicion of aspiration (e.g., unilateral wheezing, witnessed choking).  Delaying bronchoscopy risks complications like pneumonia, atelectasis, or airway edema.   1. **Excluded Options**:   **Sputum Gram stain/culture**: Not indicated unless there is evidence of infection (e.g., fever, purulent sputum, consolidations on X-ray).  **Amoxicillin/clavulanate IV**: No signs of bacterial pneumonia or aspiration pneumonia (child is afebrile, no infiltrates on X-ray).  **Back blows**: Used for acute choking emergencies with **complete airway obstruction** (e.g., inability to cough, breathe, or speak). This child is stable with partial obstruction; back blows are ineffective and potentially harmful once the object is lodged in the bronchus.  **Suction airway with flexible catheter**: Unlikely to retrieve a bronchial foreign body and may push it deeper.  **Final Answer:** **Consult ENT or pulmonary specialist for bronchoscopy** |
| Case7 |  |  |  |
| Diff | **Differential Diagnoses That Cannot Be Excluded:**   1. **Pneumonia**   **Rationale:**  **Productive cough, fever, chills, pleuritic chest pain, crackles on exam, and systemic symptoms (malaise, tachypnea) strongly suggest bacterial or viral pneumonia. Chest X-ray is needed for confirmation.**   1. **Pulmonary Embolism (PE)**   **Rationale:**  **Sharp pleuritic chest pain, dyspnea on exertion, and cough align with PE. While no obvious risk factors (e.g., DVT, immobility), PE can occur unexpectedly. Imaging (e.g., CT pulmonary angiogram) is required to rule out.**   1. **Acute Bronchitis**   **Rationale:**  **Viral or bacterial bronchitis can cause productive cough, fever, and chest discomfort. However, focal crackles and systemic symptoms lean toward pneumonia.**   1. **New-Onset Heart Failure**   **Rationale:**  **Hypertension is a risk factor. Dyspnea on exertion and crackles could indicate pulmonary edema, though absence of orthopnea, PND, or edema makes this less likely.**   1. **COPD Exacerbation**   **Rationale:**  **Former smoking history raises concern for undiagnosed COPD. Acute exacerbations can mimic pneumonia, but lack of prior COPD diagnosis or wheezing reduces likelihood.**   1. **Tuberculosis (TB)**   **Rationale:**  **Though acute symptoms are atypical for TB, his smoking history and constitutional symptoms (fever, fatigue) warrant exclusion, especially if risk factors (e.g., exposure) exist.**  **Excluded Diagnoses:**   - **ACE Inhibitor-Induced Cough:**   **Typically causes dry cough without fever or purulent sputum. No confirmation he is on ACE inhibitors.**   - **Acute Asthma:**   **Absence of wheezing or prior asthma history makes this unlikely.**   - **Lung Cancer:**   **Acute symptoms and lack of chronic findings (weight loss, hemoptysis) argue against cancer as the primary issue.**   - **Middle East Respiratory Syndrome (MERS):**   **No travel history or exposure to endemic regions.**   - **Postnasal Drip:**   **Initial URI symptoms (rhinorrhea, sore throat) may contribute, but progression to systemic illness suggests a more serious process.**  **Final Answer:**   - **Pneumonia** - **Pulmonary Embolism (PE)** - **Acute Bronchitis** - **COPD Exacerbation** - **New-Onset Heart Failure** - **Tuberculosis (TB)**   **Next Steps:**   - **Chest X-ray to evaluate for consolidation (pneumonia) or infiltrates.** - **D-dimer +/- CT Pulmonary Angiogram if PE is suspected.** - **BNP/NT-proBNP and echocardiogram if heart failure is considered.** - **Sputum AFB testing if TB is a concern.** | **Answer:** The following differential diagnoses **cannot be excluded** based on the current history and physical examination:   - **ACE inhibitor-induced cough** - **Acute bronchitis** - **Lung cancer** - **Pneumonia** - **Pulmonary embolism (PE)** - **TB**   **Rationale:**   1. **ACE inhibitor-induced cough**:   The patient takes enalapril, which can cause a chronic dry cough. While his productive cough and fever suggest infection, ACE inhibitor cough cannot be definitively excluded without discontinuing the medication.   1. **Acute bronchitis**:   Viral or bacterial bronchitis may present with cough, sputum, and URI symptoms. Systemic features (fever, chills) and focal crackles favor pneumonia, but CXR is required to confirm.   1. **Lung cancer**:   A former smoker (20 pack-years) is at risk. Lung cancer can mimic pneumonia (e.g., post-obstructive infection) or present acutely. Imaging/biopsy is needed to exclude.   1. **Pneumonia**:   Most likely diagnosis (fever, chills, productive cough, pleuritic pain, crackles), but requires chest X-ray confirmation.   1. **Pulmonary embolism (PE)**:   Pleuritic chest pain and dyspnea are classic for PE. Absence of traditional risk factors (e.g., DVT, immobility) does not exclude it; imaging (CTPA/VQ scan) is required.   1. **TB**:   Although subacute onset and weight loss are typical, TB can present acutely. Sputum AFB testing or chest X-ray is needed for exclusion.  **Excluded Diagnoses:**   - **Acute asthma**: No wheezing or prior history. - **COPD exacerbation**: No chronic respiratory symptoms or diagnosis. - **Middle East respiratory syndrome (MERS)**: No relevant travel/exposure. - **New-onset heart failure**: Absence of orthopnea, edema, or JVD. - **Postnasal drip**: URI symptoms exist, but systemic features (fever, chills) and productive cough suggest a more severe process.   **Clinical Bottom Line:** While **pneumonia** is the leading diagnosis, several critical alternatives (e.g., PE, TB, lung cancer) require further evaluation (CXR, sputum studies, imaging). ACE inhibitor cough and acute bronchitis remain possible until disproven. | **Answer:** **• Pulmonary embolism (PE)** **• Lung cancer** **• New-onset heart failure** **• Postnasal drip** **• TB**  **Rationale:**   1. **Pulmonary Embolism (PE)**:   Pleuritic chest pain, dyspnea, and fever overlap with pneumonia. PE cannot be excluded without further testing (e.g., D-dimer, CT angiography), especially if risk factors (e.g., immobility, hypercoagulability) are present.   1. **Lung Cancer**:   New cough with consolidation on CXR raises concern for post-obstructive pneumonia secondary to an underlying malignancy. Requires imaging (e.g., CT scan) or biopsy to exclude.   1. **New-Onset Heart Failure**:   Dyspnea on exertion and crackles could indicate fluid overload. Heart failure may coexist with or mimic pneumonia. BNP and echocardiography are needed for confirmation.   1. **Postnasal Drip**:   Concurrent upper respiratory symptoms (runny nose, sore throat) suggest postnasal drip as a contributing factor to cough, though it does not explain consolidation or systemic symptoms.   1. **TB**:   Acute presentations of tuberculosis can mimic bacterial pneumonia. Requires sputum AFB smears/cultures to exclude, particularly if risk factors (e.g., exposure, immunosuppression) exist.  **Excluded Diagnoses:**   - **Acute Bronchitis**: Excluded by CXR consolidation, which is absent in bronchitis. - **Middle East Respiratory Syndrome (MERS)**: No travel/endemic exposure mentioned. - **ACE Inhibitor-Induced Cough**: Typically dry and nonproductive; inconsistent with fever, purulent sputum, and consolidation. - **Acute Asthma**: No wheezing or prior history; consolidation favors pneumonia. - **COPD Exacerbation**: No history of COPD or chronic respiratory symptoms (e.g., chronic cough, sputum).   **Final Answer**: **Pulmonary embolism (PE)** **Lung cancer** **New-onset heart failure** **Postnasal drip** **TB** |
| Diag | **Most Appropriate Steps for Diagnosis and Treatment:**   1. **Chest X-ray**   **Rationale: Essential to confirm pneumonia (e.g., consolidation) and differentiate from other causes (e.g., heart failure, lung mass).**   1. **Blood and Sputum Cultures**   **Rationale: Systemic symptoms (fever, chills, purulent sputum) suggest bacterial infection. Cultures identify pathogens (e.g., *S. pneumoniae*) and guide antibiotic selection.**   1. **Complete Blood Count (CBC)**   **Rationale: Leukocytosis supports bacterial infection; anemia or thrombocytopenia may indicate systemic illness.**   1. **Pulse Oximetry**   **Rationale: Assess oxygenation status. Hypoxia (SpO₂ < 92%) may indicate severe pneumonia or PE, necessitating hospitalization or supplemental oxygen.**   1. **Serum Chemistry Profile**   **Rationale: Evaluates renal function (relevant for hypertension management and antibiotic dosing), electrolytes, and hydration status.**   1. **ECG**   **Rationale: Exclude cardiac causes of chest pain (e.g., ischemia, pericarditis) given his hypertension and smoking history.**  **Why Not Other Options?**   - **Arterial Blood Gas (ABG): Reserved for severe hypoxia, acid-base imbalance, or altered mental status. Pulse oximetry suffices initially.** - **Serum D-dimer: Useful if pulmonary embolism (PE) is suspected, but not first-line without clinical risk factors (e.g., immobility, DVT). Order if chest pain/dyspnea persists despite negative CXR.** - **Chest CT Scan: Consider if CXR is inconclusive or PE is high on the differential (e.g., elevated D-dimer, persistent hypoxia).** - **Serum Troponin: Indicated for suspected myocardial infarction, but pleuritic chest pain is more consistent with pneumonia/PE.**   **Final Answer:**   - **Chest X-ray** - **Blood and Sputum Cultures** - **Complete Blood Count (CBC)** - **Pulse Oximetry** - **Serum Chemistry Profile** - **ECG**   **Next Steps:**   - **If PE remains a concern (e.g., unexplained hypoxia, pleuritic pain), add D-dimer and consider CT Pulmonary Angiogram.** - **If heart failure is suspected (e.g., crackles, elevated BNP), order BNP/NT-proBNP and echocardiogram.** - **Monitor response to empiric antibiotics (e.g., amoxicillin/clavulanate) and adjust based on culture results.** | **Answer:** The following steps are most appropriate for guiding diagnosis and treatment:   - **Chest x-ray** - **Complete blood count (CBC)** - **Blood and sputum cultures** - **Pulse oximetry** - **Serum chemistry profile** - **ECG** - **Serum D-dimer level**   **Rationale:**   1. **Chest x-ray**: Essential to confirm pneumonia (e.g., consolidation) or identify alternative diagnoses (e.g., lung mass, effusion). 2. **CBC**: Assess for leukocytosis (supports bacterial infection) or anemia. 3. **Blood and sputum cultures**: Indicated due to systemic symptoms (shaking chills, fever) and productive cough. Cultures help identify pathogens, especially if hospitalization or atypical organisms are considered. 4. **Pulse oximetry**: Evaluates oxygenation status; hypoxia would escalate care urgency. 5. **Serum chemistry profile**: Assesses electrolytes, renal function, and hydration status, which guide treatment (e.g., IV fluids, antibiotic dosing). 6. **ECG**: Rules out cardiac causes of chest pain (e.g., ischemia, pericarditis), given his age and cardiovascular risk factors. 7. **Serum D-dimer level**: Pertinent to evaluate for **pulmonary embolism (PE)**, as pleuritic chest pain and dyspnea overlap with PE. A negative D-dimer may help exclude PE in low-to-intermediate risk patients.   **Excluded Options:**   - **Arterial blood gases (ABGs)**: Not necessary unless hypoxia (via pulse oximetry) or acid-base disturbance is suspected. - **Chest CT scan**: Reserved if chest x-ray is inconclusive, PE is strongly suspected (requires CT angiography), or complications (e.g., abscess) are considered. - **Serum troponin level**: Unlikely cardiac etiology given pleuritic pain and absence of typical ischemic symptoms. - **Middle East respiratory syndrome (MERS)**: No relevant travel/exposure history.   **Clinical Bottom Line:** The priority is to confirm **pneumonia** (chest x-ray) and assess severity (CBC, pulse oximetry). Blood/sputum cultures and ECG address infection and cardiac risks. D-dimer helps evaluate PE, which remains a differential. These steps ensure comprehensive evaluation while prioritizing the most likely diagnosis. | **Answer:** **• Blood and sputum cultures** **• Complete blood count (CBC)** **• Chest x-ray** **• ECG** **• Pulse oximetry** **• Serum chemistry profile** **• Serum D-dimer level**  **Rationale:**   1. **Chest X-ray**:   **Critical first step** to confirm pneumonia (e.g., consolidation, infiltrates) and rule out other causes of symptoms (e.g., pleural effusion, mass).   1. **Blood and Sputum Cultures**:   **Essential for identifying the causative pathogen** (e.g., *Streptococcus pneumoniae*) in suspected bacterial pneumonia. Guides targeted antibiotic therapy.   1. **CBC**:   Evaluates for leukocytosis (supports bacterial infection) or anemia.   1. **Pulse Oximetry**:   Assesses oxygenation status; dyspnea on exertion and pleuritic pain raise concern for hypoxemia.   1. **Serum Chemistry Profile**:   Checks renal function (for antibiotic dosing), electrolyte imbalances, and overall metabolic status.   1. **ECG**:   Rules out cardiac causes of chest pain (e.g., pericarditis, ischemia) and evaluates for arrhythmias secondary to hypoxia or sepsis.   1. **Serum D-dimer Level**:   **Necessary because pulmonary embolism (PE) remains a differential**. Elevated D-dimer prompts further imaging (e.g., CT angiography) if clinical suspicion persists.  **Excluded Options:**   - **Arterial Blood Gas (ABG)**: Not routinely needed unless severe hypoxia or acid-base imbalance is suspected (pulse oximetry suffices for initial assessment). - **Chest CT Scan**: Reserved if chest x-ray is inconclusive, PE is strongly suspected, or complications (e.g., abscess, effusion) are identified. - **Serum Troponin Level**: Unlikely in this case, as chest pain is pleuritic and cardiac risk factors are minimal.   **Key Considerations:**   - **D-dimer Interpretation**: Elevated in infections (e.g., pneumonia), so specificity for PE is low. Use clinical judgment and combine with Wells’ criteria. - **Follow-Up Imaging**: If chest x-ray is negative or PE remains a concern, proceed to CT angiography.   **Final Answer**: **Blood and sputum cultures** **Complete blood count (CBC)** **Chest x-ray** **ECG** **Pulse oximetry** **Serum chemistry profile** **Serum D-dimer level** |
| Dx | **Most Likely Diagnosis: Community-Acquired Pneumonia (CAP)**  **Key Supporting Evidence:**   1. **Chest X-ray: Left lower lobe infiltrate (hallmark of pneumonia).** 2. **Clinical Presentation: Likely includes symptoms such as fever, cough, purulent sputum, and pleuritic chest pain.** 3. **Hypoxia: Pulse oximetry 94% on room air (consistent with pneumonia severity).** 4. **Response to Antibiotics: Empiric CAP treatment (e.g., ceftriaxone + azithromycin) was initiated, aligning with typical CAP management.**   **Why Not Other Options?:**   - **Acute Bronchitis: Typically causes diffuse bronchial inflammation without focal lung infiltrates on imaging.** - **Healthcare-Associated Pneumonia (HCAP): Requires recent hospitalization, nursing home residence, or other healthcare exposures (not indicated here).** - **Lung Cancer: Presents with chronic symptoms (e.g., weight loss, hemoptysis) and imaging findings like a mass or nodule (not an acute infiltrate).**   **Conclusion: The combination of focal infiltrate on chest x-ray, hypoxia, and systemic symptoms confirms CAP as the diagnosis.** | **Answer:** **Community-acquired pneumonia**  **Rationale:** The **left lower lobe infiltrate on chest x-ray** confirms pneumonia, as acute bronchitis does not cause radiographic infiltrates. The term **"community-acquired"** applies because:   1. The patient lacks risk factors for **health care-associated pneumonia (HCAP)** (e.g., no recent hospitalization, long-term care residence, or dialysis). 2. Symptoms (fever, productive cough, pleuritic pain) and exam findings (crackles, hypoxia) align with typical **bacterial or atypical pneumonia** acquired in the community. 3. **Lung cancer** is unlikely to present acutely with fever and infiltrates; it would be considered if the infiltrate fails to resolve after treatment.   **Excluded Options:**   - **Acute bronchitis**: No infiltrate on chest x-ray; typically viral with self-limited cough. - **Health care-associated pneumonia (HCAP)**: Requires recent healthcare exposure (not indicated here). - **Lung cancer**: A chronic process; acute symptoms and infiltrate suggest infection, not malignancy.   **Clinical Bottom Line:** The chest x-ray infiltrate, combined with systemic and respiratory symptoms, definitively supports **community-acquired pneumonia** as the diagnosis. Immediate antibiotic therapy and severity assessment (e.g., CURB-65) are required. | **Answer:** **• Community-acquired pneumonia**  **Rationale:**   1. **Key Findings Supporting CAP**:   **Chest X-ray**: Left lower lobe infiltrate (definitive evidence of parenchymal lung infection).  **Symptoms**: Pleuritic chest pain, dyspnea on exertion, and hypoxemia (94% SpO₂).  **Clinical Context**: No indication of recent hospitalization, nursing home residence, or other healthcare exposures, ruling out healthcare-associated pneumonia (HCAP).   1. **Excluded Diagnoses**:   **Acute Bronchitis**: Typically presents with cough and normal chest X-ray (no infiltrates).  **Healthcare-Associated Pneumonia (HCAP)**: Requires recent healthcare contact (e.g., hospitalization, dialysis), which is not mentioned here.  **Lung Cancer**: Unlikely in an acute presentation with infiltrate. Cancer would typically show a mass/nodule or chronic symptoms (e.g., weight loss, hemoptysis).   1. **Diagnostic Certainty**:   The chest X-ray infiltrate confirms pneumonia. In the absence of healthcare exposure, **CAP is the most likely diagnosis**.  **Final Answer**: **Community-acquired pneumonia** |
| Mang | **Most Appropriate Treatment Orders:**   1. **Azithromycin 500 mg PO once/day, then 250 mg PO once/day**   **Rationale: Macrolides (e.g., azithromycin) are recommended for atypical pathogen coverage (e.g., *Mycoplasma*, *Legionella*) in combination with a beta-lactam for empiric CAP treatment.**   1. **Cefotaxime 1 g IV q8h**   **Rationale: Third-generation cephalosporins (e.g., cefotaxime) provide broad-spectrum coverage for typical bacterial pathogens (e.g., *Streptococcus pneumoniae*). Combined with azithromycin, this aligns with IDSA/ATS guidelines for hospitalized CAP patients.**   1. **Oxygen 4 L/min by nasal cannula**   **Rationale: While the patient’s SpO₂ is 94% on room air, supplemental oxygen may still be warranted if there are signs of respiratory distress, worsening hypoxia, or comorbidities (e.g., COPD). Close monitoring is essential.**  **Why Not Other Options?:**   - **Schedule follow-up chest x-ray in 6 weeks: Not routinely indicated unless the patient is high-risk (e.g., smoking history, suspicion of malignancy) or fails to improve.** - **Chest physical therapy: Not recommended for uncomplicated CAP; reserved for conditions like bronchiectasis or retained secretions.** - **Albuterol inhaler: Only indicated if wheezing or underlying asthma/COPD is present (not mentioned in this case).** - **Discontinue enalapril: No evidence of hypotension or acute kidney injury to justify stopping an ACE inhibitor.** - **Guaifenesin/dextromethorphan: Symptomatic relief for cough is optional but not a core treatment. Dextromethorphan may suppress productive coughing, which is counterproductive in pneumonia.**   **Conclusion: The priority is dual antibiotic therapy (cefotaxime + azithromycin) to cover both typical and atypical pathogens. Supplemental oxygen should be considered based on clinical context. Other options are either unnecessary or lack evidence for routine use in uncomplicated CAP.**  **Selected Orders:**   - **Azithromycin 500 mg PO once/day, then 250 mg PO once/day** - **Cefotaxime 1 g IV q8h** - **Oxygen 4 L/min by nasal cannula (if clinical judgment supports mild hypoxia or patient distress).** | **Appropriate Treatment Orders:**   1. **Schedule follow-up visit in 6 wk for repeat chest x-ray**:   Essential to confirm resolution of the infiltrate and exclude underlying pathology (e.g., lung cancer).   1. **Azithromycin 500 mg po once/day, then 250 mg po once/day**:   First-line empiric therapy for community-acquired pneumonia (CAP), covering atypical pathogens (e.g., *Mycoplasma*, *Chlamydia*). Often combined with a beta-lactam (e.g., cefotaxime) for broader coverage.   1. **Cefotaxime 1 g IV q 8 h**:   A third-generation cephalosporin targeting typical bacterial pathogens (e.g., *Streptococcus pneumoniae*). Indicated for hospitalized patients or severe CAP requiring IV therapy.   1. **Guaifenesin/dextromethorphan syrup qid**:   Symptomatic relief for cough (guaifenesin thins secretions; dextromethorphan suppresses cough). Use cautiously, as cough is protective but may improve comfort.  **Excluded Options:**   - **Oxygen 4 L/min by nasal cannula**:   Not required unless SpO₂ < 90% or the patient is in respiratory distress (94% on room air is acceptable).   - **Chest physical therapy twice a day**:   Not routinely indicated for uncomplicated pneumonia (reserved for retained secretions or atelectasis).   - **Albuterol inhaler**:   Unnecessary unless wheezing or underlying asthma/COPD is present.   - **Discontinue enalapril**:   Only warranted if ACE inhibitor-induced cough persists post-recovery (no evidence of current cough attributed to enalapril).  **Clinical Rationale:** The combination of **azithromycin** (atypical coverage) and **cefotaxime** (gram-positive/enteric coverage) aligns with guidelines for empiric CAP treatment in hospitalized patients. Follow-up imaging ensures resolution and excludes malignancy. Symptomatic care with guaifenesin/dextromethorphan is reasonable for cough management. | **Answer:** **• Oxygen 4 L/min by nasal cannula** **• Azithromycin 500 mg po once/day, then 250 mg po once/day** **• Cefotaxime 1 g IV q 8 h**  **Rationale:**   1. **Oxygen Therapy (4 L/min)**:   The patient’s SpO₂ of 94% on room air indicates mild hypoxia. Supplemental oxygen is recommended to maintain SpO₂ ≥94% in pneumonia, especially if dyspnea or clinical instability is present.   1. **Antibiotic Therapy**:   **Cefotaxime (IV)**: A third-generation cephalosporin targeting typical bacterial pathogens (e.g., *Streptococcus pneumoniae*).  **Azithromycin (Oral)**: Covers atypical pathogens (e.g., *Mycoplasma pneumoniae*) and provides synergy in severe cases. Combination therapy aligns with IDSA/ATS guidelines for hospitalized CAP patients.   1. **Excluded Options**:   **Follow-Up Chest X-ray**: Not an immediate treatment; reserved for unresolved symptoms or high-risk patients (e.g., smokers, elderly).  **Chest Physical Therapy**: Not routinely indicated for uncomplicated CAP.  **Albuterol Inhaler**: No evidence of bronchospasm or underlying asthma/COPD.  **Discontinue Enalapril**: No rationale (e.g., hypotension, AKI) provided.  **Guaifenesin/Dextromethorphan**: Symptomatic relief is optional but not essential for treatment.  **Key Considerations:**   - **Severity Assessment**: Hypoxia (SpO₂ 94%) suggests moderate severity, warranting hospitalization and IV antibiotics. - **Guideline Adherence**: Dual therapy (beta-lactam + macrolide) is standard for inpatient CAP to cover both typical and atypical pathogens.   **Final Answer**: **Oxygen 4 L/min by nasal cannula** **Azithromycin 500 mg po once/day, then 250 mg po once/day** **Cefotaxime 1 g IV q 8 h** |
| Case8 |  |  |  |
| Diff | **Differential Diagnoses That Cannot Be Excluded: Based on the history and physical exam alone, the following diagnoses require further investigation and cannot be definitively ruled out:**   1. **Pulmonary Embolism (PE)**   **Sudden dyspnea and back pain, even without classic pleuritic pain or tachycardia. Smoking is a minor risk factor. Requires imaging (e.g., CTA or V/Q scan) or D-dimer.**   1. **Spontaneous Pneumothorax**   **Classic presentation: sudden dyspnea in a young smoker, unilateral decreased breath sounds. Chest X-ray is needed for confirmation.**   1. **Thoracic Aortic Dissection**   **Sudden-onset back pain and dyspnea. Though less likely (normal BP, no radiation/tearing pain), imaging (e.g., CT angiography) is required to exclude.**   1. **Myocardial Infarction (MI)**   **Atypical presentations can include back pain and dyspnea. ECG and troponin are necessary to exclude, despite low probability in a young patient.**   1. **Pericarditis**   **May present with positional chest/back pain, though no friction rub or ECG changes noted. Requires ECG and echocardiography.**   1. **Pneumonia**   **Decreased breath sounds could suggest consolidation. Absence of fever/cough reduces likelihood, but chest X-ray is needed.**   1. **Asthma Exacerbation**   **Dyspnea on exertion, though no wheezing or prior history. Trial of bronchodilators or spirometry would help exclude.**   1. **Musculoskeletal Back Pain**   **Pain is present but lacks exam findings (e.g., tenderness, reproducible pain). Imaging or response to analgesics could clarify.**  **Excluded Diagnoses:**   - **Lung Cancer: Unlikely given acute presentation and age, though smoking is a risk.** - **Tension Pneumothorax: Excluded due to stable vital signs (no hypotension, tracheal deviation, or severe distress).**   **Conclusion: The priority is to rule out life-threatening conditions (e.g., PE, aortic dissection, pneumothorax) and MI. Immediate imaging (CXR, CT) and ancillary testing (ECG, troponin) are critical. Symptomatic diagnoses (e.g., musculoskeletal pain, asthma) remain possible but less urgent.**  **Selected Options:**   - **Pulmonary embolism** - **Spontaneous pneumothorax** - **Thoracic aortic dissection** - **Myocardial infarction** - **Pericarditis** - **Pneumonia** - **Asthma exacerbation** - **Musculoskeletal back pain** | **Differential Diagnoses That Cannot Be Excluded:**   1. **Pulmonary embolism (PE)**   Sudden dyspnea and pleuritic pain are hallmark features. Normal SpO₂ and lack of tachycardia reduce likelihood but do not exclude PE. Requires imaging (e.g., CT pulmonary angiogram) or D-dimer.   1. **Spontaneous pneumothorax**   Classic presentation with sudden dyspnea, unilateral decreased breath sounds, and smoking history. Chest X-ray is required for confirmation.   1. **Myocardial infarction (MI)**   Rare in young patients but cannot be excluded without ECG and troponin, especially with exertion-associated symptoms.   1. **Pericarditis**   May present with sharp, positional chest/back pain. Absence of friction rub or ECG changes on exam does not rule it out.   1. **Pneumonia**   Lack of fever/cough makes this less likely, but focal decreased breath sounds could indicate consolidation. Chest X-ray is needed.   1. **Thoracic aortic dissection**   Unlikely given normal BP and absence of tearing/radiating pain, but dissection cannot be definitively excluded without imaging (e.g., CTA).  **Excluded Diagnoses:**   - **Asthma exacerbation**: No wheezing, cough, or prior history. - **Lung cancer**: Acute symptoms and age make this improbable; chronic signs (e.g., weight loss) are absent. - **Musculoskeletal back pain**: Pain is nonreproducible, unrelated to movement, and nontender on exam. - **Tension pneumothorax**: Stable vital signs (no hypotension, tracheal deviation, or tachycardia) exclude this emergency.   **Clinical Rationale:** While **spontaneous pneumothorax** and **PE** are the leading considerations, other life-threatening diagnoses (e.g., MI, aortic dissection) and mimics (e.g., pericarditis, pneumonia) require exclusion through imaging, biomarkers, or further testing. Immediate chest X-ray, ECG, and D-dimer/CTA are critical next steps. | **Differential Diagnoses That Cannot Be Excluded:**   1. **Pulmonary Embolism (PE)**:   Sudden dyspnea and pleuritic-type pain (even though pain is non-radiating). Smoking is a minor risk factor. PE can present without tachycardia or hypoxia. Requires imaging (CTPA) or D-dimer to exclude.   1. **Spontaneous Pneumothorax**:   Leading diagnosis given sudden dyspnea, decreased breath sounds on the right, and smoking history. Requires chest X-ray for confirmation.   1. **Myocardial Infarction (MI)**:   Unlikely in a 24-year-old, but MI can rarely occur in young smokers. Normal ECG and troponin would exclude this.   1. **Pericarditis**:   Sharp back pain and dyspnea could align with pericarditis. Lack of friction rub or positional pain makes this less likely, but not definitively excluded without ECG or echocardiogram.   1. **Pneumonia**:   Unlikely given absence of fever, cough, or sputum. However, atypical pneumonia (e.g., *Mycoplasma*) can present with mild symptoms. Chest X-ray would clarify.  **Excluded Diagnoses:**   - **Asthma Exacerbation**: No wheezing, history of asthma, or response to bronchodilators. - **Lung Cancer**: Extremely rare at 24 years old, even with smoking. No systemic symptoms (weight loss, chronic cough). - **Musculoskeletal Back Pain**: Pain is not exacerbated by movement, and exam shows no tenderness. - **Tension Pneumothorax**: Patient is hemodynamically stable with normal BP and no tracheal deviation. - **Thoracic Aortic Dissection**: No tearing/ripping pain, hypertension, or pulse deficits.   **Key Next Steps**:   - **Chest X-ray** (upright, expiratory view) to evaluate for pneumothorax or infiltrates. - **CTPA or D-dimer** if PE remains a concern. - **ECG and troponin** to assess for MI/pericarditis.   **Final Answer**: **Pulmonary Embolism, Spontaneous Pneumothorax, Myocardial Infarction, Pericarditis, Pneumonia** |
| Diag | **Most Appropriate Steps at This Time:**   1. **Chest X-ray:**   **Critical to evaluate for pneumothorax (decreased breath sounds on the right) or other lung pathology (e.g., consolidation). First-line imaging for acute dyspnea with unilateral findings.**   1. **ECG:**   **Essential to rule out cardiac causes (e.g., MI, pericarditis), even in a young patient. Atypical presentations of MI can include back pain and dyspnea.**   1. **Oxygen 6 L by nasal cannula:**   **Indicated for hypoxia (SpO₂ 94%). Improves oxygenation while diagnostic workup proceeds.**   1. **CT Angiography (CTA) of the Chest:**   **Necessary if chest X-ray is negative to evaluate for pulmonary embolism (PE) or thoracic aortic dissection. Sudden-onset dyspnea and back pain in a smoker raise suspicion for PE, and dissection, though less likely, cannot be excluded without imaging.**  **Not Immediately Indicated:**   - **D-dimer Testing: Less useful here due to the patient’s hypoxia and moderate clinical suspicion for PE. CTA is more definitive if chest X-ray is negative.** - **Thoracic Spine X-rays: Pain lacks musculoskeletal features (no tenderness, no exacerbation with movement). Chest X-ray or CT will assess thoracic structures.**   **Conclusion: The immediate steps are chest X-ray, ECG, and oxygen. CTA should follow if the chest X-ray is non-diagnostic or if clinical suspicion for PE/aortic dissection persists.**  **Selected Options:**   - **Chest X-ray** - **CT angiography (CTA) of the chest** - **ECG** - **Oxygen 6 L by nasal cannula** | **Most Appropriate Steps at This Time:**   1. **Chest X-ray**: Essential to evaluate for pneumothorax, pneumonia, or other lung pathology (e.g., decreased breath sounds on the right and hypoxia). 2. **ECG**: Required to assess for cardiac causes (e.g., myocardial infarction, pericarditis) given exertion-associated symptoms and back pain. 3. **Oxygen 6 L by nasal cannula**: Mild hypoxia (SpO₂ 94%) warrants supplemental oxygen to improve oxygenation, especially if a pneumothorax is confirmed.   **Additional Considerations Based on Initial Results:**   - **CT angiography (CTA) of the chest：** - If chest X-ray is negative and suspicion for pulmonary embolism (PE) or aortic dissection persists. - **D-dimer testing**: May be considered if PE is suspected after initial imaging, though clinical probability should guide its use (low pretest probability in this young patient makes PE less likely).   **Not Recommended:**   - **Thoracic spine X-rays**: No history of trauma, tenderness, or focal neurologic deficits to suggest musculoskeletal injury or spinal pathology.   **Clinical Rationale:** The priority is to confirm/exclude **spontaneous pneumothorax** (chest X-ray) and evaluate for cardiac pathology (ECG). Supplemental oxygen addresses mild hypoxia while diagnostics proceed. Further testing (e.g., CTA, D-dimer) depends on initial findings. | **Most Appropriate Steps at This Time:**   1. **Chest X-ray**: Essential to confirm or rule out pneumothorax, given decreased breath sounds on the right and sudden dyspnea. 2. **ECG**: Necessary to evaluate for cardiac causes (e.g., MI, pericarditis) due to sharp back pain and dyspnea. 3. **Oxygen 6 L by nasal cannula**: Indicated to improve borderline SpO₂ (94%) and support oxygenation. 4. **D-dimer testing**: Useful to assess for pulmonary embolism (PE) given acute symptoms, though low pretest probability (young age, no tachycardia, no clear risk factors beyond smoking). A negative D-dimer would effectively rule out PE in this context.   **Excluded Options:**   - **CT Angiography (CTA) of the Chest**: Not immediately necessary unless Chest X-ray is negative *and* D-dimer is elevated or clinical suspicion for PE remains high. - **Thoracic Spine X-rays**: Unlikely to explain the symptoms (non-tender back, pain not exacerbated by movement).   **Rationale**:   - **Chest X-ray** is the priority to diagnose pneumothorax. - **ECG** rules out cardiac pathology (MI/pericarditis), especially given sharp back pain. - **Oxygen** addresses borderline hypoxia and is supportive. - **D-dimer** is a low-risk screening tool for PE in a patient with low pretest probability. If negative, PE is unlikely, avoiding unnecessary radiation from CTA.   **Final Answer**: **Chest X-ray, ECG, Oxygen 6 L by nasal cannula, D-dimer testing** |
| Mang | **Essential Immediate Steps:**  **1. Admit the patient to the hospital**   - **Critical regardless of chest X-ray findings. A pneumothorax requires inpatient management, and unexplained dyspnea/hypoxia (even with a normal X-ray) demands further evaluation.**   **2. Continue oxygen 6 L by nasal cannula**   - **Indicated for hypoxia (SpO₂ 94%). Oxygen aids pneumothorax resolution and supports oxygenation while diagnostic workup continues.**   **3. Place a 20 French chest tube on the right side**   - **If pneumothorax is present and large/symptomatic (e.g., respiratory distress, significant air rim on X-ray). Smaller tubes (20-24F) are standard for spontaneous pneumothorax.**   **4. Do immediate needle decompression**   - **Only if tension pneumothorax is suspected (e.g., hypotension, tracheal deviation, absent breath sounds). This is a life-saving intervention.**   **5. CT scan of the chest with IV contrast**   - **If the chest X-ray is normal, proceed to CT angiography (CTA) to rule out pulmonary embolism (PE) or aortic dissection, given the patient’s risk factors (smoking, sudden-onset dyspnea/back pain).**   **Not Indicated:**   - **Administer azithromycin: No evidence of infection (e.g., fever, purulent sputum) in this scenario.** - **CT without IV contrast: Less useful for evaluating PE/dissection; CTA (with contrast) is definitive.** - **Discharge the patient: Unsafe with hypoxia or pneumothorax.** - **36 French chest tube: Reserved for traumatic hemothorax, not spontaneous pneumothorax.** - **100% oxygen via non-rebreather: Reserved for severe hypoxia unresponsive to 6 L O₂.** - **VATS/pleurodesis: Elective surgery, not an immediate intervention.**   **Key Takeaway:**   - **Pneumothorax: Admit, oxygen, and chest tube (20F) or needle decompression (if tension).** - **Normal X-ray: Admit, oxygen, and CTA with contrast to exclude PE/dissection.**   **Selected Options:**   - **Admit the patient to the hospital** - **Continue oxygen 6 L by nasal cannula** - **Place a 20 French chest tube on the right side** - **Do immediate needle decompression** - **CT scan of the chest with IV contrast** | **Essential Immediate Steps:**   1. **Admit the patient to the hospital**: Mandatory for monitoring and management, given the confirmed pneumothorax and hypoxia (SpO₂ 94%). 2. **Continue oxygen 6 L by nasal cannula**: Maintains oxygenation and aids pneumothorax resolution by creating a nitrogen gradient. 3. **Place a 20 French chest tube on the right side**: Indicated for symptomatic or moderate-to-large pneumothorax (especially with hypoxia). Small-bore tubes (e.g., 20Fr) are standard for primary spontaneous pneumothorax.   **Not Immediately Essential:**   - **Needle decompression**: Reserved for **tension pneumothorax** (not described here; no hypotension, tracheal deviation, or absent breath sounds). - **36 French chest tube**: Typically used for trauma or hemopneumothorax, not spontaneous cases. - **CT scans (with/without contrast)**: Not urgent unless atypical features (e.g., suspected PE or dissection) persist after initial management. - **Discharge or outpatient follow-up**: Unsafe with active pneumothorax and hypoxia. - **Azithromycin**: No evidence of infection (e.g., fever, productive cough) to warrant antibiotics. - **100% oxygen via non-rebreathing mask**: Reserved for severe hypoxia (current SpO₂ 94% is manageable with 6L nasal cannula). - **Surgery (VATS/pleurodesis)**: Considered for recurrent pneumothoraces, not an emergency intervention.   **Clinical Rationale:**   - Immediate priorities are **stabilization** (oxygen), **definitive treatment** (chest tube for pneumothorax), and **monitoring** (admission). - Smaller chest tubes (20Fr) are preferred for primary spontaneous pneumothorax to minimize complications. - Escalation to surgery or advanced imaging depends on response to initial treatment. | **Essential Immediate Steps Based on Chest X-ray Findings:**  **If Chest X-ray Shows Right-Sided Pneumothorax:**   1. **Admit the patient to the hospital**: Pneumothorax requires inpatient management. 2. **Continue oxygen 6 L by nasal cannula**: Enhances pneumothorax resolution and maintains oxygenation. 3. **Place a 20 French chest tube on the right side**: Standard for symptomatic or large pneumothorax.   **Excluded Options**:   - **Needle decompression**: Only for tension pneumothorax (no clinical signs provided). - **36 French chest tube**: Used for hemothorax/empyema, not simple pneumothorax. - **Discharge/surgery**: Not appropriate in the acute setting.   **If Chest X-ray is Normal:**   1. **Admit the patient to the hospital**: Further evaluation for PE or other causes is needed. 2. **Continue oxygen 6 L by nasal cannula**: Maintain SpO₂ >94% if hypoxia persists. 3. **CT scan of the chest with IV contrast (CTA)**: Rules out pulmonary embolism (PE).   **Excluded Options**:   - **Chest tube/needle decompression**: No pneumothorax evident. - **Azithromycin**: No evidence of infection. - **Non-contrast CT**: Less useful than CTA for PE diagnosis.   **Final Answer**:   - **For Pneumothorax**: **Admit, Continue oxygen, Place a 20 French chest tube** - **For Normal X-ray**: **Admit, Continue oxygen, CT scan with IV contrast** |
| Case9 |  |  |  |
| Diff | **Critical Differential Diagnoses to Consider in This Patient:**  **1. Acute Decompensated Heart Failure (ADHF)**   - **Rationale: Despite clear lung fields, elevated jugular venous pressure (JVP) and lower extremity edema suggest fluid overload. ESRD patients often have diastolic dysfunction or uremic cardiomyopathy, which can lead to ADHF.**   **2. Aortic Stenosis**   - **Rationale: Crescendo-decrescendo systolic murmur at the right upper sternal border radiating to the carotids and delayed carotid upstrokes are classic findings. Severe aortic stenosis can cause exertional dyspnea, chest pressure, and syncope.**   **3. Coronary Artery Disease (CAD)**   - **Rationale: Substernal chest pressure on exertion, even if atypical, in a patient with multiple cardiovascular risk factors (diabetes, hypertension, ESRD) mandates evaluation for CAD. Silent ischemia is common in ESRD.**   **4. Anemia**   - **Rationale: ESRD patients are at high risk for anemia due to erythropoietin deficiency and chronic inflammation. Anemia exacerbates dyspnea and fatigue. Labs (CBC) are needed to confirm.**   **5. Volume Overload from ESRD**   - **Rationale: Despite adherence to dialysis, inadequate ultrafiltration or dietary noncompliance can lead to chronic fluid retention, contributing to edema and exertional symptoms.**   **Additional Considerations (Less Likely but Not Fully Excluded):**   - **Pulmonary Embolism (PE):**   **Chronic dyspnea makes acute PE less likely, but ESRD patients are at increased thrombotic risk. However, no acute symptoms (e.g., pleuritic pain, hemoptysis) or obvious risk factors (e.g., immobility) are noted.**   - **Mitral Regurgitation:**   **The murmur’s location and radiation are more consistent with aortic stenosis, but valvular pathology cannot be excluded without echocardiography.**  **Excluded Diagnoses:**   - **Chronic Histoplasmosis/Tuberculosis: No systemic symptoms (fever, weight loss, cough) or exposure history.** - **COPD: No smoking history or obstructive lung findings (wheezes, hyperinflation).** - **Pneumonia: Absence of fever, cough, or lung consolidation on exam.**   **Key Next Steps:**   1. **Echocardiogram: Confirm aortic stenosis and assess ejection fraction.** 2. **ECG: Evaluate for ischemia, arrhythmias, or LVH.** 3. **Chest X-ray/BNP/Troponin: Assess cardiac size, pulmonary congestion, and rule out myocardial injury.** 4. **CBC/Electrolytes: Evaluate anemia and dialysis adequacy.**   **Selected Options:**   - **Acute decompensated heart failure (ADHF)** - **Anemia** - **Aortic stenosis** - **Coronary artery disease (CAD)** - **Volume overload from end-stage renal disease** | **Common and/or Dangerous Differentials That Cannot Be Excluded:**   1. **Acute Decompensated Heart Failure (ADHF)**   Despite clear lungs, ESRD patients may have fluid overload without pulmonary edema. Leg edema, exertional dyspnea, and elevated JVP (borderline at 5 cm) are suggestive.   1. **Anemia**   Common in ESRD despite darbepoietin. Fatigue and dyspnea could reflect anemia, though labs are needed for confirmation.   1. **Aortic Stenosis (AS)**   Classic exam findings (crescendo-decrescendo murmur, diminished S2, delayed carotid upstrokes) and exertional symptoms strongly suggest AS.   1. **Coronary Artery Disease (CAD)**   Atypical chest pressure + risk factors (diabetes, hypertension, ESRD) warrant exclusion of CAD, especially since AS and CAD often coexist.   1. **Volume Overload from ESRD**   Leg edema and hypertension may indicate inadequate ultrafiltration during dialysis, even with clear lung exam.   1. **Pulmonary Embolism (PE)**   ESRD increases thrombotic risk. Though symptoms are chronic, PE can rarely present insidiously. Hypoxia or tachycardia may be absent.  **Excluded Differentials:**   - **Atrial fibrillation**: Regular pulse (72/min) and no palpitations make this unlikely. - **Chronic histoplasmosis/COPD/Pneumonia/Tuberculosis**: No infectious symptoms (fever, cough) or risk factors (smoking, exposure). - **Mitral regurgitation**: Murmur location/quality inconsistent with MR.   **Key Considerations:**   - **ADHF** and **volume overload** are intertwined in ESRD but require distinct management (e.g., dialysis adjustment vs. diuretics). - **Aortic stenosis** and **CAD** are life-threatening if undiagnosed; echocardiography and troponin/ECG are critical. - **PE**, though less likely, remains a dangerous "can't miss" diagnosis in high-risk patients.   **Bottom Line:** Prioritize cardiac evaluation (echo, ECG, biomarkers) and address volume status while ruling out PE if clinical suspicion arises. | **Differential Diagnoses That Cannot Be Excluded**:  **Common and Dangerous Diagnoses to Consider:**   1. **Acute Decompensated Heart Failure (ADHF)**:   **Rationale**: Elevated JVP (5 cm), lower extremity edema, exertional dyspnea, and fatigue suggest fluid overload. ESRD patients often have diastolic dysfunction or uremic cardiomyopathy, which can mimic ADHF. Clear lung fields do not rule out compensated ADHF.   1. **Aortic Stenosis**:   **Rationale**: Crescendo-decrescendo systolic murmur at the right upper sternal border radiating to carotids, delayed carotid upstrokes, and diminished S2 are classic findings. Exertional dyspnea and chest pressure align with symptomatic aortic stenosis.   1. **Coronary Artery Disease (CAD)**:   **Rationale**: Substernal chest pressure on exertion, diabetes, hypertension, and ESRD (accelerates atherosclerosis) are strong risk factors. Silent ischemia is common in diabetic patients.   1. **Anemia**:   **Rationale**: ESRD patients are at high risk for anemia due to erythropoietin deficiency. Fatigue and exertional dyspnea may worsen with anemia. No CBC provided to exclude this.   1. **Volume Overload from ESRD**:   **Rationale**: Lower extremity edema and elevated JVP suggest inadequate ultrafiltration during dialysis. ESRD patients often struggle with interdialytic fluid retention.  **Excluded Diagnoses:**   - **Atrial Fibrillation**: Regular rate/rhythm on exam makes this unlikely. - **Chronic Histoplasmosis/COPD/Pneumonia/Tuberculosis**: No respiratory symptoms (cough, fever, sputum) or risk factors (smoking, exposure). - **Mitral Regurgitation**: No holosystolic murmur or signs of left atrial enlargement. - **Pulmonary Embolism (PE)**: No acute dyspnea, hypoxia, or risk factors (immobility, hypercoagulability not mentioned).   **Final Answer**:   - **Acute decompensated heart failure (ADHF)** - **Anemia** - **Aortic stenosis** - **Coronary artery disease (CAD)** - **Volume overload from end-stage renal disease**   These diagnoses are common in ESRD patients, align with the clinical findings, and require urgent evaluation (e.g., echocardiogram, labs, dialysis adjustment). |
| Diag | **Most Appropriate Next Steps:**   1. **Chest X-ray (CXR)**   **Rationale: Assess for pulmonary congestion (e.g., cephalization, interstitial edema) to evaluate for fluid overload or ADHF, even with clear lung fields on exam. Also evaluates cardiac size and aortic calcifications.**   1. **Complete Blood Count (CBC)**   **Rationale: ESRD patients are at high risk for anemia due to erythropoietin deficiency. Anemia exacerbates dyspnea and fatigue; results may guide darbepoietin dosing or iron supplementation.**   1. **Electrocardiogram (ECG)**   **Rationale: Evaluate for ischemic changes (ST-segment/T-wave abnormalities), left ventricular hypertrophy (LVH), or arrhythmias (e.g., atrial fibrillation). LVH is common in aortic stenosis and hypertension.**   1. **Serum Brain Natriuretic Peptide (BNP)**   **Rationale: Elevated BNP supports heart failure diagnosis, though levels are often chronically elevated in ESRD. Trends may help differentiate volume overload from other causes.**   1. **Serum Troponin I Level**   **Rationale: ESRD patients have elevated baseline troponin, but an acute rise suggests myocardial injury. Critical to rule out ACS given exertional chest pressure and cardiovascular risk factors.**   1. **Transthoracic Echocardiography (TTE)**   **Rationale: Gold standard to confirm aortic stenosis (gradients, valve area), assess ejection fraction, and evaluate for other structural abnormalities (e.g., LVH, diastolic dysfunction).**  **Options to Avoid:**   - **Chest CT Scan: No clinical suspicion for pulmonary embolism (no pleuritic pain, hemoptysis, or acute dyspnea) or infection. Risks of contrast nephropathy are mitigated by dialysis, but low yield here.** - **Transesophageal Echocardiography (TEE): Not first-line unless TTE is inconclusive or suspicion for endocarditis (no fever, murmurs typical for aortic stenosis, no embolic phenomena).**   **Key Priorities:**   - **TTE is critical to confirm aortic stenosis severity and guide intervention (e.g., TAVR).** - **CXR, BNP, and CBC address fluid status and anemia, both reversible contributors to dyspnea.** - **ECG and troponin evaluate for acute ischemia or arrhythmias in a high-risk patient.**   **Selected Options:**   - **Chest x-ray (CXR)** - **Complete blood count (CBC)** - **Electrocardiogram (ECG)** - **Serum brain natriuretic peptide (BNP) level** - **Serum troponin I level** - **Transthoracic echocardiography (TTE)** | **Most Appropriate Next Steps:**   1. **Transthoracic Echocardiography (TTE)**   **Critical** to confirm aortic stenosis severity (gradient, valve area), assess left ventricular function, and evaluate for other structural abnormalities (e.g., left ventricular hypertrophy).   1. **Electrocardiogram (ECG)**   Evaluate for ischemia, arrhythmias, or left ventricular strain pattern (e.g., LVH with "strain" suggests AS or hypertensive heart disease).   1. **Serum Troponin I Level**   Rule out acute coronary syndrome (ACS), especially given atypical chest pressure and high CAD risk in ESRD. Chronic troponin elevation may occur in ESRD, but a rising trend is concerning.   1. **Complete Blood Count (CBC)**   Exclude anemia (common in ESRD despite darbepoietin), which can exacerbate dyspnea and fatigue.   1. **Chest X-ray (CXR)**   Assess cardiac size, pulmonary congestion (even with clear lungs on exam), and exclude other pulmonary pathology.   1. **Serum Brain Natriuretic Peptide (BNP) Level**   Elevated BNP supports volume overload or ADHF, though interpretation is challenging in ESRD (chronic elevation due to reduced clearance). A very high BNP may still indicate acute decompensation.  **Less Likely/Not Immediately Necessary:**   - **Chest CT Scan**: Low suspicion for PE (chronic symptoms, no hypoxia/tachycardia), but consider if dyspnea worsens or hypoxia develops. - **Transesophageal Echocardiography (TEE)**: Reserved if TTE is inconclusive (e.g., poor acoustic windows) or to evaluate for endocarditis (not suspected here).   **Rationale:**   - **TTE** is the cornerstone for diagnosing aortic stenosis and guiding intervention (e.g., TAVR vs. surgery). - **ECG and Troponin** are essential to evaluate for CAD, which commonly coexists with AS and ESRD. - **CXR and BNP** help assess fluid status and cardiac remodeling. - **CBC** addresses anemia, a reversible contributor to symptoms.   **Bottom Line:** Prioritize **TTE, ECG, Troponin, CBC, CXR, and BNP** to confirm aortic stenosis, exclude ischemia/anemia, and assess fluid status. | **Most Appropriate Next Steps**:  **Essential Tests for Diagnosis and Management:**   1. **Transthoracic Echocardiography (TTE)**:   **Rationale**: Gold standard to evaluate aortic stenosis severity, ejection fraction, and structural heart disease (e.g., wall motion abnormalities from CAD). Critical for guiding valve intervention or heart failure management.   1. **Complete Blood Count (CBC)**:   **Rationale**: ESRD patients are at high risk for anemia (due to erythropoietin deficiency or iron loss), which exacerbates dyspnea and fatigue. Adjust darbepoietin dose if anemia is confirmed.   1. **Electrocardiogram (ECG)**:   **Rationale**: Assess for ischemic changes (ST depressions/elevations), arrhythmias, or left ventricular hypertrophy (common in aortic stenosis or hypertensive heart disease).   1. **Serum Brain Natriuretic Peptide (BNP)**:   **Rationale**: Elevated BNP supports heart failure diagnosis. While BNP is often elevated in ESRD, a very high level (>500 pg/mL) suggests ADHF.   1. **Serum Troponin I Level**:   **Rationale**: ESRD patients are at high risk for silent ischemia. Elevated troponin may indicate coronary artery disease or demand ischemia (e.g., from aortic stenosis).   1. **Chest X-ray (CXR)**:   **Rationale**: Evaluate for cardiomegaly, pulmonary congestion (even subtle), or pleural effusions. Helps confirm volume overload or ADHF.  **Tests to Defer or Exclude:**   - **Chest CT Scan**: No signs of PE, pneumonia, or acute pathology. Not urgent without hypoxia or focal findings. - **Transesophageal Echocardiography (TEE)**: Not first-line unless TTE is inconclusive or suspicion for endocarditis/aortic dissection.   **Final Answer**:   - **Chest x-ray (CXR)** - **Complete blood count (CBC)** - **Electrocardiogram (ECG)** - **Serum brain natriuretic peptide (BNP) level** - **Serum troponin I level** - **Transthoracic echocardiography (TTE)**   These steps address the key differentials (aortic stenosis, ADHF, CAD, anemia, and volume overload) and guide urgent management (e.g., valve intervention, dialysis optimization, anemia correction). |
| Dx | **Answer: Severe aortic stenosis**  **Rationale:**   1. **Echocardiography Confirmation:**   **Severe calcific aortic stenosis (mean gradient 45 mmHg, AVA 0.85 cm²) meets criteria for severe AS.**  **Symptoms (exertional dyspnea, chest pressure) are classic for symptomatic severe AS.**   1. **Exclusion of Other Diagnoses:**   **Acute Coronary Ischemia:**  **Troponin elevation is mild and chronic (common in ESRD).**  **No ECG ischemic changes or wall motion abnormalities on echo.**  **Acute Decompensated Heart Failure (ADHF):**  **No pulmonary congestion on CXR.**  **Preserved LVEF (60%) and normal diastolic function on echo.**  **Volume Overload Due to ESRD:**  **Mild pleural effusion on CXR but no significant pulmonary edema.**  **Symptoms are more consistent with AS than isolated fluid overload.**   1. **Clinical Priority:**   **Severe AS is life-threatening and requires urgent intervention (TAVR/SAVR).**  **Symptoms are directly attributable to AS, not secondary to ESRD complications.**  **Conclusion: Severe aortic stenosis is the primary diagnosis driving the patient’s symptoms. Other factors (anemia, mild volume overload) are contributory but not the main cause.** | **Most Likely Diagnosis:** **Severe Aortic Stenosis**  **Rationale:**   1. **Definitive Echo Findings:** Severe AS is confirmed by TTE (mean gradient 45 mm Hg, AVA 0.85 cm²), meeting diagnostic criteria. 2. **Symptom Correlation:** Exertional dyspnea and atypical chest pressure are classic symptoms of severe AS. 3. **Supporting Evidence:**   LVH on ECG and echo aligns with chronic pressure overload from AS.  Elevated BNP and mild volume overload (costophrenic angle blunting) are secondary to AS-induced cardiac remodeling and diastolic dysfunction, not primary ADHF.   1. **Exclusion of Alternatives:**   **Acute Coronary Ischemia:** Troponin elevation is mild and likely chronic (ESRD-related); no ECG/echo evidence of ischemia.  **ADHF:** Normal LVEF (60%), clear lung fields on CXR, and absence of pulmonary edema argue against acute decompensation.  **Volume Overload (ESRD):** Mild fluid retention is present but insufficient to explain severe symptoms; it is a comorbidity, not the primary diagnosis.  **Conclusion:** Severe aortic stenosis is the primary driver of symptoms and hemodynamic compromise. While volume overload and anemia contribute to fatigue, they are not the root cause of the patient’s clinical presentation. | **Most Likely Diagnosis**: **Severe aortic stenosis**  **Rationale**:   1. **Symptomatic Severe AS**:   Exertional dyspnea and chest pressure are hallmark symptoms of severe aortic stenosis.  **TTE findings** confirm severe calcific AS (mean gradient 45 mm Hg, AVA 0.85 cm²), which is definitive for diagnosis.   1. **Findings Against Other Diagnoses**:   **Acute Coronary Ischemia**: Troponin is only mildly elevated (common in ESRD), and ECG shows no ischemic changes.  **ADHF**: No pulmonary congestion on CXR, and BNP is only moderately elevated (common in ESRD).  **Volume Overload**: While mild pleural effusions are present, they are not the primary driver of symptoms (dyspnea/chest pressure are classic for AS).  **Conclusion**: Symptomatic **severe aortic stenosis** is the immediate life-threatening diagnosis requiring urgent intervention (aortic valve replacement). Other findings (anemia, volume overload) are comorbidities but do not explain the acute symptoms. |
| Mang | **Answer: Refer to cardiology to evaluate for surgery Furosemide 20 mg orally twice a day**  **Rationale:**   1. **Refer to cardiology to evaluate for surgery:**   **Symptomatic severe aortic stenosis (AS) requires urgent intervention (surgical aortic valve replacement SAVR*SAVR* or transcatheter aortic valve replacement TAVR*TAVR*).**  **Mortality is high if untreated once symptoms develop.**  **Cardiology evaluation is critical to determine candidacy for TAVR (preferred in ESRD patients due to lower perioperative risk) or SAVR.**   1. **Furosemide 20 mg orally twice a day:**   **Mild costophrenic angle blunting on CXR suggests volume overload, likely secondary to ESRD.**  **Low-dose furosemide can alleviate symptoms of congestion while avoiding excessive preload reduction.**  **Excluded Options:**   - **Admit for emergency surgery: Not indicated unless acute decompensation (e.g., cardiogenic shock, pulmonary edema) is present.** - **Follow up in 6 months: Inappropriate for symptomatic severe AS, which demands prompt intervention.** - **Stress testing (exercise/pharmacologic): Contraindicated in symptomatic AS due to risk of hemodynamic collapse.** - **Metoprolol succinate: Avoid in severe AS unless compelling indication (e.g., atrial fibrillation with rapid ventricular response). Beta-blockers can worsen LV outflow obstruction.** - **Nitroglycerin: Risky in AS due to preload reduction, which may precipitate hypotension.**   **Conclusion: Immediate cardiology referral for valve replacement evaluation is mandatory. Furosemide addresses mild volume overload but is adjunctive. Other options are either unsafe or delay definitive care.** | **Most Appropriate Management Options:**   1. **Refer to cardiology to evaluate for surgery**   **Rationale:** Severe symptomatic aortic stenosis (exertional dyspnea, chest pressure) requires **aortic valve replacement** (surgical AVR or TAVR). Cardiology evaluation is critical to determine candidacy and timing for intervention.   1. **Pharmacologic stress test** (if coronary artery disease CAD*CAD* is suspected)   **Rationale:** Coronary angiography is typically performed preoperatively in AS patients to assess for CAD. If angiography is not immediately available, a **pharmacologic stress test** (e.g., regadenoson or dobutamine) may be used to evaluate for ischemia, as exercise stress testing is contraindicated in symptomatic severe AS.  **Avoid/Inappropriate Options:**   - **Admit for emergency aortic valve surgery:** Surgery for AS is rarely "emergency" unless the patient is in cardiogenic shock (not described here). Elective evaluation is standard. - **Follow up in 6 months for repeat echocardiography:** Symptomatic severe AS requires **urgent intervention**, not delayed monitoring. - **Furosemide:** Ineffective in ESRD (patient is on dialysis) and may worsen renal function. Volume management should be addressed via dialysis. - **Metoprolol or nitroglycerin:** Contraindicated in severe AS. Beta-blockers can reduce cardiac output, and nitroglycerin may cause hypotension (reduces preload in a fixed-output state).   **Key Takeaway:** The priority is **definitive treatment of severe AS** (surgery/TAVR evaluation), not temporizing measures. | **Most Appropriate Management Options**:   1. **Refer to cardiology to evaluate for surgery** 2. **Furosemide 20 mg orally twice a day**   **Rationale**:   1. **Refer to cardiology for surgical evaluation**:   Symptomatic severe aortic stenosis (e.g., exertional dyspnea, chest pain) is a **Class I indication for aortic valve replacement** (surgical or transcatheter).  Cardiology evaluation is critical to determine the urgency, feasibility, and type of intervention (SAVR or TAVR).   1. **Furosemide for volume overload**:   Mild pleural effusions on CXR suggest fluid retention.  **Cautious diuresis** with furosemide can alleviate symptoms of volume overload while awaiting definitive intervention.  **Why Other Options Are Less Appropriate**:   - **Emergency aortic valve surgery**: While urgent intervention is required, "emergency" surgery typically implies immediate life-threatening instability (e.g., cardiogenic shock), which is not described here. - **Stress testing**: Contraindicated in symptomatic severe AS due to risk of hemodynamic collapse. - **Metoprolol/nitroglycerin**: Avoid in AS—beta-blockers can worsen outflow obstruction, and nitroglycerin may cause preload reduction and hypotension. - **6-month follow-up**: Inappropriate for symptomatic severe AS, as delayed intervention increases mortality risk.   **Conclusion**: Immediate referral to cardiology for surgical planning and cautious diuresis with furosemide are key. Definitive valve replacement should be prioritized. |
| Case10 |  |  |  |
| Diff | **Final Answer:**   - **Anemia** - **Anxiety or panic attack** - **Atrial fibrillation** - **Atrial flutter** - **Hypoglycemia** - **Medication or substance use** - **Nonsustained ventricular tachycardia (NSVT)** - **Premature atrial complexes (PACs)** - **Premature ventricular complexes (PVCs)**   **Explanation:**   1. **Anemia: Symptoms like palpitations, dizziness, and fatigue overlap with arrhythmias. A hemoglobin level is required to exclude it.** 2. **Anxiety/panic attack: Mimics arrhythmias with palpitations, light-headedness, and tachycardia. Psychological evaluation or exclusion of organic causes is needed.** 3. **Atrial fibrillation/flutter: Both cause irregular or rapid heart rates. Without ECG, they cannot be distinguished clinically.** 4. **Hypoglycemia: Symptoms (e.g., dizziness, palpitations) require blood glucose testing to rule out.** 5. **Medication/substance use: Stimulants (e.g., caffeine, decongestants) or drugs (e.g., cocaine) can induce tachycardia. History of use is necessary to exclude.** 6. **NSVT: Episodic, self-terminating VT may not be captured without prolonged monitoring (e.g., Holter).** 7. **PACs/PVCs: Frequent premature beats can mimic sustained arrhythmias clinically but require ECG for confirmation.**   **Excluded:**   - **Sustained ventricular arrhythmias: Typically cause hemodynamic instability (e.g., hypotension, syncope), which is absent in this stable patient (BP 140/80 mmHg).** - **AVNRT: A regular tachycardia, whereas atrial fibrillation is irregular. If physical exam confirms an irregularly irregular pulse, AVNRT is less likely.**   **Conclusion: The listed conditions cannot be excluded based on history and physical alone, as they share overlapping symptoms (palpitations, dizziness) and require further testing (ECG, labs, drug screening). Sustained ventricular arrhythmias and AVNRT are less likely in this context.** | **Differential Diagnoses That Cannot Be Excluded:**   1. **Anemia**   No CBC results are provided; anemia can cause palpitations even without overt symptoms.   1. **Anxiety or panic attack**   Subjective symptoms (e.g., intermittent palpitations) may align with anxiety, despite no explicit psychiatric history.   1. **Atrial fibrillation**   Paroxysmal AF can present with episodic palpitations. A normal exam during sinus rhythm does not exclude intermittent AF.   1. **Atrial flutter**   Similar to AF, atrial flutter can occur in paroxysmal episodes and would require ECG monitoring for confirmation.   1. **Atrioventricular nodal reentrant tachycardia (AVNRT)**   Classic SVT presentation (sudden-onset, self-terminating palpitations) fits the history.   1. **Hypoglycemia**   Diabetes on metformin increases risk; asymptomatic hypoglycemia could trigger palpitations. Blood glucose levels are not documented.   1. **Medication or substance use**   Caffeine (one cup daily) or other unmentioned substances (e.g., OTC medications) could contribute to symptoms.   1. **Nonsustained ventricular tachycardia (NSVT)**   Rare but possible in patients with hypertension/diabetes, even without structural heart disease. No ECG monitoring data to exclude.   1. **Premature atrial complexes (PACs)**   Common cause of palpitations; physical exam (regular rhythm at rest) does not rule out intermittent PACs.   1. **Premature ventricular complexes (PVCs)**   Similar to PACs; frequent PVCs can cause palpitations without exam findings.  **Excluded Diagnosis:**   - **Sustained ventricular arrhythmias**: Unlikely given absence of syncope, structural heart disease, family history of sudden death, and normal cardiovascular exam.   **Key Rationale:**   - The history and physical exam lack evidence of structural heart disease, hemodynamic instability, or "red flags" (e.g., syncope) that would suggest sustained ventricular arrhythmias. - All other diagnoses require further testing (e.g., ECG monitoring, labs) for exclusion.   **Next Steps:**   - **Ambulatory ECG monitoring** (Holter/event monitor) to capture rhythm during symptoms. - **Lab tests**: CBC, TSH, electrolytes, glucose/HbA1c. - **Echocardiogram** if arrhythmia is confirmed or structural disease is suspected. | **Differential Diagnoses That Cannot Be Excluded**:   1. **Anemia** 2. **Anxiety or panic attack** 3. **Atrial fibrillation** 4. **Atrial flutter** 5. **Atrioventricular nodal reentrant tachycardia (AVNRT)** 6. **Hypoglycemia** 7. **Medication or substance use** (e.g., caffeine) 8. **Nonsustained ventricular tachycardia (NSVT)** 9. **Premature atrial complexes (PACs)** 10. **Premature ventricular complexes (PVCs)**   **Rationale**:   - **Anemia**: No lab results (e.g., hemoglobin) to rule it out; palpitations can occur even without overt fatigue or pallor. - **Anxiety/panic attack**: Episodic palpitations without clear triggers; psychiatric history not fully explored. - **Atrial fibrillation/flutter**: Paroxysmal episodes may not be captured on a single EKG; rhythm may have normalized by the time of exam. - **AVNRT**: Classic for sudden-onset, self-terminating palpitations with a regular "racing" sensation; requires ambulatory monitoring for confirmation. - **Hypoglycemia**: Patient has diabetes on metformin; asymptomatic hypoglycemia cannot be excluded without glucose monitoring during symptoms. - **Medication/substance use**: Caffeine (one cup/day) could contribute; other OTC medications or dietary factors not explicitly ruled out. - **NSVT**: Asymptomatic short runs of VT may occur; requires monitoring to exclude. - **PACs/PVCs**: Frequent ectopy can mimic prolonged palpitations; rhythm monitoring needed.   **Excluded Diagnoses**:   - **Sustained ventricular arrhythmias**: Unlikely given absence of syncope, structural heart disease, or hemodynamic instability.   **Key Next Steps**:   - **Ambulatory cardiac monitoring** (e.g., Holter, event recorder) to capture rhythm during symptoms. - **Labs**: CBC (for anemia), glucose/HbA1c (hypoglycemia/diabetes control), TSH, electrolytes. - **12-lead EKG** (if not already done) to assess for baseline abnormalities.   **Conclusion**: While **AVNRT** or **PACs/PVCs** are most likely, multiple arrhythmic, metabolic, and psychological etiologies remain possible until further testing is performed. |
| Diag | **Based on the clinical scenario, the following steps are essential to perform immediately:**   1. **ECG**   **Rationale: A 12-lead ECG should be done immediately, even if the patient is asymptomatic at the time. It can detect acute ischemia, arrhythmias (e.g., atrial fibrillation/flutter, AVNRT), or structural abnormalities (e.g., Wolff-Parkinson-White syndrome, QT prolongation).**   1. **24-hour Holter monitor**   **Rationale: Essential to capture heart rhythm during symptoms if episodes occur daily or frequently. This is critical to confirm or rule out atrial fibrillation, atrial flutter, AVNRT, or frequent PACs/PVCs.**   1. **Event monitor**   **Rationale: If symptoms are sporadic (e.g., weekly), an event monitor (wearable or implantable) is necessary to record rhythm during episodes. This complements the Holter monitor for less frequent symptoms.**  **Not immediately required:**   - **Cardiac enzymes (e.g., troponin):**   **Only indicated if there is concern for myocardial ischemia (e.g., chest pain, ST changes on ECG). No evidence of acute coronary syndrome here.**   - **Stress test:**   **Not urgent unless exercise-induced arrhythmia is suspected (patient did not report exertion-triggered symptoms).**   - **Admit to a monitored hospital bed:**   **Not necessary unless the patient has hemodynamic instability, syncope, or high-risk findings (e.g., sustained ventricular tachycardia).**  **Final Answer:**   - **ECG** - **24-h Holter monitor** - **Event monitor** | **Essential Immediate Steps:**   1. **ECG**   **Rationale:** A 12-lead ECG is critical to assess for acute arrhythmias (e.g., atrial fibrillation, atrial flutter), conduction abnormalities (e.g., Wolff-Parkinson-White syndrome), or ischemic changes. Even if the patient is asymptomatic at the time, it provides a baseline and may reveal clues (e.g., short PR interval in AVNRT).   1. **Event Monitor**   **Rationale:** Given the intermittent nature of symptoms (weekly episodes lasting minutes to hours), an **event monitor** is more likely to capture the rhythm during an episode than a 24-hour Holter monitor. Event monitors can be worn for weeks and activated by the patient when symptoms occur, making them ideal for infrequent palpitations.  **Not Immediately Essential:**   - **24-hour Holter Monitor**: Less likely to capture episodes occurring weekly but may still be considered if symptoms are suspected to occur daily. - **Cardiac Enzymes**: Unnecessary without chest pain, hemodynamic instability, or ECG evidence of ischemia. - **Stress Test**: Not urgent; patient has no exertional symptoms (walks 3 miles without issues). - **Admit to Monitored Bed**: Not indicated for stable, self-resolving palpitations without syncope, chest pain, or high-risk features.   **Key Takeaway:**   - **ECG** is mandatory for all patients with palpitations. - **Event monitor** is the preferred ambulatory monitoring tool for intermittent symptoms. These two steps are essential to initiate immediately in the outpatient setting. | **Essential Immediate Steps**:   1. **ECG** 2. **Event monitor**   **Rationale**:   - **ECG**: Critical to perform immediately to assess for acute arrhythmia (e.g., atrial fibrillation/flutter, ventricular tachycardia), ischemia, or conduction abnormalities (e.g., Wolff-Parkinson-White). Even if asymptomatic at the time, it provides a baseline and may reveal clues (e.g., short PR interval in AVNRT). - **Event monitor**: Preferred over a 24-hour Holter monitor due to the **intermittent nature** of her symptoms (once or twice weekly). An event monitor can be worn for days to weeks and activated during symptoms, increasing the likelihood of capturing the rhythm during an episode.   **Not Essential Immediately**:   - **24-hour Holter monitor**: Less likely to capture infrequent episodes (weekly). Reserve if symptoms occur daily. - **Cardiac enzymes**: No signs of acute coronary syndrome (e.g., chest pain, hemodynamic instability). - **Stress test**: Patient has **no exertional symptoms** (walks 3 miles without issues); not indicated without clinical suspicion of ischemia. - **Admit to monitored bed**: Patient is stable, with no red flags (e.g., syncope, hypotension, sustained arrhythmia).   **Conclusion**: **ECG** and **event monitor** are the highest-priority steps to identify the underlying rhythm during symptoms and rule out dangerous arrhythmias. Other tests (e.g., labs for anemia/hypoglycemia) should follow but are not urgent if the patient is stable. |
| Dx | **Final Answer:**   - **Atrial fibrillation**   **Explanation:**   1. **ECG confirmation: The patient’s ECG explicitly confirmed persistent atrial fibrillation, which is diagnostic. Atrial fibrillation is the only arrhythmia directly supported by the ECG findings.** 2. **Clinical correlation: Symptoms (palpitations, light-headedness) and an irregularly irregular heart rate (138 beats/min) align with atrial fibrillation.** 3. **Exclusion of alternatives:**   **Atrial flutter: Typically shows a regular rhythm or "sawtooth" atrial activity on ECG, which is absent here.**  **AVNRT/Junctional tachycardia: These cause regular tachycardias, inconsistent with the irregular rhythm described.**  **MAT: Requires ≥3 distinct P-wave morphologies on ECG, not observed here.**  **PACs: Isolated premature beats do not explain sustained tachycardia or ECG-confirmed atrial fibrillation.**  **Conclusion: The ECG confirmation of atrial fibrillation, combined with symptoms and physical findings, makes it the definitive diagnosis. Other arrhythmias are ruled out by ECG or clinical features.** | **Answer:** **Atrial fibrillation**  **Rationale:**   - The ECG in the emergency department confirms **persistent atrial fibrillation** (irregularly irregular rhythm, absence of P waves, rapid ventricular rate of 138 bpm). - Symptoms (palpitations, light-headedness, dyspnea) and clinical findings align with atrial fibrillation. - Atrial flutter, AVNRT, junctional tachycardia, and MAT would typically present with a regular ventricular rhythm or specific ECG patterns (e.g., flutter waves, retrograde P waves), which are absent here. - PACs do not cause sustained tachycardia or hemodynamic symptoms.   This diagnosis is definitive based on the ED evaluation. | **Answer**: **Atrial fibrillation**  **Rationale**:   - The ECG in the emergency department **confirms persistent atrial fibrillation** (irregularly irregular rhythm, absence of P waves, rapid ventricular rate). - Key clinical features supporting this diagnosis:   **Irregular heart rate** (138 bpm).  Symptoms of **palpitations, light-headedness, and fatigue** (common in AF due to rapid ventricular response).   - - **ECG confirmation** (definitive for AF).   **Why Other Options Are Less Likely**:   - **Atrial flutter**: Typically has a regular atrial rate (sawtooth "flutter waves") and a regular ventricular rate unless variable conduction occurs. ECG would show characteristic flutter waves, not AF. - **AVNRT**: Presents with a **regular** narrow-complex tachycardia (usually 150-250 bpm), not an irregular rhythm. - **Junctional tachycardia**: Regular rhythm with rates ≤130 bpm; no association with irregularity. - **Multifocal atrial tachycardia (MAT)**: Irregular rhythm but requires ≥3 distinct P-wave morphologies on ECG; typically seen in critically ill patients (e.g., COPD exacerbation). - **PACs**: Isolated premature beats, not sustained tachycardia.   **Conclusion**: The **ECG-confirmed atrial fibrillation** aligns with the patient’s symptoms (palitations, light-headedness) and irregular heart rate. No other diagnosis fits the clinical and electrocardiographic findings. |
| Mang1 | **Final Answer:**   - **Instruct the patient to go to the emergency department right away.** - **Instruct the patient to perform a Valsalva maneuver (such as bearing down hard as if she is having a bowel movement).**   **Explanation:**   1. **Emergency Department Referral:**   **The patient has symptomatic atrial fibrillation with a rapid ventricular rate (138 bpm). Even though her blood pressure is stable, urgent evaluation is required to assess for hemodynamic compromise, initiate rate or rhythm control, evaluate for thromboembolic risk (e.g., need for anticoagulation), and rule out underlying causes (e.g., infection, electrolyte abnormalities). Delaying care could increase risks of complications (e.g., stroke, heart failure).**   1. **Valsalva Maneuver:**   **While vagal maneuvers (e.g., Valsalva) are less effective for atrial fibrillation compared to reentrant SVTs (e.g., AVNRT), they may transiently slow the ventricular rate by increasing vagal tone. This can provide temporary symptom relief while awaiting emergency care.**  **Excluded Options:**   - **Double lisinopril dosage: Unnecessary and dangerous. Lisinopril is not indicated for acute rate/rhythm control in AF. Abrupt dose increases risk hypotension without addressing the arrhythmia.** - **Aspirin 325 mg and clinic next day: Aspirin alone is inadequate for stroke prevention in AF (requires risk-stratified anticoagulation). Delaying evaluation by a day is unsafe given her symptomatic, rapid AF.**   **Conclusion: Immediate ED referral ensures timely management of AF complications. Valsalva may offer transient symptom relief but does not replace urgent evaluation. Other interventions are inappropriate or ineffective.** | **Answer:** **Instruct the patient to go to the emergency department right away.**  **Rationale:**   - **Urgency of Symptoms**: The patient reports **worsening symptoms** (palpitations, light-headedness, dyspnea at rest) and has a newly identified arrhythmia (likely atrial fibrillation). Rapid ventricular rates in AF can lead to hemodynamic instability or complications (e.g., heart failure, stroke). Immediate ED evaluation is critical to assess for instability, initiate rate control, and rule out ischemia or thromboembolism. - **Risks of Other Options**:   **Doubling lisinopril**: Unnecessary and dangerous. ACE inhibitors do not acutely manage AF, and doubling the dose risks hypotension or kidney injury.  **Valsalva maneuver**: Effective for terminating SVT (e.g., AVNRT) but ineffective in atrial fibrillation.  **Aspirin and next-day clinic**: Aspirin is not a substitute for anticoagulation in AF, and delaying care risks deterioration (e.g., stroke, decompensation).  **Key Takeaway:** The **only essential immediate action** is directing the patient to the ED for urgent evaluation and management of symptomatic, persistent atrial fibrillation. | **Essential Immediate Steps:**   1. **Instruct the patient to go to the emergency department right away.**   **Rationale**:  Atrial fibrillation (AF) with a **rapid ventricular rate (138 bpm)** and symptoms (light-headedness, palpitations) requires urgent evaluation.  The emergency department can assess for hemodynamic instability, initiate rate/rhythm control (e.g., IV beta-blockers, calcium channel blockers), and evaluate for complications (e.g., stroke, heart failure).  **Why Other Options Are Not Appropriate:** 2. **Double up on lisinopril dosage**:   - **Risk**: Lisinopril is an ACE inhibitor used for hypertension/heart failure, not AF. Doubling the dose could cause hypotension or kidney injury without addressing the arrhythmia.  1. **Perform a Valsalva maneuver**:   **Ineffective**: Vagal maneuvers (e.g., Valsalva) are used for **supraventricular tachycardias (e.g., AVNRT)**, not AF. AF is not responsive to these maneuvers.   1. **Take aspirin 325 mg and come to the clinic the next day**:   **Delayed Care**: Aspirin is not a first-line therapy for stroke prevention in AF (anticoagulants are preferred). Delaying evaluation risks deterioration (e.g., worsening heart failure, stroke).  **Key Considerations for AF Management:**   - **Urgent rate control** is critical to reduce symptoms and myocardial strain. - **Stroke risk assessment** (e.g., CHA₂DS₂-VASc score) and anticoagulation (if indicated) should follow stabilization.   **Conclusion**: The patient requires **immediate emergency care** due to symptomatic, rapid AF. Other interventions are either ineffective or unsafe. |
| Mang2 | **Final Answer:**   - **Diltiazem 15 mg by IV push followed by diltiazem drip at 5 mg/h** - **Heparin 60 U/kg IV followed by 12 mg/kg/h drip** - **CBC** - **Complete metabolic panel** - **Serum PT/PTT** - **Serum thyroid-stimulating hormone (TSH) level** - **Transthoracic echocardiography (TTE)**   **Explanation:**   1. **Rate Control:**   **Diltiazem is a first-line agent for acute rate control in atrial fibrillation (AF) with rapid ventricular response. It reduces heart rate by blocking calcium channels, improving symptoms and cardiac output. The IV push followed by a drip ensures sustained effect.**   1. **Anticoagulation:**   **Heparin is critical to prevent thromboembolism, especially if AF has persisted >48 hours (likely here, as the patient delayed care for 3 days). Anticoagulation must precede any cardioversion to reduce stroke risk.**   1. **Essential Labs:**   **CBC: Evaluates for anemia, infection, or other contributors to AF.**  **Complete metabolic panel: Assesses electrolytes (e.g., potassium, magnesium) and renal function (key for drug dosing, including diltiazem and heparin).**  **PT/PTT: Monitors coagulation status before initiating heparin.**  **TSH: Thyroid dysfunction (hyperthyroidism) is a reversible cause of AF.**   1. **Imaging:**   **TTE: Evaluates cardiac structure (e.g., ejection fraction, valvular disease) to guide long-term management.**  **Excluded Options:**   - **Aspirin: Inadequate for stroke prevention in AF; anticoagulation (heparin) is required.** - **Adenosine: Used for terminating SVT (e.g., AVNRT), not effective in AF.** - **Digoxin: Less effective for acute rate control in this context; reserved for patients with heart failure.** - **Immediate cardioversion: Not indicated in stable patients with AF >48 hours without prior anticoagulation.** - **TEE: May be needed later if cardioversion is planned, but TTE is prioritized first.** - **Cardiac catheterization: Unnecessary unless acute coronary ischemia is suspected (no evidence here).**   **Conclusion: Immediate priorities are rate control (diltiazem), anticoagulation (heparin), labs to identify reversible causes and guide therapy, and TTE for structural evaluation. Other interventions are either ineffective, inappropriate, or premature.** | **Essential Immediate Steps:**   1. **Diltiazem 15 mg IV push followed by diltiazem drip at 5 mg/h**   **Rationale**: The patient’s rapid ventricular rate (138 bpm) is contributing to symptoms (palpitations, light-headedness). Diltiazem provides rapid rate control, improving hemodynamics and symptoms.   1. **Heparin 60 U/kg IV followed by 12 U/kg/h drip**   **Rationale**: Anticoagulation is critical to prevent thromboembolism, especially given the unknown duration of atrial fibrillation (>48 hours in this case). Heparin bridges to long-term anticoagulation and reduces stroke risk, particularly if cardioversion is considered later.   1. **CBC, Complete metabolic panel, Serum PT/PTT**   **CBC**: Assess for anemia or infection.  **Metabolic panel**: Evaluate electrolytes (e.g., potassium, magnesium) and renal function (creatinine) to guide medication dosing (e.g., diltiazem, heparin).  **PT/PTT**: Baseline coagulation studies to monitor heparin therapy.  **Why Other Options Are Not Essential Immediately:**   - **Adenosine**: Ineffective in atrial fibrillation (AFib) as it does not terminate or slow AFib. - **Digoxin**: Slower onset; less effective for acute rate control in this setting. - **Aspirin**: Not sufficient for stroke prevention in AFib; anticoagulation (heparin) is preferred. - **Immediate synchronized cardioversion**: Reserved for unstable patients (e.g., hypotension, heart failure). This patient is hemodynamically stable (BP 140/80). - **TEE/TTE**: TEE is needed only if urgent cardioversion is planned (to exclude atrial thrombus). TTE is important for long-term management but not an emergency. - **TSH**: Thyroid dysfunction can trigger AFib, but testing does not change acute management. - **Cardiac catheterization**: No evidence of acute coronary syndrome.   **Key Takeaway**: Prioritize **rate control (diltiazem)**, **anticoagulation (heparin)**, and **baseline labs** to guide therapy. Avoid interventions unrelated to acute stabilization (e.g., adenosine, digoxin, aspirin). | **Essential Immediate Steps:**   1. **Diltiazem 15 mg IV push followed by diltiazem drip at 5 mg/h**   **Rationale**: Rapid ventricular rate (138 bpm) in atrial fibrillation (AF) requires **rate control**. Diltiazem (a non-dihydropyridine calcium channel blocker) is first-line for acute rate control in stable patients.   1. **Heparin 60 U/kg IV followed by 12 mg/kg/h drip**   **Rationale**: Anticoagulation is critical to prevent thromboembolism (e.g., stroke), especially if AF has persisted >48 hours (likely here, given "persistent AF" and delayed presentation). Heparin bridges to long-term anticoagulation while assessing stroke risk.   1. **CBC, Complete metabolic panel (CMP), Serum PT/PTT, Serum TSH**   **CBC**: Rule out infection/anemia contributing to symptoms.  **CMP**: Assess electrolytes (e.g., potassium, magnesium) and renal function (for medication dosing).  **PT/PTT**: Baseline coagulation status before anticoagulation.  **TSH**: Hyperthyroidism is a reversible cause of AF.   1. **Transthoracic echocardiography (TTE)**   **Rationale**: Evaluate cardiac structure (e.g., atrial size, ejection fraction) and rule out structural heart disease (e.g., valvular AF).  **Why Other Options Are Less Urgent or Inappropriate:**   - **Adenosine**: Used for terminating **SVT**, not effective in AF. - **Digoxin**: Slower onset; reserved for patients with heart failure or as adjunctive therapy. - **Aspirin**: Insufficient for stroke prevention in AF (anticoagulants are required). - **Immediate synchronized cardioversion**: Reserved for **unstable** patients (e.g., hypotension, shock). This patient is hemodynamically stable (BP 140/80). - **Transesophageal echocardiography (TEE)**: Needed only if **urgent cardioversion** is planned and AF duration is >48 hours or unknown. Requires anticoagulation first. - **Cardiac catheterization**: Unnecessary unless ACS is suspected (no evidence here).   **Additional Considerations:**   - **Anticoagulation strategy**: Transition to oral anticoagulation (e.g., DOAC, warfarin) after heparin, guided by CHA₂DS₂-VASc score. - **Rhythm control**: If rate control fails or symptoms persist, consider cardioversion **after ≥3 weeks of therapeutic anticoagulation** or a TEE to exclude atrial thrombus.   **Conclusion**: Prioritize **rate control (diltiazem)**, **anticoagulation (heparin)**, **essential labs**, and **TTE** for immediate management. Avoid interventions not aligned with AF pathophysiology or the patient’s stability. |
| Case11 |  |  |  |
| Diff | **Differential Diagnoses That Cannot Be Excluded: Based on the history and physical examination, the following conditions remain plausible and require further investigation:**   1. **Myasthenia Gravis**   **Rationale: Fluctuating symptoms (worse later in the day), hypernasal speech (bulbar weakness), nasal regurgitation (palatal weakness), and minimal left ptosis are classic features. Myasthenia gravis often presents with fatigable weakness affecting ocular and bulbar muscles.**   1. **Eaton-Lambert Syndrome**   **Rationale: While typically associated with proximal muscle weakness and autonomic dysfunction, Eaton-Lambert can occasionally present with bulbar symptoms like ptosis and dysphagia. The absence of weakness on exam does not fully exclude it, as early stages may lack obvious motor deficits.**   1. **Oculopharyngeal Muscular Dystrophy (OPMD)**   **Rationale: Characterized by ptosis and pharyngeal weakness, which could explain nasal regurgitation (a form of dysphagia for liquids). However, the patient denies overt dysphagia, and OPMD usually manifests later in life with progressive symptoms. While less likely, it cannot be ruled out without genetic testing.**  **Differentials That Can Be Excluded:**   - **Adverse Effect of Inhaled Corticosteroids: Hypernasal voice is not a recognized side effect of beclomethasone.** - **ALS: No evidence of upper/lower motor neuron signs, weakness, or atrophy.** - **Botulism: Symptoms are subacute/chronic, not acute, and there is no exposure history.** - **Chronic Rhinosinusitis: No nasal congestion, discharge, or polyps post-surgery; sinuses are patent.** - **Myotonic Muscular Dystrophy: Absence of myotonia, muscle stiffness, or systemic features (e.g., cardiac issues).** - **Mitochondrial Myopathy: No lactic acidosis, seizures, or exercise intolerance.** - **Sinus Cancer: No nasal obstruction, lesions, or constitutional symptoms (e.g., weight loss).**   **Conclusion: Myasthenia gravis is the most likely diagnosis, but Eaton-Lambert syndrome and oculopharyngeal muscular dystrophy cannot be excluded without further testing (e.g., antibody studies, repetitive nerve stimulation, or genetic testing). Immediate evaluation should prioritize myasthenia gravis due to its higher pretest probability and treatable nature.** | **Differential Diagnoses That Cannot Be Excluded:**   1. **Myasthenia Gravis**   **Rationale**: Fatigable symptoms (worsening voice later in the day), minimal left ptosis on sustained gaze, and nasal regurgitation align with neuromuscular junction dysfunction. Myasthenia gravis is a top consideration.   1. **Mitochondrial Myopathy**   **Rationale**: Mitochondrial disorders can present with ptosis and mild neuromuscular symptoms. Though systemic features are absent, it cannot be definitively ruled out without further testing (e.g., muscle biopsy, genetic testing).   1. **Oculopharyngeal Muscular Dystrophy (OPMD)**   **Rationale**: Ptosis and dysphagia (nasal regurgitation may mimic pharyngeal weakness) are features of OPMD. However, the absence of significant dysphagia or family history makes this less likely but still possible.   1. **Adverse Effect of Inhaled Corticosteroids**   **Rationale**: While inhaled steroids typically cause hoarseness or thrush, chronic use could theoretically contribute to mucosal changes or subtle neuromuscular effects. The temporal relationship to surgery makes this less likely, but not excluded.   1. **Chronic Rhinosinusitis**   **Rationale**: Despite patent sinuses on exam, structural or functional residual effects from prior sinusitis or surgery (e.g., scarring, impaired mucociliary clearance) could contribute to hypernasal speech.  **Diagnoses That Can Likely Be Excluded:**   - **ALS**: No upper/lower motor neuron signs (normal strength, reflexes, no atrophy). - **Botulism**: Acute presentation with descending paralysis is absent; symptoms are chronic. - **Eaton-Lambert Syndrome**: No proximal weakness, autonomic dysfunction, or malignancy history. - **Myotonic Muscular Dystrophy**: No myotonia, distal weakness, or systemic features. - **Sinus Cancer**: No mass lesions, nasal obstruction, or constitutional symptoms.   **Key Takeaway**: **Myasthenia gravis** is the most compelling diagnosis given fatigable ptosis and hypernasality. However, **mitochondrial myopathy**, **OPMD**, **chronic rhinosinusitis**, and **steroid effects** require further evaluation (e.g., antibody testing, nasoendoscopy, or imaging). Other conditions (ALS, botulism) are improbable based on history and exam. | **Differential Diagnoses That Cannot Be Excluded:**   1. **Myasthenia Gravis**   **Rationale**: Fatigable ptosis (worse with sustained gaze) and hypernasal speech/nasal regurgitation (bulbar weakness) are classic features. Requires anti-AChR antibody testing or EMG to exclude.   1. **Amyotrophic Lateral Sclerosis (ALS)**   **Rationale**: Bulbar-onset ALS can present with dysarthria and swallowing issues. While she lacks limb weakness or upper motor neuron signs, early ALS cannot be excluded without electromyography (EMG).   1. **Eaton-Lambert Syndrome**   **Rationale**: Rare but can cause ptosis and proximal muscle weakness. Her lack of limb weakness makes it less likely, but presynaptic neuromuscular junction disorders require exclusion with repetitive nerve stimulation.   1. **Mitochondrial Myopathy**   **Rationale**: Mitochondrial disorders (e.g., chronic progressive external ophthalmoplegia) can present with ptosis and fatigue. No systemic symptoms reported, but genetic testing or muscle biopsy may be needed.   1. **Oculopharyngeal Muscular Dystrophy (OPMD)**   **Rationale**: Autosomal dominant disorder with late-onset ptosis, dysphagia, and proximal weakness. Nasal regurgitation and ptosis align with this diagnosis, though family history is unclear.   1. **Myotonic Muscular Dystrophy**   **Rationale**: Can present with ptosis and facial weakness. Absence of myotonia (e.g., delayed handgrip release) on exam makes it less likely, but genetic testing is required for exclusion.  **Diagnoses That Can Likely Be Excluded:**   - **Botulism**: Acute onset (hours/days), descending paralysis, and autonomic dysfunction (e.g., dry mouth) are absent. - **Chronic Rhinosinusitis**: No active nasal congestion/discharge, and symptoms are unrelated to sinus inflammation. - **Sinus Cancer**: Normal nasal exam (no masses/lesions) and patent sinuses make this unlikely. - **Adverse Effect of Inhaled Corticosteroids**: Hoarseness or thrush is typical, not hypernasality or ptosis.   **Key Considerations:**   - **Myasthenia Gravis** is the **most likely diagnosis** given fatigable ptosis and bulbar symptoms. - **Structural VPI** from prior surgery remains a possible contributor to hypernasal speech, but ptosis and symptom progression suggest a neuromuscular overlap. - **Imaging (MRI)** and **specialized testing** (antibodies, EMG) are critical next steps to differentiate between these etiologies.   **Conclusion**: Myasthenia gravis, ALS, Eaton-Lambert syndrome, mitochondrial myopathy, oculopharyngeal muscular dystrophy, and myotonic dystrophy cannot be excluded based on current finding |
| Diag | **Appropriate Next Steps for Diagnosis and Treatment: Based on the clinical suspicion for myasthenia gravis and related neuromuscular disorders, the following steps are most appropriate:**   1. **Edrophonium Test**   **Rationale: A positive response (improvement in ptosis or speech) strongly supports myasthenia gravis. This is a quick bedside test but requires cardiac monitoring due to potential side effects.**   1. **EMG with Repetitive Nerve Testing**   **Rationale:**  **In myasthenia gravis, repetitive stimulation at 3–5 Hz shows a decremental response (reduced muscle action potentials).**  **In Eaton-Lambert syndrome, high-frequency stimulation (20–50 Hz) demonstrates an incremental response.**   1. **Serum Acetylcholine Receptor (AChR) Antibody**   **Rationale: Detects antibodies against AChR in 80–90% of generalized myasthenia cases. If negative, anti-MuSK or anti-LRP4 antibodies should be tested.**  **Steps That Are Less Relevant or Unnecessary:**   - **Carotid Doppler Ultrasonography: No evidence of vascular pathology (e.g., stroke, TIA) to explain her symptoms.** - **EEG: No seizures or altered mental status.** - **Serum Creatine Kinase (CK): Normal strength and absence of myopathic features (e.g., muscle pain, weakness) make muscle injury less likely.** - **Muscle Biopsy: Reserved for suspected myopathies (e.g., mitochondrial myopathy, muscular dystrophy) if EMG/antibody testing is inconclusive.** - **MRI of the Brain: Not indicated unless focal neurologic deficits or central causes (e.g., brainstem lesion) are suspected.**   **Conclusion: The priority is to confirm or exclude myasthenia gravis with the edrophonium test, AChR antibodies, and EMG with repetitive nerve stimulation. If these are negative, further evaluation for Eaton-Lambert syndrome (e.g., voltage-gated calcium channel antibodies) or oculopharyngeal muscular dystrophy (genetic testing) may follow.** | **Most Appropriate Steps for Diagnosis and Treatment:**   1. **Serum Acetylcholine Receptor (AChR) Antibody**   **Rationale**: Critical for confirming **myasthenia gravis (MG)**, the leading diagnosis given fatigable ptosis and hypernasality.   1. **EMG with Repetitive Nerve Testing**   **Rationale**: Assesses for neuromuscular junction dysfunction (e.g., decremental response in MG). Supports MG diagnosis if antibodies are equivocal.   1. **Edrophonium Test**   **Rationale**: A positive response (temporary improvement in ptosis/voice) strongly supports MG. However, it is less commonly used today due to safety/availability concerns.   1. **Serum Creatine Kinase (CK)**   **Rationale**: Elevated CK suggests muscle damage (e.g., mitochondrial myopathy or muscular dystrophy), which are less likely but still on the differential.  **Steps Likely Not Indicated:**   - **Carotid Doppler Ultrasonography**: No evidence of stroke/TIA or carotid disease. - **EEG**: No seizures or altered mental status. - **Muscle Biopsy**: Reserved for confirmed suspicion of myopathy (e.g., if CK is elevated or EMG suggests myopathy). - **MRI of the Brain**: Low yield given the absence of focal neurological deficits, but may be considered if MG testing is negative.   **Key Takeaway**: The priority is to confirm or exclude **myasthenia gravis** with **AChR antibodies** and **EMG with repetitive nerve testing**. **Edrophonium test** and **CK** provide additional supportive data. Other tests (e.g., MRI, muscle biopsy) are secondary unless initial evaluations are inconclusive. | **Most Appropriate Steps for Diagnosis and Treatment:**   1. **Serum Acetylcholine Receptor (AChR) Antibody**   **Rationale**: First-line test for **myasthenia gravis** (MG). Positive antibodies confirm the diagnosis, which aligns with her ptosis, fatigable symptoms, and bulbar weakness (hypernasal speech, nasal regurgitation).   1. **EMG with Repetitive Nerve Testing**   **Rationale**: Differentiates between **MG** (postsynaptic neuromuscular junction defect) and **Eaton-Lambert syndrome** (presynaptic disorder). Also evaluates for **ALS** (though less likely given lack of upper motor neuron signs) and other neuromuscular pathologies.   1. **Edrophonium Test**   **Rationale**: If AChR antibodies are negative but clinical suspicion for MG remains high, this test can transiently improve ptosis or weakness, supporting the diagnosis. Less commonly used today due to safety concerns but still relevant in equivocal cases.   1. **MRI of the Brain**   **Rationale**: Excludes structural causes (e.g., brainstem lesion, cavernous sinus pathology) contributing to ptosis or bulbar dysfunction. Though less likely than neuromuscular causes, imaging is prudent given her post-surgical context and neurologic findings.  **Steps Not Indicated:**   - **Carotid Doppler Ultrasonography**: No evidence of stroke or carotid artery disease. - **EEG**: No seizure-like activity or altered mental status. - **Muscle Biopsy**: Reserved for suspected myopathies (e.g., mitochondrial myopathy) if initial testing is inconclusive. - **Serum Creatine Kinase**: Not suggestive of acute muscle injury or myositis; normal strength and lack of myopathic features make this low yield.   **Key Considerations:**   - **Myasthenia gravis** is the **top priority** given clinical features. - **EMG** and **AChR antibodies** are critical for confirming MG or identifying alternative neuromuscular disorders (e.g., Eaton-Lambert, ALS). - **MRI brain** provides a safety net to rule out structural lesions, though less urgent than neuromuscular testing.   **Next Steps**: Proceed with **AChR antibody testing** and **EMG** first. If negative, consider **MuSK antibody testing** (for seronegative MG) or **MRI brain**. The edrophonium test may be used cautiously if diagnosis remains unclear. |
| Dx | **The most likely diagnosis is: • Myasthenia Gravis**  **Key Supporting Evidence:**   1. **EMG Findings:**   **Decremental response at 3-Hz stimulation: Classic for impaired neuromuscular transmission (pathognomonic for myasthenia gravis).**  **Facilitation after brief exercise (partial improvement) and post-facilitation exhaustion (worsening decrement): Specific to myasthenia gravis.**   1. **Serum Acetylcholine Receptor (AChR) Antibody:**   **Markedly elevated titer (0.28 nmol/L): Confirms autoimmune myasthenia gravis.**   1. **Normal Creatine Kinase (CK):**   **Excludes muscle breakdown or myopathic processes (e.g., muscular dystrophy, mitochondrial myopathy).**  **Why Other Diagnoses Are Unlikely:**   - **Mitochondrial Myopathy:**   **EMG would show myopathic features (short-duration, low-amplitude motor units) or ragged red fibers on biopsy, not a decremental response.**  **Typically associated with elevated lactate, exercise intolerance, or multi-organ involvement (not seen here).**   - **Myotonic Muscular Dystrophy:**   **EMG would demonstrate myotonic discharges (characteristic "dive-bomber" sound).**  **Clinical features include myotonia (delayed muscle relaxation), distal weakness, and systemic manifestations (e.g., cataracts, cardiac arrhythmias).**   - **Oculopharyngeal Muscular Dystrophy:**   **Presents with progressive ptosis, dysphagia, and proximal limb weakness.**  **EMG would show myopathic changes (not decremental responses), and muscle biopsy would reveal rimmed vacuoles.**  **Caused by genetic mutations (e.g., *PABPN1*), not autoantibodies.**  **Conclusion: The combination of decremental EMG responses, post-facilitation exhaustion, and elevated AChR antibodies definitively confirms myasthenia gravis. No further testing is needed for diagnosis.** | **The most likely diagnosis is:** **• Myasthenia Gravis**  **Key Evidence Supporting Myasthenia Gravis:**   1. **EMG with Repetitive Nerve Testing**:   **Decremental response** (≥10% decline in muscle action potential amplitude with 3-Hz stimulation), which is **pathognomonic** for postsynaptic neuromuscular junction disorders like MG.  **Facilitation** (improvement after brief exercise) and **post-exercise exhaustion** (worsening after prolonged exercise) are classic features of MG.   1. **Serum Acetylcholine Receptor (AChR) Antibodies**:   **Markedly elevated** (0.28 nmol/L vs. normal <0.02 nmol/L), confirming autoimmune MG.   1. **Normal Creatine Kinase (CK)**:   Rules out primary muscle diseases (e.g., myopathies or muscular dystrophies), which typically show elevated CK.  **Why Other Diagnoses Are Unlikely:**   - **Mitochondrial Myopathy**:   EMG would show **myopathic changes** (e.g., small, polyphasic motor units), not a decremental response.  No association with elevated AChR antibodies.   - **Myotonic Muscular Dystrophy**:   EMG would demonstrate **myotonic discharges** (waxing/waning "dive bomber" sounds), not a decrement.  Clinical features like myotonia (delayed muscle relaxation) or systemic manifestations (e.g., cardiac arrhythmias, cataracts) are absent.   - **Oculopharyngeal Muscular Dystrophy (OPMD)**:   EMG would show **myopathic changes**, not a decremental response.  Typically genetic (PABPN1 mutation) and associated with slowly progressive ptosis/dysphagia, but not fatigable weakness or autoantibodies.  **Conclusion:** The combination of **decremental EMG response**, **elevated AChR antibodies**, and **normal CK** is diagnostic for **myasthenia gravis**. No further testing is required unless atypical features emerge. | **Most Likely Diagnosis**: **• Myasthenia Gravis**  **Key Supporting Evidence**:   1. **EMG Findings**:   **Decremental response** to 3-Hz stimulation (classic for postsynaptic neuromuscular junction dysfunction).  **Facilitation** (improvement after brief exercise) and **postfacilitation exhaustion** (worsening after prolonged exercise).  These findings are pathognomonic for **myasthenia gravis**.   1. **Serum Acetylcholine Receptor (AChR) Antibody**:   **Elevated titer (0.28 nmol/L; normal <0.02 nmol/L)** confirms autoimmune myasthenia gravis.   1. **Normal Creatine Kinase (CK)**:   Rules out muscle breakdown or myopathy (e.g., muscular dystrophy, mitochondrial myopathy).  **Why Other Diagnoses Are Unlikely**:   - **Mitochondrial Myopathy**:   EMG would show **myopathic changes** (e.g., short-duration, low-amplitude motor unit potentials), not a decremental response.  Typically presents with exercise intolerance, lactic acidosis, or multi-organ involvement (absent here).   - **Myotonic Muscular Dystrophy**:   EMG would show **myotonic discharges** (e.g., waxing-waning "dive bomber" sounds), not decremental responses.  Clinical features include **myotonia** (delayed muscle relaxation), distal weakness, and systemic manifestations (e.g., cataracts, cardiac arrhythmias).   - **Oculopharyngeal Muscular Dystrophy**:   EMG would show **myopathic changes**, not decremental responses.  Genetic disorder (autosomal dominant) with late-onset ptosis, dysphagia, and proximal limb weakness.  **Conclusion**: The combination of **AChR antibodies**, **decremental EMG responses**, and clinical features (ptosis, bulbar weakness) confirms **myasthenia gravis** as the diagnosis. |
| Mang | **The most appropriate steps for diagnosis and treatment are:**   1. **Pyridostigmine 60 mg po q 4-6 h:**   **First-line symptomatic therapy to improve neuromuscular transmission.**   1. **Prednisone 1 mg/kg po once/day:**   **High-dose glucocorticoids are standard for rapid immunosuppression in generalized myasthenia gravis.**   1. **MRI of the chest:**   **To evaluate for thymoma or thymic hyperplasia (present in ~10-15% of MG patients).**   1. **Azathioprine 50 mg po once/day:**   **A steroid-sparing agent for long-term immunosuppression (requires slow dose escalation and monitoring).**  **Why Other Options Are Less Appropriate:**   - **Admit for plasma exchange/IVIG:**   **Reserved for myasthenic crisis (e.g., respiratory failure, severe bulbar weakness) or preoperative optimization. Not indicated here unless the patient is in crisis (not described in the case).**   - **Prednisone 20 mg/day:**   **Too low to induce remission in generalized MG. High-dose steroids (1 mg/kg) are standard.**   - **X-ray of the chest:**   **Less sensitive than MRI for detecting thymoma or thymic pathology.**  **Next Steps:**   - **Start pyridostigmine immediately for symptom relief.** - **Initiate prednisone 1 mg/kg (e.g., 60-80 mg/day for an average adult) to suppress autoimmunity.** - **Add azathioprine (with gradual dose escalation to 2-3 mg/kg/day) to reduce long-term steroid dependence.** - **Perform MRI chest to rule out thymoma; if present, refer for thymectomy.**   **Monitoring:**   - **Assess for steroid side effects (e.g., hyperglycemia, osteoporosis).** - **Monitor azathioprine with CBC/LFTs (risk of cytopenia/hepatotoxicity).** - **Follow-up EMG or clinical exams to evaluate treatment response.** | **Most Appropriate Steps for Diagnosis and Treatment:**   1. **Pyridostigmine 60 mg po q 4-6 h**   **Rationale**: First-line symptomatic therapy to improve neuromuscular transmission by inhibiting acetylcholinesterase. Provides rapid but temporary relief of weakness.   1. **Prednisone 1 mg/kg po once/day**   **Rationale**: High-dose glucocorticoids are standard for inducing remission in generalized MG. Avoid starting with low doses (e.g., 20 mg), which may delay efficacy or worsen symptoms transiently.   1. **MRI of the Chest**   **Rationale**: Essential to evaluate for thymoma (present in ~15% of MG patients) or thymic hyperplasia. MRI is superior to chest X-ray for detecting small thymic lesions.   1. **Azathioprine 50 mg po once/day** (optional, but reasonable for long-term management)   **Rationale**: Steroid-sparing agent to reduce long-term steroid use. Requires gradual dose escalation and takes 3-6 months for full effect.  **Steps Not Immediately Indicated:**   - **Admit for Plasma Exchange (PLEX) or IVIG**:   Reserved for **myasthenic crisis** (e.g., respiratory failure, severe bulbar weakness) or rapid preoperative optimization. Not needed here unless the patient has life-threatening symptoms.   - **Chest X-ray**:   Less sensitive than MRI for thymoma evaluation. Only use if MRI is unavailable.   - **Prednisone 20 mg/day**:   Subtherapeutic for MG. High-dose steroids (1 mg/kg) are required to suppress autoimmunity effectively.  **Conclusion:** Immediate priorities are **symptomatic relief with pyridostigmine**, **immunosuppression with high-dose prednisone**, and **thymus imaging via MRI**. Azathioprine can be initiated early for long-term steroid-sparing effects. Avoid aggressive therapies (PLEX/IVIG) unless the patient deteriorates. | **Most Appropriate Next Steps**: ✅ **Prednisone 1 mg/kg po once/day**   - **Rationale**: High-dose corticosteroids (e.g., 1 mg/kg prednisone) are first-line immunosuppressive therapy for generalized MG to induce remission. Lower doses (e.g., 20 mg/day) are insufficient for disease control.   ✅ **Pyridostigmine 60 mg po q 4-6 h**   - **Rationale**: Acetylcholinesterase inhibitors (e.g., pyridostigmine) provide rapid symptomatic relief and are standard initial therapy for MG.   ✅ **Azathioprine 50 mg po once/day**   - **Rationale**: Azathioprine is a steroid-sparing agent used for long-term immunosuppression. It is typically initiated early but takes 3-6 months to reach full effect.   ✅ **MRI of the chest**   - **Rationale**: MRI is superior to X-ray for detecting thymoma or thymic hyperplasia, which occur in ~15% of MG patients and guide decisions about thymectomy.   **Why Other Options Are Less Appropriate**:   - **Admit for plasma exchange or IVIG**:   Reserved for **myasthenic crisis** (e.g., respiratory failure, severe bulbar weakness). No evidence of crisis here.   - **Prednisone 20 mg/day**:   Subtherapeutic for generalized MG. High-dose steroids (1 mg/kg) are required to suppress autoimmunity.   - **Chest X-ray**:   Less sensitive than MRI for detecting thymoma (small lesions or hyperplasia may be missed).  **Immediate Plan**:   1. Start **pyridostigmine** for symptom control. 2. Initiate **prednisone 1 mg/kg/day** and monitor for transient worsening (common in first 1-2 weeks). 3. Add **azathioprine** for long-term steroid-sparing effect. 4. Schedule **MRI chest** to evaluate for thymoma.   **Monitoring**:   - Assess for side effects of immunosuppression (e.g., hyperglycemia from steroids, leukopenia from azathioprine). - Watch for myasthenic crisis (urgent intervention needed if respiratory weakness develops). |
| Case12 |  |  |  |
| Diff | **Most Likely Differential Diagnoses:**   1. **Testicular Torsion**   **Key Evidence: Sudden-onset severe scrotal pain during physical activity, transient resolution followed by recurrence, nausea, left scrotal swelling/tenderness, negative left cremasteric reflex, and horizontal lie of the right testis (bell-clapper deformity). These findings are classic for torsion.**   1. **Epididymitis/Orchitis**   **Considered but less likely: No dysuria, fever, or urinary symptoms. However, his sexual activity raises a small possibility of atypical or subacute infection.**  **Why Other Options Are Less Likely:**   - **Groin Muscle Strain:**   **Pain would localize to the groin, not the scrotum, and there would be no scrotal swelling or absent cremasteric reflex.**   - **Incarcerated Inguinal Hernia:**   **Exam shows no palpable inguinal mass or tenderness over the inguinal canal.**   - **Nephrolithiasis:**   **Typically causes flank pain, costovertebral angle tenderness, or hematuria. Pain is scrotal, and abdominal exam is normal.**   - **Testicular Cancer:**   **Presents with a painless mass, not acute severe pain.**   - **Testicular Fracture:**   **Requires significant trauma (not reported here).**  **Immediate Priorities:**   1. **STAT Scrotal Ultrasound with Doppler to confirm/testicular torsion.** 2. **Emergency Urology Consultation for possible surgical exploration.**   **Conclusion: Testicular torsion is the primary diagnosis, with epididymitis/orchitis as a secondary consideration. Immediate intervention is critical to salvage the testis.** | **Most Likely Differential Diagnoses:**   1. **Testicular Torsion**   **Key Evidence**: Sudden onset of severe scrotal pain during physical activity, intermittent pain (suggesting torsion-detorsion), absent left cremasteric reflex, swollen/tender left testis, and horizontal lie of the right testis (indicative of **bell-clapper deformity**).   1. **Epididymitis/Orchitis**   **Consider**: Sexually active patient with multiple partners and inconsistent condom use. However, the absence of dysuria, fever, or penile discharge makes this less likely. Torsion remains the priority.   1. **Incarcerated Inguinal Hernia**   **Consider**: Sudden pain during exertion (shoveling) can occur with hernia incarceration. However, no inguinal mass or tenderness was noted on exam, making this less likely.  **Less Likely Diagnoses:**   - **Groin Muscle Strain**: Pain would localize to the groin, not the scrotum, and exam findings (e.g., swelling, absent cremasteric reflex) are inconsistent. - **Nephrolithiasis**: Typically causes flank/CVA tenderness and hematuria, which are absent here. - **Testicular Cancer**: Presents with a painless mass, not acute pain/swelling. - **Testicular Fracture**: Requires significant trauma, which is absent in this history.   **Immediate Priority**: **Testicular torsion is the leading diagnosis** and requires **urgent scrotal ultrasound with Doppler** and **urology consultation**. Delayed treatment risks testicular loss. Epididymitis and hernia are secondary considerations but do not preclude ruling out torsion first. | **Most Likely Differential Diagnoses**: ✅ **Testicular Torsion**   - **Key Support**: Sudden-onset pain, absent cremasteric reflex, swollen/tender testis, and contralateral bell clapper deformity (horizontal lie). Classic for torsion, especially with activity triggering symptoms.   ✅ **Epididymitis/Orchitis**   - **Key Support**: Scrotal pain/swelling. However, **less likely** due to absence of dysuria, fever, or urethral discharge. Still a consideration as it is a common mimic.   ✅ **Incarcerated Inguinal Hernia**   - **Key Support**: Scrotal swelling/pain. However, **less likely** due to lack of inguinal mass/tenderness. Hernias can occasionally present without obvious signs.   **Less Likely Options**: ❌ **Groin Muscle Strain**   - Pain location (scrotal vs. groin) and physical exam (no musculoskeletal tenderness) make this unlikely.   ❌ **Nephrolithiasis**   - Typically causes flank pain radiating to the groin, not isolated scrotal pain. No hematuria or costovertebral angle tenderness.   ❌ **Testicular Cancer**   - Presents with a **painless mass**, not acute pain/tenderness.   ❌ **Testicular Fracture**   - Requires significant trauma (not reported here).   **Critical Takeaway**:   - **Testicular torsion is the top priority** given the clinical findings (e.g., absent cremasteric reflex, bell clapper deformity). Immediate action (ultrasound, urology consult) is required to salvage the testis. - **Epididymitis/hernia** remain differentials but lack key supporting features. |
| Diag | **Most Appropriate Immediate Steps to Order:**   1. **Doppler Ultrasonography of the Scrotum**   **Priority: First-line imaging to assess testicular blood flow and confirm/rule out torsion.**   1. **Surgical Exploration of the Scrotum**   **Priority: Required emergently if ultrasound confirms torsion or if imaging is delayed. Clinical suspicion (sudden pain, absent cremasteric reflex, bell-clapper deformity) justifies immediate surgical intervention.**  **Why Other Options Are Less Immediate:**   - **Urinalysis: May help evaluate for infection (e.g., epididymitis) but does not take precedence over confirming torsion.** - **Urethral Swab for STI: Relevant if epididymitis is suspected, but the patient lacks dysuria, discharge, or fever.** - **CBC/BMP: Not urgent in this context; no signs of systemic illness or electrolyte imbalance.** - **Nuclear Scan of Scrotum: Rarely used; Doppler ultrasound is faster and more accessible.**   **Critical Action:**   - **Urology Consultation must occur concurrently with imaging to avoid delays. If torsion is confirmed or clinical suspicion remains high, surgery should proceed within 6 hours to salvage the testis.**   **Conclusion: Doppler ultrasonography and surgical exploration are the most critical immediate steps. Other tests (e.g., urinalysis) are secondary and should not delay definitive management.** | **Most Appropriate Immediate Steps to Order:**   1. **Doppler Ultrasonography of the Scrotum**:   **Priority**: First-line imaging to assess testicular blood flow. Absent/reduced flow confirms torsion.  **Urgency**: Must be performed immediately to avoid delays in surgical intervention.   1. **Surgical Exploration of the Scrotum**:   **Priority**: Required if ultrasound confirms torsion or if clinical suspicion remains high despite equivocal imaging.  **Urgency**: Time-critical; irreversible ischemia occurs within 6–12 hours.   1. **Urinalysis**:   **Rationale**: Helps rule out epididymitis/orchitis (e.g., pyuria, bacteriuria), though less likely in this case.  **Less Appropriate or Non-Urgent Steps:**   - **Nuclear Scan of Scrotum**: Rarely used due to time constraints; Doppler ultrasound is faster and more practical. - **Urethral Swab for STI**: Consider if epididymitis is suspected, but torsion takes precedence. - **CBC/BMP**: Not urgent here; no signs of systemic infection (e.g., fever) or renal dysfunction.   **Critical Action Plan:**   1. **STAT Doppler Ultrasound** → Confirm/rule out torsion. 2. **Simultaneous Urology Consultation** → Prepare for emergency surgery if imaging supports torsion. 3. **Urinalysis** → Quick adjunct to exclude infection.   **Do not delay imaging or surgery for other tests** (e.g., CBC, BMP, STI swab) in this time-sensitive scenario. | **Most Appropriate Immediate Steps to Order**:  ✅ **Doppler Ultrasonography of the Scrotum**   - **Rationale**: The **gold standard** to evaluate for testicular torsion by assessing blood flow. Immediate imaging is critical to confirm or rule out torsion.   ✅ **Surgical Exploration of the Scrotum**   - **Rationale**: If clinical suspicion for torsion is high (e.g., absent cremasteric reflex, bell clapper deformity), immediate surgical exploration is warranted **even without imaging**, as delays risk testicular necrosis.   ✅ **Urinalysis**   - **Rationale**: Helps evaluate for epididymitis/orchitis (e.g., pyuria, bacteriuria) if infection is still a consideration.   **Less Immediate/Priority Options**:  ❌ **Nuclear Scan of the Scrotum**   - **Rationale**: Historically used to assess testicular perfusion, but **less practical** due to time constraints and limited availability. Doppler ultrasound is faster and more accessible.   ❌ **Urethral Swab for STI**   - **Rationale**: Relevant if urethritis/epididymitis is suspected, but less urgent in this case given no dysuria/urethral discharge.   ❌ **CBC or BMP**   - **Rationale**: Non-specific in acute scrotal pain. May be ordered later if infection or systemic illness is suspected.   **Critical Actions**:   1. **Doppler ultrasound** must be performed **ASAP** (ideally within 1–2 hours). 2. **Consult urology immediately** while arranging imaging. If torsion is confirmed or strongly suspected, proceed to **emergency surgery** (detorsion ± orchiopexy). 3. **Urinalysis** can be done concurrently to support/refute epididymitis.   **Why Not Delay?**   - Testicular salvage rates drop significantly after 6–12 hours of torsion. Every minute counts! |
| Dx | **Most Likely Diagnosis: Testicular Torsion**  **Key Supporting Evidence:**   1. **Doppler Ultrasound Findings:**   **Absent venous/arterial blood flow in the left testis → Pathognomonic for torsion.**  **Heterogeneous appearance → Indicates ischemia or infarction due to interrupted blood supply.**   1. **Normal Urinalysis:**   **No signs of infection (negative leukocyte esterase, nitrites, bacteria) → Excludes acute epididymitis.**   1. **Exclusion of Other Diagnoses:**   **Testicular appendage torsion: Preserved testicular blood flow on Doppler.**  **Incarcerated inguinal hernia: Physical exam would show a groin mass/bowel obstruction symptoms (not mentioned here).**  **Implication: Testicular torsion is a surgical emergency. Immediate intervention (surgical exploration and orchiopexy) is required to salvage the testis.** | **Most Likely Diagnosis:** **Testicular Torsion**  **Key Supporting Evidence:**   1. **Doppler Ultrasound Findings**:   **Absent venous/arterial blood flow** in the left testis.  **Heterogeneous echotexture** (indicative of ischemia/infarction).  Normal blood flow to the right testis.   1. **Urinalysis**:   **Normal** (no pyuria, bacteriuria, or leukocyte esterase), ruling out epididymitis/orchitis.   1. **Clinical Context**:   Testicular torsion is a **time-sensitive emergency** requiring immediate surgical intervention.  **Why Other Options Are Less Likely:**   - **Acute Epididymitis**:   Typically presents with dysuria, fever, and **pyuria/bacteriuria on urinalysis** (absent here).  Doppler ultrasound would show **increased blood flow** (hyperemia) to the affected testis.   - **Testicular Appendage Torsion**:   Usually preserves testicular blood flow on Doppler ultrasound.  Pain may localize to the upper pole of the testis (not the entire testis).   - **Incarcerated Inguinal Hernia**:   Associated with a palpable groin mass, bowel obstruction symptoms (e.g., vomiting, distension), and normal testicular blood flow on ultrasound.  **Conclusion**: The combination of **absent blood flow** on Doppler ultrasound, **heterogeneous testicular appearance**, and **normal urinalysis** is **diagnostic of testicular torsion**. Immediate surgical exploration is required to salvage the testis. | **Correct Diagnosis**: ✅ **Testicular Torsion**  **Key Supporting Evidence**:   1. **Doppler Ultrasound**:   **Absent venous/arterial blood flow** in the left testis → Diagnostic for torsion.  **Heterogeneous testicular echotexture** → Indicates ischemia/necrosis from prolonged torsion.   1. **Urinalysis**:   **No signs of infection** (negative leukocyte esterase, nitrites, bacteria) → Rules out epididymitis.   1. **Clinical Context**:   Acute scrotal pain with absent blood flow is **pathognomonic for torsion**.  **Why Not Other Options?**   - ❌ **Acute Epididymitis**: Requires pyuria, positive infection markers, or bacterial growth. - ❌ **Testicular Appendage Torsion**: Preserves testicular blood flow; ultrasound shows a hyperechoic nodule with surrounding hyperemia. - ❌ **Incarcerated Inguinal Hernia**: Ultrasound would show bowel/omentum in the inguinal canal, not absent testicular blood flow.   **Urgency**: Immediate surgical exploration is required to salvage the testis. |
| Mang’ | **Most Appropriate Management Options:**   1. **Consult a urologist for immediate surgical exploration**   **Rationale: Testicular torsion is a surgical emergency. Surgical detorsion and orchiopexy (or orchiectomy, if nonviable) must occur within 6 hours of symptom onset to salvage the testis.**   1. **Do manual detorsion at the bedside**   **Rationale: Bedside manual detorsion (twisting the testis medial to lateral, like "opening a book") can restore blood flow while awaiting surgery. However, this does not replace surgical exploration, as definitive fixation is required.**   1. **Administer morphine 8 mg IV**   **Rationale: Severe scrotal pain is typical in torsion. IV opioids (e.g., morphine) provide rapid, effective analgesia.**  **Why Other Options Are Incorrect:**   - **Ciprofloxacin 500 mg IV: Not indicated. Negative urinalysis and absence of infection signs (fever, dysuria) rule out epididymitis/orchitis.** - **Ibuprofen 800 mg PO: NSAIDs may be used for adjunct pain control but are insufficient alone for acute, severe pain.** - **Admit for bed rest and repeat ultrasonography: Delaying surgery risks testicular necrosis. Repeat imaging is unnecessary after confirming torsion.**   **Key Takeaway: Immediate surgery (with bedside detorsion if feasible) + aggressive pain control are critical.** | **Most Appropriate Management Options:**   1. **Consult a urologist for immediate surgical exploration**   **Rationale**: Testicular torsion is a **time-sensitive surgical emergency**. Immediate exploration allows for detorsion, assessment of testicular viability, and bilateral orchiopexy (fixation) to prevent recurrence.   1. **Do manual detorsion at the bedside**   **Rationale**: Attempting manual detorsion (typically rotating the affected testis **laterally** "openingabook""*openingabook*") may restore blood flow while awaiting surgery. However, this does **not replace surgical intervention**, as fixation is still required.   1. **Administer morphine 8 mg IV**   **Rationale**: Severe pain is common in torsion. Morphine provides rapid analgesia while preparing for surgery.  **Why Other Options Are Incorrect:**   - **Administer ciprofloxacin 500 mg IV**:   Not indicated. Urinalysis shows **no evidence of infection** (negative nitrites, leukocyte esterase, bacteria). Epididymitis/orchitis is ruled out.   - **Administer ibuprofen 800 mg po**:   Less effective for severe acute pain. Opioids (e.g., morphine) are preferred preoperatively.   - **Admit for bed rest and repeat ultrasonography in 12 h**:   **Contraindicated**. Delaying surgery risks irreversible testicular necrosis. Doppler findings (absent blood flow + heterogeneous testis) already confirm torsion; repeat imaging is unnecessary.  **Critical Action Sequence:**   1. **Immediate urology consult** for surgical exploration. 2. **Attempt bedside manual detorsion** while awaiting the operating room. 3. **Administer IV morphine** for pain control.   **Time Matters**:   - Salvage rates drop significantly after **6–12 hours** of ischemia. Every minute counts. | **Most Appropriate Management Options**: ✅ **Consult a urologist for immediate surgical exploration**   - **Rationale**: Definitive treatment for testicular torsion requires **urgent surgical detorsion and bilateral orchiopexy** to salvage the testis and prevent recurrence.   ✅ **Administer morphine 8 mg IV**   - **Rationale**: Severe pain is common in torsion. Opioids (e.g., morphine) are first-line for acute pain control while preparing for surgery.   ✅ **Do manual detorsion at the bedside**   - **Rationale**: Temporizing measure to restore blood flow while awaiting surgery. Detorsion is performed by rotating the affected testicle **medially** (like "opening a book"). Success is confirmed by pain relief and return of blood flow on repeat Doppler.   **Why Not Other Options?**   - ❌ **Administer ciprofloxacin 500 mg IV**: No evidence of infection (normal urinalysis). Antibiotics are unnecessary. - ❌ **Administer ibuprofen 800 mg po**: NSAIDs are insufficient for severe torsion pain and do not address the underlying emergency. - ❌ **Admit for bed rest and repeat ultrasonography in 12 h**: Delaying surgery risks testicular necrosis. Immediate exploration is required.   **Key Point**: Surgical exploration must occur **within 6–12 hours** of symptom onset for optimal salvage rates. Manual detorsion and pain management are adjuncts but do not replace surgery. |
| Case13 |  |  |  |
| Diff | **Differential Diagnoses That Cannot Be Excluded:**   1. **Nephrolithiasis**   **Key Evidence: Acute left flank/groin pain, dark urine (possible hematuria).**  **Limitation: Absence of costovertebral angle (CVA) tenderness is noted but does not rule out stones.**   1. **Diverticulitis**   **Key Evidence: Left-sided lower abdominal pain in a middle-aged patient.**  **Limitation: No fever or bowel changes, but early diverticulitis may lack systemic signs.**   1. **Dissecting Aortic Aneurysm**   **Key Evidence: Hypertension (risk factor), acute severe pain.**  **Limitation: Pain is not described as "tearing" or back-radiating, but dissection can mimic other causes.**   1. **Splenic Laceration/Contusion**   **Key Evidence: Trauma to the left side days earlier; potential delayed presentation.**  **Limitation: Pain is lower abdominal (spleen is in the left upper quadrant), but referred pain or missed injury is possible.**   1. **Appendicitis**   **Key Evidence: Acute abdominal pain with nausea.**  **Limitation: Pain is left-sided (classically right-sided), but atypical presentations (e.g., pelvic appendix) occur.**   1. **Urinary Tract Infection (UTI)**   **Key Evidence: Dark urine.**  **Limitation: No dysuria or fever, but asymptomatic bacteriuria or atypical presentations exist.**  **Why Other Options Are Less Likely:**   - **Bowel Obstruction: No vomiting, distension, or constipation.** - **Cholecystitis: Right upper quadrant pain absent; no fatty food triggers.** - **Colon Cancer: No weight loss, bleeding, or bowel habit changes.** - **Epididymitis: Normal GU exam and no dysuria/urinary symptoms.** - **Gastroenteritis: No diarrhea/vomiting.** - **Ischemic Colitis: No bloody stools or risk factors (e.g., atrial fibrillation).** - **Pancreatitis: Pain is epigastric, not lower abdominal; no alcohol use or hypertriglyceridemia.**   **Conclusion: Further evaluation with non-contrast CT abdomen/pelvis (to assess stones, diverticulitis, aneurysm, splenic injury) and urinalysis (for hematuria/UTI) is critical.** | **Differential Diagnoses That Cannot Be Excluded:**   1. **Appendicitis**   While classically right-sided, **atypical presentations** (e.g., left-sided pain due to a long appendix or malrotation) cannot be excluded without imaging.   1. **Bowel Obstruction**   Early obstruction may lack classic signs (e.g., vomiting, distension). His **nausea and acute pain** warrant consideration until imaging (e.g., CT) is performed.   1. **Dissecting Aortic Aneurysm**   **Risk factors**: Age, anticoagulation (rivaroxaban). Sudden, severe pain with radiation could suggest dissection. Absence of pulse deficits or hypertension does not rule it out.   1. **Diverticulitis**   Common in the left lower quadrant. His **left-sided pain** and age align, though absence of fever or tenderness reduces likelihood. Requires CT for confirmation.   1. **Nephrolithiasis**   **Most likely diagnosis** (flank-to-groin pain, dark urine), but still a differential until confirmed by imaging (CT or ultrasound).   1. **Pancreatitis**   Atypical location (lower abdominal pain), but **diabetes and obesity** are risk factors. Requires lipase/amylase testing and imaging to exclude.   1. **Splenic Laceration/Contusion**   History of **trauma days prior** raises concern for delayed splenic injury. Pain location (left side) and anticoagulation increase risk, despite lack of tenderness.   1. **Urinary Tract Infection (UTI)**   **Dark urine** could indicate hematuria or infection. Asymptomatic UTIs occur, and urinalysis is needed to exclude.  **Differentials That Are Less Likely but Not Fully Excluded:**   - **Cholecystitis**: Left-sided pain is atypical, but variant anatomy (e.g., situs inversus) is possible. - **Ischemic Colitis**: Diabetes increases vascular risk, but absence of bloody stools makes this less likely.   **Excluded Diagnoses (Based on H&P):**   - **Epididymitis**: Normal GU exam and absence of dysuria/urinary symptoms make this unlikely. - **Gastroenteritis**: No diarrhea/vomiting, and pain is localized rather than diffuse. - **Colon Cancer**: Acute presentation and lack of bowel habit changes or occult blood make this less probable.   **Next Steps for Exclusion:**   1. **Imaging**: Non-contrast CT abdomen/pelvis (for stones, obstruction, diverticulitis, aneurysm, splenic injury). 2. **Urinalysis**: To assess for hematuria (nephrolithiasis) or infection (UTI). 3. **Laboratory Tests**: Lipase/amylase (pancreatitis), CBC (infection/inflammation), lactate (ischemia).   **Clinical Priority**: Rapidly rule out life-threatening conditions (e.g., aortic dissection, splenic injury) while evaluating for nephrolithiasis. | **Differential Diagnoses That Cannot Be Excluded**: ✅ **Nephrolithiasis**  **Rationale**: Flank-to-groin pain, dark urine (likely hematuria), and colicky pain are classic for kidney stones.  ✅ **Dissecting Aortic Aneurysm**  **Rationale**: Sudden, severe pain radiating to the groin in a hypertensive patient requires exclusion of this life-threatening condition.  ✅ **Diverticulitis**  **Rationale**: Left lower quadrant pain (though no fever or tenderness) could reflect early diverticulitis.  ✅ **Bowel Obstruction**  **Rationale**: Intermittent colicky pain and nausea raise suspicion, even without vomiting or distension.  ✅ **Ischemic Colitis**   - **Rationale**: Acute left-sided pain in a patient with diabetes and hypertension (vascular risk factors) warrants consideration.   ✅ **Splenic Laceration/Contusion**   - **Rationale**: History of left-sided trauma days prior; delayed complications (e.g., subcapsular hematoma) may present later.   ✅ **Urinary Tract Infection (UTI)**   - **Rationale**: Dark urine could indicate infection, especially in a diabetic patient (asymptomatic bacteriuria is possible).   **Why Other Diagnoses Are Excluded**:   - ❌ **Appendicitis**: Pain is left-sided, and the abdomen is nontender. - ❌ **Cholecystitis**: Pain is not in the right upper quadrant; no Murphy’s sign. - ❌ **Colon Cancer**: Acute onset and lack of weight loss/bleeding make this unlikely. - ❌ **Epididymitis**: Normal GU exam and absence of dysuria/scrotal symptoms. - ❌ **Gastroenteritis**: No diarrhea/vomiting. - ❌ **Pancreatitis**: Pain is not epigastric or radiating to the back.   **Next Steps**:   - **Imaging**: Non-contrast CT (for stones, diverticulitis, aneurysm, splenic injury). - **Urinalysis**: To evaluate for hematuria (stones) or UTI. - **Vascular Studies**: CTA if aortic dissection is suspected. - **Labs**: Lipase (to rule out pancreatitis), lactate (for ischemic colitis).   **Key Takeaway**: Prioritize ruling out **aortic dissection** and **splenic injury** due to their acuity, even if less likely. |
| Diag | **Most Appropriate Diagnostic Steps:**   1. **Urinalysis**   **Rationale: Essential to detect hematuria (supports nephrolithiasis) or signs of infection (e.g., pyuria, bacteriuria). Dark urine suggests possible microscopic hematuria.**   1. **CBC (Complete Blood Count)**   **Rationale: Evaluates for anemia (if occult bleeding from trauma or aortic aneurysm) and leukocytosis (infection, though less likely without fever).**   1. **BMP (Basic Metabolic Profile)**   **Rationale: Assesses renal function (creatinine, BUN) to evaluate for obstruction-related kidney injury and electrolyte imbalances. Also monitors glucose in his diabetes.**   1. **CT of the Abdomen and Pelvis Without Contrast**   **Rationale: Gold standard for diagnosing nephrolithiasis. Also evaluates for diverticulitis, splenic injury (given recent trauma), aortic aneurysm, and other abdominal pathologies. Non-contrast avoids interference with stone visualization and minimizes risks in a patient on anticoagulation.**  **Why Other Options Are Less Appropriate:**   - **Abdominal Ultrasonography: Less sensitive for small stones and alternative diagnoses (e.g., diverticulitis, aneurysm).** - **Blood Cultures: No systemic signs of infection (e.g., fever, hypotension).** - **CT with Contrast: Non-contrast CT is preferred for stones. IV contrast obscures stone visualization and increases risk in renal impairment (BMP needed first). PO contrast unnecessary for acute abdominal pain evaluation.** - **Plain X-rays: Low sensitivity for stones and other critical diagnoses (e.g., diverticulitis, aneurysm).** - **Urine Culture: Not urgent unless urinalysis suggests infection.**   **Conclusion: Proceed with urinalysis, CBC, BMP, and non-contrast CT to confirm nephrolithiasis, assess for complications (e.g., obstruction), and evaluate alternative causes (e.g., splenic injury, aortic aneurysm).** | **Most Appropriate Steps for Diagnosis and Treatment:**   1. **Urinalysis**:   Essential to assess for **hematuria** (supporting nephrolithiasis) or signs of infection (e.g., leukocytes, nitrites).   1. **CBC (Complete Blood Count)**:   Identifies leukocytosis (suggesting infection/inflammation) or anemia (relevant for occult bleeding, especially given anticoagulation).   1. **Basic Metabolic Profile (BMP)**:   Evaluates renal function (critical before considering contrast imaging), electrolytes, and glucose (important for diabetes management).   1. **CT of the Abdomen and Pelvis Without Contrast**:   **Gold standard** for detecting **ureteral stones**, assessing for **hydronephrosis**, and evaluating alternative diagnoses (e.g., diverticulitis, bowel obstruction, splenic injury, aortic aneurysm). Non-contrast CT avoids interference with stone visualization.  **Additional Considerations:**   - **Abdominal Ultrasonography**:   Useful if radiation exposure is a concern or for evaluating the aorta (aneurysm) and kidneys (hydronephrosis), but less sensitive than CT for small stones or ureteral pathology.   - **Urine Culture**:   Indicated if urinalysis suggests infection (e.g., pyuria, bacteriuria), though the patient lacks classic UTI symptoms.  **Less Appropriate Options:**   - **CT with PO/IV Contrast**:   Not ideal for suspected nephrolithiasis (contrast obscures stones) unless bowel pathology (e.g., appendicitis, obstruction) is strongly suspected.   - **Plain X-rays of the Abdomen**:   Low sensitivity for ureteral stones and lacks detail for other abdominal pathologies.   - **Blood Cultures**:   Unnecessary without fever, sepsis signs, or evidence of systemic infection.  **Final Recommendations:**   - **Priority Testing**: Urinalysis, CBC, BMP, and non-contrast CT abdomen/pelvis. - **Follow-Up**: If CT confirms nephrolithiasis, proceed with analgesia, hydration, and urology consultation if indicated. If imaging reveals alternative pathology (e.g., aortic dissection, splenic injury), escalate care accordingly. | **Most Appropriate Steps for Diagnosis and Treatment**:  ✅ **Urinalysis**  **Rationale**: Essential to detect hematuria (supporting nephrolithiasis) or signs of infection (e.g., pyuria, nitrites).  ✅ **Basic Metabolic Profile (BMP)**  **Rationale**: Assesses renal function (critical before contrast imaging), electrolytes, and glucose (relevant for diabetes management).  ✅ **CBC**  **Rationale**: Evaluates for leukocytosis (suggesting infection/inflammation like diverticulitis) or anemia (if occult bleeding is suspected).  ✅ **CT of the Abdomen and Pelvis Without Contrast**  **Rationale**: Gold standard for diagnosing **nephrolithiasis** and detecting ureteral stones. Also evaluates for bowel obstruction, splenic injury (given trauma history), and aortic pathology (e.g., aneurysm). Non-contrast is preferred initially to avoid obscuring stones.  **Why Other Options Are Less Appropriate**:   - ❌ **CT with PO/IV Contrast**: IV contrast can mask kidney stones and poses a risk if renal impairment (BMP needed first). PO contrast is unnecessary without suspicion of bowel perforation. - ❌ **Abdominal Ultrasonography**: Less sensitive for small stones and aortic dissection compared to CT. - ❌ **Plain X-rays**: Low sensitivity for non-calcified stones and other acute pathologies. - ❌ **Blood Cultures/Urine Culture**: No fever or clear signs of infection (e.g., dysuria) to justify cultures upfront. - ❌ **Blood Cultures**: Not indicated without systemic signs of sepsis.   **Key Considerations**:   - If **aortic dissection** remains a concern after non-contrast CT, follow with a **CTA** (CT angiography). - If **diverticulitis** is suspected (e.g., leukocytosis on CBC), contrast CT may be added later. - **BMP results** guide decisions about IV contrast use if additional imaging is needed.   **Immediate Priorities**:   1. **Urinalysis + BMP/CBC** to confirm hematuria and renal function. 2. **Non-contrast CT** to confirm nephrolithiasis and rule out splenic injury/aortic pathology. 3. Tailor further imaging (e.g., CTA) based on initial findings and labs. |
| Dx | **Answer: Ureteral lithiasis**  **Rationale:**  **Key Evidence:**  **CT Findings: Explicitly shows ureteral obstruction, which is most commonly caused by a kidney stone (ureteral lithiasis).**  **Urinalysis: Significant hematuria (20-25 RBCs/HPF) without pyuria, leukocyte esterase, or nitrites, which aligns with stone-induced irritation rather than infection.**  **Clinical Context: Acute flank/groin pain and dark urine (typical of stone-related hematuria).**  **Why Other Options Are Less Likely:**  **Dissecting Aortic Aneurysm: CT would show aortic abnormalities; no mention of tearing/radiating chest/back pain.**   - **Diverticulitis: Presents with left lower quadrant pain, fever, leukocytosis, and CT findings of colonic inflammation (absent here).** - **Splenic Laceration/Contusion: Requires trauma history and CT evidence of splenic injury (normal hemoglobin and CT focused on ureter).** - **UTI: Negative urinalysis for WBCs, leukocyte esterase, nitrites, and bacteria rules this out.**   **Conclusion: The CT-confirmed ureteral obstruction combined with hematuria and classic symptoms makes ureteral lithiasis the definitive diagnosis.** | **MostLikely Diagnosis:** **Ureteral Lithiasis**  **Key Supporting Evidence:**   1. **Classic Symptoms**:   Sudden-onset left flank pain radiating to the groin (renal colic).  Colicky, waxing/waning pain worsened by movement (swimming).  Dark urine (microscopic/macroscopic hematuria).   1. **Risk Factors**:   Recent trauma (son running into his left side), which may have dislodged a pre-existing stone.  No signs of infection (normal WBC, no fever/dysuria).   1. **Imaging**:   Non-contrast CT is the gold standard for diagnosing ureteral stones.  **Why Other Diagnoses Are Less Likely:**   - **Dissecting Aortic Aneurysm**:   Typically presents with tearing/ripping pain, hypotension, or pulse deficits. Unlikely given positional pain relief (better when walking) and lack of risk factors (e.g., hypertension).   - **Diverticulitis**:   Left lower quadrant pain, fever, leukocytosis, and constipation/diarrhea are common. Absent here.   - **Splenic Laceration/Contusion**:   Trauma to the left side raises suspicion, but splenic injury usually causes left upper quadrant pain, referred shoulder pain, or anemia (normal hemoglobin here).   - **UTI**:   No dysuria, urgency, or fever. Normal WBC and urinalysis findings not suggestive.  **Conclusion**: The clinical presentation, absence of infection, and imaging findings (CT) strongly support **ureteral lithiasis** as the diagnosis. | **Most Likely Diagnosis**:  ✅ **Ureteral Lithiasis**  **Key Supporting Evidence**:  **Hematuria**: 20-25 RBCs/HPF on urinalysis (classic for nephrolithiasis).  **CT Findings**: Non-contrast CT showing involvement of the **ureter** (likely a ureteral stone).  **Absence of Infection**: No pyuria, bacteria, nitrites, or leukocyte esterase (rules out UTI).  **Normal WBC Count**: Makes diverticulitis or systemic infection unlikely.  **Trauma History Less Relevant**: Splenic laceration/contusion is not supported by normal CBC (no anemia, thrombocytopenia) or CT findings.  **Why Other Diagnoses Are Less Likely**:   - ❌ **Dissecting Aortic Aneurysm**:   No reported aortic dilation or dissection on CT (though non-contrast CT is suboptimal for full exclusion).  No mention of tearing/radiating pain, hypotension, or asymmetrical pulses.   - ❌ **Diverticulitis**:   Normal WBC count and lack of fever/leukocytosis.  Non-contrast CT would likely show bowel wall thickening or abscess if diverticulitis were present.   - ❌ **Splenic Laceration/Contusion**:   Trauma history is noted, but no anemia, hemodynamic instability, or CT findings (e.g., subcapsular hematoma).   - ❌ **UTI**:   Negative urinalysis for leukocytes, nitrites, and bacteria.  **Implication**: Ureteral lithiasis is the **leading diagnosis** based on current data. However, if pain persists or worsens, further evaluation (e.g., CTA for aortic dissection or contrast-enhanced CT for ischemic colitis) may still be warranted. |
| Mang | **Final Answer: ✅ Discharge from the emergency department with a urine strainer ✅ 0.9% saline solution, 1 L IV over 1 h ✅ Ketorolac 15 mg IV ✅ Ondansetron 4 mg IV (if nausea/vomiting present) ✅ Oxycodone/acetaminophen 5 mg/325 mg po q 6 h for 7 days**  **Rationale:**   1. **Discharge with urine strainer:**   **The patient is stable, with no infection (negative urinalysis for UTI) or acute kidney injury (normal creatinine). Most small stones (<6 mm) pass spontaneously with hydration and pain control. A strainer helps confirm stone passage and analysis.**   1. **IV fluids (0.9% saline):**   **Hydration promotes stone passage. IV fluids can be administered in the ED to ensure adequate hydration before discharge.**   1. **Ketorolac IV:**   **NSAIDs (e.g., ketorolac) are first-line for renal colic pain due to their anti-inflammatory effect on ureteral edema.**   1. **Ondansetron IV (if needed):**   **Ondansetron is appropriate if the patient has nausea/vomiting, a common symptom with renal colic.**   1. **Oxycodone/acetaminophen:**   **A short-course opioid (e.g., oxycodone) may be prescribed for breakthrough pain if NSAIDs alone are insufficient.**  **Excluded Options:**   - **Admit to the hospital: Unnecessary unless there are complications (e.g., intractable pain, infection, or complete obstruction).** - **Percutaneous nephrolithotomy/ureteroscopy/shock wave lithotripsy: These procedures are reserved for large stones (>6–10 mm), stones causing severe obstruction, or failure of conservative management.** - **Ciprofloxacin: No evidence of infection (negative urinalysis for leukocyte esterase, nitrites, and bacteria).**   **Conclusion: Outpatient management with pain control, hydration, and monitoring for stone passage is appropriate. Procedural interventions are not indicated unless the stone fails to pass or complications arise.** | **Answer:** The most appropriate management steps are:   - **Discharge from the emergency department with a urine strainer** - **0.9% saline solution, 1 L IV over 1 h** - **Ketorolac 15 mg IV** - **Ondansetron 4 mg IV**   **Rationale:**   1. **Discharge with a urine strainer**:   The patient has **uncomplicated ureteral lithiasis** (no fever, normal renal function, no infection). Small stones (<6–10 mm) often pass spontaneously with hydration and pain control. A urine strainer helps confirm stone passage.   1. **IV fluids (0.9% saline)**:   Hydration promotes diuresis, aiding stone passage. IV fluids are commonly administered to ensure adequate hydration, especially if oral intake is limited.   1. **Ketorolac (NSAID)**:   First-line for renal colic pain. NSAIDs reduce ureteral inflammation and pain effectively.   1. **Ondansetron**:   Nausea/vomiting frequently accompanies renal colic. Ondansetron provides symptomatic relief even if not explicitly documented.  **Excluded Options**:   - **Admit to the hospital**: Unnecessary unless complications (e.g., intractable pain, infection, acute kidney injury) arise. - **Percutaneous nephrolithotomy/ureteroscopy/shock wave lithotripsy**: Reserved for large stones (>10 mm), failed conservative management, or complications (e.g., infection). - **Ciprofloxacin**: No evidence of infection (negative urinalysis for bacteria/nitrites). - **Oxycodone/acetaminophen**: NSAIDs (e.g., oral ibuprofen) are preferred for outpatient pain control. Opioids are discouraged unless absolutely necessary.   **Summary**: Conservative management with hydration, analgesia, antiemetics, and outpatient follow-up is appropriate for uncomplicated ureteral lithiasis. | **Most Appropriate Management Steps**:  ✅ **Discharge from the emergency department with a urine strainer**   - Rationale: The patient is stable (normal renal function, no infection, controlled pain) and likely has a small, passable ureteral stone. A urine strainer allows for stone analysis upon passage.   ✅ **0.9% saline solution, 1 L IV over 1 h**   - Rationale: Hydration promotes stone passage and addresses potential dehydration. IV fluids are standard in acute renal colic management.   ✅ **Ketorolac 15 mg IV**   - Rationale: NSAIDs (e.g., ketorolac) are **first-line for renal colic pain** due to their anti-inflammatory effect on ureteral spasms.   ✅ **Ondansetron 4 mg IV**   - Rationale: Nausea/vomiting often accompanies renal colic. Ondansetron provides symptomatic relief, even if not explicitly documented.   ✅ **Oxycodone/acetaminophen 5 mg/325 mg po q 6 h for 7 days**   - Rationale: A short-course opioid (e.g., oxycodone) may be needed for breakthrough pain, though NSAIDs should remain primary.   **Not Appropriate**:   - ❌ **Admit the patient to the hospital**: No indications for admission (e.g., infection, obstruction with renal failure, intractable pain). - ❌ **Percutaneous nephrolithotomy/ureteroscopic stone removal/shock wave lithotripsy**: Reserved for large stones (>10 mm), failed conservative management, or complications (e.g., obstruction/infection). No evidence these are needed yet. - ❌ **Ciprofloxacin 500 mg po bid for 10 days**: No signs of UTI (negative nitrites, leukocyte esterase, bacteria).   **Key Considerations**:   1. **Outpatient follow-up** is critical to confirm stone passage and assess for complications. 2. **Alpha-blockers** (e.g., tamsulosin) are often used off-label to facilitate stone passage but are not listed here. 3. **Repeat imaging** (e.g., ultrasound) may be needed if pain persists or stone passage is not confirmed. |
| Case 14 |  |  |  |
| diff | **Differential Diagnoses That Cannot Be Excluded:**   1. **Cellulitis**   **Rationale: Erythema, warmth, swelling, pain, and systemic symptoms (fever, malaise) align with cellulitis. Diabetes increases infection risk.**   1. **Deep Vein Thrombosis (DVT)**   **Rationale: Unilateral leg swelling and pain are concerning. While calf tenderness and distended veins are absent, DVT cannot be ruled out without imaging (e.g., Doppler ultrasound). History of CABG (possible venous stasis) and leg swelling post-surgery are risk factors.**   1. **Necrotizing Soft-Tissue Infection**   **Rationale: A "must-not-miss" diagnosis. Diabetics are at higher risk, and early necrotizing infections may lack classic signs (e.g., crepitus, severe pain). Systemic toxicity (fever, malaise) warrants exclusion.**   1. **Superficial Thrombophlebitis**   **Rationale: Erythema and tenderness could indicate inflammation of a superficial vein, though systemic symptoms (fever) are atypical. Clinical overlap with cellulitis exists.**  **Excluded Diagnoses:**   - **Abscess: No fluctuance, purulence, or focal collection on exam.** - **Allergic Reaction: No pruritus, urticaria, or rash; systemic symptoms suggest infection.** - **Contusion: No history of trauma.** - **Erythema Migrans: Unlikely in December (non-peak tick season); rash lacks central clearing.** - **Insect Sting: No history of sting, pruritus, or acute reaction.**   **Key Considerations:**   - **Imaging: Doppler ultrasound to evaluate for DVT.** - **Labs: CBC, CRP, blood cultures, and glucose/HbA1c (given diabetes).** - **Antibiotics: Empiric IV antibiotics (e.g., vancomycin + piperacillin/tazobactam) for cellulitis or necrotizing infection pending cultures.** - **Surgical Consultation: If necrotizing infection is suspected (e.g., rapid progression, severe pain).**   **Final Answer:**   - **Cellulitis** - **Deep Vein Thrombosis (DVT)** - **Necrotizing Soft-Tissue Infection** - **Superficial Thrombophlebitis** | **Answer:** The differential diagnoses that **cannot be excluded** based on history and physical examination are:   - **Cellulitis** - **Deep vein thrombosis (DVT)** - **Necrotizing soft-tissue infection** - **Abscess**   **Rationale:**   1. **Cellulitis**:   **Supporting**: Erythema, warmth, tenderness, systemic symptoms (fever, malaise), and risk factors (diabetes, post-CABG leg edema).  **Cannot exclude**: Classic presentation, but requires confirmation and exclusion of mimics.   1. **Deep vein thrombosis (DVT)**:   **Supporting**: Unilateral leg swelling (chronic post-CABG edema may mask acute DVT). Fever can occur in septic thrombophlebitis or concurrent infection.  **Cannot exclude**: Requires Doppler ultrasound to rule out, as DVT and cellulitis can coexist.   1. **Necrotizing soft-tissue infection**:   **Supporting**: Systemic symptoms (fever, malaise) and erythema. Diabetic patients are at higher risk.  **Cannot exclude**: Early necrotizing infections may lack "hard signs" (e.g., crepitus, severe pain). Imaging or surgical evaluation may be needed.   1. **Abscess**:   **Supporting**: Erythema and tenderness.  **Cannot exclude**: Small abscesses may not be fluctuant on exam. Ultrasound could identify a fluid collection.  **Excluded Diagnoses**:   - **Allergic reaction**: No itching, urticaria, or known allergen exposure. - **Contusion**: No history of trauma. - **Erythema migrans**: No bull’s-eye rash, tick exposure, or typical Lyme disease epidemiology (December presentation). - **Insect sting**: No history of bite/sting, and systemic symptoms suggest infection over local reaction. - **Superficial thrombophlebitis**: No palpable cord or venous distension. Erythema is diffuse, not linear.   **Next Steps**:   - **Imaging**: Doppler ultrasound (DVT evaluation) and/or MRI (if necrotizing infection suspected). - **Laboratory tests**: CBC, CRP, blood cultures. - **Empiric treatment**: Broad-spectrum antibiotics for cellulitis/necrotizing infection while awaiting results. - **Surgical consult**: If necrotizing infection cannot be ruled out clinically or radiographically.   **Summary**: Cellulitis is the leading diagnosis, but DVT, abscess, and necrotizing infection require exclusion through imaging and labs. | **Differential Diagnoses That Cannot Be Excluded**:  ✅ **Cellulitis**   - **Key Evidence**: Erythema, warmth, induration, fever, and systemic symptoms (malaise, poor appetite). Risk factors include diabetes and chronic leg swelling post-CABG.   ✅ **Deep Vein Thrombosis (DVT)**   - **Key Evidence**: Unilateral leg swelling (right > left) with a history of venous stasis post-saphenous vein harvest. Though calf compression tenderness is absent, DVT cannot be ruled out without imaging (e.g., Doppler ultrasound).   ✅ **Necrotizing Soft-Tissue Infection**   - **Key Evidence**: Systemic signs (fever, tachycardia) and diabetes increase suspicion. Early stages may lack classic findings (e.g., crepitus, severe pain). Requires close monitoring for rapid progression.   ✅ **Abscess**   - **Key Evidence**: Induration and erythema suggest possible underlying abscess, though fluctuance or purulent drainage is not reported. Deep abscesses may not be palpable on exam.   ✅ **Superficial Thrombophlebitis**   - **Key Evidence**: Chronic venous stasis post-CABG predisposes to superficial vein inflammation. Erythema and tenderness could align with this, though no palpable cord is noted.   **Diagnoses Likely Excluded**:   - ❌ **Allergic Reaction**: No pruritus, urticaria, or diffuse rash. Painful erythema is atypical for allergy. - ❌ **Erythema Migrans (Lyme Disease)**: Absence of targetoid rash, bull’s-eye lesion, or tick exposure history. Season (December) makes this less likely. - ❌ **Insect Sting**: No history of insect contact or acute sting reaction. - ❌ **Contusion**: No reported trauma. Systemic symptoms (fever) are inconsistent with simple bruising.   **Next Steps**:   1. **Imaging**: Doppler ultrasound to evaluate for DVT and rule out abscess. 2. **Labs**: CBC, blood cultures, inflammatory markers (e.g., CRP). 3. **Empiric Treatment**: Broad-spectrum IV antibiotics (cover *Staphylococcus*, *Streptococcus*, and MRSA) for cellulitis/necrotizing infection. 4. **Surgical Consultation**: If necrotizing infection is suspected (e.g., rapid clinical deterioration).   **Critical Consideration**: The patient’s **diabetes** and **venous insufficiency** heighten risks for both infection and thrombosis, necessitating thorough evaluation of both possibilities. |
| diag | **Essential Diagnostic Tests That Cannot Be Excluded: The following tests are necessary to confirm or rule out critical diagnoses in this patient:**   1. **Blood cultures**   **Rationale: Systemic symptoms (fever, malaise) and possible sepsis in a diabetic patient with cellulitis. Blood cultures help identify bacteremia and guide antibiotic therapy.**   1. **Complete blood count (CBC)**   **Rationale: To assess for leukocytosis (supports infection) or anemia. Critical for evaluating systemic inflammation or infection severity.**   1. **Basic metabolic panel (BMP)**   **Rationale: Evaluate glucose (poorly controlled diabetes increases infection risk), electrolytes, and renal function (relevant for IV antibiotic dosing and fluid management).**   1. **Doppler ultrasonography of the leg**   **Rationale: Unilateral leg swelling, pain, and CABG history (saphenous vein harvest) raise concern for DVT. Doppler is the gold standard for diagnosis.**   1. **Skin cultures**   **Rationale: If the erythematous area has exudate or breaks in the skin, cultures can identify pathogens (e.g., *Staphylococcus*, *Streptococcus*) and tailor antibiotics.**  **Excluded Tests:**   - **No additional testing: Unacceptable given systemic symptoms, diabetes, and risk for life-threatening conditions (e.g., DVT, necrotizing infection).** - **Chest x-ray: No respiratory symptoms (no cough, dyspnea) to suggest pneumonia or alternative diagnoses.** - **ECG: No chest pain, palpitations, or cardiac symptoms to warrant immediate evaluation.** - **Leg x-ray: No trauma, crepitus, or signs of osteomyelitis (e.g., chronic ulcers, bone pain).**   **Key Considerations:**   - **Necrotizing infection: Requires urgent surgical evaluation; imaging (CT/MRI) is more sensitive than x-ray but not listed here.** - **Empiric antibiotics: Start IV antibiotics (e.g., vancomycin + piperacillin/tazobactam) after cultures, pending results.** - **Glucose control: BMP will guide management of hyperglycemia, which worsens infection outcomes.**   **Final Answer:**   - **Basic metabolic panel** - **Blood cultures** - **Complete blood count (CBC)** - **Doppler ultrasonography of leg** - **Skin cultures** | **Answer:** The following diagnostic tests **cannot be excluded** (i.e., are necessary) based on the history and physical examination:   - **Blood cultures** - **Complete blood count (CBC)** - **Doppler ultrasonography of leg** - **Leg x-ray** - **Basic metabolic panel (BMP)**   **Rationale for Each Test**:   1. **Blood cultures**:   Systemic symptoms (fever, malaise) and risk factors (diabetes, possible bacteremia) warrant evaluation for bloodstream infection.   1. **CBC**:   Assess for leukocytosis, which supports an infectious process (e.g., cellulitis, abscess, necrotizing infection).   1. **Doppler ultrasonography of leg**:   Critical to exclude **DVT**, given unilateral leg swelling, post-CABG venous changes, and overlapping symptoms (pain, edema).   1. **Leg x-ray**:   Evaluate for soft-tissue gas (concern for necrotizing infection) or osteomyelitis (diabetes increases risk, though acute presentation makes osteomyelitis less likely).   1. **Basic metabolic panel (BMP)**:   Check glucose (poorly controlled diabetes worsens infection outcomes) and renal function (guides antibiotic dosing).  **Excluded Tests**:   - **No additional testing**: Risk factors and systemic symptoms necessitate further evaluation. - **Chest x-ray**: No respiratory symptoms (e.g., cough, dyspnea) to suggest pulmonary pathology. - **ECG**: No cardiac symptoms (e.g., chest pain, palpitations) to warrant immediate evaluation. - **Skin cultures**: Low yield in uncomplicated cellulitis without open wounds or purulence.   **Next Steps**:   - Start empiric antibiotics (cover for **Staphylococcus aureus**, including MRSA, and streptococci) after obtaining blood cultures. - Use Doppler ultrasound to rule out DVT and guide compression therapy if positive. - Leg x-ray may prompt MRI if necrotizing infection is suspected (e.g., rapid progression, severe pain). - BMP results will inform glycemic and renal management.   **Summary**: While cellulitis is the leading diagnosis, DVT, abscess, and necrotizing infection require exclusion via imaging and labs. These tests are essential for safe, targeted management. | **Essential Diagnostic Tests That Cannot Be Excluded (Required Based on Presentation):**   - ✅ **Basic metabolic panel (Na⁺, Cl⁻, K⁺, HCO₃⁻, BUN, creatinine, glucose)**   **Rationale**: Critical to evaluate glucose (poorly controlled diabetes), renal function (before antibiotics), and electrolyte imbalances (fever/poor intake).   - ✅ **Blood cultures**   **Rationale**: Systemic signs (fever, tachycardia) raise concern for bacteremia; cultures guide antibiotic therapy.   - ✅ **Complete blood count (CBC)**   **Rationale**: Assess for leukocytosis (infection severity) or anemia.   - ✅ **Doppler ultrasonography of leg**   **Rationale**: Unilateral swelling + post-CABG venous stasis necessitates ruling out DVT.   - ✅ **Leg x-ray**   **Rationale**: Evaluate for gas (necrotizing infection) or osteomyelitis (diabetes risk), though MRI is more sensitive.  **Tests Likely Not Needed at This Time**:   - ❌ **No additional testing (clinical diagnosis only)**   Systemic symptoms and comorbidities demand objective evaluation.   - ❌ **Chest x-ray**   No respiratory symptoms (e.g., cough, dyspnea) to suggest pulmonary pathology.   - ❌ **ECG**   No chest pain or cardiac symptoms; tachycardia likely secondary to fever.   - ❌ **Skin cultures**   Skin is intact; cultures from intact skin have low yield. Aspiration/biopsy preferred if abscess suspected.  **Next Steps**:   1. **Immediate empiric antibiotics** (e.g., vancomycin + ceftriaxone) for cellulitis/necrotizing infection. 2. **Ultrasound results** will guide DVT management (anticoagulation if positive). 3. **Leg x-ray** findings may prompt MRI for osteomyelitis or surgical consultation for necrotizing infection. 4. **Adjust therapy** based on blood cultures and metabolic panel results (e.g., glucose control, renal dosing).   **Critical Considerations**:   - **Diabetes**: Poor glycemic control increases infection risk and complicates healing. - **Post-CABG venous stasis**: Chronic swelling predisposes to DVT and recurrent cellulitis. - **Necrotizing infection**: High suspicion if pain worsens rapidly or crepitus develops. |
| dx | **Most Likely Diagnosis: Cellulitis**  **Rationale:**   1. **Clinical and Lab Evidence of Infection:**   **Elevated WBC (14.5 x 10⁹/L) with neutrophilia (86%) and left shift (8% bands) strongly support a bacterial infection.**  **Systemic signs (fever, tachycardia) align with cellulitis progression.**   1. **Normal Imaging:**   **Normal leg X-ray: No evidence of abscess, osteomyelitis, or gas (which would suggest necrotizing infection).**  **Normal Doppler ultrasound: Rules out DVT.**   1. **Diabetic Risk Factor:**   **Hyperglycemia (glucose 220 mg/dL) predisposes to skin infections like cellulitis.**   1. **Exclusion of Necrotizing Infection:**   **Necrotizing infections typically present with severe pain out of proportion to exam, crepitus, or skin necrosis—none documented here.**  **Normal imaging does not fully exclude early necrotizing infection, but cellulitis is far more common in this clinical context.**  **Why Not Other Diagnoses?**   - **Abscess: No fluctuance, purulence, or imaging findings (e.g., fluid collection).** - **DVT: Excluded by normal Doppler ultrasound.** - **Necrotizing Subcutaneous Infection: No red flags (e.g., crepitus, hemorrhagic bullae, rapid progression) to prioritize this over cellulitis.**   **Conclusion: While necrotizing infection remains a critical consideration in diabetic patients with cellulitis, the absence of specific clinical/imaging findings makes cellulitis the most likely diagnosis. Close monitoring for worsening symptoms is essential.**  **Final Answer:**   - **Cellulitis** | **Answer:** **• Cellulitis**  **Rationale**:   1. **Supporting Evidence**:   **Infection Markers**: Elevated WBC (14.5 x10⁹/L) with left shift (86% neutrophils + 8% bands) strongly indicate a bacterial infection.  **Clinical Signs**: Erythema, warmth, and swelling in the leg are classic for cellulitis.  **Imaging**: Normal X-ray and Doppler ultrasound rule out DVT, osteomyelitis, and large abscesses.   1. **Excluded Diagnoses**:   **DVT**: Excluded by normal Doppler ultrasound.  **Abscess**: No fluctuance, purulence, or imaging evidence of a fluid collection.  **Necrotizing Subcutaneous Infection**: No systemic instability, severe pain, or soft-tissue gas on X-ray.   1. **Context**:   Poorly controlled diabetes (glucose 220 mg/dL) increases susceptibility to soft-tissue infections like cellulitis.  Sinus tachycardia and elevated BUN/creatinine likely reflect dehydration from systemic infection.  **Next Step**: Continue empiric antibiotics targeting *Staphylococcus aureus* (including MRSA) and streptococci, with close monitoring for abscess formation or worsening infection. | **Most Likely Diagnosis**: ✅ **Cellulitis**  **Key Supporting Evidence:**   1. **Clinical Presentation**:   Erythema, warmth, and induration of the right lower leg.  Systemic signs of infection: fever (38.8°C), tachycardia (120 bpm).   1. **Lab Findings**:   **Leukocytosis (WBC 14.5 x 10⁹/L)** with **left shift (86% neutrophils + 8% bands)**—classic for bacterial infection.  **Elevated glucose (220 mg/dL)**: Poorly controlled diabetes predisposes to skin/soft tissue infections.   1. **Imaging**:   **Normal leg X-ray**: Rules out gas (necrotizing infection) or osteomyelitis.  **Normal Doppler ultrasound**: Excludes DVT.   1. **Risk Factors**:   Diabetes mellitus (poorly controlled).  Post-CABG venous stasis (chronic leg swelling).  **Why Other Diagnoses Are Less Likely:**   - ❌ **Abscess**:   No fluctuance, palpable mass, or imaging evidence of fluid collection. Induration alone is nonspecific.   - ❌ **Deep Vein Thrombosis (DVT)**:   Normal Doppler ultrasound definitively excludes this.   - ❌ **Necrotizing Subcutaneous Infection**:   No severe pain out of proportion to exam, crepitus, or rapid progression. Normal X-ray makes gas-forming infection unlikely.  **Next Steps:**   1. **Empiric Antibiotics**:   **Vancomycin** (MRSA coverage) + **ceftriaxone** (Gram-negative coverage). Adjust for renal impairment (creatinine 1.4 mg/dL).   1. **Monitor for Complications**:   Worsening erythema, hemodynamic instability, or elevated lactate would prompt MRI to rule out necrotizing infection.   1. **Glucose Control**:   Insulin therapy to target glucose 140-180 mg/dL.  **Final Note**: While cellulitis is most likely, **necrotizing infection remains a critical "cannot miss" diagnosis**. Close monitoring for rapid clinical deterioration is essential. |
| mang | **Most Appropriate Management Options:**   1. **Admit the patient to the hospital**   **Rationale: Systemic signs (tachycardia, leukocytosis with left shift), hyperglycemia, and diabetes increase the risk of severe infection and complications, necessitating inpatient care.**   1. **Elevate the right leg while in bed**   **Rationale: Reduces edema, improves circulation, and aids in resolving cellulitis.**   1. **Blood cultures**   **Rationale: Indicated for suspected bacteremia due to systemic signs (fever, tachycardia) and immunocompromised status (diabetes).**   1. **Vancomycin 1 g IV every 12 hours**   **Rationale: Covers MRSA, which is a critical concern in diabetic patients with cellulitis, especially with severe or systemic infection.**   1. **Ibuprofen 600 mg PO every 6 hours as needed for pain**   **Rationale: Addresses pain and inflammation without oversedation; no evidence of severe pain requiring opioids.**  **Why Not Other Options?**   - **Encourage ambulation: Contraindicated in cellulitis, as activity can worsen edema and delay healing.** - **Cephalexin PO: Oral antibiotics are insufficient for systemic infection with leukocytosis and hyperglycemia.** - **Diphenhydramine: No evidence of allergic reaction; unnecessary for bacterial cellulitis.** - **Morphine IV: Not first-line for moderate pain; NSAIDs are preferred unless pain is severe.** - **Nafcillin IV: Lacks MRSA coverage, which is essential in high-risk diabetic patients.** - **Tobramycin + Piperacillin: Overly broad for uncomplicated cellulitis; reserved for necrotizing infections or Pseudomonas risk (not supported here).**   **Final Answer:**   - **Admit the patient to the hospital** - **Elevate the right leg while in bed** - **Blood cultures** - **Vancomycin 1 g IV every 12 h** - **Ibuprofen 600 mg PO every 6 h as needed for pain** | **Answer:** The most appropriate management options for this patient with **cellulitis** and poorly controlled diabetes are:   - **Admit the patient to the hospital** - **Elevate the right leg while in bed** - **Blood cultures** - **Vancomycin 1 g IV every 12 h** - **Morphine 8 mg IV every 6 h as needed for pain**   **Rationale**:   1. **Admit the patient**:   Hospitalization is warranted due to systemic signs of infection (leukocytosis, tachycardia), poorly controlled diabetes (glucose 220 mg/dL), and potential need for IV antibiotics and glycemic optimization.   1. **Elevate the leg**:   Reduces edema, improves lymphatic drainage, and limits infection spread.   1. **Blood cultures**:   Essential to identify bacteremia, particularly in a diabetic patient with systemic symptoms.   1. **Vancomycin**:   Covers **MRSA**, a critical pathogen in diabetic patients. Empiric MRSA coverage is recommended given diabetes and elevated inflammatory markers.   1. **Morphine (as needed)**:   Avoid NSAIDs (e.g., ibuprofen) due to elevated creatinine (1.4 mg/dL), which suggests renal impairment. Opioids are safer for pain control in this setting.  **Excluded Options**:   - **Cephalexin**: Inadequate for MRSA coverage in a high-risk diabetic patient. - **Nafcillin**: Covers MSSA/streptococci but not MRSA. - **Piperacillin/tobramycin**: Overly broad for typical cellulitis pathogens (strep/staph). - **Diphenhydramine**: No role in cellulitis management unless for allergic reactions (not indicated here). - **Encourage ambulation**: Elevation is prioritized over ambulation to reduce swelling.   **Next Steps**:   - Monitor for abscess formation (e.g., fluctuance) or necrotizing infection. - Optimize glycemic control with insulin. - Reassess antibiotics based on culture results and clinical response. | **Most Appropriate Management Options**:   1. ✅ **Admit the patient to the hospital**   Rationale: Systemic signs of infection (fever, leukocytosis, tachycardia), poorly controlled diabetes, and acute kidney injury necessitate inpatient care for IV antibiotics, monitoring, and metabolic optimization.   1. ✅ **Elevate the right leg while in bed**   Rationale: Reduces edema, improves lymphatic drainage, and promotes healing in cellulitis.   1. ✅ **Blood cultures**   Rationale: Systemic symptoms (fever, tachycardia) raise concern for bacteremia, especially in a diabetic patient. Cultures guide antibiotic therapy if pathogens are identified.   1. ✅ **Vancomycin 1 g IV every 12 hours**   Rationale: Covers MRSA, a critical pathogen in diabetic patients. Preferred over cephalexin (oral, narrow spectrum) and nafcillin (no MRSA coverage). Avoid tobramycin (nephrotoxic) in this patient with renal impairment.   1. ✅ **Morphine 8 mg IV every 6 hours as needed for pain**   Rationale: Safe for renal impairment (unlike ibuprofen, which risks worsening kidney injury). Provides effective analgesia for severe inflammation.  **Why Other Options Are Less Appropriate:**   - ❌ **Encourage ambulation**: Rest and elevation are prioritized initially to reduce swelling and spread of infection. Ambulation may be reintroduced later. - ❌ **Cephalexin 500 mg PO every 6 hours**: Oral therapy is insufficient for systemic infection; IV antibiotics are required. - ❌ **Diphenhydramine**: No role in cellulitis management unless for allergic reactions (not described here). - ❌ **Ibuprofen**: Avoid in acute kidney injury (creatinine 1.4 mg/dL). - ❌ **Nafcillin**: Lacks MRSA coverage, which is critical in diabetic patients. - ❌ **Tobramycin + piperacillin**: Tobramycin is nephrotoxic and contraindicated in renal impairment.   **Key Considerations:**   - **MRSA coverage** is essential given diabetes and healthcare exposure (post-CABG). - **Renal dose adjustments** for vancomycin are required (monitor trough levels). - **Monitor for necrotizing infection**: If clinical deterioration occurs (e.g., severe pain, hemodynamic instability), urgent MRI or surgical consult is needed.   **Final Selection**: Admit, elevate leg, blood cultures, vancomycin, and morphine. |
| Case 15 |  |  |  |
| Diff1 | **Most Likely Components of the Nervous System Involved:**   1. **Spinal Cord**   **Rationale:**  **Bilateral sensory loss from the waist to toes (dermatomal level) suggests a thoracic spinal cord lesion affecting sensory tracts (e.g., spinothalamic or dorsal columns).**  **Prior left leg numbness (2 years ago) and current perineal sensory changes align with disseminated lesions in time and space, classic for multiple sclerosis (MS).**  **No motor deficits or reflex abnormalities suggest a partial spinal cord lesion sparing motor pathways.**   1. **Peripheral Nerves (less likely but possible)**   **Rationale:**  **Subjective "funny" sensation in fingertips and perineum could suggest peripheral nerve involvement.**  **However, the lack of motor weakness, reflex changes, or stocking-glove distribution makes this less likely.**  **Why Not Other Options?**   - **Autonomic Ganglia: Normal bladder/bowel function and absence of autonomic symptoms (e.g., orthostatic hypotension) rule this out.** - **Cerebral Cortical Sensory Areas: Bilateral waist-down sensory loss is not consistent with cortical lesions, which typically cause contralateral focal deficits (e.g., hemisensory loss).** - **Midbrain: No cranial nerve deficits (e.g., diplopia, pupillary changes) or motor signs (e.g., rigidity, tremor) to suggest midbrain involvement.**   **Final Answer:**   - **Spinal Cord** - **Peripheral Nerves (less likely, but possible subtle involvement)**   **Clinical Correlation: The presentation strongly suggests multiple sclerosis with spinal cord demyelination. Recurrent episodes, young age, and lack of systemic/infectious features support this. MRI of the spine and brain is critical to confirm lesions.** | **Answer**: The components of the nervous system most likely involved in this patient’s symptoms are:   - **Spinal cord**   **Rationale**:   1. **Spinal Cord**:   **Key Findings**: Bilateral sensory loss from the waist down ("novocaine-like" sensation) with preserved motor function and reflexes strongly suggests a **spinal cord lesion**. The sensory level at the waist (thoracic dermatomes) and perineal involvement (S2-S4) localize to the spinal cord.  **Clinical Context**: The relapsing-remitting pattern (prior episode of left leg numbness) and absence of systemic symptoms align with **demyelination** (e.g., multiple sclerosis), which often affects the spinal cord.  **Exam Consistency**: Subjective sensory complaints with a normal sensory exam are common early in MS, as subtle lesions may not yet produce objective deficits.   1. **Peripheral Nerves**:   **Excluded**: Peripheral neuropathy typically presents with distal, symmetric "stocking-glove" sensory loss and reduced reflexes. This patient’s abrupt sensory level, preserved reflexes, and lack of weakness argue against peripheral nerve involvement.   1. **Cerebral Cortical Sensory Areas**:   **Excluded**: Cortical lesions (e.g., parietal lobe) would cause complex sensory deficits (e.g., agnosia, neglect) rather than a bilateral sensory level. The patient’s normal mental status and exam findings do not support cortical involvement.   1. **Midbrain**:   **Excluded**: Midbrain lesions would cause cranial nerve deficits (e.g., oculomotor abnormalities, ptosis) or motor/sensory dissociation (e.g., Weber syndrome), which are absent here.   1. **Autonomic Ganglia**:   **Excluded**: Normal bladder, bowel, and cardiovascular function rule out autonomic involvement.  **Conclusion**: The **spinal cord** is the primary site of pathology, likely due to a demyelinating lesion (e.g., MS). The relapsing history, sensory level, and lack of alternative explanations strongly support this localization. Further imaging (MRI spine) and CSF analysis are needed to confirm the diagnosis. | **Most Likely Components Involved**:   1. ✅ **Spinal Cord**   **Rationale**:  **Bilateral ascending sensory symptoms** (from waist to toes) suggest involvement of **sensory tracts in the spinal cord** (e.g., dorsal columns or spinothalamic tracts).  Prior episode of left leg numbness (resolved spontaneously) supports **relapsing-remitting demyelination** (e.g., multiple sclerosis affecting the spinal cord).  **Normal peripheral nerve exam** (intact reflexes, no weakness) argues against peripheral neuropathy.   1. ❌ **Peripheral Nerves**   **Why Unlikely**:  Peripheral neuropathy typically causes **distal, symmetric "glove-and-stocking" sensory loss**, not ascending/bilateral symptoms from the waist down.  Normal reflexes, strength, and sensory exam (light touch, pinprick, vibration) on testing make peripheral nerve involvement improbable.  **Why Other Options Are Unlikely:**   - ❌ **Autonomic Ganglia**:   Autonomic involvement would cause symptoms like orthostatic hypotension, urinary retention, or gastrointestinal dysmotility, which are absent here.   - ❌ **Cerebral Cortical Sensory Areas**:   Cortical sensory deficits (e.g., agraphesthesia, astereognosis) are not described. Symptoms are localized to the lower body, not a cortical distribution.   - ❌ **Midbrain**:   Midbrain lesions would cause cranial nerve deficits (e.g., pupillary abnormalities, oculomotor palsies) or motor/sensory deficits in the face/upper extremities, which are absent.  **Key Clinical Clues for Spinal Cord Involvement:**   - **Sensory level at the waist**: Suggests a **thoracic spinal cord lesion** affecting sensory tracts below T10. - **"Funny" perineal sensation**: May indicate **sacral spinal cord or conus medullaris involvement**. - **Relapsing-remitting history**: Classic for **demyelinating diseases like multiple sclerosis**, which frequently affects the spinal cord.   **Next Steps to Confirm:**   1. **MRI of the Spinal Cord**:   Look for **demyelinating lesions** (e.g., MS plaques) or other structural abnormalities (e.g., transverse myelitis).   1. **Lumbar Puncture**:   Check for **oligoclonal bands** or elevated IgG index to support MS diagnosis.   1. **Evoked Potentials**:   Assess for subclinical demyelination in other CNS regions.  **Final Answer**: **Spinal Cord**. |
| Diff2 | **Differential Diagnoses That Cannot Be Excluded: Based on the history and physical exam alone, the following conditions remain plausible and require further investigation:**   1. **Multiple Sclerosis (MS)**   **Key Support: Recurrent episodes of sensory deficits (current bilateral numbness, prior left leg numbness) with dissemination in time and space.**   1. **Acute Viral Infection**   **Consideration: Viral myelitis (e.g., post-infectious or subclinical viral trigger) despite no reported recent illness.**   1. **Connective Tissue Disorder**   **Example: Lupus-associated myelopathy or neuropsychiatric lupus, even in the absence of systemic symptoms.**   1. **Lumbosacral Plexitis**   **Rationale: Although bilateral sensory loss and perineal involvement are more typical of spinal cord pathology, plexitis can rarely present asymmetrically or bilaterally.**   1. **Myelopathy Due to Vitamin Deficiency**   **Example: B12 deficiency causing subacute combined degeneration, though no anemia or dietary risk is noted.**   1. **Peripheral Neuropathy Due to Toxins**   **Consideration: Insidious toxin exposure (e.g., heavy metals) could theoretically cause neuropathy, though no history of exposure is reported.**   1. **Sarcoidosis**   **Rationale: Neurosarcoidosis can present with isolated spinal cord involvement without systemic signs (e.g., lung or skin lesions).**   1. **Spinal Cord Compression by Tumor**   **Key Point: Slow-growing tumors (e.g., ependymoma) may lack pain or motor deficits early in their course.**   1. **Spinal Cord Hemorrhage**   **Consideration: Small hemorrhages (e.g., arteriovenous malformation) might cause subacute symptoms without acute pain.**   1. **Spinal Epidural Abscess**   **Rationale: Rarely presents without fever or back pain, especially in immunocompetent patients.**  **Why These Are Not Ruled Out:**   - **Imaging/Labs Not Done: MRI, CSF analysis, and bloodwork (e.g., B12, ANA, infectious serology) are pending.** - **Clinical Overlap: Many conditions share features of sensory myelopathy (e.g., MS, compression, infection).**   **Less Likely but Still Possible:**   - **Spinal Epidural Abscess and Hemorrhage are less probable due to the absence of fever, back pain, or acute deficits, but they cannot be definitively excluded without imaging.** - **Peripheral Neuropathy Due to Toxins is unlikely given the clinical pattern but remains a consideration until toxicology screening is performed.**   **Final Answer: All listed options cannot be excluded based on history and physical exam alone. Further diagnostic testing (e.g., MRI, labs) is required to narrow the differential.** | **Answer**: The following differential diagnoses **cannot be excluded** based on the current history and physical examination:   - **Multiple sclerosis (MS)** - **Myelopathy due to vitamin deficiency (e.g., B12)** - **Connective tissue disorder (e.g., lupus)** - **Sarcoidosis** - **Spinal cord compression by tumor**   **Rationale**:   1. **Multiple Sclerosis (MS)**:   Strongly suspected due to the relapsing-remitting pattern (prior left leg numbness and current episode) and sensory symptoms consistent with spinal cord demyelination. Requires MRI and CSF analysis to confirm.   1. **Myelopathy due to Vitamin Deficiency (e.g., B12)**:   Subacute combined degeneration from B12 deficiency can cause sensory deficits and spinal cord dysfunction. Early stages may lack anemia or macrocytosis. Serum B12 testing is needed to exclude.   1. **Connective Tissue Disorder (e.g., Lupus)**:   Autoimmune conditions like lupus can present with isolated neurological involvement (e.g., transverse myelitis). Absence of systemic symptoms (rash, arthritis) does not rule this out. Autoantibody testing (ANA, anti-dsDNA) is required.   1. **Sarcoidosis**:   Neurosarcoidosis can mimic MS with spinal cord lesions. Though systemic symptoms (e.g., lung involvement) are absent, isolated neurological sarcoidosis is possible. Imaging (MRI) and biopsy (if lesions are found) are needed.   1. **Spinal Cord Compression by Tumor**:   Slow-growing tumors (e.g., ependymoma, meningioma) may cause progressive sensory loss without pain or motor deficits. Normal spinal exam does not exclude this. MRI spine is necessary.  **Excluded or Less Likely Diagnoses**:   - **Acute Viral Infection**: No fever, recent illness, or acute onset makes viral myelitis less likely. - **Lumbosacral Plexitis**: Typically presents with pain, asymmetry, and weakness, which are absent here. - **Peripheral Neuropathy due to Toxins**: No toxin exposure history and non-"stocking-glove" sensory loss argue against this. - **Spinal Cord Hemorrhage**: Subacute progression and lack of trauma/pain make this unlikely. - **Spinal Epidural Abscess**: No fever, back pain, or risk factors (e.g., IV drug use) are noted.   **Next Steps**:   - **MRI of the Brain and Spinal Cord**: To identify demyelinating lesions, tumors, or structural abnormalities. - **Blood Tests**: B12, folate, ANA, ESR, ACE (for sarcoidosis), and infectious workup (e.g., Lyme serology). - **Lumbar Puncture**: For CSF analysis (oligoclonal bands, IgG index, cytology). - **Neurology Consultation**: For further evaluation and management.   **Key Takeaway**: While MS is the leading diagnosis, several mimics require exclusion through imaging and laboratory testing. | **Differential Diagnoses That Cannot Be Excluded**:   1. ✅ **Multiple Sclerosis (MS)**   **Rationale**: Relapsing-remitting sensory symptoms (current ascending numbness, prior left leg numbness) and normal exam are classic for early MS. Requires MRI/CSF for confirmation.   1. ✅ **Myelopathy due to Vitamin Deficiency (e.g., B12)**   **Rationale**: Subacute combined degeneration (B12 deficiency) can cause ascending sensory deficits. Normal diet does not rule out deficiency; serum B12 testing is needed.   1. ✅ **Spinal Cord Compression by Tumor**   **Rationale**: Slow-growing tumors (e.g., ependymoma) may present with insidious sensory deficits without pain or motor signs. MRI is required to exclude.   1. ✅ **Sarcoidosis**   **Rationale**: Neurosarcoidosis can mimic MS with spinal cord lesions. Lack of systemic symptoms (e.g., cough, rash) does not exclude isolated neurological involvement.   1. ✅ **Connective Tissue Disorder (e.g., SLE)**   **Rationale**: Autoimmune disorders like lupus can cause myelopathy or neuropathic symptoms. Serologic testing (e.g., ANA, anti-dsDNA) is needed.   1. ✅ **Spinal Cord Hemorrhage**   **Rationale**: Rare but possible if atypical presentation (e.g., slow hemorrhage). MRI can confirm/exclude.  **Why Other Options Are Less Likely (But Not Fully Excluded):**   - ❌ **Acute Viral Infection (e.g., ADEM, viral myelitis)**:   Unlikely due to **absence of fever, recent illness, or vaccination history**, but viral myelitis (e.g., enterovirus) could present without systemic symptoms.   - ❌ **Lumbosacral Plexitis**:   Typically causes **pain, weakness, or asymmetric symptoms**, which are absent here.   - ❌ **Peripheral Neuropathy due to Toxins**:   Ascending numbness from the waist is inconsistent with "glove-and-stocking" distribution. No toxin exposure reported, but heavy metals (e.g., lead) require screening.   - ❌ **Spinal Epidural Abscess**:   Unlikely without **fever, back pain, or tenderness**, but immunocompetent patients may lack systemic signs early.  **Key Next Steps to Exclude These Diagnoses:**   1. **MRI Spine/Brain**:   Rule out structural lesions (tumor, compression, hemorrhage, abscess) and identify demyelinating lesions (MS) or granulomas (sarcoidosis).   1. **Serum B12/Methylmalonic Acid**:   Exclude vitamin deficiency.   1. **Autoimmune/Inflammatory Markers**:   ANA, anti-dsDNA, ACE levels (sarcoidosis), aquaporin-4/MOG antibodies (NMOSD/MOGAD).   1. **CSF Analysis**:   Oligoclonal bands (MS), lymphocytosis (infection/autoimmunity), ACE levels (neurosarcoidosis).  **Final Answer**: **MS, Myelopathy due to vitamin deficiency, Spinal cord compression by tumor, Sarcoidosis, Connective tissue disorder, Spinal cord hemorrhage**. |
| Diag1 | **Most Appropriate Immediate Next Steps:**   1. **MRI of the Thoracic Spine**   **Rationale:**  **The patient has a sensory level at the waist (thoracic dermatomes) and perineal involvement, strongly suggesting a thoracic spinal cord lesion.**  **MRI is the gold standard to evaluate for myelopathy (e.g., demyelination in MS, tumor, abscess, or compression).**   1. **MRI of the Cervical Spine**   **Rationale:**  **Spinal cord lesions can extend across levels, and cervical involvement (e.g., in MS or compression) might explain subtle upper extremity symptoms (e.g., "funny" fingertip sensations).**  **Why Not Other Options?**   - **Carotid Doppler Ultrasonography: Irrelevant for spinal cord or peripheral nerve pathology; used for carotid stenosis/stroke evaluation.** - **CT of Chest/Abdomen: Less sensitive than MRI for spinal cord lesions. May be considered later if malignancy is suspected (e.g., paraneoplastic myelopathy), but not first-line.** - **Doppler Ultrasonography of the Legs: Unrelated to sensory myelopathy; useful for DVT or arterial insufficiency, which are not suggested here.** - **EMG of the Legs: Peripheral neuropathy is less likely given the dermatomal sensory loss (spinal cord pattern). EMG can wait until MRI rules out myelopathy.** - **X-ray of the Thoracic Spine: Low utility; cannot visualize soft tissue (e.g., spinal cord lesions, tumors).**   **Clinical Priorities:**   - **MRI of the Thoracic Spine is urgent to exclude compressive lesions (e.g., tumor, abscess) or demyelination (MS).** - **If thoracic MRI is unrevealing or if symptoms suggest higher involvement, MRI of the Cervical Spine should follow.** - **Bloodwork (e.g., B12, ANA, infectious serology) and lumbar puncture (if MRI suggests MS) are also critical but not listed as options.**   **Final Answer:**   - **MRI of the Thoracic Spine** - **MRI of the Cervical Spine** | **Answer**: The most appropriate immediate next steps are:   - **MRI of the Thoracic Spine** - **MRI of the Cervical Spine**   **Rationale**:   1. **MRI of the Thoracic Spine**:   **Critical Priority**: The patient’s sensory level at the waist (thoracic dermatomes) and perineal involvement (S2-S4) localize the lesion to the **thoracic spinal cord**. MRI is the gold standard for evaluating spinal cord pathology (e.g., demyelination, compression, tumors).  **Clinical Relevance**: Essential to confirm/exclude MS, spinal cord compression (e.g., tumor), or inflammatory lesions (e.g., transverse myelitis, sarcoidosis).   1. **MRI of the Cervical Spine**:   **Additional Imaging**: While the thoracic spine is the primary focus, **MS often involves multiple spinal cord levels**. Cervical spine MRI may reveal additional lesions, especially if symptoms suggest higher involvement (e.g., hand numbness, Lhermitte’s sign).  **Excluded Options**:   - **Carotid Doppler Ultrasonography**: Not relevant here. Used for carotid stenosis evaluation in stroke, which is unrelated to this patient’s spinal cord symptoms. - **CT of Chest/Abdomen**: Less sensitive than MRI for spinal cord lesions. May be considered later if malignancy or sarcoidosis is strongly suspected, but MRI spine is the immediate priority. - **Doppler Ultrasonography of the Legs**: No clinical suspicion for DVT (absence of leg swelling/pain). - **EMG of the Legs**: Peripheral neuropathy is unlikely given the sensory level and preserved reflexes/motor function. EMG is not urgent. - **X-ray of the Thoracic Spine**: Insufficient for evaluating spinal cord lesions (only assesses bony structures, not soft tissue).   **Next Steps After MRI**:   - If MRI confirms demyelination (e.g., MS), proceed with **brain MRI** and **lumbar puncture** for CSF analysis. - If MRI is negative, consider **vitamin B12/folate testing**, **autoimmune serology** (e.g., ANA, anti-dsDNA), and **sarcoidosis workup** (e.g., serum ACE, chest CT).   **Key Takeaway**: MRI of the **thoracic spine** is urgent to confirm spinal cord pathology. Adding **cervical spine MRI** ensures comprehensive evaluation for multifocal disease (e.g., MS). | **Most Appropriate Immediate Next Steps**:   1. ✅ **MRI of the Thoracic Spine**   **Rationale**:  The patient’s **ascending sensory symptoms from the waist down** strongly suggest a **thoracic spinal cord lesion** (e.g., demyelination, compression, or inflammation).  MRI is the **gold standard** for visualizing spinal cord pathology (e.g., MS plaques, tumors, or hemorrhage) and is critical to confirm/exclude myelopathy.  A sensory level at the waist localizes to the **thoracic cord (T10 level)**, making this the highest-yield imaging target.   1. ✅ **MRI of the Cervical Spine**   **Rationale**:  While symptoms suggest thoracic involvement, **cervical spinal cord lesions** can sometimes manifest with lower extremity symptoms due to descending/ascending tract involvement.  In **demyelinating diseases like MS**, multiple spinal lesions may coexist, and imaging the entire spine (cervical + thoracic) is often standard to assess disease burden.  **Why Other Options Are Less Appropriate:**   - ❌ **X-ray of the Thoracic Spine**:   X-rays lack sensitivity for spinal cord pathology (e.g., demyelination, tumors) and are only useful for evaluating **bony abnormalities** (e.g., fractures, spondylosis), which are not suspected here.   - ❌ **CT of Chest/Abdomen**:   CT is less sensitive than MRI for spinal cord lesions. It may be used to screen for **paraneoplastic/malignancy-related compression**, but MRI of the spine is the priority.   - ❌ **EMG of the Legs**:   EMG evaluates **peripheral nerves or muscles**, which are unlikely given the normal reflexes, lack of weakness, and ascending (not distal) sensory symptoms.   - ❌ **Carotid/Leg Doppler Ultrasonography**:   Carotid Doppler assesses for stroke risk, and leg Doppler evaluates for DVT or arterial insufficiency—neither is relevant to this patient’s myelopathic presentation.  **Key Considerations:**   - **MRI of the thoracic spine** is the **highest-priority test** to identify lesions (e.g., MS plaques, tumors, or transverse myelitis). - **Adding cervical spine MRI** increases sensitivity for multifocal disease (e.g., MS) and is often performed concurrently in clinical practice. - **Labs for vitamin B12, inflammatory markers, and CSF analysis** (via lumbar puncture) should follow MRI to further narrow the differential (e.g., B12 deficiency, MS, sarcoidosis).   **Final Answer**: **MRI of the Thoracic Spine** and **MRI of the Cervical Spine**. |
| Diag2 | **Most Appropriate Steps:**   1. **MRI of the Brain**   **Rationale:**  **Transverse myelitis can be associated with demyelinating diseases like multiple sclerosis (MS) or neuromyelitis optica spectrum disorder (NMOSD).**  **Brain MRI identifies lesions suggestive of MS (e.g., periventricular or cortical lesions) or NMOSD (e.g., brainstem lesions).**   1. **Spinal Tap (Lumbar Puncture)**   **Rationale:**  **CSF analysis is critical to:** Essential to evaluate for:  **Inflammation** (elevated white blood cells, protein).  **Oligoclonal bands** (supportive of MS).  **Infectious Causes** (e.g., viral PCRs for HSV, VZV, enterovirus).  **Autoantibodies** (e.g., aquaporin-4 IgG for NMOSD, MOG antibody).  **Why Other Options Are Less Appropriate:**   - **CT of the Spine:**   **Already superseded by MRI, which is superior for evaluating spinal cord inflammation. CT lacks sensitivity for soft-tissue lesions.**   - **Doppler Studies of the Legs:**   **Relevant only if DVT is suspected (e.g., immobility-related complications), not for diagnosing myelitis.**   - **EEG:**   **Used to evaluate seizures or encephalopathy, which are not typical in isolated transverse myelitis.**   - **Nerve Conduction Studies/EMG:**   **Assess peripheral nerves/muscles, not central spinal cord pathology. Reserved if peripheral neuropathy is suspected (e.g., overlapping symptoms).**  **Clinical Priorities:**   - **Urgent CSF Analysis: Guides treatment (e.g., immunosuppression for autoimmune causes, antivirals for infections).** - **Brain MRI: Determines if myelitis is part of a broader demyelinating process (e.g., MS, NMOSD).** - **Initiate IV Steroids: While not listed here, methylprednisolone is first-line therapy while awaiting test results.**   **Final Answer:**   - **MRI of the Brain** - **Spinal Tap (Lumbar Puncture)**   - - - **Detect oligolls, protei** | **Answer**: The most appropriate immediate next steps are:   - **MRI of the Brain** - **Spinal Tap (Lumbar Puncture)**   **Rationale**:   1. **MRI of the Brain**:   **Critical for Demyelinating Diseases**: Transverse myelitis (TM) can be a manifestation of **multiple sclerosis (MS)** or **neuromyelitis optica spectrum disorder (NMOSD)**. Brain MRI identifies demyelinating lesions (e.g., periventricular or cortical lesions in MS, or optic nerve involvement in NMOSD).  **Guides Diagnosis**: Findings differentiate MS (brain lesions) from NMOSD (often brain-sparing) or isolated TM.   1. **Spinal Tap (Lumbar Puncture)**:   **CSF Analysis**: Essential to evaluate for:  **Inflammation** (elevated white blood cells, protein).  **Oligoclonal bands** (supportive of MS).  **Infectious Causes** (e.g., viral PCRs for HSV, VZV, enterovirus).  **Autoantibodies** (e.g., aquaporin-4 IgG for NMOSD, MOG antibody).  **Excluded Options**:   - **CT of the Spine**: Already diagnosed TM via MRI; CT lacks sensitivity for spinal cord pathology and adds no additional value. - **Doppler Studies of the Legs**: No clinical suspicion for DVT (no leg swelling/pain). - **EEG**: Unrelated to spinal cord pathology unless seizures/encephalopathy are present (not mentioned here). - **Nerve Conduction Studies/EMG**: Symptoms localize to the spinal cord (sensory level at the waist), not peripheral nerves. EMG is unnecessary unless peripheral neuropathy is suspected (e.g., "stocking-glove" sensory - **Additional Considerations**: - **Autoimmune/Antibody Testing**:**Aquaporin-4 IgG** (NMOSD).   **MOG antibody** (MOG-associated disease).  **ANA, anti-dsDNA, ANCA** (systemic autoimmune disorders).   - **Vitamin B12/Folate Levels**: Exclude metabolic mimics (e.g., subacute combined degeneration)   **Key Takeaway**: **Brain MRI** and **lumbar puncture** are critical to identify the underlying cause of transverse myelitis (e.g., MS, NMOSD, infection) and guide immunosuppressive therapy (e.g., steroids, plasma exchange) or disease-modifying agents. | **Most Appropriate Next Steps**:   1. ✅ **MRI of the Brain**   **Rationale**:  Transverse myelitis (TM) is often associated with **demyelinating diseases** (e.g., multiple sclerosis MS*MS* or neuromyelitis optica spectrum disorder NMOSD*NMOSD*).  **Brain MRI** can identify lesions suggestive of MS (e.g., periventricular/juxtacortical lesions) or NMOSD (e.g., optic nerve, hypothalamus, or area postrema lesions).  Guides differentiation between isolated TM vs. TM as part of a broader autoimmune/inflammatory disorder.   1. ✅ **Spinal Tap (Lumbar Puncture)**   **Rationale**:  **CSF analysis** is critical  **Why Other Options Are Less Appropriate:**   - ❌ **CT of the Spine**:   Already superseded by MRI, which is far superior for evaluating spinal cord inflammation, demyelination, or structural lesions. CT lacks sensitivity for TM.   - ❌ **Doppler Studies of the Legs**:   Irrelevant unless there is clinical suspicion for vascular compromise (e.g., DVT), which is unrelated to TM.   - ❌ **EEG**:   Evaluates seizure activity or encephalopathy, which are not part of TM’s presentation (unless coexisting CNS involvement is suspected, but brain MRI would be more informative).   - ❌ **Nerve Conduction Studies/EMG**:   Assesses peripheral nerve/muscle dysfunction, not spinal cord pathology. TM primarily involves the central nervous system.  **Additional Considerations:**   - **Blood Tests**:   Order **AQP4-IgG**, **MOG-IgG**, vitamin B12/copper levels, inflammatory markers (ESR/CRP), and autoimmune panels (e.g., ANA, anti-dsDNA) to identify underlying causes.   - **Acute Treatment**:Start **high-dose IV steroids** (methylprednisolone) immediately to reduce inflammation. Consider plasma exchange if no response to steroids.   **Final Answer**: **MRI of the Brain** and **Spinal Tap (Lumbar Puncture)**. |
| dx | **Most Likely Diagnosis:**  **Multiple Sclerosis (MS)**  **Key Supporting Evidence:**   1. **Brain MRI Showing Primary Demyelinating Disease:**   **MS is characterized by dissemination in space and time on MRI, with typical lesions in periventricular, cortical/juxtacortical, infratentorial, or spinal cord regions.**  **Acute demyelinating lesions often enhance with contrast during active inflammation.**   1. **Clinical Context:**   **Transverse myelitis (mentioned earlier) is a common presentation of MS.**  **Primary demyelination on MRI strongly aligns with MS over other options.**  **Why Other Diagnoses Are Less Likely:**   1. **Acute Disseminated Encephalomyelitis (ADEM):**   **Typically occurs post-infectious/post-vaccination, with multifocal neurologic deficits (e.g., encephalopathy, seizures).**  **More common in children and usually monophasic (no relapses).**   1. **Infectious Meningoencephalomyelitis:**   **Requires evidence of infection (e.g., fever, CSF with neutrophilic pleocytosis, positive PCR/cultures for pathogens like HSV, enteroviruses).**  **No mention of infectious symptoms or CSF findings supporting this.**   1. **Sarcoidosis:**   **Neurosarcoidosis often presents with leptomeningeal enhancement, hypothalamic/pituitary lesions, or cranial neuropathies.**  **Systemic involvement (e.g., lung disease, elevated ACE levels) is common but not described here.**  **Critical Next Steps to Confirm MS:**   1. **Confirm Dissemination in Time:**   **Compare current MRI with prior imaging (if available) to demonstrate new lesions over time.**   1. **CSF Analysis:**   **Oligoclonal bands (present in >85% of MS cases) support the diagnosis.**  **Elevated IgG index.**   1. **Exclude NMOSD/MOGAD:**   **Ensure aquaporin-4 IgG and MOG antibody testing is negative (these conditions mimic MS but require distinct treatments).**  **Final Answer: Multiple sclerosis is the most likely diagnosis based on the clinical and imaging findings.** | **Answer**: The most likely diagnosis is **Multiple Sclerosis (MS)**.  **Rationale**:   1. **Clinical and Radiologic Alignment**:   **Primary demyelinating disease** on brain MRI is a hallmark of MS. Typical MS lesions include periventricular, cortical/juxtacortical, or infratentorial lesions.  Transverse myelitis (spinal cord inflammation) is a common initial presentation of MS (clinically isolated syndrome, CIS).   1. **Differentiation from Other Options**:   **Acute Disseminated Encephalomyelitis (ADEM)**:  Typically occurs **post-infection/vaccination**, is **monophasic**, and more common in children.  Brain lesions in ADEM are often **widespread, ill-defined, and asymmetric**, unlike the discrete lesions of MS.  **Infectious Meningoencephalomyelitis**:  Unlikely without systemic signs of infection (e.g., fever, CSF pleocytosis with neutrophils, positive viral/bacterial PCR/cultures).   - - **Sarcoidosis**:   Neurosarcoidosis can mimic MS but often involves **leptomeningeal enhancement**, cranial neuropathies, or systemic findings (e.g., lung/lymph node involvement).   1. **Supporting Evidence**:   **CSF Findings**: Oligoclonal bands (OCBs) in the CSF strongly support MS (present in >90% of MS cases).  **Antibody Testing**: Negative aquaporin-4 IgG/MOG antibodies help exclude NMOSD/MOGAD.  **Key Takeaway**: The combination of **brain MRI showing demyelination** and **transverse myelitis** is highly suggestive of MS, especially if CSF shows OCBs. NMOSD or MOGAD remain possible if antibody testing is pending, but MS is the most likely diagnosis based on typical imaging and clinical context. | **Most Likely Diagnosis**: **Multiple Sclerosis (MS)**  **Key Reasoning:**   1. **MRI Findings**:   **Primary demyelinating disease** on brain MRI aligns with MS, which classically shows **disseminated white matter lesions** (e.g., periventricular, juxtacortical, infratentorial).  Transverse myelitis (as previously discussed) can be the **first presentation** of MS (clinically isolated syndrome CIS*CIS*), especially if brain lesions are already present.   1. **CSF Findings** (from prior spinal tap):   **Oligoclonal bands** (present in >90% of MS cases) strongly support MS over other diagnoses.  Elevated IgG index further corroborates MS.   1. **Exclusion of Alternatives**:   **ADEM**: Typically monophasic, occurs post-infection/vaccination, and involves **encephalopathy** (not mentioned here). Less likely in adults without prior triggers.  **Infectious Meningoencephalomyelitis**: CSF would show pleocytosis/pathogens (likely ruled out by prior spinal tap).  **Sarcoidosis**: Requires systemic involvement (e.g., lung/lymph node disease) or CSF findings like elevated ACE/lymphocytosis. Less likely without such features.  **Supporting Features of MS:**   - **Dissemination in Space/Time**:   Brain MRI lesions in multiple regions (e.g., periventricular, cortical/juxtacortical) + prior/new symptoms/lesions.   - **Typical Demographics**:   Most common in young adults (20-40 years), though age is unspecified here.  **Why Not Other Options?:**   - ❌ **ADEM**: Absence of encephalopathy, prior infection/vaccination, or pediatric age makes this less likely. - ❌ **Infectious Meningoencephalomyelitis**: No evidence of CSF pleocytosis or pathogens. - ❌ **Sarcoidosis**: No systemic symptoms (e.g., fever, cough, uveitis) or suggestive CSF/biomarkers (e.g., ACE).   **Final Answer**: **Multiple Sclerosis (MS)** is the most likely diagnosis. |
| mang | **Initial Therapies for Confirmed Multiple Sclerosis (MS):**  **The following options are appropriate first-line disease-modifying therapies (DMTs) for MS based on current guidelines:**  **1. Dimethyl fumarate 120 mg PO twice daily for 7 days**   - **Rationale: A standard oral DMT. The 120 mg twice-daily dose is the initial escalation regimen (to reduce side effects like flushing) before increasing to the maintenance dose of 240 mg twice daily. Effective for relapsing-remitting MS (RRMS).**   **2. Interferon beta-1a, 30 mcg IM once weekly**   - **Rationale: A first-line injectable therapy (e.g., Avonex). Reduces relapse rates and MRI lesion activity in RRMS.**   **Additional Considerations:**   - **Natalizumab 300 mg IV every 4 weeks:**   **Reserved for highly active MS (e.g., rapid disease progression, ≥2 relapses in a year) or patients refractory to first-line therapies. Not typically "initial" unless aggressive disease is evident.**  **Requires John Cunningham virus (JCV) antibody testing due to PML risk.**  **Excluded Options:**   - **Azathioprine/Cyclophosphamide: Not first-line DMTs. Used off-label or in resource-limited settings.** - **IVIG: Reserved for特殊情况 (e.g., pregnancy, intolerance to other DMTs).** - **Mitoxantrone: Used in aggressive/progressive MS but carries significant toxicity (e.g., cardiotoxicity).** - **Methylprednisolone: Acute relapse treatment, not a long-term DMT.**   **Final Answer: Dimethyl fumarate and interferon beta-1a are appropriate initial therapies.**   - **Natalizumab may be considered in highly active disease but is not universally first-line.** - **Other options (azathioprine, cyclophosphamide, IVIG, mitoxantrone) are not standard initial choices.** - **Methylprednisolone is for acute relapses, not maintenance therapy.** | **Answer**: The following can be considered initial therapies for this patient:   - **Dimethyl fumarate 120 mg po bid for 7 days** - **Interferon beta-1a, 30 mcg IM once/wk** - **Methylprednisolone 1 g IV once per day for 3 d** - **Natalizumab 300 mg IV q 4 wk**   **Rationale**:   1. **Methylprednisolone (1 g IV for 3 days)**:   **Acute Relapse Management**: High-dose IV steroids are standard for acute exacerbations to reduce inflammation and accelerate recovery.   1. **Disease-Modifying Therapies (DMTs)**:   **Dimethyl Fumarate**:  **First-line oral DMT**: Reduces relapse rates by modulating immune responses. The dosing schedule (120 mg bid for 7 days followed by 240 mg bid) is typical for initiation.  **Interferon Beta-1a**:  **First-line injectable DMT**: Decreases relapse frequency and MRI lesion activity. Weekly IM injections are a standard regimen.  **Natalizumab**:  **High-efficacy DMT**: Used for highly active MS (e.g., rapid progression, frequent relapses). Requires **JC virus antibody testing** due to PML risk, but it can be first-line in JC virus-negative patients.   1. **Excluded Options**:   **Azathioprine/Cyclophosphamide/Mitoxantrone**: Not first-line due to toxicity or lack of robust evidence for MS.  **IVIG**: Reserved for refractory cases or pregnancy, not standard initial therapy.  **Key Takeaway**: For confirmed MS, **acute relapse treatment (steroids)** and **initiation of a DMT** are critical. First-line DMTs include **dimethyl fumarate**, **interferon beta-1a**, and **natalizumab** (in select high-risk patients). Choice depends on disease activity, patient preference, and risk factors (e.g., JC virus status for natalizumab). | **Initial Therapies for Multiple Sclerosis (MS):**  **Appropriate Options:**   1. **Dimethyl fumarate 120 mg PO BID for 7 days** (escalating to 240 mg BID):   First-line oral disease-modifying therapy (DMT) for relapsing-remitting MS (RRMS). Reduces relapse rates by ~50%.   1. **Interferon beta-1a, 30 mcg IM once/week**:   First-line injectable DMT for RRMS. Reduces relapse rates by ~30% and slows disability progression.   1. **Natalizumab 300 mg IV every 4 weeks**:   High-efficacy DMT for RRMS, used in patients with **high disease activity** (e.g., frequent relapses, active MRI lesions). Requires **JCV antibody testing** due to PML risk.   1. **Methylprednisolone 1 g IV once/day for 3 days**:   Standard acute therapy for MS relapses to reduce inflammation and accelerate recovery.  **Why Other Options Are Inappropriate:**   - ❌ **Azathioprine**: Not FDA-approved for MS; limited evidence and higher risk of side effects. - ❌ **Cyclophosphamide**: Reserved for refractory/aggressive MS (e.g., rapidly progressive cases). - ❌ **IVIG**: Used in specific scenarios (e.g., pregnancy, intolerance to DMTs) but not first-line. - ❌ **Mitoxantrone**: Reserved for advanced/aggressive MS due to cardiotoxicity and leukemia risk.   **Final Answer**: The most appropriate initial therapies are **dimethyl fumarate**, **interferon beta-1a**, **natalizumab** (for high-efficacy needs), and **methylprednisolone** (for acute relapse management). |
| Case 16 |  |  |  |
| Diff | **Essential Differential Diagnoses for the Patient's Current Condition:**  **The patient’s recurrent lethargy, bradycardia (HR 30), hypotension (BP 74/52), and respiratory depression (RR 10) after a suicide attempt involving multiple medications necessitate consideration of the following key diagnoses:**  **1. Beta-Blocker Overdose**   - **Rationale: Beta-blockers cause profound bradycardia and hypotension. The patient’s history of hypertension (on three antihypertensives) raises suspicion for beta-blocker use (e.g., metoprolol).**   **2. Calcium Channel Blocker (CCB) Overdose**   - **Rationale: Non-dihydropyridine CCBs (e.g., verapamil, diltiazem) also cause bradycardia, hypotension, and shock. Overdose mimics beta-blocker toxicity and is common in patients on antihypertensives.**   **3. Opioid Overdose (Recurrent)**   - **Rationale: The patient initially improved with naloxone, but its short half-life (30–90 minutes) allows recurrent opioid toxicity (e.g., respiratory depression, lethargy). Oxycodone overdose is confirmed by the empty pill bottle.**   **4. Hypoglycemia**   - **Rationale: Hypoglycemia can cause altered mental status, bradycardia, and hypotension. Immediate glucose testing is critical, though less likely in this overdose context.**   **5. Tricyclic Antidepressant (TCA) Overdose**   - **Rationale: TCAs cause sodium channel blockade (QRS widening on ECG), seizures, and arrhythmias. Though the patient lacks classic anticholinergic signs (e.g., dilated pupils), his depression history raises concern for TCA use.**   **Less Likely but Possible Considerations:**   - **Digoxin Overdose: Unlikely without a history of heart failure/arrhythmias or hyperkalemia/nausea.** - **Adrenal Insufficiency/Hypothyroidism: Chronic conditions with slower onset; less likely in this acute overdose setting.** - **Elevated Intracranial Pressure (ICP): No Cushing’s triad (hypertension, bradycardia, irregular respirations) or pupillary abnormalities.** - **Myocardial Infarction (MI)/Pulmonary Embolism (PE): MI may cause bradycardia (e.g., inferior MI), but no chest pain or ECG changes noted. PE typically presents with tachycardia, hypoxia, and hypotension.**   **Final Answer: The essential differential diagnoses are: Beta-blocker overdose, Calcium channel blocker overdose, Opioid overdose, Hypoglycemia, and Tricyclic antidepressant (TCA) overdose.** | **Essential Differential Diagnoses**:   1. **Beta-blocker overdose** 2. **Calcium channel blocker (CCB) overdose** 3. **Recurrent opioid overdose** 4. **Digoxin overdose** 5. **Hypoglycemia**   **Rationale**:   - **Beta-blocker/CCB Overdose**:   **Severe bradycardia (HR 30)** and **hypotension (BP 74/52 mmHg)** are hallmark findings. Beta-blockers and CCBs (e.g., verapamil, diltiazem) impair cardiac conduction and vascular tone, which aligns with the patient’s presentation.  The patient’s history of hypertension (on three antihypertensives) increases suspicion for these overdoses.   - **Recurrent Opioid Overdose**:   Initial improvement with naloxone (opioid antagonist) followed by **relapsing lethargy/respiratory depression (10/min)** suggests naloxone’s shorter half-life compared to opioids (e.g., oxycodone).   - **Digoxin Overdose**:   Causes **profound bradycardia** and hypotension. Though not explicitly listed in his medications, digoxin toxicity should be considered in unexplained bradyarrhythmia.  Associated findings (e.g., hyperkalemia, nausea) are absent, but serum digoxin levels/ECG (check for heart block) are critical.   - **Hypoglycemia**:   A rapid, reversible cause of altered mental status. Requires immediate fingerstick glucose testing.  **Excluded/Unlikely Diagnoses**:   - **TCA Overdose**: Pupils are **reactive** (TCA overdose causes dilated pupils), and there’s no QRS widening or anticholinergic signs. - **Adrenal Insufficiency/Hypothyroidism**: Unlikely without chronic stigmata (e.g., hyperpigmentation, cold intolerance) and given the acute overdose context. - **Elevated ICP/MI/PE**: No focal neurologic deficits, chest pain, or hypoxia (pulse ox 94% rules out significant PE).   **Immediate Actions**:   1. **Repeat naloxone** (0.4–2 mg IV) and start an infusion to counteract recurrent opioid effects. 2. **Antidotes for Beta-blocker/CCB Overdose**:   **Calcium gluconate (1–3 g IV)** for CCB toxicity.  **Glucagon (3–10 mg IV bolus)** for beta-blocker toxicity.   1. **Atropine (1 mg IV)** and **vasopressors (e.g., norepinephrine)** for refractory bradycardia/hypotension. 2. **Check glucose**, ECG, and toxicology screen.   **Key Takeaway**: In a suicidal overdose with **bradycardia + hypotension**, prioritize **beta-blocker/CCB toxicity** and **recurrent opioid effects**. Always rule out hypoglycemia and consider digoxin if arrhythmias are present. | **Essential Differential Diagnoses in This Case:** The patient’s clinical deterioration (profound bradycardia, hypotension, respiratory depression) following a mixed overdose necessitates consideration of the following key diagnoses:   1. **Beta-Blocker Overdose**   **Rationale:** Bradycardia and hypotension are hallmark features of beta-blocker toxicity. The patient’s hypertension regimen likely includes a beta-blocker (e.g., metoprolol), and overdose would suppress cardiac output and chronotropy.   1. **Calcium Channel Blocker (CCB) Overdose**   **Rationale:** Non-dihydropyridine CCBs (e.g., diltiazem, verapamil) cause bradycardia, hypotension, and myocardial depression. This aligns with the patient’s vital signs and history of hypertension.   1. **Opioid Overdose (Recurrence)**   **Rationale:** Initial response to naloxone suggests opioid involvement. However, naloxone’s short half-life (30–90 minutes) may allow resedation if a long-acting opioid (e.g., extended-release oxycodone) was ingested. Respiratory depression (RR 10/min) supports this.   1. **Digoxin Overdose**   **Rationale:** Digoxin toxicity can cause profound bradycardia and hypotension. While less likely without a history of heart failure/arrhythmias, accidental ingestion (e.g., mistaken for another medication) should be considered.  **Less Likely but Worth Considering:**   - **Tricyclic Antidepressant (TCA) Overdose:** Unlikely due to the absence of anticholinergic signs (e.g., dilated pupils, tachycardia) or ECG changes (QRS widening). - **Hypoglycemia:** Rapidly excluded with a bedside glucose check. Bradycardia is atypical. - **Myocardial Infarction (MI):** Possible but lacks typical symptoms (e.g., chest pain) or ECG findings. - **Elevated Intracranial Pressure (ICP):** Inconsistent with normal pupillary reactivity and absence of hypertension (Cushing’s triad requires elevated BP).   **Immediate Next Steps:**   - **Repeat Naloxone** (0.4–2 mg IV) and start a **naloxone infusion** to counteract recurrent opioid effects. - **ECG** to assess for heart block (beta-blocker/CCB), hyperkalemia (digoxin), or QRS widening (TCA). - **Bedside Glucose** to rule out hypoglycemia. - **IV Calcium** (for CCB toxicity) and **Glucagon** (for beta-blocker toxicity) as antidotes. - **Toxicology Consultation** for guidance on specific antidotes (e.g., digoxin immune fab if digoxin suspected).   **Conclusion:** The most critical differentials are **beta-blocker overdose**, **calcium channel blocker overdose**, **recurrent opioid toxicity**, and **digoxin overdose**. These require urgent intervention to reverse cardiotoxicity and respiratory depression. |
| diag | **Essential Immediate Steps:**   1. **2 L 0.9% Normal Saline IV Bolus:**   **Rationale: Address hypotension and potential hypovolemia. Fluid resuscitation is critical in shock, even if the cause is cardiogenic (e.g., beta-blocker/CCB overdose), as initial stabilization.**   1. **Naloxone 0.4 mg IV and Monitoring for Improvement:**   **Rationale: Recurrent opioid toxicity is likely due to naloxone’s short half-life. Redosing naloxone can reverse recurrent respiratory depression (RR 10/min) and lethargy.**   1. **Cardiac Monitoring:**   **Rationale: Severe bradycardia (HR 30) and hypotension require continuous telemetry to detect arrhythmias (e.g., heart block from CCB/beta-blocker toxicity).**   1. **Bedside Glucose Level:**   **Rationale: Hypoglycemia can mimic or exacerbate altered mental status and bradycardia. Rapid glucose testing is critical for immediate correction if low.**   1. **Serum Acetaminophen Level and Serum Salicylate Levels:**   **Rationale: Co-ingestion of acetaminophen or salicylates is common in suicide attempts. Both require specific, time-sensitive treatments (e.g., NAC for acetaminophen, alkalinization for salicylates).**   1. **Serum Electrolyte Concentrations:**   **Rationale: Electrolyte imbalances (e.g., hyperkalemia in digoxin overdose, hypocalcemia in CCB overdose) can worsen cardiovascular instability.**  **Less Immediate but Still Important Steps:**   - **Urine Toxicology Screening: Useful for identifying co-ingested substances but does not guide immediate management.** - **Chest X-ray: Indicated if aspiration or pulmonary edema is suspected (e.g., hypoxia, respiratory distress), but the patient’s SpO₂ is stable at 94%.** - **CBC: May help assess for infection or anemia but is not urgent in this overdose context.** - **Blood Cultures: Unnecessary unless sepsis is suspected (no fever or focal infection signs).**   **Non-Essential in the Acute Setting:**   - **Serum Digoxin Level: Unlikely unless digoxin use is confirmed (no history of heart failure/arrhythmias).** - **Serum TSH: Hypothyroidism is a chronic cause of bradycardia, not an acute priority here.** - **Myocardial Infarction/Pulmonary Embolism Workup: No chest pain, ECG changes, or hypoxia to suggest MI/PE.**   **Final Answer: The essential immediate steps are: 2 L 0.9% normal saline IV bolus, Naloxone 0.4 mg IV, Cardiac monitoring, Bedside glucose level, Serum acetaminophen level, Serum salicylate levels, and Serum electrolyte concentrations.** | **Essential Immediate Steps**:   1. **Naloxone 0.4 mg IV and monitoring for improvement in symptoms**:   Critical for **recurrent opioid toxicity** (naloxone’s effect wears off faster than opioids). Repeat doses or a continuous infusion may be needed.   1. **Cardiac monitoring**:   Vital due to **severe bradycardia (HR 30)** and hypotension. Beta-blocker/CCB overdose can cause lethal arrhythmias or heart block.   1. **Bedside glucose level**:   Rapidly rule out **hypoglycemia**, a reversible cause of altered mental status.   1. **2 L 0.9% normal saline IV bolus**:   Address hypotension (BP 74/52), though use cautiously if cardiogenic shock is suspected (e.g., beta-blocker/CCB toxicity).   1. **Serum electrolyte concentrations**:   Check for **hypokalemia/hyperkalemia**, which exacerbate cardiac instability (especially with digoxin/CCB overdose).   1. **Serum acetaminophen level**:   **Mandatory** in unknown overdoses, as acetaminophen toxicity is common, insidious, and treatable with *N-acetylcysteine*.   1. **Urine toxicology screening**:   Identifies co-ingestants (e.g., beta-blockers, CCBs, TCAs) to guide antidotes, though results may not be immediate.  **Excluded/Nonurgent Steps**:   - **Blood cultures/CBC/chest x-ray**: No signs of infection or pulmonary pathology. - **Serum digoxin/salicylate levels/TSH**: Less likely in this context (no history of digoxin use, no salicylate symptoms, acute vs. chronic hypothyroidism).   **Rationale**:   - Prioritize interventions targeting **opioid reversal**, **cardiovascular stabilization**, and **reversible metabolic causes** (e.g., hypoglycemia). - Acetaminophen and toxicology screens are critical for hidden ingestions. - Avoid delays with nonurgent tests (e.g., TSH, cultures) until initial stabilization.   **Key Takeaway**: Immediate management focuses on **naloxone**, **fluid resuscitation**, **cardiac monitoring**, and **rapid metabolic checks** (glucose, electrolytes), while toxicology workup (acetaminophen, urine screen) guides long-term care. | **Essential Immediate Steps:** The patient’s critical condition (bradycardia, hypotension, respiratory depression) requires urgent interventions to stabilize and identify life-threatening toxicities. The following steps are **immediately essential**:   1. **Naloxone 0.4 mg IV and monitoring for improvement in symptoms**   **Rationale:** The patient initially improved with naloxone, suggesting opioid involvement. However, naloxone’s short duration (30–90 minutes) may allow resedation if a long-acting opioid (e.g., extended-release oxycodone) was ingested. Repeat dosing or a continuous infusion may be necessary.   1. **Cardiac monitoring**   **Rationale:** Profound bradycardia (HR 30) and hypotension (BP 74/52) raise concern for **beta-blocker or calcium channel blocker toxicity**, which can progress to heart block or asystole. Continuous ECG monitoring is critical to detect arrhythmias.   1. **Bedside glucose level**   **Rationale:** Hypoglycemia can mimic or exacerbate altered mental status. Rapid glucose testing is essential to rule out this reversible cause.   1. **2 L 0.9% normal saline IV bolus**   **Rationale:** Hypotension requires fluid resuscitation, though caution is needed if cardiac dysfunction (e.g., calcium channel blocker overdose) is suspected. Fluids may improve preload and cardiac output.   1. **Serum electrolyte concentrations**   **Rationale:** Electrolyte imbalances (e.g., hyperkalemia from digoxin toxicity, hypocalcemia from calcium channel blocker overdose) can worsen cardiac instability. Immediate correction (e.g., IV calcium for hypocalcemia) may be lifesaving.   1. **Serum acetaminophen level**   **Rationale:** Acetaminophen overdose is common in suicide attempts and can be asymptomatic early. If elevated, **N-acetylcysteine (NAC)** must be started promptly to prevent liver failure.   1. **Serum digoxin level**   **Rationale:** Though not explicitly mentioned in his history, digoxin toxicity can cause profound bradycardia and hypotension. If confirmed, **digoxin immune fab** is the antidote.  **Lower-Priority Steps in This Acute Setting:**   - **Blood cultures/CBC/TSH/chest x-ray:** Not immediately relevant to acute overdose management. - **Urine toxicology screening:** Results are delayed and unlikely to change acute management. - **Serum salicylate levels:** The patient lacks classic signs (tachycardia, hyperventilation, tinnitus).   **Conclusion:** Prioritize **naloxone**, **fluid resuscitation**, **cardiac monitoring**, **glucose check**, **electrolytes**, **acetaminophen level**, and **digoxin level**. These steps address the most likely and life-threatening toxicities (opioids, beta-blockers, calcium channel blockers, digoxin) and guide antidote use (naloxone, calcium, glucagon, digoxin fab). |
| dx | **Answer: Calcium channel blocker overdose**  **Key Supporting Evidence:**   1. **Persistent Bradycardia (HR 30) and Hypotension (76/54):**   **Unresponsive to IV fluids and naloxone, ruling out hypovolemia and pure opioid toxicity.**  **Classic for non-dihydropyridine CCB overdose (e.g., verapamil, diltiazem), which depresses cardiac conduction and vascular tone.**   1. **2nd-Degree (Type I) AV Block on ECG:**   **CCBs directly inhibit AV node conduction, leading to progressive heart block.**   1. **Hypoglycemia (Glucose 68 mg/dL):**   **Some CCBs (e.g., verapamil) impair insulin secretion, contributing to hypoglycemia.**   1. **Absence of Other Toxins:**   **Acetaminophen, salicylates, digoxin, and opioids (reversed by naloxone) are ruled out.**  **Why Not Other Options?:**   - **Beta-blocker overdose: Can cause bradycardia/hypotension but typically lacks hypoglycemia.** - **Digoxin overdose: Ruled out by undetectable digoxin level.** - **Opioid overdose: Reversed by naloxone; no improvement here.** - **Hypocalcemia/Hypokalemia/Hypothyroidism: Normal electrolytes and TSH.** - **Marijuana: Does not cause life-threatening bradycardia/hypotension.**   **Next Steps:**   - **Administer calcium gluconate (1-3 g IV) to improve cardiac contractility.** - **Consider high-dose insulin euglycemic therapy (HIET) for refractory shock.** - **Prepare for vasopressors (e.g., norepinephrine) if hypotension persists.** | **Most Likely Diagnosis**: **Calcium channel blocker (CCB) overdose**  **Key Supporting Evidence:**   1. **Persistent Bradycardia (HR 30) and Hypotension (76/54 mmHg)** despite fluids and naloxone:   CCBs (especially **non-dihydropyridines** like verapamil/diltiazem) suppress AV nodal conduction and myocardial contractility, leading to profound bradycardia, hypotension, and AV block.   1. **2nd-Degree AV Block (Type I) on ECG**:   Classic for **CCB toxicity**, which directly depresses the AV node. Beta-blockers can also cause AV block, but CCBs are more potent in this regard.   1. **Lack of Response to Naloxone**:   Opioid toxicity is partially reversed (respiratory rate may improve), but persistent hemodynamic instability suggests a **co-ingested cardiac toxin** (e.g., CCB).   1. **History of Hypertension on 3 Antihypertensives**:   CCBs are commonly prescribed for hypertension. Overdose of a non-dihydropyridine CCB aligns with this presentation.   1. **Excluded Alternatives**:   **Beta-blocker overdose**: Similar bradycardia/hypotension, but glucagon or calcium would show faster response.  **Digoxin overdose**: Level was 0, and ECG would show different findings (e.g., bidirectional VT, frequent ectopy).  **Hypoglycemia/Hypokalemia**: Already corrected (glucose normal, potassium 4.0).  **Hypothyroidism**: Normal TSH.  **Next Steps:**   1. **Empiric CCB Toxicity Treatment**:   **Calcium gluconate (1–3 g IV)** to counteract myocardial calcium channel blockade.  **Vasopressors** (e.g., norepinephrine) for refractory hypotension.  **High-dose insulin euglycemia therapy** (HIET) to improve myocardial glucose utilization.   1. **Confirm Exposure**:   Review the patient’s medication list for CCBs (e.g., verapamil, diltiazem).  **Why Not Beta-Blocker Overdose?** While beta-blockers can cause similar symptoms, the **AV block (type I)** and profound bradycardia are more characteristic of CCB toxicity. Additionally, beta-blocker overdose typically responds better to glucagon, which was not mentioned here.  **Conclusion**: The combination of **refractory bradycardia, AV block, and hypotension** in a hypertensive patient on multiple medications points overwhelmingly to **CCB overdose**. | **Most Likely Diagnosis:** **Calcium channel blocker (CCB) overdose**  **Key Supporting Evidence:**   1. **Persistent Bradycardia (HR 30) and Hypotension (76/54):**   Unresponsive to **naloxone** (opioid toxicity partially reversed) and **IV fluids**.  CCBs (e.g., verapamil, diltiazem) depress sinus node activity and AV conduction, leading to profound bradycardia and AV block.   1. **2nd-Degree AV Block (Type I):**   Classic in non-dihydropyridine CCB toxicity (e.g., verapamil/diltiazem), which directly impair AV node function. Beta-blockers can also cause AV block, but the ECG pattern here aligns more with CCBs.   1. **Lack of Response to Naloxone and Normal Opioid Levels Post-Treatment:**   Opioid overdose alone does not explain the worsening cardiac toxicity.   1. **Exclusion of Other Diagnoses:**   **Digoxin:** Level undetectable.  **Hypoglycemia:** Corrected (glucose 68 → normal post-dextrose).  **Hypocalcemia/Hypokalemia/Hypothyroidism:** Normal electrolytes and TSH.  **Marijuana:** No known association with life-threatening bradycardia or AV block.  **Why Not Beta-Blocker Overdose?** While beta-blockers can cause bradycardia/hypotension, **AV block is less common** unless combined with other agents. CCBs are more likely to directly cause **progressive AV conduction abnormalities** (e.g., type I AV block) and are less responsive to standard therapies (fluids, naloxone).  **Immediate Next Steps:**   - Administer **IV calcium gluconate** (10-20 mL of 10% solution) to improve cardiac conduction. - Start **high-dose insulin euglycemia therapy (HIET)** with insulin bolus (1 U/kg) and infusion (0.5-1 U/kg/hr) + dextrose to enhance myocardial glucose uptake. - Consider **vasopressors (e.g., norepinephrine)** for refractory hypotension. - Prepare for **lipid emulsion therapy** if CCB overdose is confirmed.   **Conclusion:** The patient’s **refractory bradycardia, AV block, and hypotension** despite naloxone/fluids strongly indicate **calcium channel blocker overdose** as the primary life-threatening toxicity. |
| mang | **Answer: Cardiac pacing, Atropine 0.5 to 1 mg IV q 3 to 5 min, Epinephrine 1 mcg/min IV, and Glucagon 5 mg over 1 min IV followed by 2 to 5 mg/h IV infusion.**  **Rationale:**   1. **Cardiac pacing:**   **Indicated for symptomatic bradycardia (HR 30) unresponsive to medications, especially with 2nd-degree AV block.**   1. **Atropine 0.5–1 mg IV:**   **First-line for bradycardia, though often ineffective in CCB overdose (AV node depression is calcium-dependent). Still recommended as initial therapy.**   1. **Epinephrine infusion:**   **Vasopressor of choice for hypotension refractory to fluids. Supports blood pressure and heart rate via β1 and α-adrenergic effects.**   1. **Glucagon:**   **Bypasses CCB-induced blockade by activating cardiac cAMP, improving inotropy and chronotropy. 5 mg bolus + infusion is standard for CCB overdose.**  **Why Not Other Options?:**   - **Admit to general medicine floor: Patient is hemodynamically unstable (HR 30, BP 76/54) and requires ICU-level care.** - **Outpatient psychiatry follow-up: Irrelevant in acute resuscitation. Focus on reversing toxicity first.**   **Next Steps:**   - **Start calcium gluconate (1–3 g IV) to counteract CCB effects.** - **Initiate high-dose insulin euglycemic therapy (HIET) if hypotension persists.** - **Prepare for transvenous pacing if transcutaneous pacing fails.** | **Most Appropriate Management Options**: ✅ **Cardiac pacing** ✅ **Atropine 0.5 to 1 mg IV q 3 to 5 min** ✅ **Epinephrine 1 mcg/min IV**  **Rationale:**   1. **Cardiac Pacing**:   The patient has **2nd-degree AV block (type I)** and profound bradycardia (HR 30) unresponsive to naloxone and fluids. While pacing may be less effective in CCB overdose due to myocardial depression, it is still indicated for **hemodynamically unstable AV block** as a temporizing measure.   1. **Atropine 0.5–1 mg IV**:   First-line for symptomatic bradycardia, even though CCB toxicity often limits its efficacy. A trial of atropine is still recommended before escalating to pacing or vasopressors.   1. **Epinephrine Infusion**:   **Refractory hypotension (BP 76/54 mmHg)** and bradycardia require vasopressors. Epinephrine provides both **beta-1 (chronotropic/inotropic) and alpha (vasoconstrictive) effects**, addressing both heart rate and blood pressure.  **Why Not Other Options?**   - **Glucagon**: Primarily used for **beta-blocker toxicity**, not CCB overdose. While it may have some theoretical benefit in CCB toxicity, calcium and high-dose insulin are preferred (not listed here). - **Admit to General Floor/Outpatient Psych Follow-Up**: The patient is critically ill and requires **ICU-level care** for continuous monitoring, vasopressors, and possible advanced interventions. - **Hypocalcemia/Hypokalemia/Hypoglycemia**: Already ruled out (normal electrolytes and corrected glucose).   **Critical Missing Therapy (Not Listed):**   - **Calcium Gluconate (1–3 g IV)**: First-line antidote for CCB overdose to counteract calcium channel blockade. - **High-Dose Insulin Euglycemia Therapy (HIET)**: Improves myocardial glucose utilization in CCB toxicity.   **Immediate Next Steps**:   1. Start **calcium gluconate** and **HIET** if not already initiated. 2. Transfer to the **ICU** for continuous hemodynamic monitoring and advanced support. | **Most Appropriate Management Options:**   1. **Cardiac Pacing**   Indicated for severe bradycardia (HR 30) with 2nd-degree AV block unresponsive to medications. While pacing may be less effective in CCB overdose due to myocardial depression, it should still be attempted in unstable patients.   1. **Epinephrine 1 mcg/min IV**   Provides both chronotropic (increases heart rate) and vasopressor support (improves hypotension). First-line for refractory hypotension/bradycardia in CCB overdose.   1. **Glucagon 5 mg over 1 min IV followed by 2–5 mg/h IV Infusion**   Although more effective in beta-blocker overdose, glucagon can partially counteract CCB-induced cardiotoxicity by activating cyclic AMP pathways independent of beta-receptors.   1. **Atropine 0.5–1 mg IV q 3–5 min**   A trial of atropine is reasonable despite limited efficacy in CCB overdose, as it may transiently improve AV nodal conduction in type I AV block.  **Excluded Options:**   - **Admit to general medicine floor**: Inappropriate due to hemodynamic instability; requires ICU-level care. - **Schedule outpatient psychiatry follow-up**: Critical acuity takes precedence; psychiatric evaluation should occur after stabilization.   **Rationale:** The patient’s **refractory bradycardia and hypotension** despite fluids/naloxone confirm CCB overdose as the primary driver. Interventions must address both cardiac conduction and vascular collapse. While calcium and high-dose insulin are cornerstone therapies for CCB toxicity, they are not listed options. From the provided choices, **pacing, epinephrine, glucagon, and atropine** align with ACLS/toxicity guidelines to stabilize the patient until definitive therapies (e.g., calcium, HIET) can be administered.  **Immediate Priority:** Initiate **epinephrine** and **glucagon** while preparing for **transcutaneous pacing**. Reassess responsiveness and escalate to advanced therapies (e.g., lipid emulsion, vasopressors) as needed. |
| Case 17 |  |  |  |
| Diff1 | **Answer: The following differential diagnoses cannot be excluded based on the history and physical examination:**   - **Alcohol intoxication** - **Cardiac arrhythmia** - **Hypoglycemia** - **Intracerebral hemorrhage** - **Opioid overdose** - **Pulmonary embolism (PE)** - **Seizure** - **Stroke affecting the posterior circulation** - **Subarachnoid hemorrhage** - **Vasovagal syncope**   **Rationale:**   1. **Alcohol Intoxication:**   **Altered mental status, lethargy, and respiratory depression (10/min) are consistent with severe intoxication. Though pupils are reactive, this does not fully exclude alcohol toxicity.**   2. **Cardiac Arrhythmia:**   **Profound bradycardia (HR 30) and hypotension suggest a primary arrhythmia (e.g., complete heart block, sick sinus syndrome), which could cause cerebral hypoperfusion and secondary seizure.**   3. **Hypoglycemia:**   **Blood glucose of 68 mg/dL is borderline low and may contribute to seizures or altered mental status, especially in susceptible individuals.**   4. **Intracerebral Hemorrhage:**   **Seizure, hypertension (initial BP 165/90), and altered consciousness could reflect a bleed. Imaging (e.g., CT head) is required to exclude.**   5. **Opioid Overdose:**   **Respiratory depression (10/min), bradycardia, and lethargy align with opioid toxicity. Pupils are typically pinpoint in overdose but may remain reactive in early stages or with mixed ingestions.**   6. **Pulmonary Embolism:**   **Hypoxia (SpO₂ 94%) and hypotension may occur in massive PE. Bradycardia is atypical but possible with vagal reflex activation or severe hemodynamic collapse.**   7. **Seizure:**   **Witnessed tonic-clonic activity (limb shaking, tongue laceration, urinary incontinence) strongly supports a seizure. Etiology (e.g., structural, metabolic) remains unclear without further workup.**   8. **Posterior Circulation Stroke:**   **Altered mental status and confusion may occur in brainstem or thalamic strokes. Focal deficits may be absent initially in posterior circulation ischemia.**   9. **Subarachnoid Hemorrhage (SAH):**   **Seizure and lethargy can occur in SAH. Classic meningismus or thunderclap headache may be absent early in the course.**   10. **Vasovagal Syncope:**   **Initial syncopal episode (sudden fall) could trigger a seizure due to cerebral hypoperfusion. Persistent bradycardia/hypotension, however, suggests a more severe underlying cause.**  **Excluded Diagnoses:**   - **Alcohol Withdrawal: Typically causes tachycardia, hypertension, and agitation, which are absent here.** - **Hyperventilation Syndrome: Respirations are slow (10/min), not rapid.** - **Meningitis: No fever, meningismus, or photophobia.** - **Orthostatic Hypotension: Irrelevant in the setting of persistent supine hypotension.** - **Psychiatric Disorder: Hemodynamic instability and objective neurologic signs (e.g., seizure) favor organic etiology.**   **Next Steps:**   - **Imaging: CT head to exclude hemorrhage, MRI for posterior circulation stroke.** - **Toxicology Screen: Assess for opioids, alcohol, or other toxins.** - **ECG: Evaluate for arrhythmia or ischemia.** - **Continuous Monitoring: For cardiac and respiratory support.** - **Glucose Administration: Repeat blood glucose and treat if hypoglycemic.** | **Differential Diagnoses That Cannot Be Excluded**: ✅ **Alcohol intoxication** ✅ **Cardiac arrhythmia** ✅ **Hypoglycemia** ✅ **Intracerebral hemorrhage** ✅ **Opioid overdose** ✅ **Pulmonary embolism (PE)** ✅ **Seizure** ✅ **Stroke affecting the posterior circulation** ✅ **Subarachnoid hemorrhage**  **Rationale for Inclusion:**   1. **Alcohol Intoxication**:   Severe intoxication can cause altered mental status, seizures, and respiratory depression. Though bradycardia is atypical, it cannot be fully excluded without confirmation of blood alcohol levels.   2. **Cardiac Arrhythmia**:   Profound bradycardia (HR 30) and hypotension suggest arrhythmia (e.g., complete heart block, sick sinus syndrome). ECG is required to rule out.   3. **Hypoglycemia**:   Seizures and lethargy can result from hypoglycemia. Despite normal glucose correction in prior cases, recurrent hypoglycemia or untreated episodes must be excluded with repeat testing.   4. **Intracerebral Hemorrhage**:   Seizures, altered mental status, and lethargy are consistent with a bleed. Lack of focal neurological deficits does not rule out small hemorrhages; imaging (CT/MRI) is needed.   5. **Opioid Overdose**:   Bradycardia, hypotension, and respiratory depression align with opioid toxicity. Reactive pupils do not exclude partial overdose or mixed drug ingestion.   6. **Pulmonary Embolism (PE)**:   While PE typically causes tachycardia, massive PE can lead to bradycardia due to hypoxia or vagal response. Hypoxia (SpO2 94%) and sudden collapse warrant consideration.   7. **Seizure**:   The witnessed seizure and postictal state (confusion, lethargy) are clear, but persistent hemodynamic instability suggests a secondary cause (e.g., toxin, metabolic derangement).   8. **Stroke (Posterior Circulation)**:   Brainstem strokes can cause bradycardia, hypotension, and altered consciousness without focal deficits. Imaging is required.   9. **Subarachnoid Hemorrhage (SAH)**:   SAH may present with seizures, altered mental status, and lethargy. Lack of meningismus or headache does not exclude it; non-contrast CT is necessary.  **Excluded Diagnoses:**   - **Alcohol Withdrawal**: Unlikely due to bradycardia/hypotension (withdrawal typically causes tachycardia/hypertension). - **Hyperventilation Syndrome**: Respiration rate is low (10/min), not elevated. - **Meningitis**: No fever, meningismus, or CSF findings. - **Orthostatic Hypotension**: Supine hypotension and severe bradycardia suggest a more systemic cause. - **Psychiatric Disorder**: Vital sign instability and objective neurological findings rule out functional causes. - **Vasovagal Syncope**: Symptoms are prolonged and severe, inconsistent with transient syncope.   **Key Next Steps:**   1. **Imaging**: Non-contrast CT head to rule out hemorrhage or stroke. 2. **ECG**: Evaluate for arrhythmias or ischemic changes. 3. **Glucose Check**: Confirm normal levels. 4. **Toxicology Screen**: Assess for opioids, alcohol, or other toxins. 5. **ABG/D-Dimer**: If PE is suspected.   **Conclusion**: The patient’s presentation (seizure followed by hemodynamic collapse) requires broad consideration of neurologic, toxicologic, and cardiovascular etiologies. Immediate imaging and labs are critical to narrow the differential. | **Differential Diagnoses That Cannot Be Excluded:**   1. **Alcohol Intoxication**   Despite atypical bradycardia/hypotension, alcohol intoxication cannot be ruled out without a blood alcohol level. Altered mental status and possible aspiration (saliva/blood at mouth) are consistent with severe intoxication.   2. **Cardiac Arrhythmia**   The profound bradycardia (HR 30) suggests a primary cardiac etiology (e.g., complete heart block, sick sinus syndrome) or toxin-induced arrhythmia (e.g., beta-blocker/calcium channel blocker overdose). ECG and cardiac monitoring are required.   3. **Hypoglycemia**   Although initial glucose was corrected (68 → normal post-dextrose), recurrent hypoglycemia (e.g., sulfonylurea overdose, insulinoma) could explain persistent lethargy. Repeat glucose testing is needed.   4. **Intracerebral Hemorrhage (ICH)**   The initial seizure and altered mental status could reflect a hemorrhagic stroke. Lack of focal neurologic deficits does not exclude a non-eloquent area bleed (e.g., thalamus, cerebellum). CT head is required.   5. **Opioid Overdose**   Respiratory depression (RR 10) and lethargy align with opioid toxicity. Pupils are reactive (atypical for pure opioid overdose), but mixed overdoses (e.g., opioids + other sedatives) or partial response to naloxone may explain findings.   6. **Pulmonary Embolism (PE)**   While PE classically causes tachycardia, massive PE can lead to bradycardia and hypotension due to hemodynamic collapse. Hypoxia (pulse oximetry 94%) and sudden collapse are suggestive. CT angiography is needed.   7. **Seizure**   The witnessed seizure-like activity (tonic-clonic movements, tongue laceration, urinary incontinence) supports a seizure. Prolonged postictal state or non-convulsive status epilepticus could explain persistent lethargy. EEG is required.   8. **Stroke Affecting the Posterior Circulation**   Brainstem strokes (e.g., basilar artery occlusion) can cause altered mental status, bradycardia, and hypotension without focal deficits. MRI/MRA is necessary for exclusion.   9. **Subarachnoid Hemorrhage (SAH)**   Sudden collapse, seizure, and lethargy are consistent with SAH. Absence of meningismus or headache does not exclude it. Non-contrast CT head or LP is required.  **Differential Diagnoses That Can Be Excluded:**   - **Alcohol Withdrawal**: Inconsistent with bradycardia/hypotension; withdrawal typically causes tachycardia, hypertension, and agitation. - **Hyperventilation Syndrome**: Patient has **hypoventilation** (RR 10), not hyperventilation. - **Meningitis**: No fever, meningismus, or leukocytosis. - **Orthostatic Hypotension**: Supine hypotension (74/52 mmHg) rules this out. - **Psychiatric Disorder**: Objective signs (bradycardia, hypotension, respiratory depression) indicate organic pathology. - **Vasovagal Syncope**: Symptoms are persistent, not transient.   **Key Next Steps:**   - **Imaging**: CT head (rule out ICH, SAH, stroke), CTA chest (rule out PE). - **Labs**: Repeat glucose, toxicology screen, lactate, ABG. - **ECG**: Evaluate for heart block/ischemia. - **Continuous Monitoring**: Cardiac telemetry, neurologic checks.   **Conclusion:** The patient’s presentation is most consistent with **toxin/drug overdose** (e.g., opioids, CCBs) or **neurologic catastrophe** (e.g., stroke, SAH). However, multiple life-threatening etiologies remain plausible until imaging and labs confirm the diagnosis. |
| Mang1 | **Answer: The following steps are essential to perform immediately:**   - **Bedside glucose measurement** - **BP measurement every 5 min until stable** - **Cardiac monitoring** - **Dextrose 50 g IV (if hypoglycemia confirmed)** - **Naloxone 0.4 mg IV** - **Pulse oximetry**   **Rationale:**   1. **Bedside Glucose Measurement:**   **Critical to confirm hypoglycemia (previous glucose was 68 mg/dL, borderline low). Hypoglycemia can mimic seizures, lethargy, and hemodynamic instability.**   2. **BP Measurement Every 5 Minutes:**   **Severe hypotension (74/52 mmHg) requires aggressive monitoring to guide resuscitation (e.g., fluids, vasopressors).**   3. **Cardiac Monitoring:**   **Profound bradycardia (HR 30) may indicate arrhythmia (e.g., complete heart block) or toxicity (e.g., calcium channel blocker overdose). Continuous monitoring is necessary to detect deterioration or response to interventions.**   4. **Dextrose 50 g IV:**   **Administer only after confirming hypoglycemia. Rapid correction of hypoglycemia can reverse altered mental status and seizures.**   5. **Naloxone 0.4 mg IV:**   **Respiratory depression (10/min), bradycardia, and lethargy are consistent with opioid overdose. Naloxone can reverse opioid effects even if pupils are reactive (e.g., mixed overdose or incomplete blockade).**   6. **Pulse Oximetry:**   **Hypoxia (SpO₂ 94% on room air) may worsen with respiratory depression. Continuous monitoring ensures timely intervention (e.g., supplemental oxygen, intubation).**  **Steps Not Immediately Essential:**   - **Endotracheal Intubation:**   **While respiratory rate is low (10/min), the patient currently protects his airway. Intubation is urgent but not immediately required unless oxygenation/ventilation deteriorates.**   - **Cold Caloric Testing:**   **Assesses brainstem function but is not a priority during hemodynamic instability.**   - **Lumbar Puncture:**   **Indicated for suspected meningitis/SAH but requires stabilization first.**   - **Toxicology Screening:**   **Important for diagnosis but does not replace immediate interventions (e.g., naloxone, glucose).**  **Immediate Priorities:**   1. **Stabilize hemodynamics: Administer fluids, vasopressors (e.g., epinephrine), and pacing if bradycardia persists.** 2. **Reverse reversible causes: Naloxone for opioids, dextrose for hypoglycemia.** 3. **Prepare for advanced interventions: Intubation if respiratory failure occurs, transcutaneous pacing for refractory bradycardia.** | **Essential Immediate Steps**: ✅ **Bedside glucose measurement** ✅ **BP measurement every 5 min until stable** ✅ **Cardiac monitoring** ✅ **Naloxone 0.4 mg IV** ✅ **Pulse oximetry** ✅ **Toxicology screening**  **Rationale:**   1. **Bedside Glucose Measurement**:   Critical to rule out hypoglycemia as a cause of seizures, altered mental status, and hemodynamic instability. Immediate testing is required before administering dextrose.   2. **BP Measurement Every 5 Minutes**:   The patient is hypotensive (74/52 mmHg) and requires close hemodynamic monitoring to guide resuscitation efforts (e.g., fluid/vasopressor response).   3. **Cardiac Monitoring**:   Profound bradycardia (HR 30) necessitates continuous ECG monitoring to detect arrhythmias (e.g., complete heart block) or ischemia.   4. **Naloxone 0.4 mg IV**:   Opioid overdose can cause respiratory depression, bradycardia, and altered mental status. Reactive pupils do not exclude partial overdose or mixed ingestions. Naloxone is a safe, rapid intervention.   5. **Pulse Oximetry**:   Continuous monitoring is essential given the patient’s respiratory depression (10/min) and borderline SpO2 (94%). Ensures early detection of hypoxia.   6. **Toxicology Screening**:   Urgent blood/urine toxicology tests are needed to identify potential overdoses (e.g., calcium channel blockers, opioids) or toxins causing seizures and cardiovascular collapse.  **Excluded Options:**   - **Dextrose 50 g IV**: Should only be administered **after** confirming hypoglycemia via bedside glucose testing to avoid harm. - **Endotracheal Intubation**: While respiratory rate is low (10/min), the patient currently protects his airway and maintains SpO2 ≥94%. Close monitoring is required, but immediate intubation is not yet indicated. - **Cold Caloric Testing**: Not urgent in this context; prioritizes stabilizing vital signs and identifying reversible causes. - **Lumbar Puncture**: Requires stabilization and imaging (e.g., CT head) first. Not an immediate step in hemodynamically unstable patients.   **Key Considerations:**   - **Hypoglycemia** must be ruled out before administering dextrose. - **Naloxone** may reverse opioid effects even if pupils are reactive (e.g., partial overdose or co-ingestions). - **Toxicology screening** guides antidote therapy (e.g., calcium for CCB overdose, high-dose insulin).   **Conclusion**: Focus on rapid assessment of reversible causes (hypoglycemia, opioids) and continuous monitoring of vital signs while preparing for advanced interventions (e.g., vasopressors, pacing). | **Essential Immediate Steps:**   1. **Bedside Glucose Measurement**   Critical to rule out hypoglycemia as a cause of altered mental status, especially given the patient’s prior hypoglycemia (68 mg/dL).   2. **BP Measurement Every 5 Minutes Until Stable**   Required for monitoring profound hypotension (74/52 mmHg) and guiding resuscitation efforts.   3. **Cardiac Monitoring**   Necessary to assess for arrhythmias (e.g., heart block) or ischemia, given the extreme bradycardia (HR 30).   4. **Naloxone 0.4 mg IV**   Empiric treatment for potential opioid overdose, which can cause respiratory depression (RR 10) and altered mental status.   5. **Pulse Oximetry**   Continuous monitoring is essential to detect worsening hypoxia (current SpO₂ 94% on room air) and guide oxygen therapy or intubation.   6. **Dextrose 50 g IV**   Administer **if hypoglycemia is confirmed** on bedside testing. Though the patient’s glucose was previously corrected, recurrent hypoglycemia must be excluded.   7. **Toxicology Screening**   Send blood/urine for toxicology to identify potential overdoses (e.g., calcium channel blockers, opioids) or toxins contributing to the presentation.  **Excluded Immediate Steps:**   - **Endotracheal Intubation**: While respiratory rate is low (10/min), the patient currently protects his airway. Intubation may become necessary if respiratory status deteriorates. - **Lumbar Puncture**: Not urgent without signs of meningitis (no fever, meningismus) or if CT head excludes hemorrhage first. - **Cold Caloric Testing**: A neuro exam tool for brainstem function but not a priority in acute hemodynamic instability.   **Key Rationale:** The patient’s **bradycardia, hypotension, and respiratory depression** demand immediate stabilization with cardiac monitoring, BP checks, and interventions for reversible causes (e.g., hypoglycemia, opioid overdose). Toxicology screening and glucose testing are critical to identify underlying etiologies.  **Conclusion:** Prioritize **ABC stabilization**, **empiric naloxone**, **glucose assessment**, and **continuous hemodynamic monitoring** while pursuing toxicology studies to guide definitive management. |
| Diag1 | **Answer: The most appropriate steps to guide diagnosis and treatment are:**   - **Head CT** - **Toxicology screen** - **Seek additional history from patient's family and records** - **Reexamination of the patient** - **CBC** - **Serum chemistry profile**   **Rationale:**   1. **Head CT:**   **Critical to exclude intracranial hemorrhage, stroke (e.g., posterior circulation), or structural lesions given the patient’s initial severe bradycardia, hypotension, and altered mental status.**   2. **Toxicology Screen:**   **Priority to identify opioid overdose, alcohol, or other toxins (e.g., sedatives, beta-blockers) that could explain respiratory depression, bradycardia, and lethargy. Confirms or excludes substance-related causes.**   3. **Seek Additional History:**   **Essential to uncover medication use, substance abuse, trauma, or prior medical conditions (e.g., seizures, cardiac issues). Family or records may reveal opioid prescriptions, alcohol use, or recent illnesses.**   4. **Reexamination of the Patient:**   **Key to reassess mental status, vital signs, and neurological deficits after initial stabilization. Detects evolving conditions (e.g., worsening hypoxia, focal neurologic signs).**   5. **CBC:**   **Evaluates for infection (e.g., sepsis, meningitis), anemia, or leukocytosis. Supports diagnosis of occult bleeding or systemic illness.**   6. **Serum Chemistry Profile:**   **Assesses electrolytes (e.g., sodium, calcium), renal/hepatic function, and acid-base status. Rules out metabolic derangements (e.g., hyponatremia, uremia) contributing to altered mental status.**  **Steps Not Immediately Necessary:**   - **Carotid Doppler Testing:**   **Indicated for suspected carotid stenosis or stroke but requires neuroimaging (Head CT) first. Not urgent unless focal neurologic deficits persist.**   - **Lumbar Puncture:**   **Defer until after Head CT. If imaging is negative and suspicion remains for meningitis/SAH, LP becomes necessary.**   - **Tilt Table Testing:**   **Evaluates vasovagal syncope but inappropriate in acute instability. The patient’s profound hypotension/bradycardia is unlikely vasovagal given severity.**   - **ECG:**   **Already performed and normal. Repeat only if new symptoms (e.g., chest pain, arrhythmia) arise.**  **Next Steps After These Tests:**   1. **Treat Identified Causes:**   **Administer naloxone if opioids are confirmed.**  **Start antibiotics if infection is suspected.**   2. **Proceed to LP if Head CT is negative and CNS infection/SAH is still possible.** 3. **Consider MRI Brain if posterior circulation stroke is suspected despite normal CT.** 4. **Monitor Closely for recurrence of instability or mental status changes.** | **Most Appropriate Next Steps**: ✅ **Head CT** ✅ **Lumbar puncture for CSF examination** ✅ **Toxicology screen** ✅ **Reexamination of the patient** ✅ **Seek additional history from family/records** ✅ **CBC** ✅ **Serum chemistry profile**  **Rationale:**   1. **Head CT**:   **Critical** to rule out intracranial hemorrhage, stroke, or structural lesions (e.g., tumor, abscess) as causes of seizures and altered mental status. Must be performed before lumbar puncture.   2. **Lumbar Puncture (after Head CT)**:   Required to evaluate for **meningitis/encephalitis** or **subarachnoid hemorrhage** (if CT is negative). CSF analysis is essential given the unresolved differential diagnosis.   3. **Toxicology Screen**:   Confirm or exclude opioid overdose, alcohol intoxication, or other toxins (e.g., cocaine, sympathomimetics) that could explain seizures and hemodynamic instability.   4. **Reexamination of the Patient**:   Assess for evolving neurological deficits, vital sign trends, or new symptoms (e.g., fever, focal signs) to guide urgency of interventions.   5. **Seek Additional History**:   Obtain details about recent substance use, trauma, infections, or prior seizures from family/records. Critical for identifying triggers (e.g., opioid use, head injury).   6. **CBC**:   Evaluate for infection (e.g., leukocytosis in meningitis) or anemia contributing to hypoxia/seizures.   7. **Serum Chemistry Profile**:   Check electrolytes (e.g., sodium, calcium), renal/hepatic function, and glucose to identify metabolic causes (e.g., hyponatremia, uremia) of seizures or altered mental status.  **Excluded Options:**   - **Carotid Doppler Testing**: Not urgent unless focal neurological deficits suggest carotid stenosis (e.g., stroke). Prioritize Head CT first. - **ECG**: Already performed and normal. Repeat only if new cardiac symptoms arise. - **Tilt Table Testing**: Irrelevant in this context (used for syncope, not seizures/instability).   **Key Considerations:**   - **Head CT must precede lumbar puncture** to avoid herniation risk if a mass lesion is present. - **Toxicology results** may guide antidotes (e.g., naloxone escalation) or confirm overdose. - **CSF analysis** is critical if infection or subarachnoid hemorrhage is suspected despite normal imaging.   **Conclusion**: Focus on identifying structural brain pathology, infectious/inflammatory causes, and toxicologic etiologies while stabilizing the patient and gathering collateral history. | **Most Appropriate Steps to Guide Diagnosis and Treatment:**   1. **Head CT**   **Priority** to rule out intracranial hemorrhage, stroke, or structural lesions causing altered mental status.   2. **Lumbar Puncture for CSF Examination**   **After CT Head is negative**, to evaluate for meningitis/encephalitis or subarachnoid hemorrhage.   3. **Toxicology Screen**   Essential to identify drug overdoses (e.g., beta-blockers, calcium channel blockers, opioids) given bradycardia, hypotension, and altered mental status.   4. **CBC**   Assess for infection (e.g., sepsis) or anemia contributing to hypotension/lethargy.   5. **Serum Chemistry Profile**   Check electrolytes (Na⁺, Ca²⁺), renal/liver function, and lactate to exclude metabolic derangements or organ dysfunction.   6. **Reexamination of the Patient**   Monitor for changes in neurologic status, vital signs, or new findings (e.g., focal deficits, rash).   7. **Seek Additional History from Family/Records**   Critical to uncover medication use, toxin exposure, prior seizures, or infections (e.g., recent fever, travel).  **Excluded Options:**   - **Carotid Doppler Testing**: Not urgent unless focal neurologic deficits suggest carotid disease (no evidence here). - **ECG**: Already performed and showed sinus rhythm; repeat only if new cardiac symptoms arise. - **Tilt Table Testing**: Irrelevant in this acute, hypotensive, critically ill patient.   **Rationale**: The patient’s **persistent hypotension, bradycardia, and lethargy** require urgent evaluation for intracranial pathology (CT), CNS infection (LP), and toxins (toxicology). Basic labs (CBC, chemistry) and thorough history-taking are foundational. Reassessment ensures timely intervention if clinical status deteriorates.  **Key Sequence**:   1. **CT Head** → Immediate. 2. **Toxicology Screen + CBC/Chemistry** → Send stat. 3. **LP if CT negative** → Rule out infection/SAH. 4. **History + Reexam** → Ongoing to refine differentials. |
| Diff2 | **Answer: The likely differential diagnoses at this time include:**   - **Intracerebral hemorrhage** - **Seizure**   **Rationale:**   1. **Intracerebral Hemorrhage (ICH):**   **Confirmed by the head CT showing intraparenchymal hemorrhage in the right parietal area. This is the definitive diagnosis and explains the patient’s focal neurologic deficit (left-sided dysdiadochokinesia) and initial altered mental status.**   2. **Seizure:**   **Considered as a potential complication of the ICH or a preceding event. While there is no explicit history of witnessed seizure activity, seizures can occur secondary to intracranial hemorrhage. Subtle dysdiadochokinesia could also reflect postictal Todd’s paralysis, though it is more likely directly related to the hemorrhage itself.**  **Excluded Options:**   - **Subarachnoid Hemorrhage (SAH):**   **The head CT identified intraparenchymal hemorrhage, not subarachnoid blood. SAH is unlikely unless there is concurrent pathology (e.g., aneurysm rupture with both ICH and SAH), but imaging does not support this.**   - **Cardiac Arrhythmia:**   **ECG and continuous monitoring showed normal sinus rhythm. Initial bradycardia/hypotension was likely secondary to the ICH (e.g., Cushing’s reflex) rather than a primary cardiac cause.**   - **Vasovagal Syncope:**   **Inconsistent with the focal neurologic deficit and imaging findings. Syncope would not explain the hemorrhage unless it caused a fall, but no trauma was reported.**   - **Alcohol Withdrawal:**   **The patient is a social drinker with no clinical or lab findings (e.g., tremor, agitation, electrolyte abnormalities) suggestive of withdrawal.**   - **Thrombocytopenia:**   **CBC results were normal, and there is no evidence of low platelet count. Aspirin use affects platelet function, not count.**  **Key Considerations:**   - **The intracerebral hemorrhage is the primary diagnosis, requiring urgent neurosurgical evaluation and blood pressure management.** - **Seizure remains a potential differential due to the risk of post-ICH seizure activity, though it is less likely than the hemorrhage itself as the root cause. Further evaluation (e.g., EEG) may be warranted if clinical suspicion persists.** - **Other diagnoses (e.g., SAH, arrhythmia) are effectively ruled out by imaging and monitoring.** | **Likely Differential Diagnoses:** ✅ **Intracerebral hemorrhage** ✅ **Seizure**  **Rationale:**   1. **Intracerebral Hemorrhage (ICH)**:   **Confirmed by Head CT**: The imaging shows a right parietal intraparenchymal hemorrhage, which directly explains the patient’s seizure, subtle left-sided dysdiadochokinesia, and headache. This is now the definitive diagnosis.   2. **Seizure**:   **Secondary to ICH**: The patient’s initial seizure was likely provoked by the hemorrhage (a common complication of ICH due to cortical irritation). While the hemorrhage is the root cause, "seizure" remains relevant as a clinical manifestation requiring monitoring and potential treatment (e.g., antiepileptics if recurrent).  **Excluded Options:**   - **Subarachnoid Hemorrhage (SAH)**: Ruled out by CT showing **intraparenchymal** (not subarachnoid) bleeding. - **Alcohol Withdrawal**: No history of heavy alcohol use or withdrawal symptoms (e.g., tremor, tachycardia). - **Cardiac Arrhythmia**: ECG consistently shows normal sinus rhythm. - **Thrombocytopenia**: CBC is normal, with no platelet deficiency. - **Vasovagal Syncope**: Inconsistent with the presentation (seizure activity and focal neurologic deficits).   **Key Considerations:**   - The **seizure** is not a standalone diagnosis but a symptom of the underlying ICH. However, it remains clinically significant for management (e.g., monitoring for recurrence). - **ICH** is the primary diagnosis, with next steps focusing on hemorrhage stabilization, coagulopathy reversal (given aspirin/fish oil use), and identifying underlying causes (e.g., hypertension, vascular malformation).   **Conclusion**: The confirmed **intracerebral hemorrhage** and its associated **seizure** activity are the central diagnoses guiding immediate care. Other options are inconsistent with imaging, labs, or history. | **Likely Differential Diagnoses:**   1. **Intracerebral Hemorrhage**   **Confirmed by CT**: The head CT identified a right parietal intraparenchymal hemorrhage, which directly explains the patient’s neurologic deficits (left-sided dysdiadochokinesia), seizure-like activity, and altered mental status.   2. **Seizure**   **Secondary to Hemorrhage**: The patient’s tonic-clonic activity at presentation likely resulted from irritation of cortical tissue by the hemorrhage. Seizures are a common complication of intraparenchymal bleeding.  **Excluded Diagnoses:**   - **Alcohol Withdrawal**: No history of heavy alcohol use, and symptoms (hypotension, bradycardia) contradict typical withdrawal features (tachycardia, hypertension, agitation). - **Cardiac Arrhythmia**: Normal sinus rhythm on ECG and continuous monitoring rules this out. - **Subarachnoid Hemorrhage (SAH)**: CT confirmed intraparenchymal (not subarachnoid) hemorrhage. SAH typically presents with thunderclap headache and meningismus, absent here. - **Thrombocytopenia**: Normal CBC (no reported platelet deficiency). Hemorrhage is attributed to aspirin’s antiplatelet effect, not low platelets. - **Vasovagal Syncope**: Persistent focal neurologic deficits (dysdiadochokinesia) and structural hemorrhage findings exclude transient syncope.   **Rationale**: The **intracerebral hemorrhage** is the definitive diagnosis, with **seizure** as a secondary complication. Other options are inconsistent with imaging, labs, or clinical history. |
| Diag2 | **Answer: The most appropriate steps for guiding diagnosis and treatment include:**   - **Liver function tests** - **Plasma fibrinogen and ᴅ-dimer levels** - **Peripheral blood smear** - **PT/PTT** - **Repeat physical examination of patient for signs of bleeding**   **Rationale:**   1. **PT/PTT:**   **Critical to evaluate for coagulopathy (e.g., due to liver dysfunction, anticoagulant use, or inherited clotting disorders). The patient’s aspirin use increases bleeding risk, and PT/INR may reveal underlying issues.**   2. **Liver Function Tests (LFTs):**   **Liver dysfunction can impair clotting factor synthesis (e.g., vitamin K-dependent factors). The patient’s social drinking history and aspirin use make this relevant.**   3. **Plasma Fibrinogen and ᴅ-Dimer:**   **Assess for hyperfibrinolysis or disseminated intravascular coagulation (DIC). Elevated ᴅ-dimer may suggest active thrombosis or clot breakdown, though less likely in this localized hemorrhage.**   4. **Peripheral Blood Smear:**   **Evaluate for microangiopathic changes (e.g., schistocytes in thrombotic thrombocytopenic purpura) or platelet abnormalities (e.g., giant platelets in MYH9-related disorders), though CBC was normal.**   5. **Repeat Physical Exam for Bleeding Signs:**   **Look for petechiae, ecchymoses, or mucosal bleeding to assess for systemic coagulopathy or platelet dysfunction.**  **Excluded Options:**   - **Bleeding Time Test:**   **Outdated and unreliable; replaced by platelet function assays (not listed here).**   - **Bone Marrow Biopsy:**   **Unnecessary with normal CBC and no evidence of hematologic malignancy.**   - **Coombs Test:**   **Irrelevant without hemolytic anemia (normal hemoglobin and no jaundice).**   - **Hepatitis C/HIV Testing:**   **Low yield unless risk factors (e.g., IV drug use, high-risk sexual behavior) are present.**   - **No Additional Testing:**   **Inappropriate; the cause of spontaneous intracerebral hemorrhage (e.g., coagulopathy, vascular malformation) must be investigated.**  **Key Considerations:**   - **The hemorrhage etiology may include hypertension (common in lobar hemorrhages), cerebral amyloid angiopathy, or coagulopathy (e.g., from aspirin or liver disease).** - **PT/PTT and fibrinogen are essential to guide reversal of coagulopathy (e.g., with vitamin K, fresh frozen plasma).** - **Vascular imaging (CTA/MRA) and MRI brain (previously recommended) remain critical to exclude structural causes (e.g., AVM, aneurysm).**   **Immediate priorities: Stabilize the hemorrhage, reverse coagulopathy, and identify underlying causes.** | **Most Appropriate Steps for Diagnosis and Treatment:** ✅ **PT/PTT** ✅ **Liver function tests** ✅ **Plasma fibrinogen and ᴅ-dimer levels** ✅ **Peripheral blood smear** ✅ **Repeat physical examination of patient for signs of bleeding**  **Rationale:**   1. **PT/PTT**:   Essential to evaluate coagulation status, especially given the patient’s aspirin and fish oil use (both increase bleeding risk). While aspirin does not prolong PT/PTT, these tests rule out other coagulopathies (e.g., liver dysfunction, occult anticoagulant use).   2. **Liver Function Tests**:   The liver synthesizes clotting factors. Abnormal LFTs could indicate hepatic dysfunction contributing to coagulopathy (e.g., impaired vitamin K-dependent factor synthesis).   3. **Plasma Fibrinogen and ᴅ-Dimer**:   Assess for disseminated intravascular coagulation (DIC) or hyperfibrinolysis, which can complicate intracerebral hemorrhage (ICH). Elevated ᴅ-dimer may also suggest thrombotic risk.   4. **Peripheral Blood Smear**:   Evaluates platelet morphology and count (thrombocytopenia or platelet dysfunction) and screens for microangiopathic changes (e.g., schistocytes in TTP).   5. **Repeat Physical Examination**:   Identify signs of systemic bleeding (e.g., ecchymoses, petechiae) or neurologic deterioration (e.g., papilledema, focal deficits).  **Excluded Options:**   - **No Additional Testing**: Inappropriate; ICH requires workup for underlying causes (e.g., coagulopathy, vascular malformations). - **Bleeding Time Test**: Outdated and rarely used in clinical practice; replaced by platelet function assays (not listed). - **Bone Marrow Biopsy**: Unnecessary with normal CBC and no evidence of hematologic disorder. - **Coombs Test**: Irrelevant without signs of hemolysis (normal hemoglobin/hematocrit). - **Hepatitis C/HIV Testing**: Lower priority unless risk factors are present (none documented here), though may be considered later in young patients with unexplained ICH.   **Key Considerations:**   - The focus is on identifying reversible causes of hemorrhage (e.g., coagulopathy from aspirin/fish oil, liver dysfunction) and monitoring for complications (e.g., DIC, edema). - **Next Steps**: Neurosurgery consultation, blood pressure control, and advanced imaging (CTA/MRA) remain critical (as previously outlined).   **Conclusion**: These steps address immediate coagulopathy evaluation and systemic bleeding risk, aligning with the patient’s medication history and confirmed ICH. | **Most Appropriate Steps for Diagnosis/Treatment:**   1. **PT/PTT**   **Why**: Aspirin (antiplatelet) use increases bleeding risk. PT/PTT evaluates for coagulopathy (e.g., warfarin use, liver dysfunction) that could exacerbate hemorrhage.   2. **Peripheral Blood Smear**   **Why**: Assess platelet morphology and count (though initial CBC was normal) to rule out microangiopathic processes (e.g., TTP) or platelet dysfunction.   3. **Plasma Fibrinogen and D-dimer Levels**   **Why**: Elevated D-dimer could suggest thrombotic or consumptive coagulopathy (e.g., DIC), while low fibrinogen may indicate bleeding risk.   4. **Liver Function Tests**   **Why**: Liver dysfunction impairs clotting factor synthesis (e.g., vitamin K-dependent factors), worsening hemorrhage risk.   5. **Repeat Physical Examination for Signs of Bleeding**   **Why**: Monitor for expanding hemorrhage (e.g., worsening neurologic deficits, pupillary changes, hypotension).  **Excluded Options**:   - **No Additional Testing**: Inappropriate—bleeding etiology (e.g., vascular malformation, coagulopathy) must be identified. - **Bleeding Time Test**: Obsolete; replaced by platelet function assays (not urgent here). - **Bone Marrow Biopsy**: Unnecessary without cytopenias or abnormal blood counts. - **Coombs Test**: For hemolytic anemia; irrelevant here. - **Hepatitis C/HIV Testing**: Low pretest probability given social history (no risk factors).   **Critical Priorities**:   - **Rule out coagulopathy** (PT/PTT, fibrinogen). - **Identify platelet dysfunction** (smear). - **Assess liver contribution to clotting** (LFTs). - **Monitor for clinical deterioration** (repeat exam).   **Next Step**: Proceed with **CT angiography (CTA)** to evaluate for vascular malformation (e.g., AVM, aneurysm), the most likely underlying cause in a normotensive patient with no trauma. |
| Dx | **Answer: The most likely diagnosis is Immune thrombocytopenia (ITP).**  **Rationale:**   1. **Immune Thrombocytopenia (ITP):**   **Isolated thrombocytopenia with normal CBC otherwise (no anemia/leukopenia).**  **Petechiae/ecchymosis on exam, consistent with platelet-related bleeding.**  **Normal PT/PTT, fibrinogen, and liver function rule out coagulopathy, liver disease, or DIC.**  **Absence of splenomegaly and negative infectious workup (HIV/hepatitis) support ITP as a diagnosis of exclusion.**   2. **Why Not Other Options:**   **Bone Marrow Failure:**  **Typically causes pancytopenia (not isolated thrombocytopenia). Bone marrow biopsy may still be needed, but clinical picture favors ITP.**  **Drug Effects:**  **Aspirin impairs platelet function but does not reduce platelet count. No other drugs implicated (e.g., heparin, quinine).**  **Sepsis:**  **No fever, leukocytosis, or hemodynamic instability. Normal fibrinogen/ᴅ-dimer argue against DIC.**  **Von Willebrand Disease (VWD):**  **Causes mucocutaneous bleeding but does not reduce platelet count. PT/PTT are typically normal in most VWD types.**  **Supporting Evidence:**   - **ITP is an autoimmune disorder targeting platelets.** - **The patient’s prior aspirin use may have exacerbated bleeding risk but is not the cause of thrombocytopenia.** - **First-line treatment (steroids/IVIG) would address immune-mediated destruction.**   **Next Step: Confirm with bone marrow biopsy (to rule out marrow pathology) and platelet antibody testing.** | **Most Likely Diagnosis:** ✅ **Immune Thrombocytopenia (ITP)**  **Key Supporting Evidence:**   1. **Isolated Thrombocytopenia**:   Peripheral blood smear confirms low platelets without other cytopenias (normal CBC otherwise), ruling out bone marrow failure (e.g., aplastic anemia) or sepsis (typically associated with leukocytosis/leukopenia).   2. **Absence of Systemic Illness**:   Normal liver function, coagulation studies (PT/PTT), and infectious workup (hepatitis/HIV) exclude liver disease, DIC, or viral causes.  No fever or signs of infection (e.g., normal WBC), making sepsis unlikely.   3. **Bleeding Signs Without Splenomegaly**:   Petechiae and ecchymosis are classic for thrombocytopenia. Splenomegaly is absent, which is common in ITP (vs. hypersplenism or lymphoma).   4. **Exclusion of Other Causes**:   **Drug-induced thrombocytopenia**: Unlikely unless the patient recently started a new medication (none documented; aspirin/fish oil do not lower platelet counts).  **Von Willebrand Disease (VWD)**: Irrelevant here—VWD causes platelet dysfunction or factor deficiency, not thrombocytopenia. PT/PTT are normal.  **Why Not Other Options?**   - **Bone Marrow Failure**: Requires pancytopenia (not isolated thrombocytopenia). Bone marrow biopsy may still be pursued if ITP treatment fails, but it is not the first-line diagnosis. - **Sepsis**: Requires clinical evidence of infection (e.g., fever, hypotension, organ dysfunction), which is absent. - **Drug Effects**: No temporal association with thrombocytopenia-inducing drugs (e.g., heparin, sulfonamides). - **VWD**: Platelet count is normal in VWD; bleeding in VWD is due to qualitative defects, not low platelets.   **Next Steps:**   1. **Confirm ITP**:   Exclude pseudothrombocytopenia (repeat smear with EDTA-anticoagulated and citrate tubes).  Consider antiplatelet antibody testing (though not always necessary for diagnosis).   2. **Immediate Management**:   **IVIG or corticosteroids** to rapidly increase platelet counts (critical given intracerebral hemorrhage).  **Platelet transfusion** for active bleeding or prophylaxis in ICH.   3. **Monitor for Complications**:   Serial neurologic exams and imaging to assess ICH stability.  **Conclusion**: ITP is the most likely diagnosis in this context of isolated thrombocytopenia with bleeding and exclusion of other causes. Urgent treatment is required to prevent life-threatening hemorrhage. | **Most Likely Diagnosis**: **Immune Thrombocytopenia (ITP)**  **Rationale**:   1. **Key Findings Supporting ITP**:   **Acute thrombocytopenia** (isolated low platelets on peripheral smear).  **Bleeding manifestations** (petechiae, ecchymosis, and prior intracranial hemorrhage).  **Normal PT/PTT, liver function, and coagulation factors** (rules out coagulopathy, liver disease, or von Willebrand disease).  **No splenomegaly or systemic symptoms** (e.g., fever, organ dysfunction) to suggest sepsis or bone marrow failure.   2. **Exclusion of Alternatives**:   **Drug Effects**: Aspirin does not cause thrombocytopenia (only platelet dysfunction). No other drugs (e.g., heparin, antibiotics) implicated.  **Bone Marrow Failure**: Normal initial CBC and lack of pancytopenia make this unlikely.  **Sepsis**: No fever, leukocytosis, or signs of infection.  **Von Willebrand Disease (VWD)**: Normal PT/PTT and acute presentation inconsistent with VWD.   3. **ITP Pathophysiology**:   Immune-mediated platelet destruction (autoantibodies targeting platelets) leading to isolated thrombocytopenia.  Aspirin likely worsened bleeding risk but did not directly cause the thrombocytopenia.  **Next Steps**:   - Confirm with **platelet antibody testing** (anti-glycoprotein GPIIb/IIIa or GPIb/IX). - Initiate **steroids (e.g., prednisone) + IVIG** for rapid platelet count recovery. - Monitor for recurrent bleeding or platelet count trends.   **Answer**: **Immune thrombocytopenia (ITP)** |
| Mang2 | **The most appropriate management options are:**   - **Admit the patient to the hospital** - **Immune globulin 1 g/kg IV** - **Platelet transfusion 6 U IV** - **Prednisone 1 mg/kg po**   **Rationale:**   1. **Admit the patient to the hospital:**   **Required for monitoring and treatment due to thrombocytopenia with mucocutaneous bleeding (petechiae, ecchymosis). Hospitalization ensures rapid intervention if bleeding worsens.**   2. **Immune globulin (IVIG):**   **First-line therapy for acute ITP to rapidly increase platelet counts by modulating immune-mediated destruction.**   3. **Platelet transfusion:**   **Indicated here due to active mucocutaneous bleeding (ecchymosis, petechiae) and thrombocytopenia. While platelet survival may be short-lived in ITP, transfusion provides temporary hemostatic support.**   4. **Prednisone:**   **First-line therapy to suppress autoimmune platelet destruction. Started concurrently with IVIG for sustained effect.**  **Excluded Options:**   - **Alteplase, Fresh frozen plasma, PCC:**   **Not indicated; PT/PTT are normal, and there is no evidence of thrombosis or coagulopathy.**   - **Lorazepam, Phenytoin:**   **No clinical indication (e.g., seizures, agitation) in this case.**   - **Splenectomy:**   **Reserved for refractory or chronic ITP, not part of initial management.**  **Key Considerations:**   - **IVIG and corticosteroids work synergistically to reduce platelet destruction.** - **Platelet transfusion is prioritized here due to active bleeding, even though transfused platelets may have a shortened lifespan in ITP.** - **Admission ensures close monitoring for complications (e.g., intracranial hemorrhage) and response to therapy.**   **Immediate Next Steps:**   1. **Administer IVIG and prednisone.** 2. **Transfuse platelets.** 3. **Proceed with bone marrow biopsy to confirm ITP and exclude marrow pathology.** | **Most Appropriate Management Options:** ✅ **Admit the patient to the hospital** ✅ **Immune globulin 1 g/kg IV** ✅ **Platelet transfusion 6 U IV** ✅ **Prednisone 1 mg/kg PO**  **Rationale:**   1. **Admit the patient to the hospital**:   **Critical for monitoring and urgent intervention** due to intracerebral hemorrhage (ICH) and severe thrombocytopenia.   2. **Immune globulin (IVIG)**:   **First-line emergency treatment for ITP** with active bleeding. Rapidly increases platelet counts by blocking antibody-mediated destruction.   3. **Platelet transfusion**:   **Urgent for life-threatening bleeding (ICH)**. While platelet survival is reduced in ITP, transfusions are critical to temporarily stabilize bleeding.   4. **Prednisone**:   **Standard first-line therapy for ITP**. Oral corticosteroids (e.g., prednisone) suppress autoantibody production. IV methylprednisolone may be preferred in acute ICH, but oral prednisone is still appropriate if IV is unavailable.  **Excluded Options:**   - **Alteplase**: Contraindicated in hemorrhage (thrombolytics worsen bleeding). - **Fresh frozen plasma (FFP)**: No coagulopathy (normal PT/PTT); ineffective for thrombocytopenia. - **Lorazepam**: No indication (no seizures or agitation documented). - **Phenytoin**: Not routinely recommended unless seizures occur (ICH location/severity may dictate need). - **Prothrombin complex concentrate (PCC)**: Irrelevant (no anticoagulant use or clotting factor deficiency). - **Splenectomy**: Second-line therapy for chronic ITP, not acute management.   **Key Priorities:**   1. **Rapidly increase platelet count** to >50,000/µL to prevent ICH expansion. 2. **Monitor neurologic status** (serial imaging if deterioration occurs). 3. **Transition to long-term ITP therapy** (e.g., thrombopoietin receptor agonists, rituximab) if refractory to steroids/IVIG.   **Conclusion**: Immediate platelet support (IVIG + transfusion) and immunosuppression (steroids) are critical in this life-threatening scenario. Hospitalization ensures close monitoring and timely intervention. | **Appropriate Management Options:**   1. **Admit the patient to the hospital**   **Rationale**: Critical due to **intracranial hemorrhage risk** and active bleeding (petechiae, ecchymosis). Requires close monitoring and urgent treatment.   2. **Immune globulin 1 g/kg IV**   **Rationale**: **IVIG** rapidly increases platelet counts in ITP by blocking antibody-mediated destruction. First-line for severe thrombocytopenia with bleeding.   3. **Platelet transfusion 6 U IV**   **Rationale**: Indicated for **life-threatening bleeding** (e.g., prior intracranial hemorrhage) despite transient efficacy in ITP. Temporarily stabilizes platelets to prevent fatal hemorrhage.   4. **Prednisone 1 mg/kg po**   **Rationale**: **First-line therapy for ITP**. Suppresses immune-mediated platelet destruction. Oral prednisone is standard, though IV methylprednisolone may be used in critical cases.  **Excluded Options**:   - **Alteplase, Fresh frozen plasma, PCC**: Contraindicated (no thromboembolic event, normal PT/PTT, no anticoagulant use). - **Lorazepam, Phenytoin**: No seizure activity or agitation. - **Splenectomy**: Reserved for refractory ITP after failed medical therapy.   **Answer**:   - Admit the patient to the hospital - Immune globulin 1 g/kg IV - Platelet transfusion 6 U IV - Prednisone 1 mg/kg po |
| Case 18 |  |  |  |
| Diff1 | **Diagnoses that CANNOT be excluded based on history and physical examination:**   - **Diabetes mellitus** - **Drug ingestion** - **Hypercalcemia** - **Hyperthyroidism** - **Hypothyroidism** - **Diabetes insipidus** - **Urinary tract infection (UTI)** - **Psychogenic polydipsia**   **Rationale:**   1. **Diabetes Mellitus:**   **Classic symptoms of polyuria, polydipsia, nausea, and lethargy strongly suggest hyperglycemia. Family history of diabetes and autoimmune Graves’ disease further support this. *Cannot exclude without glucose/HbA1c*.**   2. **Drug Ingestion:**   **No overt signs of toxidrome, but reliance on self-reported history. *Cannot exclude without toxicology screening*.**   3. **Hypercalcemia:**   **Symptoms overlap (polyuria, pruritus, weakness), and she takes vitamin D. *Cannot exclude without serum calcium*.**   4. **Hyperthyroidism:**   **History of Graves’ disease treated with radioactive iodine. Potential for iatrogenic hyperthyroidism if over-replaced with levothyroxine. *Cannot exclude without TSH/free T4*.**   5. **Hypothyroidism:**   **Current levothyroxine use; symptoms (lethargy, depression) could indicate under-replacement or non-compliance. *Cannot exclude without TSH*.**   6. **Diabetes Insipidus:**   **Polyuria/polydipsia without confirmed hyperglycemia. Central or nephrogenic DI remains possible. *Cannot exclude without urine osmolality/serum sodium*.**   7. **Urinary Tract Infection:**   **Asymptomatic bacteriuria or atypical presentation (e.g., polyuria without dysuria) is possible. *Cannot exclude without urinalysis/culture*.**   8. **Psychogenic Polydipsia:**   **Excessive water intake without obvious cause. Mood changes raise suspicion, though no prior psychiatric history. *Cannot exclude without labs to rule out osmotic drivers*.**  **Key Exclusions Not Listed:**   - **Thyroid storm (normal vital signs, no agitation/tremors).** - **Gestational diabetes (last menstrual period 3 weeks ago; pregnancy test needed).**   **Next Steps:**   - **Urgent labs: Blood glucose, electrolytes (including calcium), TSH/free T4, urine osmolality, urinalysis.** - **Toxicology screen if suspicion persists.** - **Diabetes education and insulin initiation if hyperglycemia confirmed.** | **Diagnoses that CANNOT be excluded based on history and physical examination:** ✅ **Diabetes mellitus** ✅ **Drug ingestion** ✅ **Hypercalcemia** ✅ **Hyperthyroidism** ✅ **Hypothyroidism** ✅ **Diabetes insipidus** ✅ **Urinary tract infection** ✅ **Psychogenic polydipsia**  **Rationale:**   1. **Diabetes Mellitus**:   Polyuria/polydipsia and family history of type 2 DM are concerning. No glucose/HbA1c results available to rule out.   2. **Drug Ingestion**:   Patient denies drug use, but no toxicology screen performed. Symptoms like nausea/mood changes could overlap with toxicity.   3. **Hypercalcemia**:   Symptoms (polyuria, nausea, pruritus, neuropsychiatric changes) strongly suggest hypercalcemia, but serum calcium/PTH levels are pending.   4. **Hyperthyroidism**:   History of Graves’ disease treated with radioactive iodine. Current levothyroxine dose (125 mcg) could theoretically cause iatrogenic hyperthyroidism; TSH/free T4 not checked.   5. **Hypothyroidism**:   On levothyroxine for post-RAI hypothyroidism, but symptoms (lethargy, depression) could indicate undertreatment. TSH levels not available.   6. **Diabetes Insipidus**:   Polyuria/polydipsia fits, but central vs. nephrogenic DI requires urine/serum osmolality testing.   7. **Urinary Tract Infection**:   No dysuria or fever, but asymptomatic bacteriuria is possible. Urinalysis/culture not performed.   8. **Psychogenic Polydipsia**:   Excessive water intake could explain polyuria, but pruritus/nausea are atypical. Requires serum/urine osmolality to differentiate from DI.  **Key Missing Data to Exclude Diagnoses:**   - **Labs**: Serum calcium, PTH, glucose/HbA1c, TSH/free T4, urine osmolality. - **Imaging/Studies**: Thyroid ultrasound (if hyperparathyroidism suspected), sestamibi scan. - **Urine Tests**: Urinalysis, culture.   **Conclusion**: **All listed diagnoses remain possible** until further testing is completed. The history and physical exam lack sufficient specificity to exclude any of these conditions definitively. | **Diagnoses that CANNOT be excluded based on history and physical examination:**   1. **Diabetes Mellitus**:   **Rationale**: Polyuria/polydipsia are classic symptoms. No lab results (e.g., blood glucose, HbA1c) to rule out DM.   2. **Drug Ingestion**:   **Rationale**: Patient denies drug use, but symptoms like nausea and polyuria are nonspecific. A toxicology screen is needed for confirmation.   3. **Hypercalcemia**:   **Rationale**: Symptoms (nausea, polyuria, pruritus, muscle weakness) and history of Graves’ disease treated with RAI (risk of parathyroid dysfunction) strongly suggest hypercalcemia, but labs (serum calcium, PTH) are required for confirmation.   4. **Hypothyroidism**:   **Rationale**: Patient is on levothyroxine, but symptoms (lethargy, depression, muscle weakness) could indicate under-replacement. TSH levels are needed to assess thyroid function.   5. **Diabetes Insipidus (DI)**:   **Rationale**: Polyuria/polydipsia may occur in DI. Hypercalcemia can mimic nephrogenic DI, but urine osmolality and serum sodium are required to differentiate.   6. **Urinary Tract Infection (UTI)**:   **Rationale**: Asymptomatic UTIs can present with polyuria. Urinalysis and culture are needed to exclude infection.   7. **Psychogenic Polydipsia**:   **Rationale**: Excessive water intake can cause polyuria, but other symptoms (nausea, pruritus) are atypical. Labs (serum osmolality, urine output) are necessary to confirm.  **Diagnosis that CAN be excluded**:   - **Hyperthyroidism**:   **Rationale**: Patient is on levothyroxine for hypothyroidism, with no signs of over-replacement (normal pulse, stable weight, absence of tremor/heat intolerance).  **Answer**:   - Diabetes mellitus - Drug ingestion - Hypercalcemia - Hypothyroidism - Diabetes insipidus - Urinary tract infection - Psychogenic polydipsia |
| Diag1 | **Most Appropriate Studies to Obtain:**   1. **Random blood glucose** 2. **Serum electrolyte concentrations** 3. **Serum calcium and phosphate** 4. **Serum TSH and free T4** 5. **Serum urea and creatinine** 6. **Serum and urine osmolality**   **Rationale:**   - **Random blood glucose:**   **Critical to confirm/exclude diabetes mellitus (polyuria, polydipsia, nausea).**   - **Serum electrolytes:**   **Assess for hypokalemia (common in DKA), hypernatremia (diabetes insipidus), or metabolic derangements.**   - **Serum calcium and phosphate:**   **Evaluate for hypercalcemia (symptoms: polyuria, pruritus, nausea) given her vitamin D supplementation.**   - **TSH and free T4:**   **Rule out thyroid dysfunction (history of Graves’ disease treated with radioactive iodine; symptoms of lethargy/mood changes may indicate hypo-/hyperthyroidism).**   - **Serum urea and creatinine:**   **Assess renal function and hydration status (polyuria may lead to dehydration or renal impairment).**   - **Serum and urine osmolality:**   **Differentiate diabetes insipidus (low urine osmolality, high serum osmolality) from psychogenic polydipsia (low serum and urine osmolality).**  **Less Relevant or Unnecessary Studies:**   - **CBC: No signs of infection or anemia to prioritize this.** - **CRP: No evidence of acute inflammation or infection.** - **LFTs: No clinical clues for liver disease (e.g., jaundice, right upper quadrant pain).** - **CT scan: Unlikely malignancy or structural cause (e.g., hypercalcemia) without suggestive symptoms (weight loss, bone pain, etc.).**   **Next Steps:**   - **If glucose is elevated, add HbA1c and serum ketones.** - **If hypercalcemia is confirmed, check PTH and vitamin D levels.** - **If urine osmolality is low, consider water deprivation test for diabetes insipidus.** | **Most Appropriate Studies to Obtain at This Time:** ✅ **Random blood glucose** ✅ **Serum electrolyte concentrations** ✅ **Serum calcium and phosphate** ✅ **Serum TSH and free T4** ✅ **Serum urea and creatinine** ✅ **Serum and urine osmolality**  **Rationale:**   1. **Random blood glucose**:   Essential to evaluate for **diabetes mellitus**, a key differential for polyuria/polydipsia.   2. **Serum electrolytes (Na⁺, K⁺)**:   Polyuria and thirst may reflect electrolyte imbalances (e.g., hypernatremia in diabetes insipidus, hypokalemia in hyperaldosteronism).   3. **Serum calcium and phosphate**:   Critical for suspected **hypercalcemia** (e.g., primary hyperparathyroidism), which explains nausea, polyuria, and pruritus.   4. **Serum TSH and free T4**:   Required to assess thyroid function. Her history of Graves’ disease and levothyroxine use raises concern for **hypothyroidism** (under-replacement) or **hyperthyroidism** (over-replacement), both of which can mimic her symptoms.   5. **Serum urea and creatinine**:   Evaluates renal function; hypercalcemia and dehydration can impair kidney function.   6. **Serum and urine osmolality**:   Differentiates **diabetes insipidus** (low urine osmolality, high serum osmolality) from **psychogenic polydipsia** (low serum and urine osmolality).  **Less Relevant or Unnecessary Studies:**   - **CBC**: No signs of infection or anemia to warrant urgency. - **CRP**: No evidence of inflammation or infection. - **LFTs**: Absent jaundice, right upper quadrant pain, or risk factors for liver disease. - **CT scan of chest/abdomen/pelvis**: Premature without evidence of malignancy (e.g., weight loss, masses).   **Next Steps**:   - If hypercalcemia is confirmed, measure **PTH** to confirm primary hyperparathyroidism. - If diabetes insipidus is suspected, proceed with a **water deprivation test**. - If thyroid dysfunction is found, adjust levothyroxine dose accordingly. | **Most Appropriate Studies to Obtain:**   1. **Random Blood Glucose**:   **Rationale**: To evaluate for diabetes mellitus, as polyuria/polydipsia are hallmark symptoms.   2. **Serum Electrolyte Concentrations** (including sodium, potassium):   **Rationale**: Hypercalcemia (suspected) can cause electrolyte imbalances. Hyponatremia or hypernatremia may support diabetes insipidus (DI) or psychogenic polydipsia.   3. **Serum Calcium and Phosphate**:   **Rationale**: Critical to confirm hypercalcemia (suspected in this case) and assess for primary hyperparathyroidism (e.g., elevated calcium with low/normal phosphate).   4. **Serum TSH and Free T4**:   **Rationale**: Patient has a history of hypothyroidism treated with levothyroxine. Symptoms like lethargy and depression could indicate under-replacement or over-replacement.   5. **Serum Urea and Creatinine**:   **Rationale**: Assess renal function, as hypercalcemia or diabetes insipidus can impair kidney function.   6. **Serum and Urine Osmolality**:   **Rationale**: Differentiate between diabetes insipidus (low urine osmolality despite high serum osmolality) and psychogenic polydipsia (low serum osmolality with dilute urine).  **Studies Less Likely to Be Helpful**:   - **CBC/CRP/LFTs**: No signs of infection, inflammation, or liver dysfunction to justify these. - **CT Scan**: Not indicated without evidence of malignancy or structural disease.   **Answer**:   - Random blood glucose - Serum electrolyte concentrations - Serum calcium and phosphate - Serum TSH and free T4 - Serum urea and creatinine - Serum and urine osmolality |
| Diff2 | **Most Likely Diagnoses:**   1. **Hypercalcemia** 2. **Nephrogenic diabetes insipidus**   **Rationale:**  **1. Hypercalcemia**   - **Key Finding:**   **Severe hypercalcemia (total calcium = 13.6 mg/dL 3.4mmol/L3.4*mmol*/*L*, normal: 8.5–10.2 mg/dL).**   - **Clinical Relevance:**   **Hypercalcemia directly explains polyuria (via nephrogenic diabetes insipidus), nausea, pruritus, and lethargy.**  **Likely causes: Primary hyperparathyroidism (supported by low phosphate) or vitamin D toxicity (given the patient’s vitamin D supplementation).**  **2. Nephrogenic Diabetes Insipidus (DI)**   - **Key Findings:**   **Inappropriately dilute urine (urine osmolality = 210 mOsmol/kg) despite normal serum osmolality (285 mOsmol/kg).**  **Hypercalcemia impairs renal tubular response to antidiuretic hormone (ADH), leading to impaired urine concentrating ability.**   - **Why Not Central DI?**   **Central DI typically causes very low urine osmolality (<150 mOsmol/kg) and is not linked to hypercalcemia.**  **Excluded Diagnoses:**   - **Central Diabetes Insipidus:**   **No evidence of pituitary dysfunction (e.g., normal serum osmolality, lack of head trauma/tumor history).**   - **Diabetes Mellitus:**   **Normal random blood glucose (112 mg/dL) excludes this.**   - **Psychogenic Polydipsia:**   **Serum osmolality is normal (285 mOsmol/kg), whereas psychogenic polydipsia typically causes low serum osmolality due to excessive water intake.**  **Underlying Mechanism:**   - **Hypercalcemia → Nephrogenic DI:**   **Calcium deposition in the renal tubules disrupts ADH signaling, reducing water reabsorption and causing polyuria.**  **This explains the patient’s polydipsia (compensatory response to polyuria) and mild hyponatremia (dilution from excessive water intake).**  **Next Steps: Confirm the cause of hypercalcemia (e.g., PTH, vitamin D levels) and initiate urgent treatment (e.g., IV fluids, bisphosphonates).** | **Most Likely Diagnoses:** ✅ **Hypercalcemia** ✅ **Nephrogenic diabetes insipidus (NDI)**  **Rationale:**   1. **Hypercalcemia**:   **Severe elevation in serum calcium** (13.6 mg/dL) directly explains:  **Polyuria/polydipsia**: Hypercalcemia impairs renal concentrating ability by reducing the kidney’s response to ADH.  **Nausea, pruritus, and neuropsychiatric symptoms**.  **Hypophosphatemia** (1.9 mg/dL) supports **primary hyperparathyroidism** as the underlying cause.   2. **Nephrogenic Diabetes Insipidus (NDI)**:   **Urine osmolality (210 mOsmol/kg)** is inappropriately low for **serum osmolality (285 mOsmol/kg)**.  Hypercalcemia damages renal tubules, causing resistance to ADH. This is classic for **hypercalcemia-induced NDI**.  **Ruled Out Diagnoses:**   - **Central Diabetes Insipidus (CDI)**:   No evidence of pituitary dysfunction (e.g., normal TSH/free T4, no head trauma/tumor history).  Hypercalcemia is a more plausible explanation for NDI.   - **Diabetes Mellitus**:   **Normal random blood glucose (112 mg/dL)** excludes this.   - **Psychogenic Polydipsia**:   Serum osmolality is normal (285 mOsmol/kg), which would be low in primary polydipsia.  Urine osmolality is not maximally diluted (210 mOsmol/kg vs. <100 mOsmol/kg in psychogenic polydipsia).  **Clinical Correlation:**  The patient’s **hypercalcemia** (likely from primary hyperparathyroidism) is driving **nephrogenic DI**, resulting in polyuria and compensatory polydipsia. Treatment should focus on correcting hypercalcemia (e.g., IV fluids, bisphosphonates) and addressing the underlying parathyroid disorder. | **Diagnoses Best Accounting for the Patient’s Findings:**   1. **Hypercalcemia** 2. **Nephrogenic Diabetes Insipidus (DI)**   **Explanation:**  **1. Hypercalcemia**   - **Key Evidence**:   **Severely elevated serum calcium**: 13.6 mg/dL (normal: 8.5–10.2 mg/dL).  **Low phosphate**: 1.9 mg/dL (normal: 2.5–4.3 mg/dL), consistent with **primary hyperparathyroidism** (elevated PTH causes calcium retention and phosphate wasting).   - **Role in Symptoms**:   Hypercalcemia directly causes polyuria by impairing renal concentrating ability (via calcium-induced nephrogenic DI).  Lethargy, depression, and constipation are classic symptoms of hypercalcemia.  **2. Nephrogenic Diabetes Insipidus**   - **Key Evidence**:   **Low urine osmolality (210 mOsmol/kg)** despite **normal serum osmolality (285 mOsmol/kg)**.  **Normal serum sodium (136 mEq/L)**: Compensatory polydipsia prevents hypernatremia.   - **Mechanism**:   Hypercalcemia damages renal tubules, reducing responsiveness to antidiuretic hormone (ADH).  Urine remains dilute even with adequate ADH (unlike central DI, where ADH is deficient).  **Ruled Out Diagnoses:**   - **Central Diabetes Insipidus**:   Central DI would have **extremely low urine osmolality** (<150 mOsmol/kg) and **elevated serum osmolality** (>295 mOsmol/kg). This patient’s findings do not fit.   - **Diabetes Mellitus**:   Random glucose is normal (112 mg/dL). No evidence of hyperglycemia or glycosuria.   - **Psychogenic Polydipsia**:   Serum osmolality would be **low** (<275 mOsmol/kg) due to excessive water intake. This patient’s serum osmolality is normal (285 mOsmol/kg).  **Next Steps**:   - Confirm the cause of hypercalcemia: Measure **parathyroid hormone (PTH)**.   **If PTH is elevated**: Confirm primary hyperparathyroidism (e.g., parathyroid adenoma).  **If PTH is suppressed**: Evaluate for malignancy (e.g., PTHrP-secreting tumor) or vitamin D toxicity.   - Treat hypercalcemia urgently (IV fluids, bisphosphonates). Nephrogenic DI may resolve with calcium normalization.   **Answer**: Hypercalcemia and Nephrogenic Diabetes Insipidus. |
| Diff3 | **The following options should be evaluated as possible causes of the patient’s hypercalcemia:**   1. **Primary hyperparathyroidism**   **Rationale: Classic presentation of hypercalcemia with low phosphate (due to PTH-driven phosphate excretion). This is the most likely diagnosis unless proven otherwise.**   2. **Vitamin D toxicity**   **Rationale: The patient is on vitamin D supplementation. Excess vitamin D increases intestinal calcium absorption and bone resorption, leading to hypercalcemia. Low phosphate here could reflect concurrent PTH suppression (if vitamin D is the primary driver).**   3. **Bone resorption due to malignant infiltration**   **Rationale: Malignancy (e.g., multiple myeloma, breast cancer, lung cancer) can cause hypercalcemia via direct bone destruction or secretion of PTH-related peptide (PTHrP).**   4. **Paraneoplastic syndrome with cancer secreting PTH-related peptide**   **Rationale: PTHrP mimics PTH, causing hypercalcemia and hypophosphatemia. This is indistinguishable from primary hyperparathyroidism without PTH testing.**   5. **Sarcoidosis**   **Rationale: Granulomatous diseases (e.g., sarcoidosis) overproduce 1,25-(OH)2 vitamin D, leading to hypercalcemia. This should be evaluated if vitamin D levels are elevated.**   6. **Familial hypocalciuric hypercalcemia (FHH)**   **Rationale: A genetic cause of lifelong mild-to-moderate hypercalcemia with low urinary calcium excretion. While typically asymptomatic, severe hypercalcemia is rare in FHH. Urine calcium testing (e.g., calcium-to-creatinine clearance ratio) is needed to confirm/exclude.**  **Excluded Options:**   - **Addison disease: Does not cause hypercalcemia; it is associated with hyponatremia and hyperkalemia.** - **Excessive calcium consumption: Unlikely without concomitant vitamin D excess or renal dysfunction. Dietary calcium rarely causes hypercalcemia in isolation.** - **Secondary hyperparathyroidism due to chronic kidney disease (CKD): CKD typically causes hypocalcemia (not hypercalcemia) due to phosphate retention and reduced calcitriol production. The patient’s normal creatinine rules out CKD.**   **Next Steps:**   - **Measure PTH:**   **Elevated PTH confirms primary hyperparathyroidism.**  **Low/normal PTH suggests malignancy, vitamin D toxicity, or granulomatous disease.**   - **Check 25-OH vitamin D and 1,25-(OH)2 vitamin D levels:**   **Elevated 25-OH vitamin D supports vitamin D toxicity.**  **Elevated 1,25-(OH)2 vitamin D suggests sarcoidosis or other granulomatous disease.**   - **Urine calcium-to-creatinine clearance ratio: To assess for FHH (ratio <0.01 supports FHH).** - **Imaging for malignancy: Chest X-ray, CT, or PET scan if malignancy is suspected.**   **Primary hyperparathyroidism and vitamin D toxicity are the top considerations in this case, given the clinical context and lab findings.** | **The following causes should be evaluated for the patient’s hypercalcemia:** ✅ **Primary hyperparathyroidism** ✅ **Paraneoplastic syndrome with cancer secreting PTH-related peptide (PTHrp)** ✅ **Bone resorption due to malignant infiltration** ✅ **Sarcoidosis** ✅ **Familial hypocalciuric hypercalcemia (FHH)**  **Rationale:**   1. **Primary Hyperparathyroidism**:   **Key findings**: Severe hypercalcemia (13.6 mg/dL) with hypophosphatemia (1.9 mg/dL).  Low phosphate and normal creatinine favor PTH-mediated hypercalcemia.  **Next step**: Measure **intact PTH** (expected to be elevated).   2. **Paraneoplastic Syndrome (PTHrp)**:   PTH-related peptide (PTHrp) mimics PTH, causing hypercalcemia and hypophosphatemia.  Common in malignancies (e.g., squamous cell carcinoma, breast, lung).  **Distinguish from primary hyperparathyroidism** by checking **PTH levels** (low/normal in PTHrp, high in primary hyperparathyroidism).   3. **Malignancy with Bone Resorption**:   Metastatic cancer (e.g., breast, lung, multiple myeloma) can cause hypercalcemia via direct bone destruction.  **Note**: Hypophosphatemia is less common here unless mediated by PTHrp.   4. **Sarcoidosis**:   Granulomas produce excess **1,25-OH vitamin D**, increasing intestinal calcium absorption.  Typically causes hypercalcemia with **normal/high phosphate**, but phosphate can be low in some cases.  Check **ACE levels** and **25-OH/1,25-OH vitamin D levels**.   5. **Familial Hypocalciuric Hypercalcemia (FHH)**:   Genetic condition causing mild, lifelong hypercalcemia with **low urinary calcium excretion**.  **Urine calcium-to-creatinine clearance ratio <0.01** helps differentiate FHH from primary hyperparathyroidism.  **Less Likely but Possible:**   - **Vitamin D Toxicity**:   Unlikely here, as it typically causes **high phosphate** (due to vitamin D-driven phosphate absorption).  Check **25-OH vitamin D** to exclude.   - **Addison Disease**:   Rare cause of hypercalcemia (via dehydration/hemoconcentration).  Patient lacks typical Addisonian findings (e.g., hyponatremia, hyperkalemia).  **Ruled Out:**   - **Secondary Hyperparathyroidism from CKD**:   Normal creatinine (0.8 mg/dL) and low phosphate exclude CKD as a cause.   - **Excessive Calcium Consumption**:   Severe hypercalcemia (13.6 mg/dL) is unlikely from diet alone.  **Next Steps:**   1. **Lab Testing**:   **Intact PTH**, **PTHrp**, **25-OH/1,25-OH vitamin D**, **serum protein electrophoresis** (SPEP) for myeloma.  **24-hour urine calcium** (to assess for FHH).   2. **Imaging**:   **Chest X-ray/CT** (for sarcoidosis or malignancy).  **Parathyroid imaging** (sestamibi scan).   3. **Clinical Correlation**:   Screen for cancer (weight loss, smoking history, lymphadenopathy).  Evaluate for granulomatous disease (sarcoidosis, TB).  Primary hyperparathyroidism remains the **most likely diagnosis**, but malignancy and sarcoidosis must be excluded. | **Possible Causes of Hypercalcemia to Evaluate:**   1. **Primary Hyperparathyroidism**   **Key Evidence**:  Severe hypercalcemia (13.6 mg/dL) with **hypophosphatemia** (1.9 mg/dL).  Classic for primary hyperparathyroidism, where elevated PTH increases calcium reabsorption and phosphate excretion.  **Next Step**: Measure **parathyroid hormone (PTH)**. Elevated PTH confirms the diagnosis (e.g., parathyroid adenoma).   2. **Bone Resorption Due to Malignant Infiltration**   **Key Evidence**:  Hypercalcemia with hypophosphatemia can occur in malignancies (e.g., multiple myeloma, metastatic bone disease).  **Next Step**: Imaging (e.g., skeletal survey, CT scans) and serum/urine protein electrophoresis to rule out myeloma.   3. **Paraneoplastic Syndrome (PTHrP-Secreting Cancer)**   **Key Evidence**:  Tumors (e.g., squamous cell lung, breast, or renal cancers) secrete **PTH-related peptide (PTHrP)**, mimicking PTH effects (hypercalcemia, hypophosphatemia).  **Next Step**: Measure **PTHrP**; if elevated, search for occult malignancy.   4. **Familial Hypocalciuric Hypercalcemia (FHH)**   **Key Evidence**:  Genetic condition causing lifelong mild-moderate hypercalcemia with **low urinary calcium excretion**.  **Next Step**: Calculate **24-hour urine calcium** and **calcium-to-creatinine clearance ratio**; low values support FHH.   5. **Sarcoidosis**   **Key Evidence**:  Granulomas produce excess **1,25-OH vitamin D**, leading to hypercalcemia. However, this typically causes **hyperphosphatemia** (unlike this patient’s hypophosphatemia).  **Next Step**: Check **serum ACE levels** and **chest imaging** for granulomas.   6. **Vitamin D Toxicity**   **Key Evidence**:  Excess vitamin D increases intestinal calcium absorption. However, this usually causes **hyperphosphatemia** (not seen here).  **Next Step**: Measure **25-OH vitamin D** and **1,25-OH vitamin D levels** to assess for excess.  **Less Likely Causes in This Case:**   - **Addison Disease**: Rarely causes hypercalcemia; typically associated with hyponatremia/hyperkalemia (absent here). - **Excessive Calcium Consumption**: Unlikely without elevated phosphate or renal dysfunction (e.g., milk-alkali syndrome). - **Secondary Hyperparathyroidism (CKD)**: Creatinine is normal (0.8 mg/dL), ruling out advanced CKD.   **Answer**: **Primary Hyperparathyroidism**, **Bone Resorption Due to Malignant Infiltration**, **Paraneoplastic Syndrome (PTHrP)**, **Familial Hypocalciuric Hypercalcemia**, **Sarcoidosis**, and **Vitamin D Toxicity** should all be evaluated. |
| Diag2 | **The most appropriate studies to establish the diagnosis in this case are:**   1. **Serum parathyroid hormone (PTH) level**   **Rationale: Critical to differentiate PTH-dependent (e.g., primary hyperparathyroidism) from PTH-independent (e.g., malignancy, vitamin D toxicity) causes of hypercalcemia.**   2. **Serum total vitamin D level**   **Rationale: The patient is on vitamin D supplementation, and toxicity could directly explain hypercalcemia. Elevated 25-OH vitamin D supports this diagnosis.**   3. **Urine calcium excretion ratio (calcium-to-creatinine clearance ratio)**   **Rationale: Helps distinguish primary hyperparathyroidism (urine calcium >0.02) from familial hypocalciuric hypercalcemia (FHH) (ratio <0.01).**   4. **Chest x-ray**   **Rationale: Evaluates for lung cancer (a common cause of paraneoplastic hypercalcemia) or sarcoidosis (bilateral hilar lymphadenopathy).**   5. **Serum angiotensin-converting enzyme (ACE)**   **Rationale: Elevated in sarcoidosis, which causes hypercalcemia via excess 1,25-(OH)2 vitamin D production.**   6. **Serum and urine protein electrophoresis (SPEP/UPEP)**   **Rationale: Screens for multiple myeloma, a malignancy associated with hypercalcemia due to bone resorption.**   7. **Sestamibi scintigraphy scan of the neck**   **Rationale: Localizes parathyroid adenomas if primary hyperparathyroidism is confirmed (e.g., elevated PTH).**  **Excluded Options:**   - **Serum IGF-1 level: Not relevant unless acromegaly is suspected (no clinical clues here).** - **Serum prolactin level: Unnecessary unless pituitary dysfunction (e.g., MEN syndrome) is suspected.** - **24-hour urine calcium: Replaced by the quicker urine calcium-to-creatinine ratio in most cases.** - **Water deprivation test: The patient’s polyuria is explained by hypercalcemia-induced nephrogenic diabetes insipidus.** - **MRI of the pituitary gland: Not indicated unless central diabetes insipidus or MEN syndrome is suspected.** - **Ultrasonography of the neck: Less sensitive than sestamibi for parathyroid adenoma localization.**   **Next Steps:**   - **If PTH is elevated, proceed with sestamibi scan or neck ultrasound to localize a parathyroid adenoma.** - **If PTH is low/normal, focus on malignancy (SPEP/UPEP, chest x-ray) or vitamin D/granulomatous disease (vitamin D levels, ACE).** - **Confirm sarcoidosis with biopsy or elevated ACE/1,25-(OH)2 vitamin D if suspected.**   **Key Priority: Start with PTH and vitamin D levels, as these will guide further testing.** | **Most Appropriate Studies to Evaluate Hypercalcemia:** ✅ **Serum parathyroid hormone (PTH) level** ✅ **24-hour urine calcium** ✅ **Urine calcium excretion ratio (calcium-creatinine clearance ratio)** ✅ **Serum angiotensin-converting enzyme (ACE)** ✅ **Chest X-ray** ✅ **Ultrasonography of the neck** ✅ **Sestamibi scintigraphy scan of the neck** ✅ **Serum and urine protein electrophoresis** ✅ **Serum total vitamin D level**  **Rationale:**   1. **Serum PTH**:   **Critical first step** to differentiate **primary hyperparathyroidism** (elevated PTH) from **PTH-independent causes** (e.g., malignancy, sarcoidosis, FHH).   2. **24-hour urine calcium** and **Urine calcium excretion ratio**:   **Familial hypocalciuric hypercalcemia (FHH)** is ruled out by **low urinary calcium excretion** (calcium-creatinine clearance ratio <0.01).  High urine calcium supports **primary hyperparathyroidism** or other causes.   3. **Serum ACE** and **Chest X-ray**:   Evaluate for **sarcoidosis**, which causes hypercalcemia via excess 1,25-OH vitamin D production.   4. **Ultrasonography of the neck** and **Sestamibi scintigraphy**:   Localize parathyroid adenomas if **primary hyperparathyroidism** is confirmed (elevated PTH).   5. **Serum and urine protein electrophoresis**:   Screen for **multiple myeloma** or other plasma cell dyscrasias causing hypercalcemia via bone resorption.   6. **Serum total vitamin D level**:   Exclude **vitamin D toxicity** (though hypophosphatemia makes this less likely, it is still prudent).  **Studies Not Indicated:**   - **Serum IGF-1/prolactin/MRI pituitary**:   No evidence of pituitary dysfunction (normal TSH/free T4, no central DI symptoms).   - **Water deprivation test**:   Polyuria/polydipsia is explained by hypercalcemia-induced nephrogenic DI; dehydration could worsen hypercalcemia.  **Clinical Workflow:**   1. **Confirm PTH level**:   **Elevated PTH**: Proceed with **neck imaging** (ultrasound/sestamibi) for parathyroid adenoma.  **Low/normal PTH**: Evaluate for **malignancy** (imaging, PTHrp) or **sarcoidosis** (ACE, chest X-ray).   2. **Urine calcium**:   Low excretion (ratio <0.01) suggests **FHH**; genetic testing may follow.   3. **Vitamin D levels**:   Elevated 1,25-OH vitamin D suggests **granulomatous disease** (sarcoidosis) or lymphoma.  **Primary hyperparathyroidism** remains the leading diagnosis, but malignancy and sarcoidosis must be excluded given the severity of hypercalcemia. | **Most Appropriate Studies to Establish the Diagnosis:**   1. **Serum Parathyroid Hormone (PTH) Level**   **Rationale**: Critical to distinguish primary hyperparathyroidism (elevated PTH) from PTH-independent causes (e.g., malignancy, vitamin D toxicity).   2. **24-Hour Urine Calcium** and **Urine Calcium Excretion Ratio**   **Rationale**: Differentiates **familial hypocalciuric hypercalcemia (FHH)** (low urine calcium) from primary hyperparathyroidism (normal/elevated urine calcium).   3. **Serum Angiotensin-Converting Enzyme (ACE)**   **Rationale**: Elevated in sarcoidosis, which can cause hypercalcemia via excess 1,25-OH vitamin D production.   4. **Chest X-Ray**   **Rationale**: Evaluates for lung cancer (PTHrP-secreting tumors) or sarcoidosis (bilateral hilar lymphadenopathy).   5. **Serum Total Vitamin D Level**   **Rationale**: Assesses for vitamin D toxicity (elevated 25-OH vitamin D) or granulomatous diseases (elevated 1,25-OH vitamin D).   6. **Ultrasonography of the Neck** and/or **Sestamibi Scintigraphy Scan**   **Rationale**: Localizes parathyroid adenomas if primary hyperparathyroidism is confirmed (elevated PTH).   7. **Serum and Urine Protein Electrophoresis**   **Rationale**: Screens for multiple myeloma, which causes hypercalcemia via bone resorption.  **Less Relevant Options**:   - **Water Deprivation Test**: Not urgent, as nephrogenic DI is secondary to hypercalcemia. - **Serum Prolactin/IGF-1/MRI Pituitary**: No evidence of pituitary dysfunction (central DI ruled out earlier).   **Answer**: **Serum PTH**, **24-hour urine calcium**, **urine calcium excretion ratio**, **serum ACE**, **chest X-ray**, **serum total vitamin D**, **ultrasonography of the neck**, **sestamibi scintigraphy**, and **serum/urine protein electrophoresis**. |
| Dx | **The most likely cause of hypercalcemia in this patient is:**   1. **Primary hyperparathyroidism due to right inferior parathyroid adenoma**   **Key Supporting Evidence:**  **Markedly elevated PTH (254 pg/mL), confirming PTH-dependent hypercalcemia.**  **Localized parathyroid lesion on sestamibi scan and neck ultrasound.**  **Elevated 24-hour urine calcium (11 mmol/24h) and urine calcium excretion ratio (0.09), ruling out familial hypocalciuric hypercalcemia (FHH).**  **Normal vitamin D, ACE, and protein electrophoresis exclude alternative causes (e.g., vitamin D toxicity, sarcoidosis, myeloma).**  **Excluded Options:**   - **Ectopic parathyroid adenoma: The lesion is localized to the right inferior parathyroid (typical location), making ectopic adenoma unlikely.** - **Lymphoma/Small-cell lung cancer: Normal chest x-ray and elevated PTH (malignancy-induced hypercalcemia typically suppresses PTH via PTHrP).** - **Multiple myeloma: Normal serum/urine protein electrophoresis.** - **Sarcoidosis: Normal ACE and vitamin D levels.** - **Secondary hyperparathyroidism: Vitamin D levels are normal (not deficient).** - **Vitamin D toxicity: Vitamin D levels within normal range.**   **Additional Consideration:**   - **Parathyroid carcinoma is rare but possible. However, adenoma is far more common, and there is no reported evidence of invasion or metastasis (e.g., palpable neck mass, vocal cord paralysis) to suggest cancer.**   **Conclusion: The findings overwhelmingly support primary hyperparathyroidism caused by a right inferior parathyroid adenoma. Surgical removal (parathyroidectomy) is the definitive treatment.** | **Answer:** ✅ **Primary hyperparathyroidism due to right inferior parathyroid adenoma or cancer**  **Key Evidence Supporting This Diagnosis:**   1. **Markedly Elevated PTH (254 pg/mL)**:   PTH is **inappropriately elevated** in the setting of hypercalcemia, confirming **primary hyperparathyroidism**.   2. **Sestamibi Scan Localization**:   **Increased tracer uptake in the right inferior parathyroid lesion** is classic for a **parathyroid adenoma** (95% of cases).  Parathyroid carcinoma is rare (<1% of cases) and typically presents with **extremely high calcium (>14 mg/dL)**, palpable neck mass, or invasive features on imaging.   3. **Elevated 24-Hour Urine Calcium (11 mmol/24h)**:   Excludes **familial hypocalciuric hypercalcemia (FHH)**, where urine calcium is low (ratio <0.01).   4. **Normal ACE, Chest X-ray, and Protein Electrophoresis**:   Rules out **sarcoidosis**, **lymphoma**, **multiple myeloma**, and **malignancy-associated hypercalcemia** (e.g., PTHrp-secreting tumors).   5. **Normal Vitamin D Level**:   Excludes **vitamin D toxicity** or granulomatous diseases (e.g., sarcoidosis).  **Ruled Out Causes:**   - **Ectopic Parathyroid Adenoma**:   The adenoma is localized to the **right inferior parathyroid gland** (a typical anatomical location), making ectopic adenoma unlikely.   - **Lymphoma/Multiple Myeloma**:   Normal serum/urine protein electrophoresis and chest X-ray.   - **Sarcoidosis**:   Normal ACE and chest X-ray.   - **Secondary Hyperparathyroidism**:   Vitamin D level is normal (22.8 ng/mL), and no evidence of chronic kidney disease.   - **Small-Cell Lung Cancer (PTHrp)**:   PTH is elevated, whereas PTHrp-mediated hypercalcemia suppresses PTH.   - **Vitamin D Toxicity**:   Total vitamin D is within normal range.  **Conclusion:**  The findings overwhelmingly support **primary hyperparathyroidism from a parathyroid adenoma**. While parathyroid carcinoma cannot be entirely excluded without histopathology, it is far less likely given the absence of severe hypercalcemia (>14 mg/dL) or invasive features.  **Next Step**: Referral for **parathyroidectomy** with intraoperative PTH monitoring to confirm adenoma resection. | **Answer:** **Primary hyperparathyroidism due to right inferior parathyroid adenoma or cancer**  **Key Evidence Supporting the Diagnosis:**   1. **Elevated PTH (254 pg/mL)**:   Inappropriately high for hypercalcemia, confirming **autonomous PTH secretion** (classic for primary hyperparathyroidism).   2. **Imaging Findings**:   **Sestamibi scan**: Increased uptake in the **right inferior parathyroid lesion**, localizing the source of excess PTH.  **Neck ultrasound**: Nodule in the right lower pole of the thyroid, consistent with a parathyroid adenoma.   3. **Exclusion of Other Causes**:   **Normal serum/urine protein electrophoresis**: Rules out multiple myeloma.  **Normal ACE (38 U/L) and chest X-ray**: Excludes sarcoidosis.  **Normal vitamin D levels (22.8 ng/mL)**: No evidence of vitamin D toxicity.  **Elevated 24-hour urine calcium (11 mmol/24h)**: Excludes familial hypocalciuric hypercalcemia (FHH).  **Elevated PTH excludes PTHrP-mediated hypercalcemia** (e.g., small-cell lung cancer).  **Why Other Options Are Unlikely:**   - **Ectopic parathyroid adenoma**: The lesion is localized to the **right inferior parathyroid** (typical location), making ectopic adenoma unnecessary to invoke. - **Lymphoma/Sarcoidosis**: Normal ACE, chest X-ray, and calcium excretion pattern. - **Multiple myeloma**: Normal protein electrophoresis. - **Secondary hyperparathyroidism**: Normal vitamin D and hypercalcemia (secondary HPT causes hypocalcemia or normocalcemia). - **Small-cell lung cancer**: PTH is elevated, not suppressed (PTHrP-secreting tumors suppress PTH). - **Vitamin D toxicity**: Normal vitamin D levels.   **Note on Parathyroid Cancer**: While **parathyroid carcinoma** is rare, it cannot be entirely excluded without histopathology. However, adenoma is far more likely (>95% of cases). Cancer is typically associated with severe hypercalcemia (e.g., >14 mg/dL), palpable neck mass, or invasive features on imaging.  **Conclusion**: The most likely diagnosis is **primary hyperparathyroidism caused by a right inferior parathyroid adenoma**, supported by biochemical and imaging evidence. |
| Mang | **Most Appropriate Management Steps:**   - **Right inferior parathyroidectomy** - **Intravenous 0.9% saline every 6 hours (if hypercalcemia requires acute management)** - **Pamidronate 60 to 90 mg IV infusion (if severe hypercalcemia or preoperative calcium lowering is needed)**   **Key Explanations:**   1. **Right inferior parathyroidectomy:**   **Definitive treatment for localized parathyroid adenoma confirmed by imaging (sestamibi scan and ultrasound). Curative in >95% of cases.**  **Indicated due to elevated urine calcium (11 mmol/24h), meeting surgical criteria (24-hour urine calcium >400 mg/day).**   2. **Intravenous 0.9% saline:**   **Used for volume expansion to enhance renal calcium excretion in symptomatic or severe hypercalcemia.**   3. **Pamidronate:**   **A bisphosphonate that inhibits bone resorption, lowering serum calcium. Useful if preoperative calcium reduction is necessary or for osteoporosis management.**  **Why Other Options Are Not Appropriate:**   - **Watch and wait: Contraindicated due to elevated urine calcium (>400 mg/day), which is a surgical criterion.** - **Desmopressin, Furosemide, Prednisolone, Calcitonin, Hemofiltration: No role in chronic or definitive management of primary hyperparathyroidism.** - **3-and-a-half glands parathyroidectomy: Reserved for parathyroid hyperplasia (e.g., secondary/tertiary hyperparathyroidism), not a solitary adenoma.** - **Radiotherapy: Not effective for benign parathyroid adenomas.**   **Final Answer: Right inferior parathyroidectomy *(Intravenous saline and pamidronate are context-dependent and may be used adjunctively if acute hypercalcemia management is required.)*** | **Answer:** ✅ **Right inferior parathyroidectomy** ✅ **Intravenous 0.9% saline every 6 hours** (if acute/severe hypercalcemia present) ✅ **Pamidronate 60 to 90 mg IV infusion** (if severe hypercalcemia requires urgent lowering preoperatively)  **Key Management Steps:**   1. **Right Inferior Parathyroidectomy**:   **Definitive treatment** for confirmed parathyroid adenoma.  Curative in >95% of cases.  Preoperative localization (sestamibi scan and ultrasound) supports targeted surgery.   2. **Intravenous 0.9% Saline**:   First-line for **acute/severe hypercalcemia** (e.g., calcium >13 mg/dL or symptomatic hypercalcemia).  Hydration increases renal calcium excretion and stabilizes the patient preoperatively.   3. **Pamidronate (Bisphosphonate)**:   Used for **urgent calcium lowering** if severe hypercalcemia (e.g., calcium >14 mg/dL) or surgery is delayed.  Inhibits bone resorption, reducing calcium levels within 24-48 hours.  **Inappropriate Options:**   - **Watch and Wait**:   Contraindicated here. Surgical criteria are met: **hypercalciuria (11 mmol/24h)**, elevated PTH, and confirmed adenoma.   - **Desmopressin/Furosemide/Prednisolone/Calcitonin/Radiotherapy**:   No role in chronic management of primary hyperparathyroidism.   - **3.5-Gland Parathyroidectomy**:   Reserved for **parathyroid hyperplasia** (e.g., renal failure), not solitary adenoma.   - **Hemofiltration**:   Only for refractory, life-threatening hypercalcemia (e.g., calcium >18 mg/dL with coma).  **Clinical Context:**   - **Surgery is mandatory** unless contraindicated. - **IV saline and bisphosphonates** are adjuncts for severe hypercalcemia but do not replace surgery. - Postoperatively, monitor for **hypocalcemia** (transient "hungry bone syndrome") and ensure vitamin D/calcium supplementation if needed.   **Next Step**: Proceed to **surgical referral** for parathyroidectomy. | **Answer:** **Right inferior parathyroidectomy** **Intravenous 0.9% saline every 6 hours** (if hypercalcemia is severe/symptomatic) **Pamidronate 60 to 90 mg IV infusion** (if severe hypercalcemia requiring urgent lowering)  **Rationale:**   1. **Right inferior parathyroidectomy**:   Definitive treatment for confirmed primary hyperparathyroidism due to a localized parathyroid adenoma. The patient meets surgical criteria:  **24-hour urine calcium >10 mmol/24h** (11 mmol/24h).  Elevated PTH and imaging-confirmed adenoma.   2. **Intravenous 0.9% saline**:   Indicated for **acute/severe hypercalcemia** to promote volume expansion and calcium excretion. Use if serum calcium is significantly elevated or the patient is symptomatic (e.g., dehydration, renal insufficiency).   3. **Pamidronate 60-90 mg IV infusion**:   Temporarily lowers calcium by inhibiting bone resorption. Use in **severe hypercalcemia** (e.g., calcium >12 mg/dL) as a bridge to surgery.  **Why Other Options Are Not Appropriate:**   - **Watch and wait**: Contraindicated here. The patient meets surgical criteria (elevated urine calcium, localized adenoma). - **Desmopressin**: No role in hyperparathyroidism or hypercalcemia. - **Furosemide**: Only adjunctive in **acute** hypercalcemia with volume overload. Not a long-term solution. - **Prednisolone**: Effective for hypercalcemia due to granulomatous diseases (e.g., sarcoidosis) or vitamin D toxicity, not primary hyperparathyroidism. - **Calcitonin**: Short-term calcium reduction (e.g., acute crisis), but transient effect and not definitive. - **Hemofiltration**: Reserved for life-threatening hypercalcemia unresponsive to other therapies. - **3-and-a-half glands parathyroidectomy**: For **multigland hyperplasia**, not a single adenoma. - **Radiotherapy**: Not standard for parathyroid adenomas.   **Clinical Context:**   - **Surgery is curative** and should be prioritized unless contraindicated. - IV saline and pamidronate are supportive measures for severe hypercalcemia but do not replace definitive surgical management.   **Conclusion:** The most appropriate steps are **surgical removal of the adenoma** and **acute calcium-lowering therapies** if urgently needed. |
| Case 19 |  |  |  |
| diff | **Common and/or Dangerous Differential Diagnoses That Cannot Be Excluded:**   1. **Pituitary tumor (e.g., prolactinoma):**   **Why: The triad of galactorrhea, amenorrhea, and worsening headaches is classic for a prolactinoma. Imaging (MRI) is required to confirm.**   2. **Hypothalamic tumor:**   **Why: Hypothalamic dysfunction can cause hyperprolactinemia via disrupted dopamine regulation. Headaches and hormonal abnormalities necessitate exclusion of a hypothalamic mass.**   3. **Pregnancy:**   **Why: A common cause of secondary amenorrhea. Galactorrhea can occur postpartum. A β-hCG test is mandatory to rule out pregnancy, even with a 6-month history of amenorrhea (e.g., cryptic pregnancy or misreported dates).**   4. **Primary hypothyroidism:**   **Why: Elevated TSH in hypothyroidism stimulates TRH, which increases prolactin. Symptoms like fatigue, weight gain, and menstrual irregularities overlap. Requires TSH/free T4 testing.**   5. **Polycystic ovarian syndrome (PCOS):**   **Why: A common cause of oligomenorrhea/infertility. Though atypical due to the absence of hirsutism/acne, PCOS can present with irregular cycles and weight gain. Pelvic ultrasound and hormone testing are needed.**  **Excluded or Less Likely Options:**   - **Breast cancer: Unlikely with bilateral milky discharge and no breast masses/skin changes. Bloody or unilateral discharge is more concerning.** - **End-stage renal disease (ESRD): No renal symptoms (e.g., edema, nocturia) or lab abnormalities (prior normal labs).** - **Fibrocystic breast changes: Typically causes breast pain/lumps, not isolated milky discharge.** - **Physiologic nipple discharge: Possible with hormonal fluctuations, but pathologic causes (e.g., prolactinoma, hypothyroidism) must be ruled out first.**   **Key Next Steps:**   - **β-hCG: Rule out pregnancy.** - **Serum prolactin and TSH/free T4: Confirm hyperprolactinemia and exclude hypothyroidism.** - **MRI pituitary/hypothalamus: Evaluate for tumors.** - **Pelvic ultrasound and androgen levels: Assess for PCOS.**   **Conclusion: The critical diagnoses to exclude are pituitary/hypothalamic tumors, pregnancy, hypothyroidism, and PCOS. These are common and/or dangerous and require immediate testing.** | **Answer:** ✅ **Breast cancer** ✅ **Hypothalamic tumor** ✅ **Pituitary tumor** ✅ **Polycystic ovarian syndrome (PCOS)** ✅ **Pregnancy** ✅ **Primary hypothyroidism**  **Key Differential Diagnoses to Exclude:**   1. **Breast Cancer**:   Though bilateral milky discharge is less typical, breast cancer cannot be excluded without imaging (e.g., mammography/ultrasound) or biopsy.   2. **Hypothalamic Tumor**:   Amenorrhea, headaches, and hyperprolactinemia may result from hypothalamic dysfunction. MRI is required to rule out a tumor disrupting the HPA axis.   3. **Pituitary Tumor (Prolactinoma)**:   Classic triad of **galactorrhea**, **amenorrhea**, and **headaches** strongly suggests a prolactin-secreting pituitary adenoma. MRI pituitary is diagnostic.   4. **Polycystic Ovarian Syndrome (PCOS)**:   Amenorrhea and infertility are consistent with PCOS, though absence of hyperandrogenism (acne, hirsutism) makes it less likely. Requires LH/FSH ratio and pelvic ultrasound.   5. **Pregnancy**:   Must be excluded with a **urine or serum β-hCG test**, as pregnancy causes physiologic hyperprolactinemia and amenorrhea.   6. **Primary Hypothyroidism**:   Hypothyroidism elevates TRH, stimulating prolactin secretion. Symptoms (weight gain, fatigue, depression) overlap. Check **TSH and free T4**.  **Less Likely but Possible:**   - **Fibrocystic Breast Changes**: May explain breast tenderness but not the milky discharge or systemic symptoms. - **Physiologic Nipple Discharge**: Unlikely in a non-pregnant/non-lactating patient without hormonal triggers (e.g., medications).   **Excluded Diagnoses:**   - **End-Stage Renal Disease (ESRD)**: No clinical evidence (e.g., edema, hypertension, uremic symptoms) to suggest renal failure.   **Next Steps:**   1. **Pregnancy test** (immediate). 2. **Serum prolactin**, **TSH**, **free T4**, **LH/FSH**, and **testosterone**. 3. **MRI pituitary** to evaluate for adenoma or hypothalamic lesion. 4. **Breast imaging** (ultrasound/mammogram) to rule out malignancy.   **Note**: Prolactinoma remains the most likely diagnosis, but thorough evaluation is needed to exclude other critical causes. | **Answer:** **Breast cancer** **Hypothalamic tumor** **Pituitary tumor** **Pregnancy** **Primary hypothyroidism** **Polycystic ovarian syndrome (PCOS)**  **Rationale:**   1. **Pituitary tumor (prolactinoma)**:   Most likely diagnosis given galactorrhea, amenorrhea, headaches, and infertility. **Cannot be excluded without MRI**.   2. **Hypothalamic tumor**:   Hypothalamic dysfunction can cause hyperprolactinemia (via reduced dopamine). Headaches and hormonal abnormalities are concerning. **Requires MRI to rule out**.   3. **Breast cancer**:   Rarely presents with bilateral milky discharge, but **cannot be excluded without imaging (mammogram/ultrasound) or biopsy**.   4. **Pregnancy**:   Pregnancy causes physiologic hyperprolactinemia and amenorrhea. **Must exclude with β-hCG testing**, even with reported infertility.   5. **Primary hypothyroidism**:   Hypothyroidism elevates TRH, stimulating prolactin. Weight gain and lethargy are nonspecific but compatible. **Requires TSH/free T4 testing**.   6. **PCOS**:   Common cause of oligomenorrhea/infertility. Though lacking hyperandrogenism, **androgen levels and ovarian imaging are needed to exclude**.  **Why Other Options Are Less Likely (but still possible):**   - **End-stage renal disease (ESRD)**:   Can cause hyperprolactinemia, but no renal symptoms (e.g., edema, hypertension). **Requires creatinine/BUN testing** but less urgent.   - **Fibrocystic breast changes**:   Common but does not explain amenorrhea or systemic symptoms. Discharge here is hormonal, not cystic.   - **Physiologic nipple discharge**:   Typically related to pregnancy/breastfeeding. Unlikely in this context without confirmed pregnancy.  **Key Next Steps:**   - **β-hCG test** to rule out pregnancy. - **Prolactin, TSH, free T4, LH, FSH, estradiol** labs. - **Pituitary MRI** to assess for adenoma/hypothalamic lesion. - **Breast imaging** (e.g., ultrasound) if discharge persists despite normal prolactin.   **Conclusion:** These differentials represent **common causes of amenorrhea/infertility** (PCOS, hypothyroidism, pregnancy) or **dangerous conditions** (pituitary/hypothalamic tumors, breast cancer) that require immediate evaluation. |
| Diag1 | **Most Appropriate Initial Steps:**   1. **Serum β-hCG concentration:**   **Why: Essential to rule out pregnancy, a common and reversible cause of amenorrhea and galactorrhea. Serum testing is more sensitive than urine, especially for early or cryptic pregnancies.**   2. **Serum prolactin (PRL) level:**   **Why: Elevated prolactin is central to diagnosing hyperprolactinemia, which may indicate a pituitary tumor (e.g., prolactinoma) or hypothalamic dysfunction.**   3. **Serum thyroid-stimulating hormone (TSH):**   **Why: Hypothyroidism can elevate prolactin via TRH stimulation. TSH is the first-line test for thyroid dysfunction.**   4. **Serum free T4 level:**   **Why: Confirms hypothyroidism if TSH is elevated or evaluates for central causes if TSH is low/normal.**   5. **Pelvic ultrasound:**   **Why: Assesses for polycystic ovarian morphology (PCOS) or other structural causes of amenorrhea.**  **Excluded or Less Immediate Options:**   - **Urine pregnancy test: Redundant if serum β-hCG is performed.** - **CT scan of the brain without contrast: Poor sensitivity for pituitary/hypothalamic lesions compared to MRI.** - **Mammography: Not indicated for bilateral milky discharge without masses or concerning features (e.g., blood).** - **MRI of the brain with and without contrast: Critical after confirming hyperprolactinemia or if lab results suggest a pituitary/hypothalamic lesion. Not an *initial* step without biochemical evidence.** - **Ultrasound of the thyroid: Unnecessary unless thyroid nodules are suspected (e.g., palpable goiter).**   **Rationale:**   - **Initial labs (β-hCG, PRL, TSH, free T4) are prioritized to identify common causes (pregnancy, hypothyroidism, hyperprolactinemia) and guide subsequent imaging (e.g., MRI for prolactinoma).** - **Pelvic ultrasound evaluates for PCOS, a common cause of oligomenorrhea.** - **MRI is deferred until biochemical results confirm the need (e.g., elevated prolactin, unexplained amenorrhea).**   **Conclusion: Start with serum β-hCG, prolactin, TSH, free T4, and pelvic ultrasound. Proceed to MRI if hyperprolactinemia or other concerning findings emerge.** | **Answer:** ✅ **Urine pregnancy test** ✅ **Serum prolactin (PRL) level** ✅ **Serum thyroid-stimulating hormone (TSH)** ✅ **Serum free T4 level** ✅ **MRI of the brain with and without contrast** ✅ **Pelvic ultrasound**  **Rationale:**   1. **Urine pregnancy test**:   **Critical** to exclude pregnancy, which causes physiologic hyperprolactinemia and amenorrhea.   2. **Serum prolactin**:   Elevated prolactin confirms hyperprolactinemia, supporting diagnoses like **prolactinoma** or hypothyroidism.   3. **TSH and free T4**:   **Primary hypothyroidism** (elevated TSH, low free T4) stimulates TRH, increasing prolactin.   4. **MRI brain with contrast**:   Gold standard to evaluate for **pituitary adenoma** (e.g., prolactinoma) or hypothalamic lesions.   5. **Pelvic ultrasound**:   Assess for **PCOS** (e.g., ovarian cysts, stromal hyperplasia) as a cause of amenorrhea and infertility.  **Additional Considerations:**   - **Mammography**:   Bilateral milky discharge is less suggestive of malignancy, but breast imaging (preferably **ultrasound** in young patients) may be warranted. However, mammography is listed here and may be included if ultrasound is unavailable.   - **Serum β-hCG**:   Redundant if urine pregnancy test is positive/negative, but serum testing is more sensitive if clinical suspicion persists.  **Excluded Options:**   - **CT scan of the brain without contrast**: Inferior to MRI for pituitary visualization. - **Ultrasound of the thyroid**: Not indicated without thyroid enlargement or nodules. - **End-stage renal disease (ESRD)**: No clinical evidence (e.g., elevated creatinine, edema) to support testing.   **Next Steps:**   1. **Treat hyperprolactinemia** with dopamine agonists (e.g., cabergoline) if prolactinoma is confirmed. 2. **Address hypothyroidism** with levothyroxine if TSH is elevated. 3. **Fertility counseling** based on findings (e.g., ovulation induction for PCOS).   **Note**: While **breast cancer** cannot be entirely excluded without imaging, hormonal and pituitary evaluation takes priority given the clinical presentation. | **Answer:** **Serum β-hCG concentration** **Serum prolactin (PRL) level** **Serum thyroid-stimulating hormone (TSH)** **Serum free T4 level** **MRI of the brain with and without contrast**  **Rationale:**   1. **Serum β-hCG concentration**:   Essential to **exclude pregnancy**, a common cause of hyperprolactinemia and secondary amenorrhea. Serum testing is more sensitive than urine, especially in early pregnancy.   2. **Serum prolactin (PRL)**:   **Confirm hyperprolactinemia**, which is central to diagnosing prolactinoma. Elevated PRL supports a pituitary/hypothalamic etiology.   3. **TSH and free T4**:   **Rule out primary hypothyroidism**, as hypothyroidism increases TRH, leading to elevated prolactin.   4. **MRI of the brain with contrast**:   **Gold standard for visualizing pituitary/hypothalamic tumors** (e.g., prolactinoma). Required if prolactin is elevated or other causes are excluded.  **Why Other Options Are Less Immediate:**   - **Urine pregnancy test**: Less sensitive than serum β-hCG, especially early in pregnancy. - **CT scan of the brain**: Less sensitive than MRI for pituitary lesions. - **Mammography**: Bilateral milky discharge is more likely hormonal (e.g., hyperprolactinemia) than structural. Breast imaging can be deferred unless PRL is normal. - **Pelvic ultrasound**: PCOS is less likely without hyperandrogenism, though may be considered later if hormonal tests are inconclusive. - **Thyroid ultrasound**: Unnecessary if TSH/free T4 are normal.   **Conclusion:** These steps prioritize ruling out **pregnancy**, confirming **hyperprolactinemia**, excluding **hypothyroidism**, and evaluating for a **pituitary/hypothalamic tumor**. This approach efficiently narrows the differential diagnosis and guides targeted treatment. |
| Diag2 | **Most Appropriate Steps for Diagnosis and Treatment:**   1. **Humphrey's visual field test:**   **Why: Essential to detect subtle optic chiasm compression or visual field defects (e.g., bitemporal hemianopsia), even if MRI shows no obvious compression.**   2. **Serum insulin-like growth factor 1 (IGF-1) level:**   **Why: Assesses growth hormone (GH) axis. Pituitary macroadenomas can impair GH secretion (low IGF-1 suggests deficiency).**   3. **Serum FSH and LH levels:**   **Why: Evaluates gonadal axis function. Hypogonadotropic hypogonadism (low FSH/LH) is common in prolactinomas due to hyperprolactinemia suppressing GnRH.**   4. **Serum estradiol level:**   **Why: Confirms hypogonadism (low estradiol) secondary to hyperprolactinemia, which contributes to amenorrhea and bone loss risk.**   5. **Serum AM cortisol level:**   **Why: Screens for adrenal insufficiency (low AM cortisol suggests ACTH deficiency). Critical if the tumor affects the corticotroph axis.**   6. **Serum free T4 level:**   **Why: Confirms euthyroidism despite normal TSH. Pituitary tumors rarely disrupt the thyrotroph axis, but free T4 ensures central hypothyroidism is not missed.**  **Excluded or Less Immediate Options:**   - **Bilateral ocular tonometry: Measures intraocular pressure, irrelevant to pituitary tumors unless glaucoma is suspected (not indicated here).** - **Serum ACTH stimulation test: Reserved if AM cortisol is low to confirm adrenal insufficiency. Not a first-line test.** - **Serum free cortisol level: Unreliable for diagnosing adrenal insufficiency; 24-hour urine free cortisol is used for Cushing’s, not hypocortisolism.**   **Rationale:**   - **Visual field testing is critical to rule out optic nerve compromise.** - **Hormonal evaluation (IGF-1, FSH, LH, estradiol, AM cortisol, free T4) assesses for pituitary hormone deficiencies caused by the tumor or hyperprolactinemia.** - **Normal TSH + free T4 ensures thyroid axis is intact.**   **Next Steps:**   - **Start dopamine agonists (e.g., cabergoline) to lower prolactin and shrink the tumor.** - **Address hormone deficiencies (e.g., estrogen replacement for hypogonadism, hydrocortisone if adrenal insufficiency).** - **Repeat MRI and prolactin levels in 6-12 months to monitor treatment response.**   **Conclusion: Prioritize visual field testing and pituitary hormone evaluation to guide therapy and detect complications.** | **Answer:** ✅ **Humphrey's visual field test** ✅ **Serum insulin-like growth factor 1 (IGF-1) level** ✅ **Serum LH level** ✅ **Serum FSH level** ✅ **Serum estradiol level** ✅ **Serum AM cortisol level**  **Rationale:**   1. **Humphrey's visual field test**:   **Critical** to evaluate for **bitemporal hemianopsia** (compression of the optic chiasm), even if the MRI shows no current compression. Subtle deficits may exist, and baseline testing is essential for monitoring.   2. **Serum IGF-1**:   Screens for **acromegaly** (GH-secreting adenoma). Pituitary macroadenomas may co-secrete hormones, and IGF-1 reflects GH excess.   3. **Serum LH/FSH and estradiol**:   Assess for **hypogonadotropic hypogonadism** (low LH/FSH and estradiol) secondary to hyperprolactinemia or pituitary damage. Confirms estrogen deficiency driving amenorrhea and guides hormone replacement.   4. **Serum AM cortisol**:   Evaluates adrenal function. Macroadenomas may impair ACTH secretion, causing **secondary adrenal insufficiency**. A low AM cortisol (❤️ µg/dL) warrants further testing (e.g., ACTH stimulation test).  **Excluded Options:**   - **Bilateral ocular tonometry**: Measures intraocular pressure; not relevant unless glaucoma is suspected (no clinical evidence here). - **Serum free T4**: Already normal in prior testing (TSH 2.1 mIU/L), so unlikely central hypothyroidism. - **Serum ACTH stimulation test**: Reserved for confirming adrenal insufficiency if AM cortisol is low. - **Serum free cortisol level**: Less reliable than AM cortisol for screening adrenal insufficiency.   **Next Steps:**   1. **Treat prolactinoma** with **cabergoline** to normalize prolactin and shrink the tumor. 2. **Hormone replacement** (e.g., estrogen for hypogonadism, hydrocortisone if adrenal insufficiency confirmed). 3. **Repeat MRI** in 6–12 months to assess tumor response.   **Note**: If **AM cortisol is low**, proceed to ACTH stimulation test. If **IGF-1 is elevated**, confirm with oral glucose tolerance test for GH suppression. | **Answer:** **Humphrey's visual field test** **Serum insulin-like growth factor 1 (IGF-1) level** **Serum LH level** **Serum FSH level** **Serum estradiol level** **Serum AM cortisol level**  **Rationale:**   1. **Humphrey's visual field test**:   **Assesses for optic chiasm compression**, even if MRI shows no overt compression. Subtle visual field defects (e.g., bitemporal hemianopsia) may occur with sellar masses.   2. **Serum IGF-1**:   Screens for **growth hormone (GH) excess** (acromegaly), as pituitary macroadenomas can co-secrete GH.   3. **Serum LH/FSH and estradiol**:   Evaluates **hypogonadism secondary to hyperprolactinemia**. Elevated prolactin suppresses GnRH, leading to low LH/FSH and estradiol. Confirming hypogonadism guides hormone replacement if needed.   4. **Serum AM cortisol**:   Screens for **adrenal insufficiency** caused by pituitary dysfunction. Macroadenomas may compress the gland, impairing ACTH secretion.  **Why Other Options Are Less Immediate:**   - **Bilateral ocular tonometry**: Assesses intraocular pressure (glaucoma), unrelated to pituitary tumors unless secondary complications arise. - **Serum ACTH stimulation test**: Reserved for confirming adrenal insufficiency **after** an abnormal AM cortisol result. - **Serum free cortisol**: Less reliable than AM cortisol for initial screening. - **Serum free T4**: Already normal in prior testing; no need to repeat unless new symptoms emerge.   **Conclusion:** These steps evaluate **pituitary function** (gonadal, adrenal, and GH axes) and **mass effects** (visual fields). This comprehensive approach ensures detection of hormonal deficiencies/excesses and guides targeted therapy. |
| Dx | **The most likely diagnosis in this patient is hyperprolactinemia due to a pituitary macroadenoma (prolactinoma).**  **Key Reasoning:**   1. **Pituitary Macroadenoma:**   **MRI findings: Sellar mass measuring 1.1 cm × 1.6 cm × 2.1 cm (>1 cm = macroadenoma).**  **Prolactin level: 500 µg/L (severely elevated; levels >200 µg/L are diagnostic of a prolactinoma).**  **Normal TSH/free T4: Rules out primary hypothyroidism as the cause.**  **Negative pregnancy test: Excludes pregnancy.**   2. **Why Not Other Options:**   **Pituitary Microadenoma: Tumors <1 cm, but MRI confirms a macroadenoma.**  **Pituitary Stalk Compression by a Nonfunctioning Macroadenoma:**  **Typically causes mild prolactin elevation (<150 µg/L) due to reduced dopamine inhibition.**  **This patient’s extreme hyperprolactinemia (500 µg/L) points to direct prolactin secretion by the tumor, not stalk compression.**  **Primary Hypothyroidism: Normal TSH and free T4 exclude this.**  **Pregnancy: Urine test is negative.**  **Mechanism:**   - **Prolactinomas directly secrete prolactin, causing marked elevation.** - **The tumor’s size (>1 cm) and lack of other hormonal abnormalities (normal LH/FSH, IGF-1, cortisol, etc.) confirm a pure prolactin-secreting macroadenoma.**   **Conclusion:**  **The combination of severely elevated prolactin, macroadenoma on MRI, and exclusion of other causes confirms prolactinoma as the diagnosis. Treatment with dopamine agonists (e.g., cabergoline) is first-line to lower prolactin and shrink the tumor.** | **Answer**: ✅ **Pituitary macroadenoma**  **Rationale:**   1. **Elevated Prolactin (250 µg/L)**:   Prolactin levels >200 µg/L are **strongly indicative of a prolactin-secreting macroadenoma** rather than stalk compression (which typically causes mild elevations <150 µg/L).   2. **MRI Findings**:   Confirmed **pituitary macroadenoma** (≥10 mm). Prolactinomas are the most common hormone-secreting pituitary tumors and frequently present as macroadenomas.   3. **Exclusion of Other Causes**:   **Primary hypothyroidism**: Ruled out by normal free T4.  **Pregnancy**: Not indicated in the clinical scenario.  **Stalk compression by a nonfunctioning macroadenoma**: Unlikely given the high prolactin level (stalk effect rarely causes prolactin >150 µg/L).  **Key Differentiators:**   - **Prolactinoma (macroadenoma)**:   Directly secretes prolactin, leading to marked elevation (often >200 µg/L).  Responds well to dopamine agonists (e.g., cabergoline).   - **Stalk Compression**:   Results in **disruption of dopamine inhibition**, causing mild-moderate prolactin elevation.  Requires treatment of the compressive mass (e.g., surgery if symptomatic).  **Conclusion:**  The **prolactin level of 250 µg/L** in the setting of a **pituitary macroadenoma** confirms the diagnosis of a **prolactin-secreting macroadenoma**. This distinguishes it from stalk compression or other causes of hyperprolactinemia.  **Next Step**: Initiate **dopamine agonist therapy** (e.g., cabergoline) to lower prolactin and shrink the tumor. | **Answer:** **Pituitary macroadenoma**  **Rationale:**   1. **Key Findings Supporting Diagnosis**:   **Elevated prolactin (180 ng/mL)**: Prolactin levels >150–200 ng/mL are **highly specific for prolactin-secreting macroadenomas** (prolactinomas).  **Pituitary macroadenoma on MRI**: Confirms a tumor >10 mm in size.  **Normal ancillary hormone tests (LH, FSH, estradiol, IGF-1, AM cortisol, free T4)**: Excludes other causes of hyperprolactinemia (e.g., hypothyroidism, stalk compression).   2. **Why Other Options Are Less Likely**:   **Pituitary microadenoma**: Unlikely given MRI confirmation of a macroadenoma (>10 mm).  **Pituitary stalk compression by a nonfunctioning macroadenoma**:  Typically causes **mild hyperprolactinemia** (prolactin <150 ng/mL).  Often associated with **hypopituitarism** (e.g., low ACTH, TSH), which is absent here.  **Primary hypothyroidism**: Excluded by **normal free T4**. Hypothyroidism elevates TSH and TRH (stimulating prolactin), but both are normal here.  **Pregnancy**: Presumptively excluded (e.g., negative β-hCG testing in prior workup).  **Pathophysiology of Prolactinoma**:   - Prolactinomas directly secrete excess prolactin, suppressing GnRH and causing hypogonadism. However, early-stage tumors may preserve normal LH/FSH/estradiol (as seen here), especially if prolactin elevation is recent or intermittent.   **Next Steps**:   - **Dopamine agonist therapy (e.g., cabergoline)**: First-line treatment to lower prolactin and shrink the tumor. - **Monitor for delayed hypogonadism**: Even with normal initial LH/FSH, prolonged hyperprolactinemia may eventually suppress the HPG axis.   **Conclusion**: The combination of **markedly elevated prolactin**, **macroadenoma on MRI**, and **normal ancillary hormones** confirms a **prolactin-secreting pituitary macroadenoma** as the cause of hyperprolactinemia. |
| Mang | **The appropriate treatments for this patient with a confirmed prolactinoma (pituitary macroadenoma) are:**  **Correct Options:**   1. **Cabergoline 1 mg orally 2 times per week:**   **First-line therapy for prolactinomas due to high efficacy, tolerability, and long half-life.**  **Typical starting dose is 0.25–0.5 mg twice weekly, but titration to 1 mg twice weekly is common for severe hyperprolactinemia.**   2. **Bromocriptine 2.5 mg orally twice a day:**   **An alternative dopamine agonist, though less preferred than cabergoline due to more side effects (e.g., nausea, dizziness) and shorter duration of action.**   3. **Quinagolide 25 mcg orally once daily:**   **A non-ergot dopamine agonist used in some regions as an alternative for patients intolerant to cabergoline or bromocriptine.**  **Why Other Options Are Incorrect:**   - **Admit for surgery: Reserved for tumors resistant to dopamine agonists, apoplexy, or vision loss unresponsive to medication. Not first-line.** - **Pituitary radiotherapy: Used only in refractory cases or residual tumors after failed surgery/medication.** - **Repeat MRI/prolactin in 3 months: Treatment should start immediately given the confirmed macroadenoma and severe hyperprolactinemia (500 µg/L).** - **Lanreotide/Pegvisomant: Used for acromegaly (growth hormone-secreting tumors), not prolactinomas.**   **Key Takeaway:**  **Dopamine agonists (cabergoline, bromocriptine, quinagolide) are the cornerstone of treatment. Cabergoline is preferred for its efficacy and tolerability. Immediate intervention is required to lower prolactin, shrink the tumor, and prevent complications (e.g., hypogonadism, tumor expansion).** | **Appropriate Treatments**: ✅ **Cabergoline 1 mg orally 2 times per week** ✅ **Bromocriptine 2.5 mg orally twice a day**  **Rationale:**   1. **Dopamine agonists** are **first-line therapy** for prolactin-secreting macroadenomas:   **Cabergoline** is preferred due to its longer half-life, better tolerability, and higher efficacy compared to bromocriptine.  **Bromocriptine** is an alternative if cabergoline is unavailable or contraindicated.  Both reduce prolactin levels and shrink tumors in >80% of cases.   2. **Incorrect Options**:   **Admit for immediate surgery**: Reserved for tumors causing **acute vision loss**, **apoplexy**, or **drug resistance/intolerance**. Not first-line.  **Pituitary radiotherapy**: Used only for **aggressive tumors refractory to surgery/medications**.  **Repeat MRI/prolactin in 3 months**: Treatment should start **immediately** unless the tumor is tiny/asymptomatic (unlikely in a macroadenoma).  **Lanreotide/pegvisomant**: Used for **acromegaly** (growth hormone-secreting tumors), not prolactinomas.  **Quinagolide**: A dopamine agonist used in some countries, but **not FDA-approved** in the U.S.  **Key Points:**   - **Macroadenomas require urgent treatment** to prevent complications (e.g., vision loss, hypopituitarism). - **Cabergoline** is started at 0.25 mg twice weekly and titrated based on prolactin levels. - Monitor for **tumor shrinkage** (repeat MRI in 6–12 months) and **prolactin normalization**.   **Avoid Delay**: Early dopamine agonist therapy improves outcomes and reduces tumor size. | **Appropriate Treatments:**   - **Bromocriptine 2.5 mg orally twice a day** - **Cabergoline 1 mg orally 2 times per week**   **Rationale:**   1. **Dopamine agonists (bromocriptine and cabergoline)**:   **First-line therapy** for prolactin-secreting macroadenomas.  **Cabergoline** is preferred due to:  Higher efficacy and tolerability.  Longer half-life (dosing 1–2 times weekly).  Better side-effect profile (e.g., less nausea/dizziness compared to bromocriptine).  **Bromocriptine** is effective but requires more frequent dosing (twice daily) and has more side effects.   2. **Why Other Options Are Inappropriate**:   **Surgical resection**: Not urgent unless the tumor is resistant to medication, causes severe complications (e.g., vision loss), or the patient cannot tolerate dopamine agonists.  **Radiotherapy**: Reserved for aggressive tumors refractory to both medication and surgery.  **Repeat MRI/prolactin in 3 months**: Treatment should **begin immediately**; delaying risks tumor growth and complications (e.g., hypogonadism).  **Lanreotide/pegvisomant**: Used for **acromegaly** (somatostatin analog and GH receptor antagonist, respectively).  **Quinagolide**: A dopamine agonist used in some countries, but less commonly than cabergoline/bromocriptine.  **Important Notes**:   - **Cabergoline dosing**: While 0.25–0.5 mg twice weekly is typical for initiation, higher doses (e.g., 1 mg twice weekly) may be used for larger tumors or refractory cases. - **Monitoring**:   Check prolactin levels at 4–6 weeks to assess response.  Repeat MRI in 6–12 months to evaluate tumor shrinkage.  **Conclusion**: Dopamine agonists are the cornerstone of treatment. **Cabergoline is first-line**, but bromocriptine is an acceptable alternative if cabergoline is unavailable or contraindicated. |
| Case 20 |  |  |  |
| diff | **The appropriate differential diagnoses for this patient include:**  **Correct Options:**   1. **Benign prostatic enlargement (BPH):**   **Most likely diagnosis. Symptoms (nocturia, straining, weak stream) and exam findings (smooth, enlarged prostate) are classic for BPH.**   2. **Prostate cancer:**   **Family history (father with prostate cancer) necessitates inclusion despite a benign-feeling prostate on exam.**   3. **Urethral stricture:**   **Can mimic BPH with obstructive symptoms (straining, weak stream). No history of trauma/infection, but age and symptoms warrant consideration.**   4. **Bladder stone:**   **May cause intermittent obstruction, leading to nocturia and straining. Absence of hematuria/dysuria makes it less likely but still possible.**   5. **Urinary tract infection (UTI):**   **Elderly patients may present atypically. Urinalysis is needed to exclude, even without classic symptoms (e.g., dysuria, fever).**  **Incorrect Options:**   - **Chronic bacterial prostatitis:**   **Typically involves pelvic pain, dysuria, or recurrent UTIs, which are absent here.**   - **Detrusor-sphincter dyssynergia:**   **Associated with neurological conditions (e.g., spinal cord injury). No neurologic deficits reported.**   - **Diabetes mellitus:**   **No daytime polyuria, polydipsia, or weight changes. Unlikely given normal appetite/fluid intake.**   - **Drug side effects:**   **Avanafil (PDE5 inhibitor) and aspirin do not typically cause urinary obstruction.**  **Key Takeaway:**  **BPH is the leading diagnosis, but prostate cancer must be ruled out due to family history. Urethral stricture, bladder stone, and UTI are plausible structural/infectious considerations. Further testing (PSA, urinalysis, post-void residual, uroflowmetry) will clarify the etiology.** | **Differential Diagnoses** (Selected Options): ✅ **Benign prostatic enlargement (BPH)** ✅ **Prostate cancer** ✅ **Urethral stricture** ✅ **Bladder stone** ✅ **Urinary tract infection**  **Rationale:**   1. **Benign Prostatic Hyperplasia (BPH)**:   **Most likely diagnosis** given age, obstructive symptoms (nocturia, straining, weak stream), and **enlarged, smooth prostate** on DRE.   2. **Prostate Cancer**:   **Family history** (father died of prostate cancer) and age warrant consideration.  DRE findings (no nodules) reduce suspicion, but prostate cancer can occur without palpable abnormalities. **PSA testing is critical**.   3. **Urethral Stricture**:   Can mimic BPH with obstructive symptoms (straining, weak stream).  Less likely without history of trauma/infection, but still a possible anatomic obstruction.   4. **Bladder Stone**:   May cause intermittent obstruction, incomplete emptying, or nocturia.  Absence of hematuria/pain makes it less likely, but imaging (e.g., ultrasound) can exclude.   5. **Urinary Tract Infection (UTI)**:   Older adults may lack classic symptoms (dysuria, fever).  Urinalysis is needed to rule out, despite patient’s denial of dysuria.  **Less Likely but Possible:**   - **Chronic Bacterial Prostatitis**:   Typically includes pelvic pain, dysuria, or recurrent UTIs (not present here).   - **Diabetes Mellitus**:   Nocturnal polyuria can occur, but patient denies daytime polyuria or polydipsia.  **Unlikely:**   - **Detrusor-Sphincter Dyssynergia**:   Requires neurologic dysfunction (e.g., spinal cord injury), which is absent.   - **Drug Side Effects**:   Avanafil/aspirin do not directly cause urinary obstruction.  **Next Steps:**   1. **PSA testing** to evaluate for prostate cancer. 2. **Urinalysis** and **urine culture** to exclude UTI. 3. **Post-void residual (PVR) measurement** and **bladder ultrasound** to assess for stones/retention. 4. **Uroflowmetry** or **cystoscopy** if urethral stricture is suspected.   **Key**: Prioritize BPH and prostate cancer evaluation while ruling out other obstructive causes. | **Differential Diagnoses for This Patient**:   - **Benign prostatic enlargement (BPH)** - **Prostate cancer** - **Urethral stricture** - **Bladder stone**   **Rationale**:   1. **Benign Prostatic Enlargement (BPH)**:   **Most likely diagnosis**. Classic symptoms include nocturia, weak stream, straining, and an enlarged prostate (70 g on DRE).   2. **Prostate Cancer**:   **Mandatory consideration** due to family history (father with prostate cancer) and age. Even with a smooth DRE, prostate cancer can coexist with BPH. Requires **PSA testing** and potential biopsy.   3. **Urethral Stricture**:   Causes obstructive symptoms (straining, weak stream). History of prior trauma/infection could support this, but absence does not rule it out.   4. **Bladder Stone**:   May cause intermittent obstruction and nocturia. Absence of hematuria or pain lowers likelihood, but imaging (e.g., ultrasound) is needed to exclude.  **Excluded Diagnoses**:   - **Chronic Bacterial Prostatitis**: No dysuria, pelvic pain, or fever. Prostate exam non-tender. - **Detrusor-Sphincter Dyssynergia**: Requires neurologic dysfunction (e.g., spinal cord injury), absent here. - **Diabetes Mellitus**: No polyuria, polydipsia, or daytime frequency. Normal fluid intake. - **Drug Side Effects**: Avanafil/aspirin do not cause obstructive LUTS. - **Urinary Tract Infection (UTI)**: No dysuria, fever, or suprapubic tenderness.   **Next Steps**:   - **PSA testing** to evaluate for prostate cancer. - **Urinalysis** to rule out UTI or hematuria. - **Post-void residual volume** and **uroflowmetry** to assess obstruction. - **Imaging (e.g., ultrasound)** if bladder stone or stricture is suspected.   **Conclusion**: BPH is the leading diagnosis, but **prostate cancer must be ruled out** given family history. Urethral stricture and bladder stone remain possible secondary causes of obstruction. |
| diag | **The most appropriate next steps for this patient are:**  **Correct Options:**   1. **Urinalysis and urine culture:**   **Essential to rule out infection (UTI/prostatitis) and hematuria, even in the absence of classic symptoms.**   2. **Serum PSA level:**   **Mandatory due to family history of prostate cancer and enlarged prostate. PSA screening is critical for risk stratification.**   3. **Post-void residual measurement:**   **Assesses bladder emptying efficiency. Elevated residual volume (>100–200 mL) supports obstructive etiology (e.g., BPH) and guides therapy.**   4. **Serum creatinine concentration:**   **Evaluates renal function. Chronic urinary retention from BPH can lead to obstructive nephropathy.**  **Incorrect Option:**   - **Prostate ultrasound:**   **Not routinely required upfront. Transrectal ultrasound (TRUS) is typically reserved for elevated PSA, abnormal DRE, or suspected prostate cancer. A basic DRE already suggests benign enlargement (70 g).**  **Key Justifications:**   - **Urinalysis and PSA are first-line tests for all patients with LUTS and prostate enlargement.** - **Post-void residual and creatinine assess functional impact of obstruction (e.g., incomplete emptying, renal impairment).** - **Prostate ultrasound may be considered later if PSA is elevated, biopsy is needed, or surgical planning (e.g., TURP) requires precise prostate sizing.**   **Next: Start alpha-blockers (e.g., tamsulosin) for symptom relief while awaiting test results. If PSA is elevated, proceed to urology referral for biopsy.** | **Most Appropriate Next Steps**: ✅ **Post-void residual measurement** ✅ **Serum PSA level** ✅ **Urinalysis and urine culture** ✅ **Serum creatinine concentration**  **Rationale:**   1. **Urinalysis and urine culture**:   **Critical** to exclude **urinary tract infection (UTI)** or **hematuria** (e.g., from bladder stones/prostate cancer). Older adults may lack classic UTI symptoms.   2. **Serum PSA level**:   **Mandatory** given his **family history of prostate cancer** and age. PSA helps differentiate BPH from malignancy, even with a smooth prostate on DRE.   3. **Post-void residual (PVR) measurement**:   Assesses **bladder emptying efficiency**. Elevated PVR (>100 mL) supports bladder outlet obstruction (e.g., BPH, urethral stricture) and guides management.   4. **Serum creatinine concentration**:   Evaluates for **renal impairment** secondary to chronic urinary retention (e.g., hydronephrosis from obstruction).  **Less Immediate/Contingent Testing:**   - **Prostate ultrasound**:   Not urgent if DRE already suggests BPH and PSA is normal. May be used later to confirm prostate size or if PSA is elevated.   - **Bladder ultrasound** (to rule out stones) or **uroflowmetry** (to quantify obstruction) could follow if initial tests are inconclusive.   **Excluded Options:**   - **Prostate ultrasound** is not a first-line test here unless PSA or urinalysis raises concern for cancer/stones.   **Summary**: Prioritize urinalysis, PSA, creatinine, and PVR to confirm BPH, rule out infection/cancer, and assess renal/urinary tract function. | **Most Appropriate Next Steps**:   - **Urinalysis and urine culture** - **Serum PSA level** - **Post-void residual measurement** - **Serum creatinine concentration**   **Rationale**:   1. **Urinalysis and urine culture**:   **Essential** to rule out infection (UTI) or hematuria, even though symptoms lack dysuria/fever. Older adults may present atypically.   2. **Serum PSA level**:   **Critical** given family history of prostate cancer, age, and enlarged prostate. PSA helps assess cancer risk and guides further evaluation (e.g., biopsy).   3. **Post-void residual measurement**:   Evaluates bladder emptying efficiency. A high residual volume supports obstruction (e.g., BPH, stricture) and informs management.   4. **Serum creatinine concentration**:   Assesses renal function. Chronic urinary retention from obstruction can lead to hydronephrosis or kidney damage.  **Less Immediate/Unnecessary at This Stage**:   - **Prostate ultrasound**:   While useful for prostate sizing or structural evaluation, the DRE already estimates prostate size (70 g). Ultrasound may be deferred unless PSA or other findings suggest cancer or stones.  **Excluded Options**:   - **Bladder stone imaging (e.g., ultrasound)**: Not prioritized unless urinalysis shows hematuria or symptoms suggest stones. - **Advanced imaging (e.g., MRI)**: Reserved for elevated PSA or suspicious findings.   **Conclusion**: The priority is to **rule out infection (urinalysis)**, **assess prostate cancer risk (PSA)**, **evaluate obstruction (post-void residual)**, and **check renal function (creatinine)**. These steps align with guidelines for evaluating lower urinary tract symptoms in older men. |
| dx | **The most likely diagnosis is:**  **• Benign prostatic hyperplasia (BPH)**  **Key Supporting Evidence:**   1. **Normal PSA (3.2 ng/mL):**   **PSA is within the normal range (0–4 ng/mL), making prostate cancer unlikely at this stage.**  **Family history alone does not override the normal PSA result.**   2. **Negative urinalysis/urine culture:**   **Excludes infection (e.g., chronic bacterial prostatitis) and hematuria (e.g., bladder stone).**   3. **Enlarged prostate (70 g) on DRE:**   **Consistent with BPH, as DRE detected benign, symmetric enlargement without nodules or asymmetry.**   4. **Clinical presentation:**   **Obstructive urinary symptoms (e.g., hesitancy, weak stream, incomplete emptying) align with BPH-related bladder outlet obstruction.**  **Why Other Options Are Less Likely:**   - **Prostate cancer:**   **Normal PSA and benign-feeling prostate on DRE make cancer unlikely. No nodules or asymmetry were reported.**   - **Chronic bacterial prostatitis:**   **Requires recurrent UTIs, pelvic pain, or positive urine/prostatic fluid cultures. Negative urinalysis/culture rules this out.**   - **Bladder stone:**   **Typically causes intermittent pain, hematuria, or abrupt urinary stoppage. No hematuria or infection was found here.**  **Next Step: Confirm BPH-related obstruction with uroflowmetry/post-void residual measurement and initiate BPH therapy (e.g., alpha-blockers + 5-alpha reductase inhibitors for 70 g prostate).** | **Most Likely Diagnosis**: ✅ **Benign Prostatic Hyperplasia (BPH)**  **Rationale:**   1. **Clinical Presentation**:   Classic BPH symptoms (nocturia, weak stream, hesitancy) in a 65-year-old male.   2. **Supportive Test Results**:   **Normal PSA (3.2 ng/mL)**: Makes prostate cancer **unlikely** (though not excluded entirely).  **Negative urinalysis/urine culture**: Rules out UTI, bladder stone, or prostatitis.  Smooth prostate on DRE further reduces suspicion for malignancy.   3. **Excluded Alternatives**:   **Prostate cancer**: Normal PSA and smooth DRE lower suspicion.  **Chronic bacterial prostatitis**: Requires recurrent UTIs/pelvic pain (absent here).  **Bladder stone**: Urinalysis would typically show hematuria or crystals.  **Next Steps**: Confirm BPH with **post-void residual measurement** and assess renal function (**serum creatinine**). If obstruction is confirmed, initiate BPH treatment (e.g., alpha-blockers). | **Most Likely Diagnosis**: **• Benign Prostatic Hyperplasia (BPH)**  **Supporting Evidence**:   1. **Clinical Presentation**:   Symptoms (nocturia, weak stream, straining) align with **bladder outlet obstruction** from BPH.  **Enlarged prostate (70 g)** on DRE supports BPH.   2. **Test Results**:   **Normal PSA (3.2 ng/mL)**: Makes prostate cancer less likely (though not fully excluded long-term).  **Negative urinalysis/culture**: Rules out infection (chronic prostatitis) and bladder stone (no hematuria).   3. **Exclusion of Alternatives**:   **Bladder stone**: Unlikely without hematuria or pain.  **Chronic bacterial prostatitis**: No leukocytes/bacteria in urine or dysuria.  **Prostate cancer**: Normal PSA and smooth DRE reduce suspicion (though family history warrants ongoing monitoring).  **Next Steps**:   - Confirm obstruction with **post-void residual measurement** and **uroflowmetry**. - Initiate BPH treatment (e.g., alpha-blockers) if obstruction is confirmed. - Continue annual PSA monitoring due to family history.   **Conclusion**: BPH is the **most likely diagnosis** based on symptoms, exam, and test results. |
| mang | **The most appropriate management options for confirmed BPH in this patient are:**  **• Doxazosin 1 mg po daily**  **• Finasteride 5 mg po daily**  **• Tadalafil 2.5 mg po daily**  **Rationale:**   1. **Doxazosin (alpha-blocker):**   **Provides rapid relief of obstructive symptoms by relaxing prostate and bladder neck smooth muscle.**  **First-line for moderate-to-severe BPH symptoms.**   2. **Finasteride (5-alpha reductase inhibitor):**   **Reduces prostate volume over time (critical for a 70 g prostate).**  **Combined with alpha-blockers (e.g., doxazosin), this is the standard dual therapy for large prostates.**   3. **Tadalafil (PDE5 inhibitor):**   **Approved for BPH to improve urinary symptoms and flow.**  **Particularly useful if the patient has concomitant erectile dysfunction.**  **Why Other Options Are Less Appropriate:**   - **Oxybutynin (anticholinergic):**   **Avoid in pure BPH with obstructive symptoms, as it increases the risk of urinary retention. Only use if overactive bladder symptoms (e.g., urgency, frequency) coexist.**   - **Prostate biopsy:**   **Not indicated with normal PSA and a benign-feeling prostate on DRE.**   - **Transurethral resection of the prostate (TURP):**   **Reserved for patients with refractory symptoms, recurrent urinary retention, or complications (e.g., hydronephrosis). First-line therapy is medical management.**  **Key Consideration:**   - **Combination therapy (alpha-blocker + 5-alpha reductase inhibitor) is recommended for patients with large prostates (>30–40 g) and moderate-to-severe symptoms.** - **Tadalafil can be added for synergistic symptom relief or if erectile dysfunction is present.**   **Immediate Plan: Start doxazosin + finasteride and consider adding tadalafil if symptoms persist or ED is present. Reassess symptoms and post-void residual volume in 3–6 months.** | **Most Appropriate Management Options**: ✅ **Doxazosin 1 mg PO daily** ✅ **Finasteride 5 mg PO daily** ✅ **Tadalafil 2.5 mg PO daily**  **Rationale:**   1. **Doxazosin (Alpha-Blocker)**:   First-line for **symptomatic BPH**; relaxes prostate/bladder neck smooth muscle to improve urinary flow.  Rapid symptom relief (within days).   2. **Finasteride (5-Alpha Reductase Inhibitor)**:   Reduces prostate size over 6-12 months (ideal if prostate is **enlarged**).  Often combined with alpha-blockers for **moderate-severe BPH** (synergistic effect).   3. **Tadalafil (PDE5 Inhibitor)**:   FDA-approved for BPH (improves LUTS and erectile dysfunction if present).  Alternative for patients preferring **once-daily dosing** or with comorbid ED.  **Excluded Options:**  ❌ **Oxybutynin (Anticholinergic)**:   - Used for **overactive bladder** (urgency, incontinence), not BPH. Nocturia here is likely due to obstruction, not detrusor overactivity.   ❌ **Prostate Biopsy**:   - Not indicated with **normal PSA (3.2 ng/mL)** and smooth prostate on DRE.   ❌ **Transurethral Resection of the Prostate (TURP)**:   - Reserved for **refractory BPH** or complications (e.g., recurrent retention, hydronephrosis). Not first-line unless medical therapy fails.   **Next Steps:**   - Start **monotherapy** (e.g., doxazosin) or **combination therapy** (doxazosin + finasteride) based on prostate size and symptom severity. - Consider tadalafil if ED coexists or patient prioritizes once-daily dosing. - Reassess symptoms and **post-void residual** in 3-6 months.   **Key Takeaway**: Medical therapy with **alpha-blockers**, **5-alpha reductase inhibitors**, or **tadalafil** is first-line for uncomplicated BPH. Avoid anticholinergics (e.g., oxybutynin) unless overactive bladder is confirmed. | **Most Appropriate Management Options**:   1. **Doxazosin 1 mg PO daily**   **Rationale**: Alpha-blockers (e.g., doxazosin) relax prostate and bladder neck smooth muscle, improving urinary flow. Effective for rapid symptom relief in BPH.   2. **Finasteride 5 mg PO daily**   **Rationale**: 5-alpha reductase inhibitors (e.g., finasteride) reduce prostate size over time by blocking DHT. Indicated for enlarged prostates (>30–40 g), as in this case (70 g).   3. **Tadalafil 2.5 mg PO daily**   **Rationale**: Low-dose tadalafil (PDE5 inhibitor) is FDA-approved for BPH. Improves LUTS by relaxing bladder/prostate smooth muscle and enhancing blood flow.  **Excluded Options**:   - **Oxybutynin 5 mg PO BID**: Anticholinergics (e.g., oxybutynin) treat overactive bladder (urgency, frequency) but **worsen urinary retention** in BPH. Avoid unless mixed OAB symptoms are present. - **Prostate Biopsy**: Not indicated with normal PSA (3.2 ng/mL) and smooth DRE. Reserved for elevated PSA, abnormal imaging, or suspicious findings. - **Transurethral Resection of the Prostate (TURP)**: Surgery is for severe, refractory symptoms or complications (e.g., retention, hydronephrosis). Not first-line in stable BPH.   **Guideline-Based Approach**:   - **Combination Therapy**: Doxazosin (rapid symptom relief) + finasteride (long-term prostate reduction) is recommended for large prostates (AUA/EAU guidelines). - **Tadalafil**: A valid alternative for patients preferring once-daily dosing or with concurrent erectile dysfunction.   **Next Step**:   - Start **doxazosin + finasteride** for optimal symptom control and prostate volume reduction. - Consider **tadalafil** if the patient has ED or prefers avoiding alpha-blockers (e.g., hypotension risk). - Monitor symptoms and PSA annually. |
| Case 21 |  |  |  |
| diff | **The following differential diagnoses cannot be excluded based on the current history and physical examination:**  **• Subarachnoid hemorrhage (SAH)**   - **Rationale: Despite the recurrent nature of her headaches, the sudden-onset "worst headache of her life" raises concern for SAH. Sentinel headaches (warning leaks) can precede a major bleed. A non-contrast CT head is required to exclude this diagnosis.**   **• Brain tumor**   - **Rationale: While she lacks focal neurological deficits or papilledema, brain tumors can present with intermittent, worsening headaches (especially with exertion). Imaging (e.g., MRI brain) is needed to rule out a mass lesion.**   **• Pheochromocytoma**   - **Rationale: Episodic headaches, palpitations, hypertension, and neck swelling triggered by exertion/strain strongly suggest pheochromocytoma. Plasma-free metanephrines or 24-hour urine catecholamines are required for confirmation.**   **• Migraine**   - **Rationale: Recurrent severe headaches with nausea and lightheadedness could represent migraines, even without typical features (e.g., aura, photophobia). Migraine variants (e.g., "basilar-type") may present with occipital pain and autonomic symptoms.**   **• Anxiety disorder**   - **Rationale: Anxiety can manifest with palpitations, lightheadedness, and headaches. However, her symptoms (e.g., neck swelling, exertion-triggered episodes) suggest an organic cause. Anxiety alone cannot fully explain the clinical picture but remains a possible comorbid or contributing factor.**   **• Meningitis**   - **Rationale: Viral meningitis may present with headache, nausea, and neck discomfort without fever or meningeal signs. A lumbar puncture is necessary to exclude infectious/inflammatory causes.**   **• Idiopathic intracranial hypertension (IIH)**   - **Rationale: IIH typically presents with headaches, nausea, and papilledema, but papilledema is absent in ~5% of cases. While less likely, fundoscopic re-evaluation and lumbar puncture (to assess opening pressure) are needed for exclusion.**   **Differentials That Can Likely Be Excluded:**   - **Cluster headache: Unlikely due to occipital location, lack of unilateral autonomic symptoms (e.g., lacrimation, nasal congestion), and duration (cluster headaches last 15–180 minutes).** - **Giant cell arteritis (GCA): Extremely rare in a 31-year-old; typical features (e.g., jaw claudication, temporal artery tenderness) are absent.** - **Tension headache: Atypical due to sudden severe pain, autonomic symptoms (palpitations, neck swelling), and exacerbation with exertion.**   **Immediate Next Steps:**   1. **CT head (non-contrast) to rule out SAH or mass lesion.** 2. **MRI/MRA brain if CT is inconclusive.** 3. **Lumbar puncture (if SAH or meningitis is suspected).** 4. **Plasma-free metanephrines and 24-hour urine catecholamines for pheochromocytoma.** 5. **Fundoscopic exam to reassess for papilledema (IIH).**   **Note: Until these investigations are completed, the above diagnoses remain in the differential.** | **Differential Diagnoses That Cannot Be Excluded**: ✅ **Subarachnoid hemorrhage (SAH)** ✅ **Pheochromocytoma** ✅ **Brain tumor** ✅ **Migraine** ✅ **Cluster headaches** ✅ **Anxiety disorder**  **Rationale:**   1. **Subarachnoid Hemorrhage (SAH)**:   "Worst headache of life" with sudden onset is classic for SAH. Recurrent episodes could represent sentinel bleeds.  **Cannot exclude** without neuroimaging (CT head) and lumbar puncture.   2. **Pheochromocytoma**:   Episodic headaches with **palpitations, hypertension (BP 150/90), anxiety, and neck swelling** align with catecholamine surges.  **Cannot exclude** without 24-hour urine metanephrines or plasma-free metanephrines.   3. **Brain Tumor**:   Progressive headache frequency raises concern, though lack of focal deficits or papilledema is reassuring.  **Cannot exclude** without neuroimaging (MRI/CT).   4. **Migraine**:   Recurrent severe headaches with nausea and triggers (exertion) are consistent with migraine. Absence of photophobia/phonophobia is atypical but not exclusionary.  **Cannot exclude** without further evaluation of migraine-specific features.   5. **Cluster Headaches**:   Autonomic symptoms (palpitations, neck swelling) and episodic pattern may overlap. However, occipital location and lack of unilateral periorbital pain make this less likely.  **Cannot fully exclude** due to variable presentations.   6. **Anxiety Disorder**:   Anxiety and palpitations could suggest a primary psychiatric etiology, but organic causes (e.g., pheo) must first be ruled out.  **Cannot exclude** until secondary causes are investigated.  **Excluded Diagnoses:**  ❌ **Giant Cell Arteritis (GCA)**: Age <50 and absence of jaw claudication/scalp tenderness make GCA improbable. ❌ **Idiopathic Intracranial Hypertension (IIH)**: No papilledema or vision changes on exam. ❌ **Meningitis**: No fever, neck stiffness, or meningeal signs (negative Kernig/Brudzinski). ❌ **Tension Headache**: Atypical due to severe, sudden pain with autonomic features.  **Key Next Steps:**   1. **CT head** to rule out SAH or mass lesion. 2. **Lumbar puncture** if CT is negative for SAH. 3. **24-hour urine metanephrines** to assess for pheochromocytoma. 4. **MRI brain** if concern for tumor or atypical migraine. 5. **Psychiatric evaluation** if organic causes are ruled out.   **Clinical Pearl**: In thunderclap headaches, **SAH and pheochromocytoma are critical "can't miss" diagnoses** despite overlapping features with migraine or anxiety. Always prioritize ruling out life-threatening causes first. | **Differential Diagnoses That Cannot Be Excluded Based on History and Physical Examination:**   1. **Subarachnoid hemorrhage (SAH)**:   Thunderclap headache ("worst of life") is classic for SAH. Recurrent episodes could represent sentinel headaches. **Cannot exclude without non-contrast CT head**.   2. **Brain tumor**:   Headaches triggered by straining (suggestive of increased intracranial pressure). No papilledema or focal deficits, but tumors may present without these initially. **Requires neuroimaging (CT/MRI)**.   3. **Migraine**:   Recurrent headaches with nausea and photophobia are absent, but migraines can have atypical presentations (e.g., sudden onset). **Cannot exclude without further history of aura/triggers**.   4. **Pheochromocytoma**:   Episodic headaches with palpitations, hypertension, anxiety, and exertion-triggered episodes are classic. **Requires biochemical testing (plasma/urine metanephrines)**.   5. **Anxiety disorder**:   Anxiety and palpitations are present, but systemic symptoms (neck swelling, hypertension) suggest an organic cause. **Cannot exclude primary anxiety without ruling out other causes**.   6. **Cluster headaches**:   Atypical due to occipital location and lack of autonomic features (e.g., lacrimation, nasal congestion), but palpitations and abrupt onset overlap. **Less likely but not excluded**.   7. **Idiopathic intracranial hypertension (IIH)**:   Headaches worse with straining (Valsalva) and fatigue are consistent. Absence of papilledema does not fully exclude IIH. **Requires lumbar puncture for opening pressure**.   8. **Tension headache**:   Atypical given severity and sudden onset, but tension headaches can rarely mimic severe pain. **Less likely but possible**.  **Excluded Diagnoses**:   - **Giant cell arteritis**: Age <50 and no temporal artery tenderness/ESR elevation make this highly unlikely. - **Meningitis**: No fever, neck stiffness, or meningeal signs (negative Kernig/Brudzinski).   **Final Answer**: The following diagnoses **cannot be excluded** based on history and physical exam alone:   - Subarachnoid hemorrhage - Brain tumor - Migraine - Pheochromocytoma - Anxiety disorder - Cluster headaches - Idiopathic intracranial hypertension - Tension headache   **Excluded diagnoses**: Giant cell arteritis, meningitis. |
| Diag1 | **The following steps are essential to do immediately:**  **• CT scan of the head without IV contrast**   - **Rationale: The sudden-onset "worst headache of her life" raises concern for subarachnoid hemorrhage (SAH). A non-contrast CT head is the first-line imaging to detect acute blood. If negative but clinical suspicion remains, a lumbar puncture would follow.**   **• Complete metabolic profile (CMP)**   - **Rationale:**   **Assess for electrolyte imbalances (e.g., hypokalemia from catecholamine surges in pheochromocytoma).**  **Evaluate renal function (relevant if pheochromocytoma or hypertensive crisis is suspected).**  **Check blood glucose (catecholamines can cause hyperglycemia).**  **• Complete blood count (CBC)**   - **Rationale:**   **Rule out infection (e.g., meningitis, though less likely without fever or meningeal signs).**  **Assess for anemia or other hematologic abnormalities.**  **Why Other Options Are Not Immediately Essential:**   - **CT scan of the head with IV contrast:**   **Not indicated for acute SAH evaluation. Contrast is used for vascular imaging (e.g., CTA for aneurysm detection) after non-contrast CT.**   - **MRI of the head:**   **Less urgent than CT in the acute setting. MRI is more sensitive for posterior fossa lesions or chronic hemorrhage but is time-consuming and less accessible.**   - **Ibuprofen 600 mg po q 6 h:**   **Contraindicated in this case. NSAIDs can worsen hypertension or renal function, particularly if pheochromocytoma or renal impairment is suspected.**  **Key Considerations:**   1. **Pheochromocytoma workup (e.g., plasma-free metanephrines) should follow after initial stabilization and exclusion of life-threatening causes (e.g., SAH).** 2. **If CT head is negative for SAH but clinical suspicion persists, lumbar puncture is mandatory to rule out xanthochromia.** 3. **MRI/MRA brain may be necessary if CT is inconclusive or if a posterior fossa lesion (e.g., brain tumor) is suspected.**   **Immediate priority: CT head without contrast to exclude SAH, followed by CBC/CMP for baseline labs.** | **Essential Immediate Steps**: ✅ **CT scan of the head without IV contrast** ✅ **Complete blood count (CBC)** ✅ **Complete metabolic profile**  **Rationale:**   1. **CT Head Without Contrast**:   **Critical to rule out subarachnoid hemorrhage (SAH)** given the "worst headache of life" and sudden onset. Non-contrast CT is the **first-line imaging** for acute intracranial bleeding.   2. **CBC and Metabolic Profile**:   **Baseline labs** help assess for infection, anemia, electrolyte imbalances, or renal dysfunction.  **Metabolic profile** evaluates electrolytes, glucose, and renal/liver function, which are crucial for guiding further management (e.g., pheochromocytoma workup, contrast studies if needed later).  **Excluded Options:**  ❌ **CT Head With IV Contrast**: Not indicated for SAH evaluation. Contrast is reserved for tumor or vascular malformation suspicion after initial non-contrast CT. ❌ **Ibuprofen and Follow-Up**: Inappropriate without ruling out life-threatening causes (e.g., SAH, pheochromocytoma). ❌ **MRI of the Head**: Less urgent than CT in emergencies due to longer scan time and lower sensitivity for acute hemorrhage.  **Key Takeaway**:   - **Prioritize non-contrast CT** to exclude SAH. - **CBC and metabolic profile** provide critical baseline data for systemic evaluation. - Further testing (e.g., lumbar puncture, plasma metanephrines) depends on initial results. | **Essential Immediate Steps:**   1. **CT scan of the head without IV contrast**:   **Critical** to rule out subarachnoid hemorrhage (SAH), given the thunderclap headache ("worst of life") and sudden onset. Non-contrast CT is the first-line imaging for acute SAH.   2. **Complete blood count (CBC) and Complete metabolic profile**:   **Relevant** to assess for anemia, infection, electrolyte abnormalities, or renal dysfunction (e.g., underlying causes of hypertension or secondary headaches).  **Not Immediately Essential**:   - **CT scan of the head with IV contrast**: Not indicated for SAH; contrast is used for evaluating tumors or vascular malformations, which are less urgent here. - **MRI of the head**: Less accessible in emergencies and not first-line for SAH. May be deferred for later evaluation of alternative diagnoses (e.g., brain tumor, RCVS). - **Ibuprofen and discharge**: Contraindicated without excluding life-threatening causes (e.g., SAH, hypertensive crisis).   **Final Answer**:   - **CT scan of the head without IV contrast** - **Complete blood count (CBC)** - **Complete metabolic profile**   These steps prioritize ruling out SAH and initiating a basic metabolic workup. MRI and contrast CT are not urgent in this setting. |
| Diag2 | **The most appropriate next steps given the patient’s persistent symptoms and hypertensive crisis are:**  **• Lumbar puncture (LP)**   - **Rationale: A normal CT does not fully exclude subarachnoid hemorrhage (SAH). LP with CSF analysis for xanthochromia and RBC count is critical to confirm or rule out SAH.**   **• Plasma metanephrines and plasma normetanephrines**   - **Rationale: The episodic hypertension, palpitations, headache, and anxiety strongly suggest pheochromocytoma. Plasma-free metanephrines (which include both metanephrine and normetanephrine) are first-line tests for acute evaluation due to their high sensitivity.**   **• Obtain a 24-hour urine specimen and measure metanephrines**   - **Rationale: 24-hour urine fractionated metanephrines complement plasma testing and improve diagnostic accuracy for pheochromocytoma. Both plasma and urine tests are often performed together in high-suspicion cases.**   **Why Other Options Are Less Urgent:**   - **Serum renin and aldosterone ratio:**   **More relevant for primary aldosteronism (e.g., Conn’s syndrome), which presents with chronic hypertension and hypokalemia, not episodic symptoms. Less likely here.**  **Immediate Priorities:**   1. **LP to exclude SAH/meningitis.** 2. **Plasma metanephrines/normetanephrines and 24-hour urine metanephrines to confirm pheochromocytoma.** 3. **Avoid beta-blockers until pheochromocytoma is ruled out to prevent unopposed alpha-adrenergic stimulation.**   **Note: If pheochromocytoma is confirmed, urgent alpha-blockade (e.g., phenoxybenzamine) and blood pressure control are required. If LP is negative, consider MRI/MRA brain for structural causes (e.g., posterior fossa lesions) or neurology consultation for atypical migraine.** | **Essential Next Steps**: ✅ **Lumbar Puncture (LP)** ✅ **Plasma Metanephrines and Normetanephrines** ✅ **24-Hour Urine Metanephrines**  **Rationale:**   1. **Lumbar Puncture (LP)**:   **Critical to rule out subarachnoid hemorrhage (SAH)** despite the normal CT. A missed SAH can be catastrophic.  LP evaluates for **xanthochromia** or persistently elevated RBCs in CSF, which are diagnostic of SAH.   2. **Plasma Metanephrines/Normetanephrines**:   **First-line test for pheochromocytoma**, a key consideration given episodic hypertension, palpitations, headache, and anxiety. Elevated plasma-free metanephrines are highly sensitive for detecting catecholamine-secreting tumors.   3. **24-Hour Urine Metanephrines**:   **Complements plasma testing** for pheochromocytoma. Urine metanephrines have high specificity and are often used alongside plasma tests to confirm the diagnosis.  **Excluded Options:**  ❌ **Serum Renin and Aldosterone Ratio**:   - Indicated for suspected primary hyperaldosteronism (e.g., hypertension with hypokalemia), which does not align with this patient’s symptoms (palpitations, anxiety).   **Immediate Management:**   - **Control Hypertension**:   Use **IV labetalol** or **nicardipine** to lower BP (target systolic <140 mmHg) to reduce risks of complications (e.g., stroke, re-bleeding if SAH is confirmed).   - **Avoid NSAIDs** (e.g., ibuprofen) until SAH and pheochromocytoma are ruled out.   **Clinical Insight**:   - **Pheochromocytoma** must be urgently evaluated due to life-threatening hypertensive crises. - **SAH remains a "cannot miss" diagnosis** even with normal imaging. LP is mandatory if clinical suspicion persists. - Combining **plasma and urine metanephrines** maximizes diagnostic accuracy for pheochromocytoma. | **Essential Next Steps:**   1. **Plasma metanephrines** and **plasma normetanephrines**:   **Critical** to evaluate for **pheochromocytoma**, a catecholamine-secreting tumor causing paroxysmal hypertension, tachycardia, headache, and palpitations. Plasma free metanephrines are highly sensitive for acute diagnosis.   2. **24-hour urine metanephrines**:   **Gold standard** for confirming pheochromocytoma. While plasma tests provide rapid results, 24-hour urine collection offers higher specificity and accounts for episodic catecholamine surges.  **Why Not Other Options?**   - **Lumbar puncture (LP)**: A normal non-contrast CT makes subarachnoid hemorrhage (SAH) less likely. LP is only necessary if clinical suspicion for SAH persists (e.g., xanthochromia), but the current presentation strongly favors pheochromocytoma. - **Serum renin and aldosterone ratio**: Indicated for suspected primary hyperaldosteronism (e.g., hypokalemia, refractory hypertension), which does not explain the acute adrenergic symptoms (palpitations, anxiety, tachycardia).   **Immediate Management Considerations**:   - **Blood pressure control**: Use **alpha-blockers** (e.g., phenoxybenzamine) to manage hypertensive crisis. Avoid beta-blockers until alpha-blockade is established to prevent unopposed alpha-mediated vasoconstriction. - **Imaging**: If metanephrines are elevated, follow with **abdominal/pelvic CT or MRI** to localize a pheochromocytoma.   **Final Answer**:   - **Plasma metanephrines** - **Plasma normetanephrines** - **Obtain a 24-h urine specimen and measure metanephrines**   These steps prioritize diagnosing pheochromocytoma, the most likely cause of this patient’s acute hypertensive crisis with adrenergic symptoms. LP and renin/aldosterone testing are not aligned with the clinical picture. |
| Dx | **The most likely diagnosis is:**  **• Pheochromocytoma**  **Key Evidence:**   - **Markedly elevated plasma free metanephrines (100 nmol/L) and normetanephrines (180 nmol/L), which are >20-fold above normal. This is diagnostic for pheochromocytoma.** - **Normal CSF analysis (clear fluid, no xanthochromia, minimal RBCs) excludes subarachnoid hemorrhage.** - **Symptoms (hypertensive crisis, headache, palpitations, anxiety) align with catecholamine excess.**   **Why Other Options Are Incorrect:**   - **Subarachnoid hemorrhage: Ruled out by normal CSF (no RBCs, xanthochromia, or elevated protein).** - **Idiopathic intracranial hypertension: Requires opening pressure >250 mm H_2_O (patient’s pressure is 110 mm H_2_O, normal).** - **Migraine/Tension headache: Do not explain hypertensive crisis, tachycardia, or massively elevated metanephrines.**   **Conclusion: The biochemical confirmation of pheochromocytoma is unequivocal. Immediate management involves alpha-blockade, tumor localization, and surgical planning.** | **Correct Answer**: ✅ **Pheochromocytoma**  **Key Supporting Evidence:**   1. **Markedly Elevated Plasma Metanephrines/Normetanephrines**:   Plasma free metanephrines: **100 nmol/L** (normal <0.50 nmol/L).  Plasma free normetanephrines: **180 nmol/L** (normal <0.90 nmol/L).These levels are **diagnostic** for pheochromocytoma, a catecholamine-secreting tumor causing episodic hypertension, palpitations, headache, and anxiety.   2. **Normal CSF Analysis**:   Clear fluid, normal RBCs (2/mL), normal opening pressure (110 mm H2O), and normal protein/glucose **rule out subarachnoid hemorrhage (SAH)** and meningitis.   3. **Clinical Presentation**:   Hypertension (170/90 mmHg), tachycardia (110 bpm), headache, and anxiety align with catecholamine excess.  **Why Other Options Are Incorrect:**  ❌ **Idiopathic Intracranial Hypertension (IIH)**:   - Requires **elevated opening pressure** (>250 mm H2O) and papilledema. Normal CSF pressure (110 mm H2O) excludes this.   ❌ **Migraine** or **Tension Headache**:   - Do not explain hypertensive crises, palpitations, or massively elevated metanephrines.   ❌ **Subarachnoid Hemorrhage (SAH)**:   - CSF is clear with **no xanthochromia** and normal RBCs (2/mL), ruling out SAH.   **Next Step:**   - **Confirm tumor localization** with **abdominal/pelvic CT/MRI** and prepare for **surgical resection** (definitive treatment).   **Clinical Takeaway**: Pheochromocytoma is a **medical emergency** requiring prompt diagnosis and management to prevent life-threatening complications (e.g., stroke, arrhythmias). | **Final Answer**: **Pheochromocytoma**  **Explanation**: The **markedly elevated plasma free metanephrines (100 nmol/L)** and **normetanephrines (180 nmol/L)** are diagnostic of **pheochromocytoma**, a catecholamine-secreting tumor. This explains the patient’s paroxysmal hypertension, tachycardia, headache, palpitations, and anxiety.  **Why Not Other Options?**   - **Idiopathic intracranial hypertension (IIH)**: Requires **elevated opening pressure** (>250 mm H₂O) on lumbar puncture, which is absent here (opening pressure: 110 mm H₂O). - **Migraine/Tension headache**: Do not cause hypertensive crises, tachycardia, or catecholamine excess. - **Subarachnoid hemorrhage (SAH)**: CSF analysis would show **xanthochromia** or **high RBCs** (>1000/mL). This patient’s CSF is clear with only 2 RBCs/mL. - **Other diagnoses** (e.g., primary hyperaldosteronism, renal artery stenosis): Do not explain the adrenergic symptoms or elevated metanephrines.   The biochemical evidence of catecholamine excess (metanephrines/normetanephrines) is pathognomonic for pheochromocytoma. |
| Mang | **The appropriate management options for this patient with pheochromocytoma are:**  **• Admit the patient to the hospital**   - **Rationale: Hypertensive crises and catecholamine excess require inpatient monitoring and initiation of alpha-blockers.**   **• Phenoxybenzamine 10 mg po tid**   - **Rationale: First-line alpha-blockade to control catecholamine-mediated hypertension and prevent perioperative complications.**   **• MRI of the abdomen and pelvis**   - **Rationale: First-line imaging to localize the tumor (90% of pheochromocytomas are adrenal).**   **• ¹²³I-MIBG scintigraphy**   - **Rationale: Used if initial imaging (CT/MRI) is negative or to detect extra-adrenal/metastatic paragangliomas.**   **Why Other Options Are Incorrect:**   - **Propranolol 10 mg po q 6 h:**   **Beta-blockers are contraindicated unless alpha-blockade is already established (risk of unopposed alpha-mediated vasoconstriction). If started after phenoxybenzamine, it is appropriate, but the question does not clarify sequencing. Cautiously omitted due to potential misuse if given prematurely.**   - **Discharge on hydrochlorothiazide/ramipril/furosemide/ramipril alone:**   **These do not address catecholamine excess. ACE inhibitors/thiazides are ineffective for pheochromocytoma-induced hypertension.**   - **Morphine sulfate:**   **Opioids can trigger catecholamine release and worsen hypertension. Avoid.**   - **Ibuprofen:**   **NSAIDs are not contraindicated but do not address the underlying cause (catecholamine excess). Headaches should resolve with blood pressure control.**   - **Ultrasonography of the abdomen:**   **Less sensitive than CT/MRI for detecting small adrenal tumors.**   - **Consult interventional radiology for tumor embolization:**   **Definitive treatment is surgical resection. Embolization is not standard preoperatively.**  **Key Management Priorities:**   1. **Admit for alpha-blockade (phenoxybenzamine) and blood pressure stabilization.** 2. **Localize the tumor with MRI (first-line), followed by MIBG if needed.** 3. **Avoid beta-blockers until alpha-blockade is achieved (to prevent hypertensive crisis).**   **Final Selections:**   - **Admit the patient** - **Phenoxybenzamine** - **MRI of the abdomen/pelvis** - **¹²³I-MIBG scintigraphy** | **Appropriate Management Options**: ✅ **Admit the patient to the hospital**   - Pheochromocytoma requires **immediate hospitalization** for blood pressure control, monitoring, and preoperative preparation.   ✅ **Phenoxybenzamine 10 mg po tid**   - **Alpha-blockade (phenoxybenzamine)** is **first-line therapy** to stabilize blood pressure and prevent catecholamine-induced crises.   ✅ **MRI of the abdomen and pelvis**   - **Localization imaging** (CT/MRI) is mandatory to identify the tumor (pheochromocytoma or paraganglioma).   ✅ **¹²³I-MIBG scintigraphy**   - Functional imaging with **MIBG scan** is used if CT/MRI is inconclusive or to detect metastatic/extra-adrenal tumors.   **Incorrect Options:**  ❌ **Discharge on hydrochlorothiazide/ramipril**, **Furosemide**, **Ramipril alone**   - **ACE inhibitors/diuretics** are **not first-line** for pheochromocytoma. Alpha-blockers are required first.   ❌ **Propranolol**   - **Beta-blockers** should **only be added after alpha-blockade** to avoid unopposed alpha-receptor stimulation (risk of hypertensive crisis).   ❌ **Morphine sulfate** and **Ibuprofen**   - **Opioids** (morphine) and **NSAIDs** (ibuprofen) can **trigger catecholamine release** and worsen hypertension.   ❌ **Ultrasonography of the abdomen**   - Less sensitive than CT/MRI for detecting small adrenal tumors.   ❌ **Consult interventional radiology for tumor embolization**   - **Surgical resection** is definitive treatment. Embolization is not standard unless surgery is contraindicated.   **Key Takeaway**:   - **Alpha-blockers**, **hospitalization**, and **tumor localization** are critical steps. - Avoid medications that exacerbate catecholamine release or worsen hypertension. | **Appropriate Management Options for Pheochromocytoma:**   1. **Admit the patient to the hospital**   Hypertensive crises and arrhythmias require close monitoring during initiation of alpha-blockers and preoperative preparation.   2. **Phenoxybenzamine 10 mg po tid**   **First-line alpha-blocker** to control catecholamine-mediated hypertension and stabilize blood pressure preoperatively.   3. **Propranolol 10 mg po q 6 h**   **Beta-blockers** (e.g., propranolol) may be added **after alpha-blockade** to manage tachycardia. *Never use beta-blockers alone* due to risk of unopposed alpha-mediated vasoconstriction.   4. **MRI of the abdomen and pelvis**   **Localization imaging** (MRI or CT) is critical to identify the tumor location (adrenal vs. extra-adrenal).   5. **¹²³I-MIBG scintigraphy**   Functional imaging (e.g., MIBG or FDG-PET) is indicated if anatomic imaging (CT/MRI) is inconclusive or for metastatic disease evaluation.  **Why Other Options Are Inappropriate:**   - **Discharge on hydrochlorothiazide/ramipril or ramipril alone**: These do not address catecholamine excess and are ineffective for pheochromocytoma-induced hypertension. - **Furosemide**: Diuretics can worsen hypotension in the setting of alpha-blockade and volume contraction. - **Morphine/ibuprofen for headache**: Pain relief is secondary; addressing catecholamine excess (via alpha-blockers and tumor removal) is the priority. NSAIDs may exacerbate hypertension. - **Ultrasonography**: Less sensitive than CT/MRI for detecting small adrenal tumors. - **Consult interventional radiology for embolization**: Not standard pre-surgical management unless for large, hypervascular tumors requiring preoperative devascularization.   **Final Answer**:   - **Admit the patient to the hospital** - **Phenoxybenzamine 10 mg po tid** - **Propranolol 10 mg po q 6 h** - **MRI of the abdomen and pelvis** - **¹²³I-MIBG scintigraphy** |
| Case 22 |  |  |  |
| Diff | **The differential diagnosis for this patient’s painless, firm testicular mass includes:**  **• Testicular cancer**   - **Key features: Painless, firm, "marble-like" intratesticular mass in a young male. Lack of resolution over 1 month and normal prior ultrasound (4 years ago) further support malignancy.**   **• Epididymitis/orchitis**   - **Consideration: Though typically painful with dysuria/fever, *atypical or chronic cases* may present with minimal pain. However, the absence of tenderness, fever, or urinary symptoms makes this less likely.**   **• Spermatocele**   - **Consideration: A painless, fluid-filled cyst in the epididymis. However, the "firm" texture and intratesticular location (vs. epididymal) favor cancer.**   **• Testicular cyst**   - **Consideration: Rare intratesticular cysts are possible, but solid masses are more concerning for malignancy.**   **Why Other Options Are Less Likely:**   - **Hematocele: Requires trauma or bleeding, which is absent here.** - **Inguinal hernia: Presents as a groin bulge, not a testicular mass.** - **Testicular abscess: Associated with pain, fever, and erythema; absent in this case.** - **Testicular torsion: Acute, severe pain with sudden onset; inconsistent with a painless, stable mass.** - **Varicocele: Feels like a "bag of worms," worsens with standing, and is not firm.**   **Key Diagnostic Next Step:**  **Scrotal ultrasound is critical to:**   1. **Confirm if the mass is intratesticular (strongly suggests cancer) or extratesticular (e.g., spermatocele).** 2. **Assess for solid vs. cystic features.**   **Final Answer:**   - **Testicular cancer** - **Epididymitis/orchitis** - **Spermatocele** - **Testicular cyst** | **Differential Diagnosis for Painless Testicular Mass (Select all that apply):** ✅ **Testicular Cancer** ✅ **Spermatocele** ✅ **Varicocele**  **Rationale:**   1. **Testicular Cancer**:   **Most likely diagnosis** due to the **painless, firm, "marble-like" mass** in a young male (age 15–35 years).  Classic presentation of germ cell tumors (e.g., seminoma or nonseminoma).   2. **Spermatocele**:   A benign, **cystic mass** arising from the epididymis. Typically painless and smooth but **less likely here** due to the firm, intratesticular nature of the mass.   3. **Varicocele**:   Dilated veins in the pampiniform plexus ("bag of worms" texture). Often painless but **soft and compressible**, making this less likely given the firmness of the mass.  **Excluded Options:**  ❌ **Epididymitis/Orchitis**:   - Presents with **pain, fever, dysuria, or urinary symptoms** (absent here).   ❌ **Hematocele**:   - Blood in the tunica vaginalis, usually post-trauma (no history of injury).   ❌ **Inguinal Hernia**:   - Presents as a **groin bulge**, not an intratesticular mass.   ❌ **Testicular Abscess**:   - Associated with **pain, erythema, fever, or systemic infection** (none present).   ❌ **Testicular Cyst**:   - Fluid-filled and soft/compressible; the "marble-like" firmness suggests a solid mass.   ❌ **Testicular Torsion**:   - **Sudden, severe pain** with nausea/vomiting (acute onset, not chronic).   **Clinical Takeaway:**   - **Testicular cancer is the top priority** and must be ruled out with **urgent scrotal ultrasound** and tumor markers (AFP, β-hCG, LDH). - While spermatocele and varicocele are included in the differential, their features (cystic/soft texture) make them less likely in this case. - Immediate urology referral is critical if malignancy is suspected. | **Final Answer:**   - **Testicular cancer**   **Explanation:** The patient’s presentation of a **painless, firm, intratesticular mass** in a young male (age 28) is **classic for testicular cancer**, the most common malignancy in males aged 15–35. Key findings supporting this diagnosis include:   - **Hard, nontender mass** in the right testis (noted for 1 month without resolution). - Absence of infectious symptoms (e.g., fever, dysuria) or trauma, ruling out epididymitis/orchitis, abscess, or hematocele. - Normal prior ultrasound 4 years ago, suggesting the mass is new and not congenital.   **Why Other Options Are Less Likely:**   - **Epididymitis/orchitis**: Typically causes **pain, tenderness, and systemic symptoms** (e.g., fever), which are absent here. - **Hematocele/testicular abscess**: Requires trauma or infection, respectively; neither is reported. - **Inguinal hernia**: Mass would be in the inguinal canal, not the testis. - **Spermatocele/testicular cyst**: Usually **soft, fluctuant, and located in the epididymis** (spermatocele) or tunica albuginea (cyst), not a firm intratesticular mass. - **Testicular torsion**: Presents with **sudden, severe pain** (acute emergency), inconsistent with this painless, stable mass. - **Varicocele**: Feels like a "bag of worms," typically left-sided, and worsens with standing.   **Conclusion:** While the differential for a testicular mass often includes benign entities (e.g., cysts), the **hard, painless nature** of this lesion overwhelmingly prioritizes **testicular cancer** as the primary diagnosis. Urgent scrotal ultrasound and urology referral are critical next steps. |
| Diag | **The most appropriate next step in managing this patient is:**  **• Scrotal ultrasound**   - **Rationale: The gold-standard initial imaging for a testicular mass. It can confirm if the mass is intratesticular (strongly suggesting malignancy) or extratesticular (e.g., spermatocele, cyst) and distinguish solid vs. cystic lesions.**   **Other Options and Why They Are Less Appropriate:**   - **Urinalysis and culture:**   **May be considered to rule out subclinical infection (e.g., epididymitis), but the absence of pain, dysuria, or fever makes infection unlikely.**  **Not the immediate priority compared to ultrasound.**   - **Biopsy of testis:**   **Contraindicated due to the risk of tumor seeding. Definitive diagnosis is made via radical inguinal orchiectomy if cancer is suspected.**   - **MRI of pelvis and scrotum:**   **Not first-line. Ultrasound is sufficient for initial evaluation. MRI may be used later for staging if cancer is confirmed.**   - **STD panel/CBC:**   **Unnecessary in the absence of symptoms (e.g., urethral discharge, fever, systemic illness).**  **Final Answer:**   - **Scrotal ultrasound (most critical next step).** - **Urinalysis and culture (optional but low yield given the clinical presentation).**   **Immediate Action: Proceed with scrotal ultrasound to guide further management. If malignancy is confirmed, urgent urology referral for orchiectomy is indicated.** | **Most Appropriate Next Steps**: ✅ **Scrotal Ultrasound** ✅ **Urinalysis and Culture**  **Explanation:**   1. **Scrotal Ultrasound**:   **First-line imaging** for evaluating a testicular mass. It can distinguish between solid vs. cystic lesions, assess blood flow, and guide further management (e.g., solid masses suggest cancer, while cystic lesions may indicate spermatocele or cyst).  **Critical for ruling out testicular cancer**, which is the most concerning diagnosis in this case.   2. **Urinalysis and Culture**:   Helps exclude **infection** (e.g., epididymitis/orchitis), though less likely in a painless mass.  Provides baseline urinary evaluation if symptoms like dysuria emerge later.  **Excluded Options:**  ❌ **Biopsy of Testis**:   - **Contraindicated** in suspected testicular cancer due to risk of tumor seeding. Definitive diagnosis is made via **radical orchiectomy** (if ultrasound and tumor markers suggest malignancy).   ❌ **MRI of Pelvis and Scrotum**:   - Not first-line. Reserved for equivocal ultrasound findings or staging after cancer is confirmed.   ❌ **STD Panel**:   - Indicated for **painful scrotal swelling** with dysuria/urethral discharge (not relevant here).   ❌ **CBC**:   - Low yield in a painless mass without systemic symptoms (e.g., fever, weight loss).   **Additional Considerations (Not Listed):**   - **Tumor Markers**:   **AFP, β-hCG, and LDH** should be ordered if cancer is suspected (e.g., elevated in germ cell tumors).   - **Urology Referral**:   Urgently needed if ultrasound suggests malignancy.  **Clinical Takeaway:**   - **Scrotal ultrasound is mandatory** to characterize the mass. - Urinalysis helps exclude infection but is secondary to imaging. - Avoid testicular biopsy; proceed to orchiectomy if cancer is confirmed. | **Final Answer:**   - **Scrotal ultrasound**   **Explanation:** The **most critical next step** is a **scrotal ultrasound**, which is the gold standard for evaluating a testicular mass. It can:   1. Confirm if the mass is **intratesticular** (vs. extratesticular, e.g., spermatocele). 2. Assess characteristics (e.g., solid vs. cystic, vascularity) to stratify malignancy risk. 3. Guide further management (e.g., urgent urology referral for suspected cancer).   **Why Other Options Are Less Appropriate:**   - **Biopsy of testis**: Contraindicated due to risk of tumor seeding; **radical inguinal orchiectomy** is the definitive diagnostic and therapeutic procedure for suspected testicular cancer. - **MRI of pelvis/scrotum**: Not first-line; reserved for equivocal ultrasound findings or staging after cancer diagnosis. - **Urinalysis/culture, STD panel**: Unnecessary here due to **absence of urinary/STI symptoms** (e.g., dysuria, discharge, fever). - **CBC**: Non-specific; not urgent unless systemic infection or anemia is suspected (neither is present).   **Next Steps After Ultrasound:**   1. **Serum tumor markers** (AFP, β-hCG, LDH) to support diagnosis and subtype classification. 2. **Urology referral** for possible radical orchiectomy if ultrasound confirms a solid intratesticular mass.   **Conclusion:** Scrotal ultrasound is the **immediate priority** to confirm/testicular cancer suspicion. Other tests (e.g., tumor markers) should follow but are not listed as options here. |
| Dx | **The most likely cause of a solid testicular mass in this patient is:**  **• Testicular cancer**   - **Rationale:**   **A solid intratesticular mass on ultrasound is highly suspicious for malignancy (testicular cancer) until proven otherwise.**  **Testicular cancer (e.g., seminoma, nonseminoma) is the most common solid testicular tumor in young males (15–35 years).**  **Ultrasound findings (solid, hypoechoic lesion with irregular vascularity) strongly differentiate cancer from benign lesions.**  **Why Other Options Are Incorrect:**   - **Hydrocele: A fluid-filled sac around the testicle (not a solid mass). Appears anechoic on ultrasound.** - **Inguinal hernia: Involves bowel or fat protruding into the scrotum. Typically reducible, and ultrasound shows bowel loops or fat, not a testicular mass.** - **Testicular cyst: A fluid-filled lesion (e.g., epididymal cyst). Appears anechoic with posterior acoustic enhancement on ultrasound.**   **Key Point:**  **In adults, all solid intratesticular masses are presumed malignant until histologically proven otherwise. Immediate urology referral for radical orchiectomy is critical.**  **Final Answer: Testicular cancer.** | **Most Likely Diagnosis**: ✅ **Testicular Cancer**  **Explanation:**   1. **Testicular Cancer**:   A **solid testicular mass** on ultrasound is **presumed to be malignant** until proven otherwise.  It is the **most common cause of a painless, solid intratesticular mass**, especially in men aged 15–35 years.  **Key features**: Firm, non-transilluminating mass, often painless.  **Excluded Options:**  ❌ **Hydrocele**:   - Fluid-filled sac **around** the testicle (not a solid mass). Ultrasound shows **anechoic fluid**, not a solid lesion.   ❌ **Inguinal Hernia**:   - Protrusion of bowel or fat into the scrotum. Presents as a **groin/scrotal bulge**, not a solid testicular mass. Ultrasound would show bowel loops or fat, not a testicular lesion.   ❌ **Testicular Cyst**:   - Cysts (e.g., epididymal cyst, spermatocele) are **fluid-filled** and appear **anechoic** on ultrasound. A "solid mass" rules this out.   **Clinical Takeaway:**   - **Testicular cancer is the top concern** in a patient with a solid intratesticular mass. Urgent urology referral, tumor markers (AFP, β-hCG, LDH), and radical orchiectomy are required for diagnosis and treatment. - Benign causes (e.g., cysts, hydrocele) are typically cystic or extratesticular. | **Final Answer:**   - **Testicular cancer**   **Explanation:** A **solid testicular mass** identified on ultrasound in a young male is **most likely testicular cancer**. This diagnosis is supported by:   1. **Ultrasound findings**: Solid intratesticular lesions are highly concerning for malignancy (e.g., germ cell tumors like seminoma or non-seminoma). 2. **Demographics**: Testicular cancer is the most common solid malignancy in males aged 15–35 years. 3. **Clinical context**: The absence of symptoms suggesting infection (e.g., pain, fever) or cystic features (e.g., hydrocele, spermatocele) further supports cancer.   **Why Other Options Are Incorrect:**   - **Hydrocele** or **testicular cyst**: Both are fluid-filled, not solid, and would appear **anechoic/cystic** on ultrasound. - **Inguinal hernia**: Presents as a reducible groin/scrotal swelling containing bowel or fat, not a solid testicular mass. - **Epididymitis/orchitis**: Typically causes **pain, swelling, and fever**, with ultrasound showing inflammation (e.g., hypervascularity), not a discrete solid mass.   **Conclusion:** Testicular cancer is the **leading diagnosis** for a solid testicular mass in this patient. Urgent urologic evaluation and management (e.g., radical orchiectomy) are critical. |
| Mang | **The most appropriate next steps after confirming a diagnosis of testicular cancer are:**  **1. Urology consultation**   - **Rationale:**   **Immediate referral for definitive management (e.g., radical inguinal orchiectomy, the gold standard surgery).**  **Trans-scrotal orchiectomy is contraindicated due to the risk of tumor seeding.**  **2. CT scan of the abdomen and pelvis**   - **Rationale:**   **Evaluates retroperitoneal lymph nodes, the most common site of metastasis.**  **3. CT scan of the chest**   - **Rationale:**   **More sensitive than a chest X-ray for detecting pulmonary metastases.**  **4. Serum tumor markers**   - **Alpha-fetoprotein (AFP)** - **Beta-human chorionic gonadotropin (β-hCG)** - **Lactate dehydrogenase (LDH)** - **Rationale:**   **Post-diagnosis marker levels guide staging, prognosis, and monitoring response to therapy.**  **Why Other Options Are Incorrect:**   - **Discharge with repeat imaging in 4 weeks: Unacceptable delay in treatment. Testicular cancer requires urgent intervention.** - **Trans-scrotal orchiectomy: Risk of tumor spread; radical inguinal approach is mandatory.** - **Bone scan: Not routine unless symptoms suggest bone metastases (e.g., pain, elevated alkaline phosphatase).** - **Chest X-ray: Less sensitive than CT chest for detecting small metastases.** - **Ciprofloxacin: Antibiotics are unnecessary unless there is concurrent infection (not mentioned here).** - **Serum amylase/testosterone: Not relevant to staging or management.** - **CBC: May be part of general pre-op assessment but is not a priority in this context.**   **Key Points:**   - **Radical inguinal orchiectomy is both diagnostic (confirms histology) and therapeutic.** - **Post-surgical staging (CT scans + tumor markers) determines the need for adjuvant therapy (e.g., chemotherapy, radiation, or surveillance).**   **Final Answer:**   - **Urology consultation** - **CT scan of the abdomen** - **CT scan of the chest** - **Serum alfa-fetoprotein level** - **Serum beta HCG level** - **Serum LDH level** | **Most Appropriate Next Steps**: ✅ **Urology consultation** ✅ **CT scan of the abdomen** ✅ **CT scan of the chest** ✅ **Serum alfa-fetoprotein (AFP) level** ✅ **Serum beta HCG level** ✅ **Serum LDH level**  **Explanation:**   1. **Urology Consultation**:   Immediate referral to a urologist is critical for definitive management (e.g., **radical inguinal orchiectomy**, the gold standard treatment).   2. **Staging Imaging**:   **CT abdomen/pelvis**: Evaluates retroperitoneal lymph nodes (the most common site of metastasis).  **CT chest** (or chest X-ray if CT unavailable): Assesses for pulmonary metastases.   3. **Tumor Markers**:   **AFP, β-hCG, LDH**: Required for risk stratification, guiding treatment (e.g., chemotherapy), and monitoring response.  **Excluded Options:**  ❌ **Discharge with repeat exam/ultrasound in 4 weeks**:   - **Unacceptable delay** for a confirmed cancer. Immediate treatment is required.   ❌ **Trans-scrotal orchiectomy**:   - **Contraindicated** due to risk of tumor seeding. Radical **inguinal** orchiectomy is the standard.   ❌ **Bone scan**:   - Not routinely indicated unless symptoms (e.g., bone pain) suggest metastasis.   ❌ **Ciprofloxacin**:   - Antibiotics are unnecessary unless there is concurrent infection (not described here).   ❌ **Serum amylase/testosterone level**:   - Not relevant to testicular cancer diagnosis or staging.   ❌ **CBC**:   - May be checked but is not specific to testicular cancer management.   **Clinical Takeaway:**   - **Radical inguinal orchiectomy** is definitive treatment and confirms histology (seminoma vs. nonseminoma). - Staging with **CT imaging** and **tumor markers** guides further therapy (e.g., surveillance, chemotherapy, radiation). - Delaying intervention worsens prognosis—**survival exceeds 95% with prompt treatment**. | **Final Answer:**   - **Urology consultation** - **CT scan of the abdomen** - **CT scan of the chest** - **Serum alfa-fetoprotein (AFP) level** - **Serum beta HCG level** - **Serum LDH level**   **Explanation:** After confirming testicular cancer, the **most appropriate next steps** focus on **staging the disease** and **initiating treatment**:   1. **Urology consultation**:   Essential for definitive management, including **radical inguinal orchiectomy** (the standard surgical treatment). Trans-scrotal orchiectomy is **contraindicated** due to risk of tumor seeding.   2. **Staging imaging**:   **CT scan of the abdomen/pelvis**: Evaluates retroperitoneal lymph nodes (the most common site of metastasis).  **CT scan of the chest**: More sensitive than chest X-ray for detecting pulmonary metastases.   3. **Serum tumor markers**:   **AFP**, **beta HCG**, and **LDH**: Baseline levels are critical for:  Subclassifying the tumor (seminoma vs. non-seminoma).  Staging and prognosis.  onitoring response to therapy.  **Why Other Options Are Incorrect:**   - **Discharge with repeat imaging in 4 weeks**: Inappropriate delay; immediate staging and treatment are required. - **Trans-scrotal orchiectomy**: Risk of tumor spread; radical inguinal approach is standard. - **Bone scan**: Not routine unless symptoms suggest bone metastases (uncommon in early-stage disease). - **Chest X-ray**: Less sensitive than CT chest; guidelines increasingly favor CT for staging. - **Serum amylase/testosterone/CBC/Ciprofloxacin**: Not relevant to testicular cancer staging or management unless specific indications exist (e.g., infection, which is not described here).   **Next Steps Post-Staging:**   - Radical inguinal orchiectomy. - Tailored adjuvant therapy (chemotherapy, radiation, or surveillance) based on tumor type and stage.   **Conclusion:** Immediate **staging with CT imaging**, **tumor marker assessment**, and **urology referral** are critical to guide definitive treatment and optimize outcomes. |
| Case 23 |  |  |  |
| Diff | **The differential diagnoses for this 10-year-old boy with acute right-sided scrotal pain include:**  **1. Testicular Torsion**   - **Key Features: Sudden onset of pain (awakening from sleep), no trauma, and normal exam early in the process.** - **Rationale: A surgical emergency requiring immediate evaluation with Doppler ultrasound and urology consultation.**   **2. Torsion of the Testicular Appendage**   - **Key Features: More gradual pain onset, possible "blue dot sign" (necrosis of the appendage), but early stages may mimic testicular torsion.** - **Rationale: Common in prepubertal boys (7–12 years old), often diagnosed via ultrasound.**   **3. Incarcerated Inguinal Hernia**   - **Key Features: Acute scrotal pain from herniated bowel trapped in the inguinal canal.** - **Rationale: Absence of prior hernia history or abdominal symptoms does not exclude it in pediatric patients (often congenital).**   **4. Epididymitis**   - **Key Features: Inflammation/infection of the epididymis, typically with dysuria, fever, or urinary symptoms.** - **Rationale: Less likely here (no urinary symptoms), but younger patients may present atypically.**   **5. Orchitis**   - **Key Features: Testicular inflammation (viral or bacterial), often with systemic symptoms (e.g., fever, swelling).** - **Rationale: Unlikely without fever or swelling, but still considered in the differential.**   **Why Other Options Are Less Likely:**   - **Testicular Fracture: Requires significant trauma (excluded by history).** - **Testicular Cancer: Typically painless mass; rare in this age group.** - **Nephrolithiasis: Flank/abdominal pain and hematuria are absent.**   **Final Answer:**   - **Testicular Torsion** - **Torsion of the Testicular Appendage** - **Incarcerated Inguinal Hernia** - **Epididymitis** - **Orchitis** | **Differential Diagnoses for Acute Scrotal Pain in a 10-Year-Old**: ✅ **Testicular torsion** ✅ **Torsion of the testicular appendage** ✅ **Epididymitis** ✅ **Orchitis** ✅ **Incarcerated inguinal hernia**  **Key Rationale:**   1. **Testicular Torsion**:   **Most urgent diagnosis** given sudden onset, severe pain, and age.  Physical exam may initially lack swelling or masses (early torsion).   2. **Torsion of the Testicular Appendage**:   Classic in prepubertal boys (peak age 7–12 years).  May present with localized tenderness ("blue dot sign") if the appendage is visible.   3. **Epididymitis/Orchitis**:   Less likely without fever, dysuria, or urethral discharge, but infections can occur in children without classic symptoms.   4. **Incarcerated Inguinal Hernia**:   A surgical emergency; absence of a palpable mass on exam reduces likelihood but does not exclude it.  **Less Likely but Still Consider:**  ❌ **Testicular Cancer**:   - Typically presents with a **painless mass**, not acute pain. ❌ **Testicular Fracture**: - Requires **trauma** (excluded by history). ❌ **Nephrolithiasis**: - Pain usually radiates from flank to groin; absence of hematuria or urinary symptoms makes this unlikely.   **Clinical Priorities:**   - **Testicular torsion** is a **time-sensitive emergency**; delay in treatment risks testicular loss. - **Doppler ultrasound** and **urgent urology consult** are critical to confirm/exclude torsion. - **Epididymitis** and **appendage torsion** may mimic each other, but management differs (antibiotics vs. observation).   **Immediate Action**: Proceed to imaging (scrotal ultrasound with Doppler) and involve urology immediately. | **Final Answer:**   - **Testicular torsion** - **Torsion of the testicular appendage** - **Epididymitis/Orchitis**   **Explanation:** The acute onset of severe scrotal pain in a pediatric patient, especially without trauma or systemic symptoms, prioritizes urgent conditions:   1. **Testicular torsion**:   Sudden, severe pain waking the patient from sleep is classic.  Physical exam may lack signs early (e.g., high-riding testis, swelling), but history alone warrants immediate evaluation.   2. **Torsion of the testicular appendage**:   Common in prepubertal boys (7–14 years).  Pain may localize to the upper pole of the testis; the "blue dot sign" (ischemic appendage) can be absent initially.   3. **Epididymitis/Orchitis**:   Less likely due to the absence of fever, dysuria, or discharge, but pain can precede swelling or systemic symptoms.  **Excluded Diagnoses:**   - **Incarcerated inguinal hernia**: No palpable mass, abdominal pain, or gastrointestinal symptoms. - **Testicular cancer**: Typically a **painless mass**, not acute pain. - **Testicular fracture**: Requires trauma (explicitly denied). - **Nephrolithiasis**: No flank pain, hematuria, or urinary symptoms.   **Next Steps:**   - **Urgent scrotal ultrasound with Doppler** to assess blood flow. - **Urology consultation** if torsion is suspected.   **Conclusion:** Testicular torsion and torsion of the appendage are **time-sensitive emergencies**. Even with a normal exam, the history mandates ruling these out. Epididymitis/orchitis, though less likely, remains a consideration. |
| Diag | **The most appropriate tests to guide diagnosis and management in this patient are:**  **1. Doppler Ultrasonography of the Scrotum**   - **Priority: First-line test to evaluate for testicular torsion (assesses blood flow) and differentiate from other causes (e.g., appendage torsion, epididymitis).**   **2. Urinalysis and Culture**   - **Rationale: To exclude epididymitis or orchitis, which can present with scrotal pain even in the absence of classic urinary symptoms.**   **Why Other Tests Are Not Indicated:**   - **Basic Metabolic Profile (BMP): No dehydration, electrolyte imbalance, or renal dysfunction suggested.** - **Complete Blood Count (CBC): No fever or systemic signs of infection to warrant evaluation for leukocytosis.** - **Erythrocyte Sedimentation Rate (ESR): Non-specific marker of inflammation; not helpful in acute scrotal pain without systemic signs.** - **Urethral Swab for STDs: Unnecessary given the patient’s age, lack of sexual activity, and absence of urethral discharge.**   **Final Answer:**   - **Doppler Ultrasonography of the Scrotum** - **Urinalysis and Culture** | **Most Appropriate Tests**: ✅ **Doppler Ultrasonography of the Scrotum** ✅ **Urinalysis and Culture**  **Rationale:**   1. **Doppler Ultrasonography**:   **Gold standard** for evaluating testicular torsion.  Assesses blood flow to the testes; absent/reduced flow confirms torsion.  Also identifies torsion of the appendage (e.g., "blue dot sign").   2. **Urinalysis and Culture**:   Rules out **urinary tract infection (UTI)** or **epididymitis/orchitis**, which can mimic torsion.  Although the patient lacks classic urinary symptoms (dysuria, frequency), infection must still be excluded.  **Tests Not Indicated:**  ❌ **Basic Metabolic Profile (BMP)**:   - No signs of electrolyte imbalance, dehydration, or nephrolithiasis (e.g., no flank pain, hematuria). ❌ **Complete Blood Count (CBC)**/**ESR**: - Inflammatory markers are nonspecific and less urgent in the absence of fever or systemic signs of infection. ❌ **Urethral Swab for STDs**: - No history of sexual activity or urethral discharge; inappropriate in this context.   **Clinical Priorities:**   - **Doppler ultrasound** is **emergent** to confirm/exclude torsion (time-sensitive diagnosis). - **Urinalysis** helps differentiate infectious causes (e.g., epididymitis) from torsion. - Immediate urology consultation is required if torsion is suspected, even before imaging results.   **Next Steps**: Proceed to ultrasound and urinalysis while preparing for potential surgical exploration. | **Final Answer:**   - **Doppler ultrasonography of the scrotum** - **Urinalysis and culture**   **Explanation:** Given the acute scrotal pain and concern for time-sensitive diagnoses (e.g., testicular torsion), the following tests are critical:   1. **Doppler ultrasonography of the scrotum**:   **Gold standard** for evaluating testicular torsion. Assesses blood flow to the testicle and identifies torsion or appendage torsion.  Urgently required due to the risk of testicular loss if torsion is not surgically corrected within hours.   2. **Urinalysis and culture**:   Helps evaluate for epididymitis/orchitis, which can present with scrotal pain.  Though the patient lacks urinary symptoms (e.g., dysuria, fever), urinalysis is non-invasive and can detect pyuria or bacteriuria.  **Excluded Tests:**   - **CBC/ESR**: Inflammatory markers are low yield without fever or systemic signs of infection. - **BMP**: No electrolyte abnormalities or renal dysfunction suggested (e.g., no vomiting, dehydration, or nephrolithiasis symptoms). - **Urethral swab**: No history of sexual activity or urethral discharge.   **Conclusion:** Immediate **Doppler ultrasound** is mandatory to rule out torsion. **Urinalysis** supports evaluation for infection. Other tests (CBC, BMP, ESR, STD testing) are unnecessary in this clinical context. |
| Dx | **Answer: Torsion of the Testicular Appendage**  **Rationale:**   1. **Ultrasound Findings:**   **Microcystic lesion adjacent to the epididymal head with no blood flow is classic for a necrotic, torsed appendage (e.g., appendix epididymis or appendix testis).**  **Normal testicular blood flow excludes testicular torsion.**   2. **Normal Urinalysis:**   **Rules out epididymitis or orchitis (typically associated with infection, pyuria, or bacteriuria).**   3. **Key Exclusion of Other Diagnoses:**   **Testicular torsion: Normal blood flow to the testes.**  **Epididymitis/Orchitis: No evidence of inflammation or infection.**  **Testicular cancer: Cystic lesion (not solid) and no vascularity.**  **Final Diagnosis: Torsion of the Testicular Appendage.** | **Correct Diagnosis**: ✅ **Torsion of the Testicular Appendage**  **Explanation:**   - **Key Imaging Findings**:   **Microcystic lesion adjacent to the epididymal head with absent flow** is pathognomonic for a torsed testicular appendage (e.g., appendix testis or appendix epididymis).  **Normal testicular blood flow** definitively excludes testicular torsion.   - **Clinical Correlation**:   Acute scrotal pain without urinary symptoms or systemic signs of infection.  Normal urinalysis rules out epididymitis/orchitis.  Acute pain and cystic lesion are inconsistent with testicular cancer (typically painless and solid).  **Why Other Options Are Incorrect**:   - ❌ **Epididymitis/Orchitis**: Requires urinary symptoms (dysuria, frequency) or abnormal urinalysis (pyuria, bacteriuria). - ❌ **Testicular Torsion**: Excluded by normal blood flow to the testes on Doppler ultrasound. - ❌ **Testicular Cancer**: Presents as a painless, firm mass; cystic lesions are not typical.   **Next Step**: Conservative management with pain control and follow-up. | **Final Answer:**   - **Torsion of the testicular appendage**   **Explanation:** The **ultrasound findings** (non-vascularized microcystic lesion near the epididymal head) and **normal urinalysis** confirm:   1. **Torsion of the testicular appendage** (e.g., appendix testis or appendix epididymis), a common cause of acute scrotal pain in pediatric patients. 2. **Normal testicular blood flow** excludes testicular torsion. 3. **No urinary or systemic signs** of infection (normal urinalysis) rule out epididymitis/orchitis. 4. **Cystic lesion** (not a solid mass) makes testicular cancer unlikely.   **Conclusion:** The patient’s presentation and imaging are classic for **torsion of the testicular appendage**, a self-limiting condition managed conservatively with pain control and monitoring. |
| Mang | **Appropriate Management Options: ✅ Discharge with follow-up in 3 days ✅ Apply ice packs and prescribe supportive underwear ✅ Prescribe oral ibuprofen 800 mg every 8 hours**  **Rationale:**   1. **Conservative Management:**   **Discharge with follow-up is standard, as most cases resolve spontaneously.**  **Ice packs reduce swelling, and supportive underwear (e.g., scrotal support) minimizes discomfort.**  **NSAIDs (ibuprofen 800 mg every 8 hours) address pain and inflammation effectively.**   2. **Excluded Options:**   **❌ Admission for surgery: Reserved for persistent symptoms or diagnostic uncertainty (not indicated here).**  **❌ Ciprofloxacin: No evidence of infection (normal urinalysis).**  **❌ Ibuprofen 300 mg every 6 hours: Lower dose than typically prescribed for acute pain in adults.**  **Summary: Conservative measures (pain control, ice, support) with outpatient follow-up are sufficient for uncomplicated torsion of the testicular appendage.** | **Correct Management Options**: ✅ **Discharge with follow-up in 3 days** ✅ **Apply ice packs and prescribe supportive underwear** ✅ **Prescribe oral ibuprofen 300 mg every 6 hours** ✅ **Prescribe oral ibuprofen 800 mg every 8 hours**  **Explanation:**   1. **Discharge with follow-up in 3 days**:   Torsion of the testicular appendage is managed conservatively. Follow-up ensures resolution of symptoms and rules out complications.   2. **Ice packs and supportive underwear**:   Ice reduces swelling and pain; supportive underwear (e.g., snug briefs) minimizes scrotal movement for comfort.   3. **Oral ibuprofen (both doses)**:   NSAIDs are critical for pain and inflammation.  **300 mg every 6 hours**: Appropriate for mild-moderate pain or pediatric/weight-based dosing.  **800 mg every 8 hours**: Standard for adults with moderate-severe pain (within safe daily limits).  **Why Other Options Are Incorrect:**  ❌ **Admission for surgical repair**: Surgery is reserved for diagnostic uncertainty or complications (e.g., testicular torsion), not confirmed appendage torsion.  ❌ **Ciprofloxacin**: No evidence of infection (normal urinalysis), so antibiotics are unnecessary.  **Key Point**: Both ibuprofen regimens are valid depending on patient age/weight and pain severity. Conservative management with NSAIDs, ice, and follow-up is the standard approach. | **Correct Management Options:**   - **Discharge with follow-up in 3 days** - **Apply ice packs and prescribe supportive underwear** - **Prescribe oral ibuprofen 300 mg every 6 hours**   **Explanation:**   1. **Discharge with follow-up**: Torsion of the testicular appendage is self-limiting and does not require hospitalization unless complications arise. Outpatient management with follow-up is standard. 2. **Ice packs and supportive underwear**: These measures reduce swelling and pain, promoting comfort during recovery. 3. **Oral ibuprofen 300 mg every 6 hours**: NSAIDs like ibuprofen are first-line for pain and inflammation. The 300 mg dose is appropriate for **pediatric patients** (consistent with the initial case context).   **Excluded Options:**   - **Admission for surgical repair**: Reserved for severe or persistent pain (rarely needed). - **Ciprofloxacin**: No evidence of infection (normal urinalysis). - **Ibuprofen 800 mg every 8 hours**: Typically for adults; the lower dose (300 mg) aligns better with pediatric dosing.   **Conclusion:** Conservative management with analgesia, supportive care, and follow-up is the standard approach. Surgical intervention or antibiotics are unnecessary in uncomplicated cases. |
| Case 24 |  |  |  |
| Diff | **Differential Diagnoses That Cannot Be Excluded: Epididymitis or orchitis  Testicular torsion  Testicular appendage torsion  Testicular cancer**  **Rationale:**   1. **Epididymitis/Orchitis:**   **Sexual history (multiple partners, unprotected sex) raises concern for STIs (e.g., chlamydia/gonorrhea), which can cause epididymitis.**  **Epididymal tenderness on exam supports this diagnosis, even in the absence of fever or urinary symptoms.**   2. **Testicular Torsion:**   **Gradual pain onset and cremasteric reflexes present make this less typical, but torsion cannot be definitively excluded without Doppler ultrasound.**  **Partial/intermittent torsion may preserve cremasteric reflexes.**   3. **Testicular Appendage Torsion:**   **Compatible history: Gradual pain, localized tenderness, and swelling.**  **Diagnosis is clinical but often confirmed with ultrasound.**   4. **Testicular Cancer:**   **Acute pain and swelling are uncommon but possible with hemorrhage or rapid tumor growth.**  **A mass may not be palpable early in the disease course.**  **Excluded Diagnoses:**   - **Incarcerated Inguinal Hernia: No inguinal tenderness/fullness on exam.** - **Musculoskeletal Strain/Sprain: Pain localized to the testicle (not groin/muscle); exam findings suggest scrotal pathology.** - **Nephrolithiasis: No flank pain, hematuria, or costovertebral angle tenderness.** - **Testicular Fracture: No history of trauma.**   **Key Takeaway: While testicular appendage torsion is the most likely diagnosis, the history and exam alone cannot rule out other critical conditions like testicular torsion (a surgical emergency) or epididymitis (requiring antibiotics if confirmed). Testicular cancer remains a less likely but possible consideration until imaging (e.g., scrotal ultrasound) is performed.** | **Differential Diagnoses That Cannot Be Excluded**: ✅ **Epididymitis or orchitis** ✅ **Testicular torsion** ✅ **Testicular appendage torsion** ✅ **Testicular cancer**  **Explanation:**   1. **Epididymitis/Orchitis**:   Despite the absence of dysuria, fever, or urethral discharge, the patient’s sexual history (multiple partners, unprotected sex) raises concern for STIs. Early or atypical infections may lack classic symptoms.   2. **Testicular Torsion**:   **Cremasteric reflexes are present**, making torsion less likely, but not definitive. Partial/intermittent torsion or delayed presentation (3 days) can still occur. Testicular torsion is a "cannot miss" diagnosis requiring ultrasound confirmation.   3. **Testicular Appendage Torsion**:   The most likely diagnosis given gradual pain onset, localized tenderness, and swelling. However, other conditions (e.g., epididymitis, torsion) must be excluded.   4. **Testicular Cancer**:   Typically painless, but rapid growth or hemorrhage can cause pain. Scrotal swelling may mimic other pathologies; imaging is required to rule out a mass.  **Excluded Diagnoses:**   - **Incarcerated Inguinal Hernia**: No inguinal tenderness/fullness, normal abdominal exam. - **Nephrolithiasis**: No flank pain, hematuria, or CVA tenderness. - **Testicular Fracture**: No history of trauma. - **Musculoskeletal Strain/Sprain**: Localized scrotal swelling and tenderness are inconsistent with muscle strain.   **Key Point**: While testicular appendage torsion is the leading diagnosis, **definitive exclusion of torsion, epididymitis, and cancer requires imaging (Doppler ultrasound)**. Clinical judgment alone is insufficient in this case. | **Differential Diagnoses That CANNOT Be Excluded:**   1. **Epididymitis or orchitis**   Lack of urinary symptoms (dysuria, frequency) and fever makes this less likely, but atypical presentations can occur. Pain and tenderness over the epididymis/testis overlap with appendage torsion.  **Key point**: Cannot definitively exclude without urinalysis or urine culture.   2. **Testicular torsion (spermatic cord torsion)**   Cremasteric reflexes are present bilaterally, which reduces likelihood, but partial/early torsion or intermittent torsion can still occur.  **Key point**: Testicular torsion is a clinical diagnosis, and imaging (e.g., Doppler ultrasound) is often required for confirmation.   3. **Musculoskeletal strain or sprain**   Pain after physical activity (basketball) and referred pain patterns could mimic scrotal pathology. Absence of trauma does not fully exclude muscle-related pain.  **Differential Diagnoses That CAN Be Excluded:**   - **Incarcerated inguinal hernia**: No inguinal tenderness, mass, or gastrointestinal symptoms. - **Nephrolithiasis**: No flank pain, hematuria, or costovertebral angle tenderness. - **Testicular cancer**: Acute pain/swelling is atypical; cancer usually presents with a painless mass. - **Testicular fracture**: Requires significant trauma, which the patient denies.   **Conclusion:** Despite the confirmed diagnosis of testicular appendage torsion, **epididymitis/orchitis**, **testicular torsion**, and **musculoskeletal strain** cannot be fully excluded based on history and physical exam alone. Further evaluation (e.g., ultrasound) may be needed to definitively rule out spermatic cord torsion or infection. |
| Diag | **Most Appropriate Steps for Diagnosis and Treatment: ✅ Sexually transmitted disease (STD) panel ✅ Urinalysis (UA) and culture ✅ Scrotal ultrasonography**  **Rationale:**   1. **Scrotal Ultrasonography:**   **Critical first step to evaluate for testicular torsion (emergency), appendage torsion, or structural abnormalities (e.g., tumor). Doppler ultrasound assesses testicular blood flow, confirming or ruling out torsion.**  **Also identifies signs of epididymitis (e.g., epididymal enlargement, hyperemia) or orchitis.**   2. **Urinalysis (UA) and Culture:**   **Detects pyuria, bacteriuria, or leukocyte esterase, supporting epididymitis/orchitis (even in the absence of dysuria/urethral discharge).**  **Negative UA reduces suspicion for bacterial infection.**   3. **STD Panel:**   **High-risk sexual history (multiple partners, no condom use) warrants testing for chlamydia, gonorrhea, and other STIs, which are common causes of epididymitis in young men.**  **Excluded Options:**   - **❌ CBC/BMP: No fever, leukocytosis, or signs of systemic infection/renal dysfunction. Limited diagnostic value here.** - **❌ MRI of Pelvis/Scrotum: Ultrasound is the gold standard for scrotal pathology. MRI is reserved for inconclusive cases.** - **❌ Biopsy of Testis: Premature without ultrasound findings of a mass. Biopsy is not performed in acute settings unless malignancy is strongly suspected.**   **Key Considerations:**   - **Testicular torsion remains a cannot-miss diagnosis until ultrasound confirms blood flow.** - **Epididymitis requires antibiotic treatment (e.g., ceftriaxone + doxycycline) if confirmed by UA/STD panel.** - **Testicular appendage torsion is a clinical/ultrasound diagnosis; management is conservative unless pain persists.**   **Immediate Next Steps:**   1. **Scrotal ultrasound to rule out torsion.** 2. **UA/STD testing to assess for infection.** 3. **Tailor treatment based on results (e.g., antibiotics for epididymitis, surgery for torsion).** | **Most Appropriate Steps for Diagnosis and Treatment**: ✅ **Scrotal ultrasonography** ✅ **Urinalysis (UA) and culture** ✅ **Sexually transmitted disease (STD) panel**  **Explanation:**   1. **Scrotal Ultrasonography**:   **Critical to rule out testicular torsion** (a surgical emergency) and confirm testicular appendage torsion.  Doppler ultrasound evaluates blood flow (absent in torsion, preserved in appendage torsion) and identifies signs of epididymitis (e.g., hyperemia, swelling) or masses (testicular cancer).   2. **Urinalysis (UA) and Culture**:   Detects pyuria/bacteriuria, supporting **epididymitis** (even without urinary symptoms).  Urine culture identifies pathogens (e.g., *E. coli*, *Chlamydia*) to guide antibiotic therapy if indicated.   3. **STD Panel**:   Given the patient’s **high-risk sexual history** (multiple partners, unprotected sex), testing for *Chlamydia trachomatis* and *Neisseria gonorrhoeae* is essential, as these are common causes of epididymitis in young men.  **Why Other Options Are Less Appropriate:**   - **CBC/BMP**:   No systemic signs of infection (afebrile, normal vitals) or renal dysfunction (no flank pain, normal BMP unlikely to change management).   - **MRI**:   Not first-line for acute scrotal pain. Ultrasound is faster, more accessible, and equally accurate.   - **Testicular Biopsy**:   Invasive and unnecessary without imaging evidence of a mass.  **Key Points**:   - **Ultrasound is mandatory** to exclude torsion and confirm appendage torsion. - **STD testing and UA** address the possibility of infectious epididymitis, even with atypical presentation. - Immediate action (ultrasound) takes precedence over labs in this acute setting. | **Most Appropriate Steps for Diagnosis and Treatment:**   1. **Urinalysis (UA) and culture**   **Rationale**: Essential to evaluate for urinary tract infection or epididymitis/orchitis. Pyuria, bacteriuria, or positive culture would support bacterial infection and guide antibiotic therapy.   2. **Sexually transmitted disease (STD) panel**   **Rationale**: The patient’s sexual history (multiple partners, no condom use) raises concern for gonorrhea, chlamydia, or other STDs causing epididymitis/orchitis. Nucleic acid amplification tests (NAATs) or urethral swabs are indicated.   3. **Scrotal ultrasonography with Doppler**   **Rationale**: First-line imaging to evaluate for testicular torsion (absent/reduced blood flow), appendage torsion (hypervascular or avascular nodule), or infection (hyperemia in epididymitis/orchitis). Critical to rule out torsion, a surgical emergency.  **Tests to Avoid or Defer:**   - **CBC/BMP**: Not immediately necessary unless systemic illness (e.g., sepsis) or renal dysfunction is suspected. - **MRI of pelvis and scrotum**: Reserved for equivocal ultrasound findings; not first-line. - **Biopsy of testis**: Contraindicated in acute settings; biopsy is for suspected cancer (atypical painless mass), which is excluded here.   **Next Steps**:   - If ultrasound confirms **testicular torsion**, immediate surgical exploration is required. - If ultrasound supports **testicular appendage torsion**, conservative management (NSAIDs, rest) is appropriate. - Positive UA/STD testing would warrant antibiotics (e.g., ceftriaxone + doxycycline for gonorrhea/chlamydia). |
| Dx | **Most Likely Diagnosis: ✅ Epididymitis or orchitis**  **Key Supporting Evidence:**   1. **Ultrasound Findings:**   **Increased vascularity in the right epididymis/testicle: Hallmark of inflammation, consistent with epididymitis (or orchitis if the testicle is involved).**  **Reactive hydrocele: Secondary to inflammatory fluid accumulation.**  **Normal echogenicity: No evidence of mass (rules out cancer) or disrupted blood flow (rules out torsion).**   2. **Clinical Context:**   **High-risk sexual history (as noted earlier) increases likelihood of STI-associated epididymitis (e.g., chlamydia, gonorrhea).**  **Negative Gram stain and normal urinalysis do not exclude STIs, which often require specialized testing (pending STD panel).**   3. **Ruled-Out Diagnoses:**   **❌ Testicular Torsion: Doppler ultrasound confirms normal blood flow, excluding torsion.**  **❌ Testicular Appendage Torsion: Typically presents with focal tenderness at the upper testicular pole and a "blue dot sign." Normal vascularity on ultrasound makes this unlikely.**  **❌ Testicular Cancer: No masses, nodules, or abnormal echogenicity on ultrasound.**  **❌ Incarcerated Inguinal Hernia: Ultrasound would show bowel or tissue in the inguinal canal; no such findings here.**  **Why Epididymitis/Orchitis is Best Supported:**   - **Increased vascularity on Doppler ultrasound is pathognomonic for inflammatory processes (e.g., infection, STIs).** - **The presence of a reactive hydrocele further supports inflammation.** - **Empiric antibiotic therapy (e.g., ceftriaxone + doxycycline) aligns with guidelines for suspected STI-related epididymitis.**   **Immediate Action:**   - **Start empiric antibiotics while awaiting STD panel results.** - **Monitor for symptom improvement and adjust treatment based on final culture/STD findings.** | **Most Likely Diagnosis**: ✅ **Epididymitis or orchitis**  **Explanation:**   1. **Ultrasound Findings**:   **Increased vascularity in the right testicle/epididymis** → Classic sign of **inflammation** (epididymitis/orchitis).  **Reactive hydrocele** → Secondary to inflammatory fluid accumulation.  **Normal testicular blood flow** → Rules out **testicular torsion** (which would show absent/restricted flow).   2. **Clinical Context**:   Sexually active young male with high-risk history → **STDs (e.g., *Chlamydia*, *Gonorrhea*)** are the most common cause of epididymitis in this demographic.  Normal urinalysis does **not exclude epididymitis**, as infections like *Chlamydia* often lack urinary symptoms.   3. **Excluded Diagnoses**:   **Testicular torsion**: Normal Doppler flow rules this out.  **Testicular appendage torsion**: Typically presents with focal tenderness (e.g., "blue dot sign") and preserved testicular blood flow, but lacks diffuse epididymal hyperemia.  **Incarcerated inguinal hernia**: Ultrasound would show bowel in the inguinal canal, not epididymal inflammation.  **Testicular cancer**: No masses or hypoechoic lesions on ultrasound.  **Next Step**:   - Start empiric antibiotics for **STD-associated epididymitis** (e.g., ceftriaxone + doxycycline) while awaiting STD panel results. | **Most Likely Diagnosis: Epididymitis or Orchitis**  **Key Supporting Evidence**:   - **Increased vascularity on Doppler ultrasound**: This indicates inflammation, which is characteristic of infection (epididymitis/orchitis). Testicular torsion would show *reduced/absent* blood flow, whereas hyperemia points to infection. - **Reactive hydrocele**: Secondary to inflammation, consistent with infectious etiology. - **Clinical context**: Sexually active patient with risk factors for STDs (multiple partners, no condom use). - **Normal echogenicity**: Rules out structural abnormalities like testicular cancer.   **Why Other Diagnoses Are Less Likely**:   - **Testicular torsion**: Doppler findings (increased flow) and absence of sudden, severe pain contradict this diagnosis. - **Testicular appendage torsion**: No "blue dot sign" or avascular/hypervascular nodule on ultrasound. - **Incarcerated inguinal hernia**: No palpable mass, bowel symptoms, or signs of obstruction. - **Testicular cancer**: Normal echogenicity and lack of a mass on ultrasound argue against malignancy.   **Conclusion**: The combination of clinical history, ultrasound findings (hyperemia, reactive hydrocele), and exclusion of surgical emergencies (torsion) strongly supports **epididymitis or orchitis**, likely secondary to a sexually transmitted infection (pending STD panel). |
| Mang | **Most Appropriate Next Steps: ✅ Ceftriaxone 250 mg IM ✅ Doxycycline 100 mg PO twice/day for 10 days ✅ Ibuprofen 400 mg PO q 6 h as needed for pain ✅ Recommend condom usage every time he engages in sexual intercourse**  **Rationale:**   1. **Ceftriaxone + Doxycycline:**   **Empiric treatment for suspected STI-associated epididymitis (e.g., chlamydia/gonorrhea) is critical, even while awaiting STD panel results.**  **While the CDC now recommends ceftriaxone 500 mg IM for gonorrhea, the 250 mg dose in this option aligns with some guidelines for uncomplicated cases or institutional protocols.**  **Doxycycline covers chlamydia and other non-gonococcal pathogens.**   2. **Ibuprofen:**   **NSAIDs (e.g., ibuprofen) are preferred for pain and inflammation reduction in epididymitis.**   3. **Condom Usage:**   **Essential for preventing future STIs and reducing transmission risk.**  **Why Other Options Are Less Appropriate:**   - **❌ Await results of STD panel, then treat accordingly: Delaying treatment risks complications and ongoing transmission. Empiric therapy is standard.** - **❌ Acetaminophen: Less effective than NSAIDs for inflammation, though acceptable if NSAIDs are contraindicated.** - **❌ Repeat ultrasonography in 1 week: Not routinely needed unless symptoms worsen or fail to improve with treatment.**   **Clinical Priority: Initiate antibiotics immediately to address likely STI pathogens, manage pain/inflammation, and emphasize prevention. Follow up on pending STD results to confirm or adjust therapy.** | **Most Appropriate Next Steps**: ✅ **Ceftriaxone 250 mg IM** ✅ **Doxycycline 100 mg PO twice daily for 10 days** ✅ **Ibuprofen 400 mg PO q6h as needed for pain** ✅ **Recommend condom usage every time he engages in sexual intercourse**  **Rationale:**   1. **Ceftriaxone + Doxycycline**:   **Empiric treatment for STD-associated epididymitis** is critical. These cover *Neisseria gonorrhoeae* (ceftriaxone) and *Chlamydia trachomatis* (doxycycline), the most common pathogens in sexually active individuals. Delaying treatment risks complications (e.g., abscess, infertility).   2. **Ibuprofen**:   NSAIDs reduce inflammation and pain (preferred over acetaminophen, which lacks anti-inflammatory effects).   3. **Condom Usage**:   Prevents future STDs and recurrence.  **Excluded Options:**   - **Await STD panel results**: Treatment should **not** be delayed. Adjustments can be made later if needed. - **Acetaminophen**: Less optimal than NSAIDs for inflammatory pain. - **Repeat ultrasound**: Unnecessary unless symptoms worsen or fail to improve.   **Next Step**: Initiate antibiotics and NSAIDs immediately, counsel on prevention, and follow up for STD results. | **Most Appropriate Next Steps (Select all that apply):**   1. **Ceftriaxone 250 mg IM** 2. **Doxycycline 100 mg PO twice/day for 10 days** 3. **Ibuprofen 400 mg PO q6h as needed for pain** 4. **Recommend condom usage every time he engages in sexual intercourse**   **Rationale**:   - **Ceftriaxone + Doxycycline**: Empiric treatment for suspected sexually transmitted epididymitis/orchitis (covers *Chlamydia trachomatis* and *Neisseria gonorrhoeae*) while awaiting STD panel results. Delaying treatment increases complications and transmission risk. - **Ibuprofen**: First-line for pain/inflammation (NSAIDs reduce swelling better than acetaminophen). - **Condom usage**: Critical to prevent future infections, given the patient’s sexual history.   **Why Other Options Are Less Appropriate**:   - **Await results of STD panel, then treat accordingly**: Treatment should **not** be delayed in suspected STDs due to high complication/transmission risk. - **Acetaminophen**: Less effective than NSAIDs for inflammatory pain. - **Repeat ultrasonography in 1 week**: Not needed unless symptoms worsen or persist. The initial ultrasound already ruled out emergencies (e.g., torsion).   **Conclusion**: Immediate empiric antibiotics, NSAIDs, and counseling on safe sex are standard of care for suspected STD-related epididymitis. |
| Case 25 |  |  |  |
| Diff | **Conditions that CANNOT be excluded as causes of exertional dyspnea: ✅ Acute decompensated heart failure (HF) ✅ Acute myocardial infarction ✅ Constrictive pericarditis ✅ COPD exacerbation ✅ Pulmonary embolism (PE)**  **Rationale:**   1. **Acute Decompensated HF:**   **Key evidence: Worsening dyspnea, orthopnea, S3 gallop, edema, weight gain, and jugular venous distention (JVD). Classic signs of volume overload.**  **Cannot exclude: This is the most likely diagnosis given his history and exam.**   2. **Acute Myocardial Infarction (Silent MI):**   **Key evidence: History of CAD and prior MI. Elderly patients may present atypically without chest pain.**  **Cannot exclude: Absence of chest pain or EKG changes in the current presentation does not rule out ischemia, especially without recent troponin results.**   3. **Constrictive Pericarditis:**   **Key evidence: Hepatomegaly, JVD, and edema mimic right-sided HF.**  **Cannot exclude: Requires imaging (e.g., CT/MRI) to differentiate from restrictive cardiomyopathy (e.g., amyloidosis).**   4. **COPD Exacerbation:**   **Key evidence: Wheezing and cough with sputum. Overlaps with HF symptoms.**  **Cannot exclude: Bibasilar crackles favor pulmonary edema, but wheezing raises concern for COPD. Spirometry or ABG needed.**   5. **Pulmonary Embolism (PE):**   **Key evidence: Dyspnea, syncope history, and hypotension.**  **Cannot exclude: PE can present without classic features (e.g., pleuritic pain). Requires D-dimer or CT angiography to rule out.**  **Conditions that CAN be excluded:**  **❌ Cardiac Tamponade:**   - **Absent muffled heart sounds, pulsus paradoxus, or pericardial effusion on prior imaging. JVD and edema are more consistent with HF.**   **❌ Pneumonia:**   - **No fever, purulent sputum, or consolidation on prior chest x-ray. White sputum and cough are nonspecific.**   **❌ Pneumothorax:**   - **Gradual symptom onset over a week, no acute pleuritic pain, or asymmetrical lung findings.**   **❌ Atrial Fibrillation (Active Episode):**   - **Physical exam documents a regular rate and rhythm, making acute AFib less likely. However, paroxysmal AFib could contribute to chronic HF but is not the immediate trigger here.**   **Critical Considerations:**   - **Amyloidosis (suspected in prior analysis) remains a key differential for refractory HF with systemic signs (e.g., macroglossia, neuropathy).** - **Diagnostic urgency: Prioritize echocardiography, cardiac biomarkers (BNP/NT-proBNP), and imaging (CT/MRI) to differentiate between HF, constrictive pericarditis, and infiltrative diseases.** - **PE and silent MI require prompt exclusion due to mortality risk.**   **Next Steps:**   - **Echocardiogram to assess LV function, pericardium, and valvular disease.** - **Troponin, D-dimer, and BNP levels.** - **Chest CT (if PE suspected) or cardiac MRI (for amyloidosis/constrictive pericarditis).** - **Pulmonary function tests if COPD is uncertain.** | **Conditions that CANNOT be excluded as causes of exertional dyspnea**: ✅ **Acute decompensated heart failure (HF)** ✅ **Acute myocardial infarction** ✅ **Atrial fibrillation** ✅ **Cardiac tamponade** ✅ **Constrictive pericarditis** ✅ **Pulmonary embolism (PE)**  **Rationale:**   1. **Acute Decompensated HF**:   **Key evidence**: Orthopnea, JVD, S3 gallop, edema, weight gain, and crackles. Classic signs of HF exacerbation.   2. **Acute Myocardial Infarction**:   **Risk factors**: History of CAD and prior MI. Atypical presentations (e.g., dyspnea without chest pain) are common in older adults. Requires troponin/ECG to exclude.   3. **Atrial Fibrillation**:   **History**: Paroxysmal AFib. Poorly controlled AFib (e.g., rapid ventricular response) can worsen HF and cause dyspnea. Rhythm status not confirmed in current presentation.   4. **Cardiac Tamponade**:   **Possible clues**: Hypotension (BP 92/64), JVD, and dyspnea. Absence of pulsus paradoxus or muffled heart sounds does not fully exclude it without echocardiography.   5. **Constrictive Pericarditis**:   **Overlap with HF**: Symptoms like edema, JVD, and dyspnea. No history of pericarditis, but imaging (echo/CT/MRI) is required to rule out.   6. **Pulmonary Embolism**:   **Risk factors**: Sedentary lifestyle, unexplained syncope. Bilateral edema is atypical for DVT, but PE can still occur without clear signs. Requires CT angiography or D-dimer.  **Excluded or Less Likely Options:**   - **COPD Exacerbation**: No smoking history or prior COPD diagnosis. Wheezing likely due to pulmonary edema ("cardiac asthma"). - **Pneumonia**: Absence of fever, purulent sputum, or leukocytosis. White phlegm may reflect pulmonary congestion. - **Pneumothorax**: Gradual onset, no pleuritic pain, trauma, or asymmetric breath sounds.   **Next Steps:**   - **Immediate tests**: ECG, troponin, BNP, CXR, echocardiogram. - **Advanced imaging**: CT chest (for PE), cardiac MRI (if constriction/tamponade suspected). - **Monitor rhythm**: Telemetry to assess for AFib.   **Critical**: Address HF exacerbation with diuretics while pursuing diagnostics for other potential causes. | **Conditions that CANNOT be excluded as causes of exertional dyspnea:**   1. **Acute decompensated heart failure (HF)**   **Key Evidence**: History of HF, worsening dyspnea, orthopnea, weight gain, elevated JVD, S3 gallop, and bilateral edema.   2. **Acute myocardial infarction**   **Key Evidence**: CAD history and atypical MI presentations in older adults. Absence of chest pain does not rule out silent ischemia. Requires troponin/ECG to exclude.   3. **Atrial fibrillation**   **Key Evidence**: Paroxysmal AF history. AF can exacerbate HF or cause hemodynamic instability, even if not currently documented.   4. **Constrictive pericarditis**   **Key Evidence**: Overlapping signs with HF (edema, JVD). Requires echocardiography to differentiate from restrictive cardiomyopathy (e.g., amyloidosis).   5. **COPD exacerbation**   **Key Evidence**: Wheezing and cough. Cardiac asthma (HF-related wheezing) mimics COPD. Absence of smoking history does not fully exclude COPD.   6. **Pneumonia**   **Key Evidence**: Cough with sputum. Elderly patients may lack fever or leukocytosis. Requires chest imaging to exclude.   7. **Pulmonary embolism (PE)**   **Key Evidence**: Dyspnea, syncope history, and HF (a risk factor for venous stasis). PE cannot be ruled out without D-dimer/CTPA.  **Conditions that CAN BE EXCLUDED:**   - **Cardiac tamponade**: No muffled heart sounds, pulsus paradoxus, or hypotension. Elevated JVD and dyspnea are better explained by HF. - **Pneumothorax**: No sudden-onset dyspnea, pleuritic pain, or trauma. Symptoms align with chronic HF exacerbation.   **Conclusion**: The patient’s clinical picture strongly supports acute HF exacerbation, but critical mimics (MI, PE, pneumonia, etc.) require exclusion. Tamponade and pneumothorax are less likely based on history and exam. |
| Diag1 | **Most Appropriate Diagnostic Steps: ✅ Transthoracic echocardiography with Doppler ultrasonography (TTE) ✅ Cardiac MRI ✅ Electrocardiogram (ECG) ✅ Chest x-ray (CXR) ✅ Serum brain natriuretic peptide (BNP) level ✅ Serum troponin level ✅ CT scan of the chest**  **Rationale:**   1. **Transthoracic Echocardiography (TTE):**   **Critical to assess cardiac structure/function, ejection fraction, valvular disease (e.g., mitral regurgitation), and signs of infiltrative cardiomyopathy (e.g., amyloidosis: thickened walls, granular sparkling).**   2. **Cardiac MRI:**   **Gold standard for evaluating restrictive vs. constrictive physiology, myocardial infiltration (e.g., amyloidosis), and pericardial disease.**   3. **Electrocardiogram (ECG):**   **Essential to detect ischemia (e.g., STEMI/NSTEMI), arrhythmias (e.g., atrial fibrillation), or low-voltage QRS complexes (suggestive of amyloidosis).**   4. **Chest X-ray (CXR):**   **Rapidly identifies pulmonary congestion, cardiomegaly, pleural effusions, or alternative diagnoses (e.g., pneumonia).**   5. **Serum BNP:**   **Elevated in heart failure; helps differentiate cardiac vs. non-cardiac dyspnea. Supports decompensated HF but may also rise in amyloidosis.**   6. **Serum Troponin:**   **Rules out myocardial infarction, especially in a patient with CAD history. Silent MI is possible in elderly/diabetic patients.**   7. **CT Scan of the Chest:**   **Excludes pulmonary embolism (CT angiography) and evaluates lung parenchyma (e.g., emphysema, interstitial disease).**  **Less Immediate Priorities:**  **❌ Arterial Blood Gas (ABG):**   - **Not urgent unless hypoxia/hypercapnia is suspected (e.g., severe COPD or PE). Oxygen saturation and clinical context guide need.**   **❌ Pulmonary Function Testing (PFT):**   - **Useful for chronic dyspnea evaluation (e.g., COPD/asthma) but less critical here given dominant cardiac signs (orthopnea, S3 gallop, edema).**   **Key Considerations:**   - **TTE and Cardiac MRI are pivotal for distinguishing amyloidosis, constrictive pericarditis, and HF exacerbation.** - **CT Chest is necessary to exclude PE, which can mimic HF and carries high mortality if untreated.** - **BNP and Troponin are rapid, low-cost tests to confirm HF and rule out ischemia.**   **Next Steps After Testing:**   - **If amyloidosis is suspected: Serum free light chains (FLC), urine protein electrophoresis (UPEP), and tissue biopsy.** - **If constrictive pericarditis is suspected: Cardiac catheterization (to assess hemodynamics) or pericardial imaging.** - **If PE is confirmed: Anticoagulation or thrombolysis.**   **Final Note: This combination of imaging and biomarkers addresses the most critical differentials (HF, amyloidosis, PE, MI) while avoiding delays in life-saving interventions.** | **Most Appropriate Diagnostic Steps**: ✅ **Chest x-ray (CXR)** ✅ **Electrocardiogram (ECG)** ✅ **Serum brain natriuretic peptide (BNP) level** ✅ **Serum troponin level** ✅ **Transthoracic echocardiography with Doppler ultrasonography (TTE)** ✅ **CT scan of the chest** ✅ **Cardiac MRI**  **Rationale:**   1. **Chest X-ray (CXR)**:   Assess for pulmonary congestion (e.g., cephalization, pleural effusions) and cardiomegaly to support **heart failure (HF)**. Exclude pneumonia/pneumothorax.   2. **Electrocardiogram (ECG)**:   Evaluate for ischemia, arrhythmias (e.g., atrial fibrillation), or signs of prior MI. Critical given his CAD history and syncope.   3. **Serum BNP**:   Confirm HF exacerbation (elevated BNP supports volume overload). Helps differentiate cardiac vs. non-cardiac dyspnea.   4. **Serum Troponin**:   Rule out acute myocardial infarction, especially with his CAD history. Atypical MI presentations (e.g., dyspnea without pain) are common in older adults.   5. **Transthoracic Echocardiography (TTE)**:   **Priority test** to assess ejection fraction, valvular function (mitral regurgitation), ventricular wall thickness, and signs of **cardiac amyloidosis** (e.g., restrictive filling, granular sparkling).   6. **CT Scan of the Chest**:   Exclude **pulmonary embolism (PE)** (syncope and sedentary lifestyle are risk factors) and evaluate for alternative lung pathology.   7. **Cardiac MRI**:   Further evaluate for **amyloidosis** (late gadolinium enhancement, T1 mapping) or restrictive/constrictive physiology if TTE is inconclusive.  **Excluded Options:**   - **Arterial Blood Gas (ABG)**: Not urgent unless severe hypoxemia or acid-base imbalance is suspected (not described here). - **Pulmonary Function Testing (PFT)**: Unlikely to aid acutely; wheezing is more likely due to pulmonary edema ("cardiac asthma") than COPD.   **Next Steps:**   - **Immediate**: CXR, ECG, BNP, troponin, and TTE are first-line. - **If PE suspected**: Proceed to CT chest. - **If amyloidosis suspected**: Cardiac MRI and hematologic workup (e.g., serum free light chains) after TTE.   **Critical**: Prioritize TTE and BNP to confirm HF exacerbation while excluding life-threatening mimics (e.g., PE, MI). Cardiac MRI and CT chest refine the diagnosis if initial tests are equivocal. | **Most Appropriate Diagnostic Steps:**   1. **Chest X-ray (CXR)**   **Rationale**: Assess for pulmonary edema, cardiomegaly, pleural effusions, or infiltrates (e.g., pneumonia). Essential to evaluate acute decompensated HF and exclude pulmonary causes of dyspnea.   2. **Electrocardiogram (ECG)**   **Rationale**: Detect ischemia (given CAD history), arrhythmias (e.g., atrial fibrillation), or other acute changes (e.g., ST-segment deviations). Critical to rule out silent MI or arrhythmia-driven symptoms.   3. **Serum Brain Natriuretic Peptide (BNP) Level**   **Rationale**: Confirm HF exacerbation (elevated BNP supports volume overload). Helps differentiate cardiac vs. pulmonary causes of dyspnea.   4. **Serum Troponin Level**   **Rationale**: Exclude acute myocardial injury, especially in a patient with CAD. Silent MI is possible in older adults or those with neuropathy (e.g., amyloidosis).   5. **Transthoracic Echocardiography (TTE) with Doppler**   **Rationale**:  Evaluate ejection fraction, valvular function (mitral regurgitation), and ventricular wall thickness.  Assess for features of **cardiac amyloidosis** (e.g., restrictive filling, thickened myocardium, speckled appearance).  Critical for guiding HF management and identifying structural contributors.  **Less Immediate or Lower Priority:**   - **Cardiac MRI**: Valuable for infiltrative diseases (e.g., amyloidosis) but typically reserved after initial TTE. - **CT Scan of the Chest**: Consider if PE or pneumonia is strongly suspected, but CXR and clinical context (no fever, pleuritic pain) may suffice initially. - **Arterial Blood Gas (ABG)**: Not routinely needed unless hypoxia or acid-base imbalance is suspected (not highlighted in presentation). - **Pulmonary Function Testing (PFT)**: More appropriate for chronic dyspnea evaluation (e.g., COPD) rather than acute decompensation.   **Conclusion**: The priority is to confirm HF exacerbation, exclude ischemia/MI, and evaluate for amyloidosis. **CXR, ECG, BNP, troponin, and TTE** are the most critical initial tests. |
| Dx1 | **Most Likely Diagnosis: ✅ Acute decompensated heart failure**  **Rationale:**   1. **Elevated BNP (1420 pg/mL) and CXR findings (perihilar opacities, pleural effusions) strongly support volume overload and acute heart failure.** 2. **Echocardiography:**   **Restrictive diastolic dysfunction (E/A >2, reduced e’ velocities) and biatrial enlargement indicate impaired ventricular filling, leading to elevated atrial pressures and pulmonary congestion.**  **Severe concentric LV hypertrophy (without hypertension/aortic stenosis) suggests an underlying restrictive cardiomyopathy (e.g., amyloidosis), which predisposes to HFpEF exacerbation.**   3. **ECG: LBBB and 1st-degree AV block are consistent with chronic myocardial disease but do not indicate acute ischemia or infarction.** 4. **Normal troponin rules out acute MI.** 5. **No pericardial effusion/thickening excludes tamponade and constrictive pericarditis.**   **Why Not Other Options?**   - **❌ Acute MI: Normal troponin, no ECG evidence of ischemia (e.g., ST elevation/depression), and normal LV wall motion on echo.** - **❌ Cardiac tamponade: Absent pericardial effusion on imaging.** - **❌ Constrictive pericarditis: No pericardial thickening, and restrictive filling is due to myocardial disease (amyloidosis), not pericardial constriction.**   **Key Insight:**  **While the underlying etiology is likely cardiac amyloidosis (as previously noted), the acute presentation with volume overload and clinical/biomarker findings aligns with acute decompensated HF. This is the immediate diagnosis guiding urgent management (e.g., diuresis), even as further workup pursues the specific restrictive cardiomyopathy cause.**  **Next Step: Initiate diuretics for congestion while confirming amyloidosis with advanced imaging (e.g., nuclear scintigraphy) and serum/urine studies.** | **Most Likely Diagnosis**: ✅ **Acute decompensated heart failure**  **Explanation:**   1. **Acute Decompensated Heart Failure (ADHF)**:   **Supporting Evidence**:  Elevated **BNP (1420 pg/mL)** strongly indicates volume overload.  **Chest X-ray**: Perihilar opacities and pleural effusions are classic signs of pulmonary congestion.  **Echocardiogram**:.  **Normal LVEF (55%)**: Confirms **HFpEF** (heart failure with preserved ejection fraction).   2. **Excluded Diagnoses**:   **Acute MI**: Mild troponin elevation (0.03 ng/mL) is nonspecific and likely due to chronic myocardial strain, not acute ischemia. No regional wall motion abnormalities on echo.  **Cardiac Tamponade**: No pericardial effusion or tamponade physiology (e.g., right atrial collapse) on echo.  **Constrictive Pericarditis**: Absent pericardial thickening and lack of respiratory variation in mitral inflow (>15% expected in constriction vs. 5% here).  **Key Takeaway:**  The constellation of **elevated BNP, pulmonary congestion, and diastolic dysfunction** confirms acute decompensation of **HFpEF**, likely exacerbated by underlying hypertensive heart disease or cardiac amyloidosis. Other diagnoses (MI, tamponade, constriction) are inconsistent with the data. | **Most Likely Diagnosis:** **Acute Decompensated Heart Failure**  **Rationale:**   1. **BNP (1420 pg/mL)** and **CXR findings** (pleural effusions, perihilar opacities) confirm **volume overload** and HF exacerbation. 2. **Echocardiography** demonstrates **HFpEF** (LVEF 55%) with **restrictive diastolic dysfunction** and LV hypertrophy. 3. **Elevated troponin** likely reflects myocardial strain or chronic injury (e.g., amyloidosis) rather than acute MI (no ischemic ECG changes or significant troponin elevation). 4. **Cardiac tamponade** and **constrictive pericarditis** are excluded by normal pericardial thickness and absence of pericardial effusion.   **Why Not Other Options?**   - **Acute MI**: No ST-segment changes, minimal troponin elevation, and normal LV systolic function on echo. - **Cardiac Tamponade**: No pericardial effusion or hemodynamic compromise (e.g., pulsus paradoxus, hypotension). - **Constrictive Pericarditis**: Normal pericardial thickness on echo and no septal bounce/respiratory mitral inflow variation. |
| Diag2 | **Most Appropriate Steps for Guiding Diagnosis and Treatment: ✅ Cardiac catheterization**  **Rationale:**   1. **Cardiac Catheterization:**   **Coronary Angiography: To exclude occult coronary artery disease (CAD) as a contributor to HF, even with normal troponin (amyloidosis and CAD can coexist, especially in older adults).**  **Hemodynamic Assessment: Right-heart catheterization can confirm elevated filling pressures (e.g., PCWP >15 mmHg), supporting HFpEF, and differentiate restrictive vs. constrictive physiology (though constriction is already ruled out by echo).**   2. **❌ Dobutamine Stress Echocardiography:**   **Contraindicated in amyloidosis due to stiff, non-compliant ventricles (poor response to inotropic stimulation) and risk of arrhythmias (given LBBB/AV block).**   3. **❌ Exercise Stress Test:**   **High-risk in acute decompensated HF and adds little diagnostic value here (no angina symptoms, normal troponin, LBBB limits ECG interpretation).**   4. **❌ Transesophageal Echocardiogram (TEE):**   **Unnecessary unless valvular pathology (e.g., endocarditis) or intracardiac thrombus is suspected (not indicated here; TTE already showed mild MR and no thrombus).**  **Key Considerations Beyond the Options Listed:**   - **Amyloidosis-Specific Workup (if not already done):**   **Nuclear scintigraphy (99mTc-PYP/DPD) to confirm ATTR amyloidosis.**  **Serum free light chains (FLC) and urine protein electrophoresis to exclude AL amyloidosis.**  **Cardiac MRI for tissue characterization (e.g., late gadolinium enhancement, extracellular volume mapping).**  **Next Steps:**   - **Prioritize cardiac catheterization to assess for CAD and hemodynamics.** - **Concurrently pursue amyloidosis-specific testing (e.g., PYP scan, FLC) to confirm the underlying etiology.** - **Initiate diuretics for volume overload and avoid therapies that worsen conduction disease (e.g., beta-blockers).** | **Most Appropriate Steps**: ✅ **Cardiac catheterization**  **Explanation:**   1. **Cardiac Catheterization**:   **Rationale**:  Assess for **coronary artery disease (CAD)** as a contributor to LV hypertrophy and HFpEF, especially if the patient has risk factors (e.g., hypertension, diabetes).  Measure **hemodynamics** (e.g., elevated left ventricular filling pressures) to confirm HFpEF and rule out other causes (e.g., constrictive pericarditis).  **Supporting Evidence**:  Mild troponin elevation (0.03 ng/mL) raises concern for chronic myocardial strain or occult ischemia.  Severe LV hypertrophy and restrictive physiology warrant evaluation for ischemic etiology.   2. **Excluded Options**:   **Dobutamine Stress Echocardiography**: Contraindicated in **acute decompensated HF** due to risk of worsening hemodynamics.  **Exercise Stress Test**: Not safe in acute HF; unreliable with **LBBB** (ECG interpretation challenges).  **Transesophageal Echocardiogram (TEE)**: No clear indication (TTE already demonstrated structural abnormalities; no suspicion of valvular lesions, thrombi, or endocarditis).  **Key Takeaway:**  Cardiac catheterization is critical to evaluate for **underlying CAD** and confirm hemodynamic profiles in HFpEF. Other stress tests are unsafe in acute decompensation, and TEE adds no incremental value based on current findings. | **Most Appropriate Steps for Guiding Diagnosis and Treatment (Select More Than One):**  **1. Cardiac Catheterization**   - **Rationale**:   While amyloidosis is suspected, cardiac catheterization can:  Assess coronary artery disease (CAD) as a contributor to HF, especially with elevated troponin.  Measure filling pressures (e.g., pulmonary capillary wedge pressure) to confirm diastolic dysfunction and guide HF management.  **Caveat**: Risks (e.g., arrhythmia, hypotension) must be weighed in severe LV hypertrophy or hemodynamic instability.  **2. Transesophageal Echocardiogram (TEE)**   - **Rationale**:   Provides detailed imaging of atrial structures, valvular pathology, and thrombi.  May clarify the severity of mitral regurgitation or biatrial enlargement if transthoracic echo is inconclusive.  Not definitive for amyloidosis but useful in ruling out other causes (e.g., atrial masses).  **Why Other Options Are Less Appropriate:**   - **Dobutamine Stress Echocardiography**:   **Contraindicated** in restrictive physiology/LV hypertrophy due to risk of arrhythmias and poor tolerance to inotropic stress.  Does not address the underlying amyloidosis workup.   - **Exercise Stress Test**:   **Not feasible** in acute decompensated HF due to limited exercise capacity.  LBBB makes ECG interpretation unreliable for ischemia.  **Key Context:**  The **priority remains confirming amyloidosis** (via serum free light chains, bone scintigraphy, or biopsy), but **none of these are listed**. Among the provided options, **cardiac catheterization** (to exclude CAD and assess hemodynamics) and **TEE** (for structural details) are the most reasonable choices.  **Answer**: **Cardiac Catheterization** and **Transesophageal Echocardiogram (TEE)**. |
| Dx2 | **Restrictive cardiomyopathy**  **Rationale:**  **The hemodynamic and imaging findings strongly support restrictive cardiomyopathy as the etiology of acute decompensated heart failure (ADHF):**   1. **Elevated Filling Pressures:**   **LVEDP = 18 mmHg, PCWP = 17 mmHg (consistent with diastolic dysfunction).**  **RAP = 12 mmHg (elevated right-sided pressures).**   2. **Key Catheterization Findings:**   **Prominent y descent on RA waveform: Suggests rapid early diastolic filling followed by abrupt cessation (classic in restrictive physiology).**  **Concordant LV/RV respiratory pressure variation: Rules out constrictive pericarditis (which shows discordance).**   3. **Prior Findings Supporting Restriction:**   **Echocardiogram: Normal LV size, thickened walls, "granular sparkling" (amyloidosis), diastolic dysfunction, and preserved EF.**  **Nonobstructive CAD on angiography excludes ischemic cardiomyopathy.**   4. **Exclusion of Other Etiologies:**   **Dilated cardiomyopathy: Requires reduced EF and LV dilation (absent here).**  **Hypertensive cardiomyopathy: No history of severe hypertension or concentric LV hypertrophy.**  **Valvular cardiomyopathy: Only mild mitral regurgitation on prior echo (insufficient to explain ADHF).**  **Underlying Cause:**  **The most likely diagnosis is cardiac amyloidosis (ATTR or AL type), given:**   - **Clinical suspicion from prior findings (e.g., thickened walls, conduction disease).** - **Restrictive hemodynamics and elevated biomarkers (e.g., NT-proBNP).** - **Next steps should include amyloid-specific testing (e.g., PYP scan, serum free light chains).**   **Final Answer: Restrictive cardiomyopathy (likely due to cardiac amyloidosis).** | **Most Likely Etiology**: ✅ **Restrictive cardiomyopathy**  **Explanation:**   1. **Key Findings Supporting Restrictive Cardiomyopathy**:   **Hemodynamics**:  Elevated **LVEDP (18 mmHg)** and **PCWP (17 mmHg)** indicate diastolic dysfunction.  **Imaging**: Prior TTE showed **severe LV hypertrophy** and **restrictive filling pattern**, consistent with myocardial stiffness (e.g., infiltrative or hypertrophic processes).  **Exclusion of Alternatives**:  Nonobstructive CAD rules out ischemic cardiomyopathy.  o valvular lesions or systolic dysfunction (Dilated cardiomyopathy excluded).   2. **Why Not Constrictive Pericarditis?**   Though hemodynamics suggest constriction (e.g., prominent y descent), the absence of pericardial thickening on prior TTE makes **restrictive cardiomyopathy** more likely.  Constrictive pericarditis is a pericardial disease (not listed as an option), whereas restrictive cardiomyopathy is myocardial.   3. **Hypertensive vs. Restrictive Cardiomyopathy**:   Hypertensive cardiomyopathy can cause LV hypertrophy and diastolic dysfunction (HFpEF), but the **restrictive hemodynamics** (e.g., elevated RV pressures, concordant respiratory changes) and absence of severe hypertension in the clinical history favor a primary restrictive process.  **Key Takeaway:**  The hemodynamic and echocardiographic findings align with **restrictive cardiomyopathy**, likely secondary to an infiltrative disease (e.g., amyloidosis) or severe LV hypertrophy causing impaired ventricular filling. Constrictive pericarditis is not listed as an option, and other etiologies (e.g., valvular, dilated) are unsupported by the data. | **Answer**: **Restrictive Cardiomyopathy**  **Key Supporting Evidence:**   1. **Hemodynamic Findings**:   **Elevated LVEDP (18 mmHg) and PCWP (17 mmHg)**: Consistent with diastolic dysfunction, a hallmark of restrictive physiology.  **Prominent y descent on RA waveform**: Indicates rapid atrial emptying during early diastole, classic in restrictive cardiomyopathy (or constrictive pericarditis).  **Concordant LV/RV pressure tracings with respiration**: Supports restrictive cardiomyopathy over constrictive pericarditis (which typically shows discordance).   2. **Nonobstructive Coronary Artery Disease**: Excludes ischemic etiology. 3. **Prior Suspicion of Amyloidosis**: Restrictive cardiomyopathy is often caused by infiltrative diseases like amyloidosis, which aligns with earlier clinical suspicion.   **Why Other Options Are Less Likely:**   - **Dilated Cardiomyopathy**: Presents with **systolic dysfunction** (reduced LV ejection fraction), not the diastolic dysfunction seen here. - **Hypertensive Cardiomyopathy**: Typically causes LV hypertrophy and diastolic dysfunction but lacks the hemodynamic features (e.g., prominent y descent) or infiltrative pathology seen here. - **Valvular Cardiomyopathy**: Requires significant valvular disease (e.g., severe aortic stenosis, mitral regurgitation), which was not reported on prior imaging.   **Conclusion**: The combination of **diastolic dysfunction**, **elevated filling pressures**, **prominent y descent**, and **concordant respiratory pressure changes** strongly supports **restrictive cardiomyopathy** as the etiology of ADHF. Amyloidosis remains the leading underlying cause in this context. |
| Diag3 | **✅ Endomyocardial biopsy ✅ Fat pad biopsy ✅ Serum ferritin level**  **Rationale:**  **To determine the etiology of restrictive cardiomyopathy, the following steps are critical:**   1. **Endomyocardial biopsy:**   **Gold standard for confirming cardiac amyloidosis or other infiltrative diseases (e.g., sarcoidosis).**  **Directly identifies amyloid deposits in the myocardium (Congo red staining with apple-green birefringence).**   2. **Fat pad biopsy:**   **Less invasive screening for systemic amyloidosis (e.g., AL type).**  **Positive in ~70-80% of AL amyloidosis cases but less sensitive for isolated cardiac involvement.**   3. **Serum ferritin level:**   **Rules out hemochromatosis (iron overload), a reversible cause of restrictive cardiomyopathy.**  **Elevated ferritin prompts genetic testing (HFE mutation) or liver biopsy for confirmation.**  **Why Not CBC with Differential?**   - **❌ CBC with differential:**   **Not specific for diagnosing amyloidosis or other restrictive etiologies.**  **May show anemia or leukocytosis in systemic inflammation/myeloma, but not definitive for etiology.**  **Key Insight:**  **The combination of biopsies (cardiac and fat pad) addresses both cardiac-specific and systemic amyloid deposition. Serum ferritin screens for hemochromatosis, a critical reversible cause. Amyloid-specific tests (e.g., nuclear scintigraphy, serum free light chains) should also be prioritized but were not listed as options.**  **Immediate Action: Proceed with tissue biopsies and iron studies to confirm amyloidosis or identify alternative etiologies.** | **Most Appropriate Steps**: ✅ **Endomyocardial biopsy** ✅ **Fat pad biopsy** ✅ **Serum ferritin level**  **Explanation:**   1. **Endomyocardial Biopsy**:   **Gold standard** for diagnosing infiltrative etiologies (e.g., **amyloidosis**, **sarcoidosis**, **hemochromatosis**) by directly assessing myocardial tissue.  Identifies amyloid deposits, granulomas, or iron overload, which are critical for confirming specific causes of restrictive cardiomyopathy.   2. **Fat Pad Biopsy**:   Less invasive than endomyocardial biopsy and **highly specific for systemic amyloidosis**.  Subcutaneous fat aspiration can detect amyloid deposits, though sensitivity for cardiac involvement is lower (~70-80%).   3. **Serum Ferritin Level**:   Screens for **iron overload disorders** (e.g., hemochromatosis). Elevated ferritin and transferrin saturation support this diagnosis.   4. **CBC with Differential**:   Less directly relevant to determining the *specific etiology* of restrictive cardiomyopathy. While it may reveal anemia (e.g., chronic disease) or eosinophilia (e.g., hypereosinophilic syndrome), these findings are nonspecific and not diagnostic.  **Key Takeaway:**  The priority is to identify **infiltrative diseases** (amyloidosis, hemochromatosis) or **inflammatory processes** (sarcoidosis). **Endomyocardial biopsy** and **fat pad biopsy** directly target amyloidosis, while **serum ferritin** evaluates for iron overload. CBC is part of routine workup but less specific for etiology. | **Answer**:   - **Endomyocardial biopsy**   **Rationale:**   1. **Endomyocardial Biopsy**:   **Critical for amyloid subtyping**: The **fat pad biopsy** confirmed amyloidosis (Congo red staining with apple-green birefringence), but determining the subtype (e.g., **AL** vs. **ATTR**) requires **cardiac tissue**.  **Guides therapy**:  **AL amyloidosis** (caused by light-chain deposits) necessitates chemotherapy or immunotherapy.  **ATTR amyloidosis** (transthyretin-related) may be treated with TTR stabilizers (e.g., tafamidis) or gene-silencing therapies.  **Confirms cardiac involvement**: Directly demonstrates amyloid infiltration in the myocardium, which impacts prognosis and treatment urgency.   2. **Other Options**:   **CBC with differential**: Already normal, ruling out infection or hematologic disorders (e.g., eosinophilia).  **Serum ferritin**: Normal (100 ng/mL), excluding iron overload (hemochromatosis) as a cause.  **Fat pad biopsy**: Already diagnostic for amyloidosis; repeating it is unnecessary unless initial results were equivocal (which they are not).  **Key Considerations:**   - **Fat pad biopsy** is sufficient to diagnose systemic amyloidosis but cannot reliably distinguish subtypes (AL vs. ATTR) or confirm cardiac involvement. - **Endomyocardial biopsy** is the gold standard to:   Confirm amyloid deposition in the heart.  Identify the subtype via **mass spectrometry**, **immunohistochemistry**, or **genetic testing**.  **Conclusion**: **Endomyocardial biopsy** is the most appropriate next step to determine the etiology (amyloid subtype) of this patient’s restrictive cardiomyopathy. The other listed tests are either redundant or noncontributory based on prior results. |
| Dx3 | **✅ Restrictive cardiomyopathy due to cardiac amyloidosis**  **Rationale:**  **The clinical, imaging, and hemodynamic findings strongly favor cardiac amyloidosis as the etiology:**   1. **Echocardiographic Features:**   **Thickened ventricular walls with "granular sparkling" appearance (classic for amyloid infiltration).**  **Diastolic dysfunction with preserved ejection fraction (EF).**   2. **Hemodynamics:**   **Elevated LVEDP (18 mmHg) and PCWP (17 mmHg), consistent with restrictive physiology.**   3. **Prior Clues:**   **Absence of severe hypertension, valvular disease, or iron overload (hemochromatosis unlikely unless serum ferritin is elevated).**  **No history of sarcoidosis (e.g., pulmonary/lymph node involvement) or hypereosinophilia (HES unlikely).**   4. **Amyloid-Specific Findings:**   **Cardiac amyloidosis (ATTR or AL type) commonly presents with restrictive hemodynamics and conduction abnormalities.**  **Endomyocardial biopsy (Congo red staining) or nuclear scintigraphy (e.g., PYP scan) can confirm amyloid deposits.**  **Exclusion of Other Etiologies:**   - **Sarcoidosis: Requires non-caseating granulomas on biopsy; typically involves lungs/lymph nodes.** - **Hemochromatosis: Requires elevated serum ferritin/transferrin saturation; presents with iron overload (e.g., liver dysfunction, diabetes).** - **Hypereosinophilic Syndrome (HES): Requires persistent eosinophilia (>1,500/µL) and eosinophilic tissue infiltration.**   **Conclusion:**  **The granular sparkling myocardium and restrictive physiology are pathognomonic for cardiac amyloidosis. Confirmatory testing (e.g., biopsy, serum free light chains, PYP scan) should follow.**  **Final Answer: Restrictive cardiomyopathy due to cardiac amyloidosis.** | **Correct Answer**: ✅ **Restrictive cardiomyopathy due to cardiac amyloidosis**  **Key Evidence Supporting Amyloidosis:**   1. **Fat Pad Biopsy**:   **Apple-green birefringence** on Congo red staining is **pathognomonic for amyloid deposits**.  Confirms **systemic amyloidosis**, which frequently involves the heart.   2. **Normal Serum Ferritin (100 µg/L)**:   Excludes **hemochromatosis** (iron overload cardiomyopathy).   3. **Normal CBC**:   No eosinophilia, ruling out **hypereosinophilic syndrome**.  No cytopenias or leukocytosis to suggest sarcoidosis-associated inflammation.   4. **Absence of Sarcoidosis Features**:   Cardiac sarcoidosis would typically show non-caseating granulomas on biopsy (not seen here).  **Why Not Other Options?**   - **Sarcoidosis**: Requires granulomas on biopsy (absent here). - **Hemochromatosis**: Requires elevated ferritin/transferrin saturation (normal in this case). - **Hypereosinophilic Syndrome**: Requires eosinophilia on CBC (normal here).   The findings **definitively confirm amyloidosis** as the etiology. Further testing (e.g., serum free light chains, Tc-PYP scan) is needed to classify the amyloid type (AL vs. ATTR). | **Answer**:   - **Restrictive cardiomyopathy due to cardiac amyloidosis**   **Rationale:**   1. **Cardiac Amyloidosis**:   **Confirmed diagnosis**: The patient’s **fat pad biopsy** demonstrated **amyloid deposits** (Congo red staining with apple-green birefringence), which is pathognomonic for amyloidosis.  **Consistent with restrictive physiology**: Amyloid infiltration causes myocardial stiffness, leading to diastolic dysfunction and restrictive cardiomyopathy.   2. **Excluded Etiologies**:   **Cardiac sarcoidosis**: Requires evidence of non-caseating granulomas (e.g., on biopsy or imaging), not identified here.  **Hemochromatosis**: Serum ferritin is normal (100 ng/mL), ruling out iron overload.  **Hypereosinophilic syndrome**: CBC with differential is normal, excluding eosinophilia.   3. **Key Supporting Evidence**:   **Amyloidosis** is the **only etiology confirmed by prior testing** (fat pad biopsy). Subtyping (e.g., AL vs. ATTR) would require endomyocardial biopsy, but the underlying etiology is already established as amyloidosis.  **Conclusion**: **Restrictive cardiomyopathy due to cardiac amyloidosis** is the most likely etiology, based on the diagnostic confirmation of amyloid deposits. Other options are inconsistent with the patient’s laboratory and biopsy findings. |
| Mang | **✅ Admit to a monitored telemetry inpatient unit for further treatment ✅ 2-g sodium restriction ✅ 1-L fluid restriction ✅ Furosemide 40 mg IV bolus followed by 10 mg/hour furosemide infusion ✅ Amyloid typing**  **Rationale:**   1. **Admit to telemetry:**   **Restrictive cardiomyopathy (especially amyloidosis) carries a high risk of arrhythmias (e.g., atrial fibrillation, heart block) and sudden cardiac death. Continuous monitoring is critical.**   2. **Sodium/fluid restriction:**   **Reduces fluid overload and preload, improving symptoms of congestion (e.g., dyspnea, edema).**   3. **IV furosemide:**   **Aggressive diuresis is needed to manage volume overload in acute decompensation. IV administration ensures rapid and reliable effect.**   4. **Amyloid typing:**   **Essential to distinguish AL (plasma cell-derived) from ATTR (transthyretin) amyloidosis, as treatment differs radically:**  **AL: Requires antiplasma cell therapy (e.g., chemotherapy, daratumumab).**  **ATTR: Managed with TTR stabilizers (tafamidis, diflunisal) or gene-silencing agents (patisiran, inotersen).**  **Why Not Other Options?:**   - **Antiplasma cell therapy: Requires confirmation of AL amyloidosis via typing (e.g., serum free light chains, biopsy). Premature without amyloid characterization.** - **Digoxin: Avoid in amyloidosis due to binding to amyloid fibrils, increasing toxicity risk.** - **Dobutamine: Ineffective in restrictive physiology (diastolic dysfunction) and may worsen arrhythmias.** - **Lisinopril/Metoprolol:**   **ACE inhibitors (lisinopril) often poorly tolerated due to hypotension and renal dysfunction in amyloidosis.**  **Beta-blockers (metoprolol) may worsen bradycardia/heart block but can be cautiously used for rate control in atrial fibrillation.**   - **Discharge home: Unsafe given the risk of rapid clinical deterioration.**   **Key Actions:**   1. **Initiate diuresis and volume management.** 2. **Confirm amyloid subtype (AL vs. ATTR) via typing (e.g., serum/urine immunofixation, bone marrow biopsy, or scintigraphy).** 3. **Avoid nephrotoxic drugs and negative inotropes (e.g., beta-blockers, calcium channel blockers) unless necessary for arrhythmias.** 4. **Plan for definitive therapy (e.g., chemotherapy for AL, tafamidis for ATTR) once etiology is confirmed.**   **Final Answer: Admission, diuresis, sodium/fluid restriction, and amyloid typing are immediate priorities.** | **orrect Answers**: ✅ **Admit to a monitored telemetry inpatient unit for further treatment** ✅ **2-g sodium restriction** ✅ **1-L fluid restriction** ✅ **Furosemide 40 mg IV bolus followed by 10 mg/hour furosemide infusion** ✅ **Amyloid typing**  **Rationale:**   1. **Admit to telemetry**:   Cardiac amyloidosis carries risks of arrhythmias, conduction abnormalities, and acute decompensation. Inpatient monitoring is critical for stabilization and further evaluation.   2. **Sodium/fluid restriction**:   Standard in heart failure management to reduce volume overload, especially in restrictive physiology.   3. **IV furosemide**:   Aggressive diuresis is often needed in amyloidosis due to restrictive filling and fluid retention. IV administration is preferred in acute settings for rapid effect.   4. **Amyloid typing**:   Essential to determine subtype (AL vs. ATTR) for targeted therapy (e.g., antiplasma cell therapy for AL, tafamidis for ATTR).  **Excluded Options:**   - **Discharge home**: Inappropriate for a newly diagnosed systemic disease with cardiac involvement and potential instability. - **Antiplasma cell therapy**: Requires confirmation of AL subtype via amyloid typing. - **Digoxin**: Contraindicated due to increased toxicity risk in amyloidosis. - **Dobutamine**: Not indicated unless cardiogenic shock is present (not described here). - **ACE inhibitors (lisinopril)/beta-blockers (metoprolol)**: Use cautiously in amyloidosis due to hypotension/conduction risks, but not first-line in acute management. - **Oral furosemide**: May be used later for chronic management but less effective than IV in acute volume overload.   **Next Steps**:   - Complete amyloid typing (e.g., serum free light chains, Tc-PYP scan). - Initiate subtype-specific therapy once confirmed (e.g., chemotherapy for AL, tafamidis for ATTR). - Monitor for arrhythmias and adjust diuretics based on response. | **Answer**:   - **Admit to a monitored telemetry inpatient unit for further treatment** - **2-g sodium restriction** - **1-L fluid restriction** - **Furosemide 40 mg IV bolus followed by 10 mg/hour furosemide infusion** - **Amyloid typing**   **Rationale:**   1. **Admit to a monitored telemetry unit**:   Cardiac amyloidosis carries a risk of arrhythmias and hemodynamic instability. Admission allows for continuous monitoring, further diagnostic evaluation (e.g., endomyocardial biopsy), and initiation of tailored therapy.   2. **Sodium (2-g) and fluid (1-L) restrictions**:   Standard measures to reduce volume overload and manage congestive symptoms in restrictive cardiomyopathy.   3. **Furosemide IV bolus and infusion**:   Effective for acute decongestion in patients with fluid overload. IV administration ensures rapid diuresis, particularly important in restrictive physiology where preload optimization is critical.   4. **Amyloid typing**:   Essential to determine the amyloid subtype (e.g., AL vs. ATTR) via **endomyocardial biopsy**, as this directly guides therapy (e.g., chemotherapy for AL amyloidosis vs. TTR stabilizers for ATTR).  **Excluded Options:**   - **Discharge home**: Inappropriate given the need for urgent amyloid typing, potential for rapid clinical deterioration, and initiation of disease-specific therapy. - **Antiplasma cell therapy**: Requires confirmation of AL amyloidosis via typing, which has not yet been performed. - **Digoxin**: Contraindicated in amyloidosis due to increased risk of toxicity from binding to amyloid fibrils. - **Dobutamine**: Inotropes are generally ineffective in restrictive cardiomyopathy and may exacerbate arrhythmias. - **ACE inhibitors (lisinopril)/Beta-blockers (metoprolol)**: Poorly tolerated in amyloidosis due to hypotension and reduced cardiac output.   **Conclusion**: Immediate priorities include hospitalization for monitoring, fluid/sodium restriction, diuresis, and definitive amyloid typing to guide subsequent therapy. |
| Case 26 |  |  |  |
| Diff | **Differential Diagnoses That Cannot Be Excluded: ✅ Acute coronary syndrome/STEMI ✅ Aortic dissection ✅ Dysrhythmia ✅ Hypoglycemia ✅ Pericardial effusion with cardiac tamponade ✅ Pulmonary embolism (PE) ✅ Septic shock ✅ Stroke/TIA ✅ Tension pneumothorax**  **Rationale:**   1. **Acute Coronary Syndrome/STEMI:**   **Midsternal chest pain, hypotension, and recent cardiac procedure (TAVR) raise concern for ischemia. ECG and troponin are needed to exclude.**   2. **Aortic Dissection:**   **Post-TAVR vascular injury risk. Chest pain and shock are red flags. Requires CT angiography or transesophageal echo.**   3. **Dysrhythmia:**   **Tachycardia and syncope post-TAVR (conduction abnormalities are common). Telemetry/ECG needed to rule out VT, heart block, or atrial arrhythmias.**   4. **Hypoglycemia:**   **Altered mental status in a diabetic or elderly patient. Requires bedside glucose testing (not mentioned in history/physical).**   5. **Pericardial Effusion/Tamponade:**   **Beck’s triad (hypotension, JVD, muffled heart sounds) is classic. Post-TAVR tamponade is a critical complication. Immediate echo is diagnostic.**   6. **Pulmonary Embolism (PE):**   **Tachycardia, hypoxia (cyanosis), and post-procedural immobility. CT pulmonary angiography or V/Q scan needed.**   7. **Septic Shock:**   **Hypothermia, hypotension, and recent hospitalization/injections. Blood cultures and lactate are required to exclude.**   8. **Stroke/TIA:**   **Confusion and syncope in a patient with dementia. Brain imaging (CT/MRI) is necessary to exclude acute cerebrovascular event.**   9. **Tension Pneumothorax:**   **Respiratory distress and hypotension. Though lungs are clear, subtle pneumothorax may not manifest with absent breath sounds. CXR or ultrasound needed.**  **Excluded Diagnoses:**   - **Dehydration: JVD and clear lungs suggest volume overload, not hypovolemia.** - **Neurogenic Shock: Typically involves bradycardia (not tachycardia) and spinal trauma history (absent here).** - **Vasovagal Syncope: Transient hypotension resolving quickly; inconsistent with persistent shock and critical vital signs.**   **Key Next Steps:**   1. **Bedside echocardiogram to evaluate for tamponade, valve function, and ventricular performance.** 2. **ECG to assess for ischemia or arrhythmia.** 3. **CT angiography (aorta, pulmonary arteries) and brain imaging.** 4. **Blood tests: Glucose, lactate, troponin, cultures.** 5. **Empirical treatment for sepsis and tamponade while awaiting results.**   **Final Answer: The listed diagnoses above cannot be excluded without further testing.** | **Correct Answers**: ✅ **Acute coronary syndrome/ST segment elevation myocardial infarction (STEMI)** ✅ **Aortic dissection** ✅ **Dysrhythmia** ✅ **Pericardial effusion with cardiac tamponade** ✅ **Pulmonary embolism (PE)** ✅ **Septic shock** ✅ **Hypoglycemia** ✅ **Stroke/transient ischemic attack (TIA)**  **Rationale:**  **Cannot be excluded:**   1. **Acute coronary syndrome/STEMI**:   Midsternal chest pain, hypotension, and recent TAVR (risk for coronary obstruction). ECG and troponin not reported; STEMI cannot be ruled out.   2. **Aortic dissection**:   Recent aortic valve procedure (TAVR) increases risk. Chest pain and hypotension are consistent, though classic "tearing" pain or pulse deficits not described. Imaging (e.g., CTA) required to exclude.   3. **Dysrhythmia**:   Tachycardia (115 bpm) and syncope suggest arrhythmia (e.g., VT, AF with RVR). Telemetry monitoring needed.   4. **Pericardial effusion with tamponade**:   **Beck’s triad** (hypotension, JVD, muffled heart sounds) is present. Recent TAVR is a key risk factor for tamponade.   5. **Pulmonary embolism (PE)**:   Dyspnea, syncope, and post-procedural immobility. PE is a common cause of shock post-surgery.   6. **Septic shock**:   Hypothermia (34.6°C), confusion, and recent procedure (groin incision). Elderly patients may lack fever; blood cultures and lactate required.   7. **Hypoglycemia**:   Confusion could indicate low glucose. No documented fingerstick glucose; cannot exclude without testing.   8. **Stroke/TIA**:   New confusion and syncope. Though focal deficits are absent, posterior circulation strokes or global hypoperfusion (e.g., from shock) may explain symptoms.  **Excluded Diagnoses:**   - **Dehydration**: JVD and clear lungs argue against hypovolemia. - **Neurogenic shock**: Requires bradycardia (not present) and spinal injury (no trauma history). - **Tension pneumothorax**: Clear lung fields and absence of trauma/respiratory asymmetry exclude. - **Vasovagal syncope**: Persistent hypotension/tachycardia and lack of prodrome (e.g., nausea) make this unlikely.   **Next Steps:**   1. **Emergent echocardiogram** to evaluate for tamponade, ventricular function, and TAVR complications. 2. **ECG and troponin** to assess for ACS. 3. **CT angiography** (chest/abdomen) to rule out PE/aortic dissection. 4. **Blood glucose**, lactate, cultures, and imaging (e.g., head CT) for stroke/sepsis evaluation. 5. **Continuous telemetry** for dysrhythmia detection.   **Immediate priorities**: Stabilize shock, identify reversible causes (e.g., tamponade, PE), and prevent organ failure. | **Answer**:   - **Acute coronary syndrome/ST segment elevation myocardial infarction (STEMI)** - **Aortic dissection** - **Dysrhythmia** - **Pericardial effusion with cardiac tamponade** - **Pulmonary embolism (PE)** - **Septic shock**   **Rationale:**   1. **Acute Coronary Syndrome/STEMI**:   **Chest pain** (constant, midsternal) and recent **TAVR** (risk of coronary obstruction) raise concern for ACS. ECG and troponin are needed to exclude.   2. **Aortic Dissection**:   Recent aortic manipulation (TAVR) and **hypotension/chest pain** warrant exclusion. Imaging (CT aorta or TEE) is required despite absence of classic "tearing" pain or pulse deficits.   3. **Dysrhythmia**:   **Syncope** and **tachycardia** suggest possible transient arrhythmia (e.g., ventricular tachycardia or heart block) post-TAVR. Telemetry monitoring is critical.   4. **Pericardial Effusion with Tamponade**:   **Beck’s triad** (hypotension, JVD, muffled heart sounds) in a post-TAVR patient strongly suggests tamponade. Bedside echocardiography is urgent.   5. **Pulmonary Embolism (PE)**:   **Dyspnea**, **syncope**, and **post-procedural immobility** are classic PE risk factors. Clear lungs do not exclude PE; CT pulmonary angiography or V/Q scan is needed.   6. **Septic Shock**:   **Hypothermia**, **confusion**, and recent procedure (TAVR) raise suspicion for sepsis. Blood cultures and empiric antibiotics are indicated despite no obvious infection source.  **Excluded Diagnoses:**   - **Dehydration**: Contradicted by **jugular venous distention** (JVD), which suggests volume overload or obstructive shock. - **Hypoglycemia**: Unlikely without diabetes history, but point-of-care glucose testing should still be performed. - **Neurogenic Shock**: No spinal trauma or bradycardia (patient is tachycardic). - **Stroke/TIA**: No focal neurologic deficits (e.g., asymmetric weakness, gaze deviation). - **Tension Pneumothorax**: Clear lung fields and absence of trauma make this unlikely. - **Vasovagal Syncope**: Inconsistent with **persistent hypotension/tachycardia** and lack of typical prodrome.   **Key Next Steps:**   1. **Bedside echocardiogram** to assess for tamponade, valve function, and myocardial contractility. 2. **ECG and troponin** to evaluate for ACS. 3. **CT angiography** (chest/abdomen/pelvis) to rule out aortic dissection and PE. 4. **Blood cultures and lactate** to assess for sepsis. 5. **Continuous telemetry** to detect arrhythmias.   **Conclusion**: These diagnoses remain on the differential until imaging, labs, and monitoring provide definitive exclusion. Immediate intervention (e.g., pericardiocentesis for tamponade, antibiotics for sepsis) may be required based on findings. |
| Mang1 | **Essential Immediate Steps: ✅ Bedside glucose measurement ✅ BP measurement every 5 min ✅ Pulse oximetry ✅ Cardiac monitoring ✅ Oxygen 15 L by nonrebreather mask**  **Rationale:**   1. **Bedside glucose measurement:**   **Critical to exclude hypoglycemia as a cause of altered mental status, even without a history of diabetes. Rapid and actionable.**   2. **BP measurement every 5 min:**   **Severe hypotension (70/21 mmHg) requires continuous monitoring to assess response to resuscitation (e.g., fluids, inotropes).**   3. **Pulse oximetry:**   **Cyanosis and dyspnea indicate hypoxia. Oxygen saturation guides oxygen therapy and identifies respiratory failure.**   4. **Cardiac monitoring:**   **Tachycardia (115 bpm) and post-TAVR status increase risk for dysrhythmias (e.g., VT, heart block). Continuous ECG detects life-threatening rhythms.**   5. **Oxygen 15 L by nonrebreather mask:**   **Cyanosis, tachypnea (34/min), and hypoxia require high-flow oxygen to improve tissue perfusion and prevent organ failure.**  **Excluded Steps:**   - **0.9% saline IV bolus:**   **Contraindicated here due to signs of obstructive shock (JVD, muffled heart sounds) suggesting tamponade. Fluids may worsen hemodynamics.**   - **Dextrose 50 g IV:**   **Only if hypoglycemia is confirmed (requires glucose result first). Blind administration risks hyperglycemia.**   - **Naloxone:**   **No evidence of opioid exposure (respiratory rate is elevated, not depressed).**   - **Toxicology screening:**   **Low suspicion for overdose given history and presentation. Not immediately lifesaving.**  **Key Considerations:**   - **Pericardial tamponade (post-TAVR complication) is the top priority. Immediate echocardiogram is needed.** - **Avoid fluid resuscitation until tamponade is ruled out (fluids increase RV/LV compression).** - **Empirical antibiotics and blood cultures should be considered for possible sepsis (hypothermia, recent procedure).**   **Final Answer: Bedside glucose, BP monitoring, pulse oximetry, cardiac monitoring, and high-flow oxygen are essential. Fluids and dextrose are deferred pending further assessment.** | **Essential Immediate Steps**: ✅ **BP measurement every 5 min** ✅ **Pulse oximetry** ✅ **Cardiac monitoring** ✅ **Oxygen 15 L by nonrebreather mask** ✅ **0.9% saline solution, 1 L IV bolus** ✅ **Dextrose 50 g IV**  **Rationale:**   1. **BP Measurement Every 5 Minutes**:   Critical to monitor response to fluid resuscitation and detect worsening hypotension. Current BP (80/35 mmHg) remains unstable, requiring frequent reassessment.   2. **Pulse Oximetry**:   Continuous monitoring is essential to ensure oxygenation (improved to 92% on O₂) and guide further respiratory support.   3. **Cardiac Monitoring**:   Required to detect dysrhythmias (e.g., VT, AF) or ischemia, given the patient’s tachycardia (115 bpm) and risk of ACS.   4. **Oxygen 15 L via Nonrebreather Mask**:   Maintains oxygenation in the setting of hypoxemia (SpO₂ 85% on room air) and reduces tissue hypoxia.   5. **0.9% Saline IV Bolus**:   Despite partial BP improvement after 1 L, persistent hypotension (80/35 mmHg) suggests ongoing fluid responsiveness, especially in septic or hypovolemic shock. Additional fluids are warranted unless contraindicated (e.g., overt cardiac failure).   6. **Dextrose 50 g IV**:   Addresses hypoglycemia (72 mg/dL), which can contribute to altered mental status or hemodynamic instability in elderly patients.  **Excluded Options:**   - **Bedside Glucose Measurement**:   Already confirmed (72 mg/dL). Recheck after dextrose administration is appropriate but not an *immediate* priority.   - **Toxicology Screening/Naloxone**:   No clinical clues (e.g., opioid toxidrome, drug exposure) to justify urgency.   - **Echocardiography/CT Scans**:   While critical for diagnosis, these require stabilization first (e.g., securing ABCs, addressing hypoglycemia/hypotension).  **Key Considerations:**   - **Fluid Balance**: Proceed cautiously with additional fluids if cardiac tamponade or cardiogenic shock is suspected, but initial improvement with 1 L supports continued judicious use. - **Hypoglycemia**: Treat first, investigate later—even mild hypoglycemia can exacerbate confusion or shock in older adults. - **Prioritize ABCs**: Stabilize oxygenation, circulation, and glucose before advanced imaging or diagnostics.   **Next**: Proceed to ECG, bedside echocardiogram, and CT imaging **after** immediate interventions above. | **Answer**:   - **Bedside glucose measurement** - **BP measurement every 5 min** - **Pulse oximetry** - **Cardiac monitoring** - **Oxygen 15 L by nonrebreather mask**   **Rationale:**   1. **Bedside glucose measurement**:   Critical to rule out hypoglycemia as a cause of altered mental status and syncope, even in patients without diabetes.   2. **BP measurement every 5 min**:   The patient is in **profound shock** (BP 70/21 mmHg). Continuous monitoring is essential to assess response to interventions and guide therapy.   3. **Pulse oximetry**:   Required to evaluate oxygenation in a patient with **dyspnea and respiratory distress**. Hypoxia must be addressed immediately.   4. **Cardiac monitoring**:   Tachycardia and post-TAVR status increase the risk of **dysrhythmias** (e.g., ventricular tachycardia, heart block). Continuous monitoring is necessary.   5. **Oxygen 15 L by nonrebreather mask**:   Empiric oxygen is warranted for respiratory distress and hypoxia (even if not yet confirmed), especially in shock. Clear lung fields reduce concern for hypercapnia in COPD.  **Excluded Options:**   - **0.9% saline solution, 1 L IV bolus**:   **Jugular venous distention (JVD)** and muffled heart sounds suggest **cardiac tamponade** (obstructive shock), where fluid resuscitation may worsen hemodynamics. Fluids should be withheld until echocardiography confirms the etiology.   - **Dextrose 50 g IV**:   Should only be given **after confirming hypoglycemia** via bedside glucose. Empiric dextrose risks hyperglycemia if not indicated.   - **Naloxone 0.4 mg IV**:   No clinical suspicion of opioid overdose (no respiratory depression, pinpoint pupils, or opioid use history).   - **Toxicology screening**:   Low suspicion for overdose/toxidrome given the clinical context (recent TAVR, no substance use history).  **Key Considerations:**   - **Pericardial tamponade** is a leading concern post-TAVR. **Fluid resuscitation is contraindicated** until tamponade is ruled out via echocardiography. - **Hypotension management** depends on the shock etiology (e.g., inotropes for cardiogenic shock, pericardiocentesis for tamponade). - **Empiric oxygen** improves oxygen delivery in shock, even if hypoxia is not yet confirmed.   **Conclusion**: Prioritize rapid diagnostics (e.g., echocardiogram, ECG) while stabilizing the patient with oxygen, monitoring, and glucose assessment. Avoid fluids until tamponade is excluded. |
| Diag | **Most Appropriate Steps for Diagnosis & Treatment:**  **✅ Echocardiography at Bedside**   - **Priority: Rapidly evaluate for pericardial effusion/tamponade (critical post-TAVR complication) and assess cardiac function (e.g., right ventricular strain from PE, LV dysfunction).** - **Action: If tamponade is confirmed → immediate pericardiocentesis.**   **✅ ECG**   - **Purpose: Detect arrhythmias (e.g., AF, heart block), ischemia (e.g., STEMI), or signs of PE (e.g., S1Q3T3, right-axis deviation).** - **Post-TAVR Risks: New conduction abnormalities or ischemia require urgent intervention.**   **✅ CT Scan of the Chest (CTPA)**   - **Indication: Evaluate for pulmonary embolism (hypoxia, tachycardia, post-procedural immobility) or aortic dissection.** - **Caveat: Proceed if patient is stable enough for transport. If unstable, prioritize bedside tests first.**   **❌ Echocardiography in a Radiology Suite**   - **Unnecessary delay. Bedside echo provides immediate answers in critical settings.**   **❌ Chest X-ray at Bedside**   - **Lower yield unless suspecting pneumothorax or consolidation. Hypoxia and hypotension are better explained by tamponade, PE, or sepsis.**   **❌ CT Scan of the Head**   - **Only if focal neurologic deficits or persistent AMS despite improved oxygenation/BP. Not priority here.**   **Rationale:**   1. **Bedside Echo First: Tamponade is the most immediately life-threatening and treatable diagnosis post-TAVR.** 2. **ECG: Quick, non-invasive, and rules out arrhythmias/ischemia.** 3. **CTPA: Gold standard for PE, which aligns with hypoxia and tachycardia.**   **Next Steps After Results:**   - **If tamponade: Pericardiocentesis.** - **If PE: Anticoagulation or thrombolysis.** - **If septic shock: Continue antibiotics, vasopressors.**   **Final Answer: ECG, bedside echocardiography, and CT scan of the chest (CTPA) are most appropriate.** | **Most Appropriate Steps for Diagnosis and Treatment**: ✅ **ECG** ✅ **Echocardiography at bedside** ✅ **CT scan of the chest** ✅ **CT scan of the head**  **Rationale:**   1. **ECG**:   **Urgent priority** to evaluate for STEMI (ST elevation, new LBBB) or dysrhythmias (e.g., VT, AF). Critical for ruling out ACS or arrhythmia as causes of syncope/hypotension.   2. **Bedside Echocardiography**:   **Immediate and safe** in an unstable patient. Assess for:  **Cardiac tamponade** (RA/RV collapse, diastolic dysfunction).  **LV/RV dysfunction** (ACS, PE).  **TAVR complications** (valve malposition, aortic root injury).  Avoids transporting an unstable patient to a radiology suite.   3. **CT Scan of the Chest**:   Required to rule out **aortic dissection** (suspected with chest pain and recent TAVR) and **pulmonary embolism** (hypoxemia, post-procedural immobility).  **Caveat**: Patient must be stable enough for transport. If BP remains critically low (80/35 mmHg), consider stabilizing first (e.g., vasopressors) or prioritize bedside tests.   4. **CT Scan of the Head**:   Evaluate for **stroke/TIA** (confusion, syncope) or intracranial hemorrhage (if anticoagulants were used post-TAVR).  Hypoxemia and shock may mimic stroke, but imaging is still necessary to exclude structural causes.  **Lower Priority or Less Critical:**   - **Chest X-ray at Bedside**:   Less urgent given clear lung fields on prior exam. May help assess for pneumothorax, pulmonary edema, or mediastinal widening (dissection), but **CT chest is more definitive**.   - **Echocardiography in Radiology Suite**:   Unnecessary risk in an unstable patient; **bedside echo is faster and safer**.  **Next Steps:**   1. **Immediate**:   **ECG** and **bedside echo** to guide resuscitation (e.g., pericardiocentesis if tamponade, inotropes for cardiogenic shock).   2. **If Stabilized**:   **CT chest** (PE/dissection) and **CT head** (stroke) to confirm/exclude critical diagnoses.   3. **Labs**:   Repeat glucose, troponin, lactate, blood cultures, and D-dimer (if PE suspected).  **Key Considerations**:   - Balance urgency of imaging with patient stability. If BP remains 80/35 mmHg despite fluids, initiate vasopressors (e.g., norepinephrine) and proceed with time-sensitive tests (ECG, echo) before advanced imaging. - **Hypoglycemia (72 mg/dL)**: Recheck glucose after treatment (e.g., IV dextrose) to ensure normalization.   **Final Priority**: Identify and treat reversible causes of shock (e.g., tamponade, PE, sepsis) while stabilizing hemodynamics. | **Most Appropriate Steps for Diagnosis and Treatment:**   1. **ECG**:   **Priority**: Immediate.  **Rationale**: Assess for signs of right heart strain (e.g., S1Q3T3 pattern, right axis deviation, T-wave inversions in V1-V4), arrhythmias, or ischemia. ECG is critical if pulmonary embolism (PE) or pericardial effusion/tamponade is suspected.   2. **Echocardiography at Bedside**:   **Priority**: **Highest urgency**.  **Rationale**:  Confirm or rule out **cardiac tamponade** (e.g., diastolic right atrial/RV collapse, pericardial effusion).  Evaluate for signs of **PE** (e.g., right ventricular dilation, McConnell’s sign).  Assess left ventricular function to exclude cardiogenic shock.  Bedside echo avoids transporting an unstable patient and provides real-time guidance for interventions (e.g., pericardiocentesis).   3. **Chest X-ray at Bedside**:   **Priority**: Secondary (if echo is delayed or unavailable).  **Rationale**:  Rule out pneumothorax, pulmonary edema, or consolidations (e.g., pneumonia).  May show an enlarged cardiac silhouette in tamponade or oligemic lung fields in massive PE.  **Less Appropriate/Contraindicated Steps:**   - **Echocardiography in a Radiology Suite**:   **Risk**: Transporting an unstable patient (BP 80/35 mmHg) is unsafe. Bedside echo is preferred.   - **CT Scan of the Chest**:   **Risk**: Requires transport and contrast administration in an unstable patient. Reserve for **stable patients** if PE remains suspected after bedside echo.  **Exception**: Consider if bedside echo is inconclusive and thrombolytics for PE are being contemplated.   - **CT Scan of the Head**:   **Not Indicated**: No clinical evidence of neurologic deficits, trauma, or altered mental status attributable to intracranial pathology.  **Clinical Reasoning:**   - The patient’s **obstructive shock physiology** (JVD, minimal fluid responsiveness, hypoxia) makes **bedside echocardiography** the **most critical test** to diagnose tamponade or PE. - **ECG** is a rapid adjunct to identify arrhythmias or right heart strain. - **Chest X-ray** is lower yield but can exclude concurrent pathologies (e.g., pneumothorax).   **Next Steps**:   - If tamponade is confirmed on echo → **emergent pericardiocentesis**. - If PE is suspected → **CT pulmonary angiography** (if stable) or empiric thrombolytics (if unstable). - If sepsis is suspected → **blood cultures and antibiotics**.   **Avoid Delays**: Prioritize bedside assessments to prevent clinical deterioration. |
| Dx | **Most Likely Diagnosis:**  **✅ Cardiac Tamponade**  **Rationale:**   1. **Key Findings:**   **Pericardial effusion confirmed on echocardiography (critical post-TAVR complication).**  **Hypotension (MAP < 65 mmHg) and hypoxia (SpO₂ 92% on O₂) → Signs of hemodynamic compromise.**  **Sinus tachycardia on ECG → Compensatory response to reduced cardiac output.**   2. **Why Tamponade Over Other Options:**   **Acute Coronary Syndrome (ACS): Unlikely. ECG shows no ST-T changes, and troponin results were not elevated (if available).**  **Dysrhythmia: Sinus tachycardia is a secondary response, not a primary arrhythmia.**  **Pulmonary Embolism (PE): Possible (hypoxia/tachycardia), but pericardial effusion with hemodynamic instability is more consistent with tamponade. PE would not explain the effusion.**   3. **Post-TAVR Context:**   **Pericardial effusion is a well-recognized complication of TAVR, often due to guidewire/pericardial trauma.**  **Tamponade can develop even without classic ECG findings (e.g., electrical alternans is absent in ~50% of cases).**  **Next Step:**   - **Emergent pericardiocentesis if echocardiography shows signs of tamponade (e.g., right atrial/ventricular collapse, IVC plethora).**   **Why Not Other Options:**   - **ACS: No ischemic ECG changes or elevated troponin.** - **Dysrhythmia: No arrhythmia beyond compensatory sinus tachycardia.** - **PE: Requires CTPA confirmation, but tamponade is the immediate life-threatening diagnosis to address first.**   **Final Answer: Cardiac Tamponade.** | **Answer:** ✅ **Cardiac Tamponade**  **Rationale:**   1. **Key Findings Supporting Tamponade**:   **Echocardiogram**: Confirmed pericardial effusion.  **Hypotension (80/35 mmHg)** and **tachycardia (115 bpm)**: Consistent with impaired cardiac output due to pericardial fluid compressing the heart.  **Chest X-ray**: Cardiac silhouette enlargement (common in pericardial effusion).  **Transient BP improvement with fluids**: Suggests preload dependence, a hallmark of tamponade physiology.   2. **Why Not Other Diagnoses?**:   **Acute Coronary Syndrome (ACS)**: No ECG changes (ST-T abnormalities) or chest pain reported.  **Dysrhythmia**: Sinus tachycardia is secondary to hypotension/hypovolemia, not a primary arrhythmia.  **Pulmonary Embolism (PE)**: Hypoxemia improved with oxygen (uncommon in massive PE). No ECG signs of right heart strain (e.g., S1Q3T3) or imaging evidence of PE (e.g., CT findings).   3. **Missing Classic Signs?**:   Absence of **jugular venous distension (JVD)** or **pulsus paradoxus** may reflect early tamponade or compensated physiology. Electrical alternans is absent on ECG but is only present in ~20% of cases.  **Next Steps:**   - **Emergent pericardiocentesis** if hemodynamics worsen (e.g., BP drops further, pulsus paradoxus develops). - **Repeat echocardiogram** to assess for tamponade physiology (e.g., right atrial/ventricular collapse, diastolic RV inversion). - **Investigate underlying cause** (e.g., infection, malignancy, autoimmune disease).   **Critical Action**: Monitor closely for progression to overt cardiac tamponade requiring immediate intervention. | **Answer:** **Cardiac Tamponade**  **Rationale:**   1. **Clinical and Echocardiographic Evidence**:   Hypotension (BP 80/35 mmHg), JVD, and pericardial effusion on echo form the **classic triad of cardiac tamponade** (Beck’s triad).  Echocardiography is the gold standard for diagnosing tamponade; diastolic collapse of the right atrium/ventricle or IVC plethora would confirm hemodynamic compromise.   2. **Exclusion of Other Options**:   **Acute Coronary Syndrome (ACS)**: Unlikely due to **no ECG changes** (normal ST-T segments, no Q-waves) and absence of chest pain.  **Dysrhythmia**: Sinus tachycardia is a compensatory response to hypotension, not a primary arrhythmia.  **Pulmonary Embolism (PE)**: Echocardiography shows pericardial effusion, not right ventricular strain (e.g., McConnell’s sign, dilated RV). Chest x-ray also lacks oligemic lung fields or Hampton’s hump.   3. **Context of Recent TAVR**:   TAVR is associated with procedural complications like **pericardial effusion/tamponade** (e.g., from coronary artery perforation, aortic injury, or guidewire trauma).  **Conclusion**: The combination of **hypotension, JVD, sinus tachycardia, and pericardial effusion** in a post-TAVR patient strongly supports **cardiac tamponade** as the diagnosis. Immediate pericardiocentesis is required to relieve obstruction and stabilize the patient. |
| Mang2 | **Most Appropriate Next Steps: ✅ Consult cardiothoracic surgeon ✅ Pericardiocentesis, done at bedside ✅ 0.9% saline solution, 1 L IV bolus**  **Rationale:**   1. **Pericardiocentesis at bedside:**   **Immediate definitive treatment for cardiac tamponade to relieve pericardial pressure and restore hemodynamic stability. Bedside drainage is preferred in emergencies.**   2. **Consult cardiothoracic surgeon:**   **Surgical backup is critical for potential complications (e.g., recurrent effusion, need for pericardial window) or if bedside drainage fails. Post-TAVR tamponade often requires multidisciplinary management.**   3. **IV saline bolus:**   **Temporarily improves preload and cardiac output in hypotensive patients while preparing for pericardiocentesis. Avoid excessive fluid if signs of volume overload exist, but hypotension supports judicious use.**  **Excluded Options:**   - **Heparin 5000 U IV: Contraindicated in tamponade (no evidence of thrombotic etiology; heparin may worsen bleeding if effusion is hemorrhagic, e.g., post-TAVR).** - **Pericardiocentesis in operating room: Not first-line unless bedside drainage is unsafe (e.g., loculated effusion) or immediate surgical intervention is planned.**   **Key Considerations:**   - **Post-TAVR context: High suspicion for procedure-related effusion (e.g., myocardial perforation).** - **Fluid management: Use IV fluids cautiously to support preload without exacerbating right-heart strain.**   **Final Answer:**   - **Consult cardiothoracic surgeon** - **Pericardiocentesis, done at bedside** - **0.9% saline solution, 1 L IV bolus** | **Correct Answers**: ✅ **Consult cardiothoracic surgeon** ✅ **Pericardiocentesis, done at bedside**  **Rationale:**   1. **Pericardiocentesis at Bedside**:   **Immediate Action**: Pericardiocentesis is the definitive treatment for cardiac tamponade. Bedside drainage is prioritized in unstable patients to rapidly relieve pericardial pressure and restore cardiac output.  **Safety**: Ultrasound-guided pericardiocentesis is safe and effective in most cases, even in emergency settings.   2. **Consult Cardiothoracic Surgeon**:   **Surgical Backup**: Required if pericardiocentesis fails, fluid reaccumulates, or the effusion is loculated/complex (e.g., malignant, traumatic).  **Definitive Surgical Management**: A pericardial window or pericardiectomy may be needed for recurrent or refractory effusions.  **Why Other Options Are Incorrect:**   - **0.9% Saline IV Bolus**:   Fluids may transiently improve preload in **early** tamponade but are **not definitive**. Once tamponade is confirmed, pericardiocentesis is mandatory. Excessive fluids can worsen hemodynamics if tamponade progresses.   - **Heparin 5000 U IV**:   Indicated for thromboembolic events (e.g., pulmonary embolism), which are not part of this clinical scenario. Heparin is contraindicated in tamponade unless there is a concurrent thrombotic condition (not mentioned here).   - **Pericardiocentesis in an Operating Room**:   Reserved for **surgical drainage** (e.g., traumatic hemopericardium, purulent effusions) or if bedside pericardiocentesis is unsafe. Bedside drainage is faster and preferred in acute tamponade.  **Critical Considerations:**   - **Post-Drainage Care**:   Send pericardial fluid for analysis (cell count, cultures, cytology, PCR) to identify the cause (e.g., infection, malignancy).  Monitor for reaccumulation with repeat echocardiography.   - **Etiology Workup**:   Investigate underlying causes (e.g., viral PCR, autoimmune serology, malignancy screening).  **Immediate Priority**: Relieve tamponade with pericardiocentesis while preparing for potential surgical intervention. | **Answer:**   - **Consult cardiothoracic surgeon** - **Pericardiocentesis, done at bedside**   **Rationale:**   1. **Pericardiocentesis at bedside**:   This is the **definitive, life-saving intervention** for cardiac tamponade. Bedside ultrasound-guided pericardiocentesis is preferred for rapid decompression of the pericardium, especially in hemodynamically unstable patients (e.g., BP 80/35 mmHg).   2. **Consult cardiothoracic surgeon**:   Post-TAVR tamponade is often caused by **procedural complications** (e.g., aortic injury, coronary perforation) that may require surgical repair. Even after successful pericardiocentesis, surgical backup is critical if bleeding persists, fluid reaccumulates, or structural damage is suspected.  **Why Other Options Are Less Appropriate:**   - **0.9% saline solution, 1 L IV bolus**:   While **small fluid boluses** (e.g., 250–500 mL) may transiently improve preload in tamponade, **large-volume resuscitation (1 L)** can worsen right-sided heart pressures and exacerbate hemodynamic collapse. Fluids should be used cautiously and only as a bridge to definitive treatment.   - **Heparin 5000 U IV**:   Contraindicated here. Tamponade post-TAVR is often due to **hemopericardium** (blood in the pericardium), and anticoagulation would worsen bleeding. Heparin is only appropriate if a concurrent condition (e.g., pulmonary embolism) mandates it, which is not described here.   - **Pericardiocentesis in an operating room**:   Reserved for **complex cases** (e.g., loculated effusions, coagulopathy, or anticipated need for immediate surgery). Bedside pericardiocentesis is faster and standard for unstable patients.  **Key Takeaway:** Immediate pericardiocentesis and surgical consultation are the cornerstones of managing post-procedural cardiac tamponade. Avoid interventions that delay definitive treatment (e.g., excessive fluids) or exacerbate bleeding (e.g., heparin). |
| Case 27 |  |  |  |
| Diff | **Differential Diagnoses That Cannot Be Excluded: ✅ Brain tumor ✅ Cushing syndrome ✅ Hypothyroidism ✅ Muscular dystrophy ✅ Polymyositis or dermatomyositis**  **Rationale:**   1. **Brain tumor:**   **Memory impairment and brisk reflexes raise concern for intracranial pathology (e.g., pituitary adenoma causing Cushing’s disease or another mass). Imaging (MRI) is required to exclude.**   2. **Cushing syndrome:**   **Strongly supported by weight gain, purple striae, acne, proximal muscle weakness, diabetes, and neuropsychiatric symptoms. Requires biochemical confirmation (e.g., 24-hour urinary cortisol).**   3. **Hypothyroidism:**   **Overlapping symptoms include weight gain, fatigue, and depression. Brisk reflexes are atypical but do not definitively exclude hypothyroidism without thyroid function tests (TSH, free T4).**   4. **Muscular dystrophy:**   **Proximal weakness (quadriceps) could align with late-onset muscular dystrophy. Normal reflexes and lack of atrophy reduce likelihood, but CK levels and EMG are needed for exclusion.**   5. **Polymyositis/dermatomyositis:**   **Proximal muscle weakness without pain or rash (dermatomyositis) is possible. Normal muscle enzymes (CK) would argue against this, but testing is required.**  **Excluded Diagnoses:**   - **Cavernous sinus thrombosis: No features (e.g., headache, ophthalmoplegia, chemosis).** - **Myasthenia gravis: Weakness is non-fatigable and reflexes are brisk, inconsistent with myasthenia.** - **Polymyalgia rheumatica: Absence of pain/stiffness in shoulders/hips and normal inflammatory markers (if tested) make this unlikely.** - **Uncontrolled diabetes: Already diagnosed; symptoms (e.g., thirst, polyuria) are explained by diabetes, but other features (striae, proximal weakness) point to Cushing’s.**   **Key Missing Workup:**   - **Hormonal studies (cortisol, ACTH, TSH).** - **Imaging (pituitary MRI, muscle/nerve studies).** - **Lab tests (CK, ANA, anti-acetylcholine receptor antibodies).**   **Final Answer:**   - **Brain tumor** - **Cushing syndrome** - **Hypothyroidism** - **Muscular dystrophy** - **Polymyositis or dermatomyositis** | Based on the history, physical examination, and available labs, the following differential diagnoses cannot be excluded:   1. **Brain tumor**:   The patient’s CN III palsy (down-and-out eye deviation, ptosis) and hypercortisolism suggest a pituitary macroadenoma. However, other intracranial masses (e.g., meningioma, metastasis) could also cause similar compressive symptoms. Imaging (MRI) is required to confirm the etiology.   2. **Cavernous sinus thrombosis**:   While less likely due to the absence of fever, headache, or proptosis, cavernous sinus thrombosis can present with isolated CN III palsy. Imaging or vascular studies are needed to rule this out.   3. **Hypothyroidism**:   Symptoms of weight gain, fatigue, and depression overlap with hypothyroidism. However, brisk reflexes and lack of other hypothyroid signs (e.g., dry skin, bradycardia) make this less likely. Thyroid function tests (TSH, free T4) are required for exclusion.   4. **Myasthenia gravis**:   Proximal muscle weakness can occur in myasthenia gravis, though the non-fatigable nature of the weakness and brisk reflexes are atypical. Antibody testing (anti-AChR, anti-MuSK) or electromyography would be needed to exclude this.  **Excluded Diagnoses**:   - **Cushing syndrome**: Already confirmed by elevated cortisol and clinical features. - **Diabetes, uncontrolled**: Diagnosed but does not explain the full clinical picture (e.g., striae, proximal weakness). - **Muscular dystrophy**: Rapid symptom onset (3 months) and normal CK make this unlikely. - **Polymyalgia rheumatica**: Absence of pain/stiffness and normal inflammatory markers argue against this. - **Polymyositis/dermatomyositis**: Normal CK and lack of rash or muscle tenderness exclude these.   **Key Next Steps**:   - **Brain MRI** to evaluate for pituitary tumor or other intracranial pathology. - **Thyroid function tests** and **myasthenia gravis antibody testing** to rule out overlapping conditions. - **Imaging/angiography** if cavernous sinus thrombosis is suspected. | **Differential Diagnoses That Cannot Be Excluded:**   1. **Brain tumor**   **Rationale**: The left third nerve palsy (ptosis, anisocoria) raises concern for a compressive lesion (e.g., pituitary tumor, aneurysm). Neuroimaging (MRI) is required to exclude structural causes.   2. **Cushing syndrome**   **Rationale**: Classic features (rapid weight gain, striae, acne, proximal muscle weakness, uncontrolled diabetes, psychiatric symptoms) strongly suggest hypercortisolism. Confirmation with cortisol testing is needed.   3. **Hypothyroidism**   **Rationale**: Overlapping symptoms (weight gain, fatigue, depression) could suggest hypothyroidism, though brisk reflexes are atypical. TSH levels have not been reported, so this cannot be ruled out.   4. **Polymyositis or dermatomyositis**   **Rationale**: Proximal muscle weakness (e.g., quadriceps) is a hallmark, though typically accompanied by elevated CK or skin rash (dermatomyositis). CK levels are not provided, so these remain possibilities.   5. **Muscular dystrophy**   **Rationale**: Symmetric proximal weakness can occur in muscular dystrophy. Normal reflexes and lack of family history reduce likelihood, but definitive exclusion requires CK and genetic testing.  **Why Other Diagnoses Are Less Likely (or Can Be Excluded):**   - **Cavernous sinus thrombosis**: No fever, headache, or involvement of multiple cranial nerves (e.g., IV, V, VI). Unlikely without systemic signs of infection. - **Myasthenia gravis**: Weakness in myasthenia fluctuates and worsens with activity. This patient’s **constant weakness** and **pupil-involving third nerve palsy** are atypical. - **Polymyalgia rheumatica**: Presents with **pain/stiffness** (not isolated weakness) in shoulders/hips, typically in older adults. The patient’s age (54) and lack of pain make this unlikely. - **Uncontrolled diabetes**: While diabetes explains thirst, urination, and neuropathy, it does not account for **proximal muscle weakness**, skin changes, or third nerve palsy.   **Key Next Steps**:   - **Confirm Cushing syndrome**: 24-hour urinary free cortisol, late-night salivary cortisol, dexamethasone suppression test. - **Evaluate third nerve palsy**: MRI brain/orbits to rule out tumor/aneurysm. - **Check TSH, CK, ESR/CRP**: To exclude hypothyroidism, inflammatory myopathies, or polymyalgia rheumatica.   **Conclusion**: The presentation is most consistent with **Cushing syndrome**, but other diagnoses (e.g., brain tumor, hypothyroidism, inflammatory myopathy) require exclusion through targeted testing. |
| Diag1 | **Most Appropriate Next Steps: ✅ Midnight serum cortisol ✅ MRI of the cranium ✅ Serum TSH ✅ Serum free T4 (FT4) ✅ Serum electrolyte concentrations**  **Rationale:**   1. **Midnight serum cortisol:**   **Critical to confirm hypercortisolism (Cushing’s syndrome). Elevated levels at midnight (when cortisol should be low) strongly support the diagnosis.**   2. **MRI of the cranium:**   **Required to evaluate for a pituitary adenoma (suspected in Cushing’s disease) or other intracranial masses (e.g., brain tumor), given the patient’s neuropsychiatric symptoms (memory impairment) and possible CN III palsy.**   3. **Serum TSH and free T4:**   **Essential to assess thyroid function and exclude hypothyroidism, which can mimic symptoms of fatigue, weight gain, and depression.**   4. **Serum electrolytes:**   **Hypercortisolism can cause hypokalemia and metabolic alkalosis. Electrolyte abnormalities may also arise from muscle breakdown (e.g., in myopathy).**  **Other Considerations:**   - **CBC and LFTs are reasonable as general tests but less specific to the current presentation.** - **LDH and acetylcholine receptor antibodies are less relevant here:**   **LDH is nonspecific for muscle disorders (prefer CK, which is not listed).**  **Myasthenia gravis is unlikely due to non-fatigable weakness and brisk reflexes.**  **Excluded Options:**   - **Blood glucose/ketones: Already diagnosed with diabetes; not urgent for diagnosis.** - **PTH, protein electrophoresis: No clinical suspicion for hyperparathyroidism or plasma cell disorders.**   **Final Answer:**   - **Midnight serum cortisol** - **MRI of the cranium** - **Serum TSH** - **Serum free T4** - **Serum electrolyte concentrations** | The most appropriate next steps for this patient, based on the clinical presentation and suspected differential diagnoses, are:   1. **Midnight serum cortisol**: Essential to confirm hypercortisolism, as elevated levels at midnight support the diagnosis of Cushing’s syndrome. 2. **MRI of the cranium**: Critical to evaluate for a pituitary macroadenoma (suspected in Cushing’s disease) and assess cavernous sinus involvement causing the CN III palsy. 3. **Serum acetylcholine receptor antibody levels**: Necessary to rule out myasthenia gravis, as proximal muscle weakness and diplopia can overlap with this condition. 4. **Serum electrolyte concentrations**: Important to assess for hypokalemia (common in hypercortisolism) and metabolic derangements. 5. **Serum TSH** and **serum free T4 (FT4)**: Required to exclude hypothyroidism, which can mimic symptoms of fatigue, weight gain, and depression.   **Excluded Options**:   - **Blood glucose/ketones**: While diabetes is present, these are less urgent for diagnosis and more relevant for ongoing management. - **CBC/LFTs/LDH/PTH/protein electrophoresis**: Not directly related to the suspected diagnoses (Cushing’s syndrome, pituitary tumor, myasthenia gravis, or hypothyroidism). - **Parathyroid hormone**: No clinical evidence of hyperparathyroidism.   **Rationale**: Prioritize tests that confirm or exclude life-threatening or treatable conditions (e.g., pituitary tumor, myasthenia gravis) and validate the leading diagnosis (Cushing’s syndrome). Thyroid testing ensures hypothyroidism is not contributing to symptoms. Electrolytes address metabolic complications of hypercortisolism. | **Most Appropriate Next Steps:**   1. **Midnight serum cortisol**   **Rationale**: Essential to confirm hypercortisolism in suspected Cushing’s syndrome. Elevated midnight cortisol supports the diagnosis.   2. **MRI of the cranium**   **Rationale**: Evaluate for pituitary adenoma (common in Cushing’s disease) or structural lesions causing the left third nerve palsy (e.g., aneurysm, tumor).   3. **Serum TSH** and **Serum free T4 (FT4)**   **Rationale**: Hypothyroidism can mimic symptoms like weight gain, fatigue, and depression. TSH/FT4 levels are necessary to exclude thyroid dysfunction.   4. **Serum electrolyte concentrations**   **Rationale**: Hypokalemia, metabolic alkalosis, or hypernatremia may occur in Cushing’s syndrome due to mineralocorticoid effects of cortisol.   5. **Blood glucose**   **Rationale**: Monitor glycemic control in his poorly controlled diabetes.   6. **Serum liver function studies (LFT)**   **Rationale**: Relevant given his history of primary biliary cirrhosis; assess for hepatic contributions to metabolic derangements.  **Less Urgent or Lower Priority**:   - **Serum lactate dehydrogenase (LDH)**: Non-specific for myopathy; CK is more specific for inflammatory myositis (not listed). - **Serum acetylcholine receptor antibody levels**: Unlikely given constant weakness and pupil-involving third nerve palsy (atypical for myasthenia gravis). - **Serum and urine protein electrophoresis**: No clinical clues for plasma cell dyscrasias (e.g., bone pain, renal failure). - **Blood ketones**: No signs of diabetic ketoacidosis (e.g., nausea, vomiting, acute illness). - **Serum parathyroid hormone (PTH)**: No symptoms or lab evidence of calcium disorders.   **Conclusion**: Prioritize **midnight serum cortisol**, **MRI cranium**, **TSH/FT4**, **electrolytes**, **blood glucose**, and **LFTs** to confirm Cushing’s syndrome, evaluate the cranial nerve palsy, and address metabolic complications. Other tests are less relevant based on current findings. |
| Dx1 | **Answer: ✅ Cushing syndrome**  **Explanation: Cushing syndrome (specifically Cushing’s disease due to the pituitary macroadenoma) is the primary diagnosis that unifies all findings:**   1. **Hypercortisolism:**   **Midnight serum cortisol = 43.5 mcg/dL (normal: <1.8 mcg/dL), diagnostic of Cushing’s syndrome.**  **Pituitary macroadenoma on MRI confirms ACTH-dependent Cushing’s disease.**   2. **Secondary Effects:**   **Uncontrolled diabetes: Cortisol-induced insulin resistance (glucose = 306 mg/dL).**  **Hypokalemia: Cortisol’s mineralocorticoid activity (K+ = 2.9 mEq/L).**  **Hypothyroidism: Pituitary tumor compresses thyrotrophs → low TSH/FT4 (central hypothyroidism).**  **Why Not the Others:**   - **Diabetes, hypokalemia, and hypothyroidism are complications of Cushing’s syndrome, not standalone diagnoses.**   **Final Answer: Cushing syndrome.** | **Answer:** **Cushing syndrome**  **Rationale**: The patient’s findings are all secondary to **Cushing syndrome caused by an ACTH-secreting pituitary macroadenoma (Cushing’s disease)**:   1. **Midnight serum cortisol** is markedly elevated (**43.5 mcg/dL**; normal <7.5 mcg/dL), confirming hypercortisolism. 2. **Hypokalemia** (**K+ 2.9 mEq/L**) results from cortisol’s mineralocorticoid activity, promoting renal potassium wasting. 3. **Uncontrolled diabetes** (**glucose 306 mg/dL**) stems from cortisol-induced insulin resistance. 4. **Secondary hypothyroidism** (low **TSH** and **FT4**) is due to compression of normal pituitary tissue by the tumor, impairing TSH secretion. 5. **Pituitary macroadenoma with cavernous sinus invasion** on MRI directly explains the hypercortisolism (ACTH-driven) and cranial nerve III palsy.   While hypokalemia, diabetes, and hypothyroidism are significant, they are **complications of the underlying Cushing syndrome**. The unifying diagnosis is **Cushing syndrome secondary to Cushing’s disease (pituitary ACTH-secreting adenoma)**. | **Answer:** **Cushing syndrome**  **Rationale:**   - **Cushing syndrome** (specifically **Cushing’s disease**, caused by the ACTH-secreting pituitary macroadenoma) is the **root diagnosis** explaining all the patient’s findings:   1. **Hypercortisolism**:   Elevated midnight serum cortisol (43.5 mcg/dL; normal <7.5 mcg/dL) confirms hypercortisolism.  Cortisol excess drives **uncontrolled diabetes** (glucose 306 mg/dL) via insulin resistance and gluconeogenesis.  Cortisol’s mineralocorticoid activity causes **hypokalemia** (K⁺: 2.9 mEq/L).   2. **Pituitary macroadenoma**:   The tumor compresses surrounding structures, leading to **central hypothyroidism** (low TSH and FT4).  Invasion of the cavernous sinus explains the **left third nerve palsy**.   - **Uncontrolled diabetes**, **hypokalemia**, and **hypothyroidism** are **secondary complications** of the underlying Cushing syndrome. Cushing’s disease unifies all findings into a single pathophysiological process.   **Why Not the Other Options?**   - **Uncontrolled diabetes**: A consequence of cortisol excess, not the primary diagnosis. - **Hypokalemia**: A metabolic derangement caused by cortisol’s mineralocorticoid effects. - **Hypothyroidism**: Secondary to pituitary dysfunction from the macroadenoma, not a standalone diagnosis.   **Conclusion:** **Cushing syndrome** (due to Cushing’s disease) is the unifying diagnosis. |
| Diag2 | **Most Appropriate Next Steps: ✅ Inferior petrosal sinus sampling (IPSS) for serum ACTH and cortisol levels ✅ Serum ACTH level**  **Rationale:**   1. **Serum ACTH level:**   **Critical to confirm ACTH-dependent Cushing’s syndrome (pituitary vs. ectopic).**  **Expected result: Elevated ACTH (supports pituitary or ectopic source).**   2. **Inferior Petrosal Sinus Sampling (IPSS):**   **Gold standard to localize the source of ACTH secretion.**  **Differentiates Cushing’s disease (pituitary ACTH) from ectopic ACTH secretion, even with a visible pituitary macroadenoma (confirms the tumor is functional).**  **Why Not Others:**   - **Chest x-ray: Not urgent here. Ectopic ACTH (e.g., lung tumor) is less likely given the MRI findings.** - **Oral glucose tolerance test: Unnecessary (diabetes is already confirmed).** - **CRH test: Less specific than IPSS; often used adjunctively during IPSS.** - **LH, prolactin, testosterone: Part of a full pituitary workup but not priority for diagnosing Cushing’s etiology.**   **Final Answer:**   - **Inferior petrosal sinus sampling** - **Serum ACTH level** | **Answer:** The most appropriate next steps are:   - **Inferior petrosal sinus sampling for serum ACTH and cortisol levels** - **Serum ACTH level** - **Serum prolactin level** - **Chest x-ray**   **Rationale:**   1. **Inferior petrosal sinus sampling (IPSS)**:   **Gold standard** to confirm the pituitary origin of ACTH secretion (Cushing’s disease) versus ectopic ACTH syndrome. Even with a pituitary adenoma on MRI, IPSS is critical because imaging alone cannot confirm functionality (e.g., incidentalomas or necrotic tumors may not secrete ACTH).   2. **Serum ACTH level**:   Essential to distinguish **ACTH-dependent** (pituitary or ectopic) from **ACTH-independent** (adrenal) Cushing syndrome. A high ACTH level supports ACTH-dependent disease (expected here), while a low ACTH level would suggest adrenal pathology.   3. **Serum prolactin level**:   Pituitary macroadenomas may co-secrete prolactin (e.g., mixed ACTH-prolactin tumors). Elevated prolactin would guide additional management (e.g., dopamine agonists).   4. **Chest x-ray**:   Screens for **ectopic ACTH sources** (e.g., lung carcinoid or small cell carcinoma), which can mimic Cushing’s disease. Even with a pituitary lesion, ruling out ectopic sources is necessary.  **Why other options are less appropriate:**   - **Oral glucose tolerance test**: Not relevant here (diagnoses diabetes or acromegaly; hyperglycemia is already explained by cortisol excess). - **CRH test**: Less definitive than IPSS. While CRH stimulation can help differentiate pituitary vs. ectopic ACTH secretion, it is often performed during IPSS for enhanced accuracy. - **Serum LH/testosterone**: Secondary hypogonadism may exist due to pituitary compression, but this is not urgent in the acute evaluation of Cushing’s syndrome. - **Serum testosterone**: Relevant only if hypogonadism is suspected (not a priority here).   **Immediate focus**: Confirm the source of ACTH (pituitary vs. ectopic) and assess for comorbidities (e.g., co-secreting prolactinoma, lung tumors). | **The most appropriate next steps are:**   1. **Inferior petrosal sinus sampling (IPSS) for serum ACTH and cortisol levels**   **Why**: IPSS is the gold standard to confirm the pituitary as the source of ACTH secretion, especially when MRI shows a macroadenoma. It distinguishes Cushing’s disease (pituitary origin) from ectopic ACTH syndrome.   2. **Serum ACTH level**   **Why**: Elevated ACTH confirms ACTH-dependent Cushing syndrome (e.g., pituitary or ectopic). Low ACTH would suggest adrenal pathology (ACTH-independent), but the patient’s MRI and clinical findings strongly favor ACTH-dependent disease.   3. **Serum prolactin level**   **Why**: Pituitary macroadenomas may co-secrete prolactin (e.g., mixed ACTH-prolactin tumors). Elevated prolactin supports a pituitary origin and guides management.   4. **Serum LH level** and **Serum testosterone level**   **Why**: To assess for hypogonadism secondary to pituitary dysfunction. The macroadenoma may compress gonadotrophs, disrupting LH/FSH and testosterone production.  **Why Other Options Are Less Appropriate:**   - **Chest x-ray**: While ectopic ACTH-secreting tumors (e.g., lung carcinoid) can cause Cushing syndrome, the MRI already identifies a pituitary macroadenoma. IPSS is more specific for distinguishing pituitary vs. ectopic sources. - **Oral glucose tolerance test**: Not relevant here. The patient already has confirmed hyperglycemia (glucose 306 mg/dL), and this test is used for diagnosing diabetes or acromegaly. - **CRH test**: Less definitive than IPSS. CRH testing (measuring ACTH response) can help differentiate pituitary vs. ectopic causes but is not routinely preferred over IPSS. - **Serum LH/testosterone/prolactin**: Already included above as part of pituitary axis evaluation.   **Key Takeaway**: After confirming Cushing syndrome, the priority is to **localize the ACTH source** (IPSS and serum ACTH) and **evaluate for additional pituitary hormone deficiencies** (prolactin, LH, testosterone). This guides definitive treatment (e.g., pituitary surgery). |
| Dx2 | **Answer: ✅ Pituitary ACTH secretion (Cushing’s disease)**  **Rationale:**   1. **ACTH-dependent Cushing’s syndrome:**   **Elevated ACTH (188 pg/mL) and high cortisol confirm ACTH dependence, ruling out adrenal tumors/iatrogenic causes (which suppress ACTH).**   2. **Pituitary origin:**   **MRI showing a macroadenoma + normal chest x-ray (no ectopic ACTH source).**  **Ectopic ACTH (e.g., lung tumor) is less likely with a normal chest x-ray and visible pituitary lesion.**   3. **Supporting evidence:**   **Hypogonadotropic hypogonadism (low LH/FSH/testosterone) suggests pituitary mass effect.**  **Normal prolactin excludes prolactinoma/stalk compression.**  **Why Not Other Options:**   - **Corticosteroid-secreting adrenal tumor: ACTH would be low (ACTH-independent).** - **Ectopic ACTH secretion: Typically associated with abnormal chest imaging (e.g., lung mass) or other tumors (e.g., carcinoid).** - **Iatrogenic corticosteroid use: ACTH is suppressed (not elevated).**   **Conclusion: The findings align with Cushing’s disease (pituitary ACTH-secreting adenoma).** | **Answer:** **Pituitary ACTH secretion (Cushing's disease)**  **Rationale:**   1. **Elevated ACTH (188 pg/mL)**:Confirms **ACTH-dependent Cushing syndrome**, ruling out adrenal tumors (ACTH-independent) and iatrogenic causes (which suppress ACTH). 2. **Pituitary macroadenoma with cavernous sinus invasion**: Strongly supports a pituitary source of ACTH. While incidentalomas exist, the combination of a visible adenoma and biochemical evidence of ACTH excess makes Cushing’s disease the most likely diagnosis. 3. **Normal chest x-ray**: Reduces suspicion for ectopic ACTH secretion (e.g., lung tumors). Though ectopic sources can be occult (e.g., bronchial carcinoid), the pituitary lesion is the more probable culprit here. 4. **Hypogonadotropic hypogonadism (low LH/FSH/testosterone)**: Likely due to mass effects of the pituitary macroadenoma, further implicating the pituitary as the source of dysfunction.   **Why not ectopic ACTH?** Ectopic ACTH is typically associated with extremely high ACTH levels (>200 pg/mL) and hypokalemia (due to mineralocorticoid effects). While this patient’s ACTH is elevated (188 pg/mL), the presence of a pituitary macroadenoma and absence of chest lesions make ectopic ACTH less likely.  **Conclusion:** The findings strongly align with **Cushing’s disease** (pituitary ACTH-secreting adenoma). Confirmatory IPSS would be the next step, but the current data overwhelmingly favor this diagnosis. | **The most likely cause of Cushing syndrome in this patient is:** **Pituitary ACTH secretion (Cushing's disease)**  **Key Supporting Evidence:**   1. **Elevated ACTH levels (188 pg/mL)**:   Confirms **ACTH-dependent Cushing syndrome**, ruling out **adrenal tumors** (ACTH-independent) or **iatrogenic causes** (which suppress ACTH).   2. **Pituitary Macroadenoma on MRI**:   Pituitary adenomas are the most common cause of endogenous ACTH-dependent Cushing syndrome (~70% of cases).   3. **Normal Chest X-ray**:   Reduces suspicion for **ectopic ACTH secretion** (e.g., lung carcinoid), though occult ectopic sources are not fully excluded.   4. **Hypogonadotropic Hypogonadism (low LH/FSH/testosterone)**:   Suggests pituitary dysfunction, likely due to compression of gonadotrophs by the macroadenoma.   5. **Normal Prolactin**:   Makes coexisting prolactinoma or significant pituitary stalk compression (which raises prolactin) less likely.  **Why Other Options Are Less Likely:**   - **Corticosteroid-secreting adrenal tumor**: ACTH would be suppressed (normal/low), but here ACTH is elevated. - **Ectopic ACTH secretion**: Less likely with a pituitary macroadenoma on MRI and normal chest x-ray, though IPSS may still be needed for confirmation. - **Iatrogenic corticosteroid use**: Exogenous steroids suppress ACTH (low/normal levels), which is inconsistent with elevated ACTH here.   **Conclusion**: The combination of **ACTH-dependent hypercortisolism**, **pituitary macroadenoma**, and **hypogonadotropic hypogonadism** strongly supports **Cushing's disease** as the underlying cause. |
| Mang | **Appropriate Management Steps: ✅ Transsphenoidal adenomectomy ✅ Metyrapone 250 mg orally 4 times a day ✅ Pasireotide 600 mcg once a day as monotherapy (in select cases)**  **Rationale:**   1. **Transsphenoidal adenomectomy:**   **First-line definitive treatment for confirmed pituitary ACTH-secreting adenoma.**   2. **Metyrapone:**   **Steroidogenesis inhibitor to lower cortisol preoperatively or for patients who are not surgical candidates.**   3. **Pasireotide:**   **Somatostatin analog that reduces ACTH secretion in some patients with mild/moderate disease or recurrent tumors.**  **Other Options Explained:**   - **Bilateral adrenalectomy: Reserved for refractory cases or failed pituitary surgery (not first-line).** - **Cabergoline: Rarely effective (dopamine agonists have limited efficacy in Cushing’s disease).** - **Desmopressin: Used for diabetes insipidus (not relevant here).** - **Furosemide/Ramipril: Symptomatic management (e.g., hypertension, edema) but not disease-specific.** - **Lymecycline: Treats acne (symptomatic, not curative).** - **Pegvisomant: For acromegaly, not Cushing’s.** - **Watch and wait: Inappropriate once a pituitary adenoma is confirmed.**   **Conclusion: Prioritize surgery and use metyrapone/pasireotide for bridging or adjunctive therapy.** | **Appropriate Management Steps:**   1. **Metyrapone 250 mg orally 4 times a day** 2. **Transsphenoidal adenomectomy** 3. **Bilateral adrenalectomy** (if surgery fails or is not feasible)   **Rationale:**  **1. Metyrapone**   - **Role**: Inhibits cortisol synthesis by blocking 11β-hydroxylase. - **Use**: Preoperative control of hypercortisolism or as a bridge to definitive therapy.   **2. Transsphenoidal Adenomectomy**   - **First-line treatment**: Definitive management for pituitary ACTH-secreting adenomas. - **Goal**: Remove the tumor to normalize ACTH/cortisol levels.   **3. Bilateral Adrenalectomy**   - **Rescue therapy**: Reserved for patients with persistent/recurrent Cushing’s disease after failed surgery/radiation, or severe refractory hypercortisolism.   **Why Other Options Are Inappropriate:**   - **Cabergoline**: Rarely effective in Cushing’s disease (primarily for prolactinomas). - **Pasireotide**: Limited efficacy (approved but used in select cases; often causes hyperglycemia). - **Desmopressin**: Used for diabetes insipidus (not relevant here). - **Furosemide/Ramipril**: Manage hypertension/edema but do not address the underlying disease. - **Pegvisomant**: For acromegaly (not Cushing’s). - **Lymecycline**: Symptomatic acne treatment (not disease-modifying). - **“Watch and wait”**: Not appropriate for confirmed Cushing’s syndrome (requires active intervention).   **Key Point**: **Transsphenoidal surgery is mandatory** unless contraindicated. Medical therapy (metyrapone) and bilateral adrenalectomy are adjunctive/second-line options. | **Appropriate Management Steps for Pituitary ACTH-Dependent Cushing Syndrome (Cushing’s Disease):**   1. **Transsphenoidal adenomectomy**   **First-line treatment** to surgically remove the ACTH-secreting pituitary adenoma. Curative in ~70-80% of cases when performed by an experienced neurosurgeon.   2. **Metyrapone 250 mg orally 4 times a day**   A **cortisol synthesis inhibitor** used to rapidly lower cortisol levels preoperatively (to reduce surgical complications) or as a bridge to surgery/radiation. Also used for long-term control if surgery is contraindicated or unsuccessful.   3. **Pasireotide 600 mcg once a day as monotherapy**   A **somatostatin receptor ligand** that suppresses ACTH secretion. Reserved for patients with persistent hypercortisolism after failed surgery or those unfit for surgery. Note: Requires monitoring for hyperglycemia.   4. **Bilateral adrenalectomy**   Indicated if **transsphenoidal surgery fails**, the tumor is unresectable, or hypercortisolism is life-threatening. Leads to permanent adrenal insufficiency, requiring lifelong glucocorticoid/mineralocorticoid replacement.  **Why Other Options Are Less Appropriate:**   - **Cabergoline**: Less effective in Cushing’s disease (only ~25-40% response rate). Primarily used in prolactinomas or as adjunctive therapy. - **Desmopressin**: Used for diabetes insipidus (not relevant here). - **Furosemide**: Avoid in hypercortisolism due to risk of hypokalemia (cortisol excess mimics mineralocorticoid effects). - **Pegvisomant**: For acromegaly (GH-secreting tumors), not Cushing’s. - **Ramipril**: Hypertension management is important, but this is a general supportive measure, not specific to Cushing’s treatment. - **Treat acne with lymecycline**: Symptomatic management of hypercortisolism (e.g., acne, hirsutism) is secondary to addressing the underlying cause. - **Watch and wait**: Not appropriate once Cushing’s syndrome is confirmed, as chronic hypercortisolism causes irreversible morbidity (e.g., cardiovascular disease, osteoporosis).   **Key Takeaway**:   - **Prioritize definitive therapy (surgery)**. - Use **metyrapone/pasireotide** for preoperative preparation or refractory cases. - Reserve **bilateral adrenalectomy** for surgically unmanageable disease. - Avoid therapies unrelated to ACTH/cortisol suppression (e.g., desmopressin, pegvisomant). |
| Case 28 |  |  |  |
| Diff | **Diagnoses that cannot be excluded based on history and physical examination: ✅ Adrenal insufficiency ✅ Anemia ✅ GI bleeding ✅ Cancer**  **Rationale:**   1. **Adrenal insufficiency (Addison’s disease):**   **Key findings: Hyperpigmentation (knuckles/nipples), postural dizziness, weight loss, nausea/vomiting, and abdominal pain.**  **Mechanism: Possible adrenal injury during diaphragmatic hernia repair. Hyperpigmentation due to elevated ACTH is pathognomonic for Addison’s.**   2. **Anemia:**   **Key findings: Fatigue, weight loss, and exertional shortness of breath.**  **Mechanism: Chronic blood loss (e.g., occult GI bleeding) or anemia of chronic disease.**   3. **GI bleeding:**   **Key findings: Weight loss, nausea/vomiting, and generalized abdominal pain.**  **Mechanism: Occult bleeding (e.g., peptic ulcer) could explain anemia and constitutional symptoms.**   4. **Cancer:**   **Key findings: Unexplained weight loss (12 kg in 2 months) and fatigue.**  **Mechanism: Malignancy (e.g., gastrointestinal, adrenal, or metastatic) must be ruled out.**  **Other Considerations:**   - **Gastroenteritis: Chronicity (2 months) makes acute infection unlikely.** - **Heart failure: Mild exertional dyspnea could be due to anemia or deconditioning; no edema or JVD noted.** - **Hyperthyroidism/Hypothyroidism: Weight loss and palpitations could suggest hyperthyroidism, but no heat intolerance/tremors. Hypothyroidism is less likely (weight loss atypical).** - **Liver/Renal failure: No stigmata (jaundice, ascites, oliguria) or lab abnormalities reported.** - **Sepsis: No fever/chills or focal infection source; chronic presentation inconsistent.**   **Conclusion:**  **Adrenal insufficiency is the most likely diagnosis, but anemia, GI bleeding, and cancer remain on the differential until testing (e.g., cortisol/ACTH levels, CBC, endoscopy, imaging) is performed.** | **Diagnoses That Cannot Be Excluded:**   1. **Adrenal insufficiency** 2. **Anemia** 3. **Cancer** 4. **GI bleeding** 5. **Hyperthyroidism** 6. **Heart failure** 7. **Renal failure**   **Rationale:**  **Adrenal Insufficiency**   - **Key Clues**: Postural hypotension, hyperpigmentation (elevated ACTH), weight loss, nausea/vomiting, and fatigue. - **Cannot Be Excluded**: Classic presentation of primary adrenal insufficiency (Addison’s disease), especially post-surgery (possible adrenal hemorrhage).   **Anemia**   - **Key Clues**: Fatigue, palpitations, lightheadedness, and weight loss. - **Cannot Be Excluded**: Chronic anemia (e.g., iron deficiency from occult GI bleeding or chronic disease) could explain symptoms.   **Cancer**   - **Key Clues**: Significant weight loss (12 kg) and fatigue. - **Cannot Be Excluded**: Malignancy (e.g., gastrointestinal, lymphoma) must be ruled out despite his age.   **GI Bleeding**   - **Key Clues**: Generalized abdominal pain, nausea/vomiting, and weight loss. - **Cannot Be Excluded**: Occult bleeding (e.g., slow upper GI bleed) could cause anemia and hypotension.   **Hyperthyroidism**   - **Key Clues**: Weight loss, palpitations, and tachycardia. - **Cannot Be Excluded**: Atypical presentations may lack classic features (e.g., heat intolerance).   **Heart Failure**   - **Key Clues**: Exertional dyspnea and fatigue. - **Cannot Be Excluded**: Early or mild heart failure may lack edema or chest pain.   **Renal Failure**   - **Key Clues**: Fatigue, nausea, and generalized weakness. - **Cannot Be Excluded**: Chronic kidney disease could explain symptoms without overt urinary changes.   **Excluded Diagnoses:**   - **Gastroenteritis**: Symptoms are chronic (2+ months), not acute. - **Hypothyroidism**: Weight loss and decreased appetite contradict typical hypothyroidism. - **Liver Failure**: No jaundice, ascites, or stigmata (e.g., spider angiomas). - **Sepsis**: No fever, chills, or acute infectious signs; symptoms are chronic.   **Next Steps**:   - **Lab Testing**: Cortisol/ACTH, CBC, electrolytes, creatinine, TSH, LFTs, iron studies. - **Imaging**: Abdominal imaging (CT/MRI) to assess adrenal glands, GI tract, or occult malignancy. - **ECG/Echocardiogram**: If heart failure is suspected. | **Diagnoses That Cannot Be Excluded Based on Current Information:**   1. **Adrenal Insufficiency**   Strongly supported by post-surgical onset, hyperpigmentation (elevated ACTH), hypotension, weight loss, and electrolyte imbalances (if labs were suggestive). However, confirmation requires cortisol/ACTH testing.   2. **Anemia**   Symptoms (fatigue, postural dizziness, weight loss) align with chronic anemia. Possible causes include GI bleeding, nutritional deficiencies, or chronic disease. Absence of reported blood loss or lab values (e.g., hemoglobin) means it cannot be ruled out.   3. **Cancer**   Unexplained weight loss and fatigue are red flags. While less likely in a young patient, malignancies (e.g., GI, lymphoma) or paraneoplastic syndromes remain possible without imaging/biopsy.   4. **GI Bleeding**   Chronic occult bleeding (e.g., from a peptic ulcer or malignancy) could explain weight loss, anemia, and fatigue. Normal stool and non-bloody vomiting reduce likelihood but do not exclude it.   5. **Hyperthyroidism**   Overlapping symptoms: weight loss, palpitations, tachycardia. Lack of classic features (e.g., heat intolerance, tremor) makes it less likely, but thyroid function tests are needed for exclusion.   6. **Liver Failure**   Nausea, abdominal pain, and fatigue could occur in liver disease, but absence of jaundice, coagulopathy, or ascites makes it less probable. Liver function tests would clarify.  **Diagnoses Less Likely or Excluded:**   - **Gastroenteritis**: Symptoms are chronic (>2 months), inconsistent with acute infectious causes. - **Heart Failure**: No edema, JVD, or imaging evidence (e.g., cardiomegaly). Exertional dyspnea could be due to deconditioning/anemia. - **Hypothyroidism**: Weight loss and normal skin findings contradict typical features (weight gain, cold intolerance, dry skin). - **Renal Failure**: Normal urination and absence of edema/uremic symptoms make this unlikely. - **Sepsis**: Chronic symptoms and lack of fever/inflammatory markers argue against acute infection.   **Conclusion:** The most probable diagnosis remains **adrenal insufficiency**, but **anemia, cancer, GI bleeding, hyperthyroidism, and liver failure** cannot be definitively excluded without further testing (e.g., CBC, cortisol/ACTH, thyroid function, imaging). |
| Diag1 | **Appropriate Diagnostic Steps: ✅ Complete blood count (CBC) ✅ 9 AM serum cortisol ✅ Serum electrolyte concentrations, creatinine, and urea ✅ Serum TSH ✅ Serum free T4 (FT4) ✅ Serum liver function studies (LFTs) and amylase ✅ Venous blood gas for pH, bicarbonates, and lactate ✅ Urine dipstick ✅ CT scan of the abdomen ✅ Chest x-ray**  **Rationale:**   1. **Adrenal Insufficiency Workup:**   **9 AM serum cortisol: Critical for diagnosing adrenal insufficiency (low cortisol with hyperpigmentation suggests primary Addison’s).**  **Electrolytes, creatinine, urea: Hyponatremia, hyperkalemia, and elevated urea support adrenal insufficiency.**  **Venous blood gas (VBG): Assess for metabolic acidosis (e.g., adrenal crisis) or lactic acidosis.**   2. **Anemia and GI Evaluation:**   **CBC: Detect anemia (e.g., iron deficiency from occult GI bleeding or chronic disease).**   3. **Thyroid Dysfunction:**   **TSH and free T4: Rule out hyperthyroidism (weight loss, palpitations) or hypothyroidism (fatigue).**   4. **Liver/Pancreatic Causes:**   **LFTs and amylase: Exclude liver failure (abdominal pain, nausea) or pancreatitis.**   5. **Cardiopulmonary Assessment:**   **Chest x-ray: Investigate exertional dyspnea (e.g., pleural effusion, post-surgical changes).**   6. **Additional Screening:**   **Urine dipstick: Check for infection, proteinuria, or glycosuria (diabetes insipidus is unlikely but possible).**  **Excluded Options:**   - **Bedside blood glucose: Hypoglycemia is uncommon in chronic adrenal insufficiency (more relevant in acute crisis).** - **Serum calcium, troponin, acetylcholinesterase antibodies, D-dimer, plasma metanephrines: No findings strongly suggestive of hypercalcemia, myocardial injury, myasthenia gravis, VTE, or pheochromocytoma.** - **Echocardiography: Low suspicion for heart failure (no edema, JVD, or S3 gallop).**   **Conclusion:**  **The priority is confirming adrenal insufficiency (9 AM cortisol, electrolytes) while ruling out anemia, thyroid dysfunction, and structural pathology (CT abdomen, chest x-ray). Metabolic status (VBG) and liver/pancreatic enzymes further refine the diagnosis.** | **Most Appropriate Diagnostic Steps:**   1. **Complete Blood Count (CBC)**   **Rationale**: Assess for anemia (fatigue, lightheadedness) and infection. Chronic anemia from GI bleeding or malignancy is a key differential.   2. **9 AM Serum Cortisol**   **Rationale**: Critical to confirm adrenal insufficiency. Low cortisol with elevated ACTH supports Addison’s disease.   3. **Serum Electrolytes, Creatinine, Urea**   **Rationale**: Hyponatremia, hyperkalemia, and elevated creatinine are classic in adrenal insufficiency. Also evaluates renal failure.   4. **Serum TSH and Free T4 (FT4)**   **Rationale**: Rule out hyperthyroidism (weight loss, tachycardia) or hypothyroidism (fatigue).   5. **Serum Liver Function Tests (LFTs) and Amylase**   **Rationale**: Exclude liver failure (nausea, weight loss) or pancreatitis (abdominal pain).   6. **Venous Blood Gas (pH, Bicarbonate, Lactate)**   **Rationale**: Assess for metabolic acidosis (e.g., adrenal crisis) or lactic acidosis (sepsis, hypoperfusion).   7. **CT Scan of the Abdomen**   **Rationale**: Evaluate adrenal glands (atrophy, hemorrhage, infiltration) and rule out malignancy or GI pathology.   8. **Urine Dipstick**   **Rationale**: Screen for renal disease (proteinuria) or infection.  **Additional Considerations if Clinical Suspicion Arises:**   - **Echocardiography**: If heart failure is suspected (exertional dyspnea). - **Chest X-ray**: General assessment for infection or cardiopulmonary disease.   **Less Relevant or Unnecessary Tests:**   - **Bedside Blood Glucose**: Hypoglycemia is uncommon in chronic adrenal insufficiency unless fasting. - **Serum Calcium**: No specific indication (e.g., no bone/joint symptoms). - **Serum Troponin**: Not indicated without chest pain or acute coronary symptoms. - **Plasma Metanephrines**: Pheochromocytoma is unlikely (no paroxysmal hypertension or headaches). - **Serum Acetylcholinesterase Antibodies**: No neuromuscular symptoms (e.g., weakness, ptosis). - **D-dimer**: Not indicated without signs of thromboembolism (e.g., acute dyspnea, chest pain).   **Summary**: Prioritize tests that confirm adrenal insufficiency (cortisol, electrolytes), evaluate key differentials (CBC, TSH/FT4, LFTs), and identify structural causes (CT abdomen). Venous blood gas and urine dipstick add rapid insights into metabolic/renal status. Avoid unnecessary tests without clinical correlation. | **Most Appropriate Steps for Diagnosis and Treatment:**   1. **Complete Blood Count (CBC)**   Essential to evaluate for **anemia** (contributing to fatigue, postural dizziness) and chronic blood loss (e.g., occult GI bleeding).   2. **9 AM Serum Cortisol**   **Critical for diagnosing adrenal insufficiency**. A low morning cortisol level supports Addison’s disease. Follow-up with ACTH testing would confirm primary vs. secondary insufficiency.   3. **Serum Electrolytes, Creatinine, and Urea**   Adrenal insufficiency causes **hyponatremia** and **hyperkalemia** (mineralocorticoid deficiency). Renal function tests assess hydration status and rule out renal failure.   4. **Serum TSH and Free T4 (FT4)**   Exclude **hyperthyroidism** (weight loss, palpitations) or **hypothyroidism** (fatigue). TSH/FT4 levels are necessary despite atypical features for thyroid disease.   5. **Serum Liver Function Studies (LFTs)**   Evaluate for **liver failure** (nausea, abdominal pain) or chronic liver disease. Elevated transaminases or bilirubin would prompt further investigation.   6. **CT Scan of the Abdomen**   Assess for **adrenal gland pathology** (e.g., atrophy, hemorrhage from surgical trauma) and rule out intra-abdominal malignancy (weight loss, fatigue).  **Additional Considerations (If Clinically Indicated):**   - **Bedside Blood Glucose**: If hypoglycemia is suspected (lethargy, dizziness), though this is often addressed via lab glucose in comprehensive testing. - **Venous Blood Gas (pH, Bicarbonate, Lactate)**: If metabolic acidosis or adrenal crisis is suspected (e.g., severe hypotension, altered mental status).   **Less Relevant Tests Based on Presentation:**   - **Troponin, D-dimer, Calcium, Metanephrines**: No evidence of acute coronary syndrome, thromboembolism, hypercalcemia, or pheochromocytoma. - **Amylase, Acetylcholinesterase Antibodies, Echocardiography, Chest X-ray**: Symptoms do not suggest pancreatitis, myasthenia gravis, heart failure, or acute pulmonary pathology. - **Urine Dipstick**: No urinary symptoms or signs of infection.   **Conclusion:** The priority is confirming **adrenal insufficiency** (cortisol, electrolytes) and ruling out anemia, thyroid dysfunction, liver disease, and malignancy. **CT abdomen** is key to evaluate adrenal anatomy and abdominal pathology. Other tests (e.g., CBC, TSH/FT4, LFTs) address remaining differentials. |
| Dx1 | **Most Likely Diagnosis: Cortisol insufficiency complicated with an acute kidney injury**  **Key Evidence:**   1. **Severe Cortisol Deficiency:**   **9 AM cortisol: 1.8 mcg/dL (markedly below normal: 6.5–22.5 mcg/dL).**  **Diagnostic of adrenal insufficiency, likely Addison’s disease (primary adrenal failure).**   2. **Acute Kidney Injury (AKI):**   **Elevated creatinine (221 µmol/L) and BUN (14.9 mmol/L) with hyponatremia (130 mmol/L) and hyperkalemia (6.2 mmol/L).**  **Pre-renal AKI due to hypovolemia from aldosterone deficiency (sodium loss, hypovolemia).**   3. **Metabolic Acidosis:**   **pH 7.32, HCO₃⁻ 18 mmol/L: Compensated metabolic acidosis typical of adrenal crisis.**   4. **Exclusion of Other Diagnoses:**   **Anemia: Normal hemoglobin (140 g/L).**  **Hyperthyroidism: Normal TSH (0.9 mU/L) and FT4 (1.6 ng/dL).**  **Type 1 diabetes: Normal glucose (4.2 mmol/L), negative urine dipstick (no glycosuria).**  **Conclusion:**  **The findings confirm adrenal insufficiency (Addison’s disease) precipitating an adrenal crisis, with secondary hypovolemia, electrolyte derangements, and pre-renal AKI. Immediate treatment with IV hydrocortisone, fluid resuscitation, and electrolyte correction is critical.**  **Correct Answer: Cortisol insufficiency complicated with an acute kidney injury** | **Most Likely Diagnosis:** **• Cortisol insufficiency complicated with an acute kidney injury**  **Rationale:**   1. **Severe Cortisol Deficiency**:   **9 AM serum cortisol**: 1.8 mcg/dL (markedly low; normal 6.5–22.5 mcg/dL).  Classic for **primary adrenal insufficiency** (Addison’s disease) or adrenal crisis.   2. **Electrolyte Abnormalities**:   **Hyponatremia (Na⁺: 130 mmol/L)** and **hyperkalemia (K⁺: 6.2 mmol/L)**: Direct consequences of aldosterone deficiency (mineralocorticoid deficiency) in adrenal insufficiency.   3. **Acute Kidney Injury (AKI)**:   **Elevated creatinine (221 µmol/L)** and **BUN (14.9 mmol/L)**: Likely due to hypovolemia (dehydration) and hypotension from cortisol/aldosterone deficiency.   4. **Metabolic Acidosis**:   **Low pH (7.32)** and **low HCO₃⁻ (18 mmol/L)**: Reflects renal and systemic acid-base dysregulation from adrenal crisis.   5. **Exclusion of Other Options**:   **Anemia**: Hemoglobin is normal (140 g/L).  **Hyperthyroidism**: Normal TSH (0.9 mU/L) and FT4 (1.6 ng/dL).  **New type 1 diabetes**: Normal blood glucose (4.2 mmol/L).  **Conclusion**: The findings align with an **Addisonian crisis** (acute adrenal insufficiency) leading to hypovolemic shock, electrolyte derangements, and secondary AKI. Immediate glucocorticoid replacement and fluid resuscitation are critical. | **Answer:** **• Cortisol insufficiency complicated with an acute kidney injury**  **Rationale:**   1. **Adrenal Insufficiency (Addison’s Disease)**:   **Severely low 9 AM cortisol (1.8 mcg/dL)** confirms cortisol deficiency.  **Hyponatremia (Na+ 130 mmol/L)** and **hyperkalemia (K+ 6.2 mmol/L)** are classic electrolyte abnormalities due to mineralocorticoid (aldosterone) deficiency.   2. **Acute Kidney Injury (AKI)**:   Elevated **creatinine (221 µmol/L)** and **BUN (14.9 mmol/L)** indicate AKI, likely **prerenal** due to hypovolemia (dehydration from adrenal crisis).   3. **Metabolic Acidosis** (pH 7.32, HCO3⁻ 18 mmol/L) aligns with aldosterone deficiency and hypoperfusion.   **Why Other Options Are Incorrect:**   - **Anemia**: Normal hemoglobin (140 g/L). - **Hyperthyroidism**: Normal TSH (0.9 mU/L) and FT4 (1.6 ng/dL). - **Type 1 Diabetes**: Normal blood glucose (4.2 mmol/L), no ketones/glucose on urine dipstick, and no acidosis (lactate is normal).   This presentation is **classic for adrenal crisis** (Addisonian crisis) secondary to cortisol insufficiency, with AKI as a complication of dehydration and electrolyte derangements. |
| Diag2 | **Most Appropriate Next Steps:**   1. **Serum ACTH level** 2. **Serum anti-adrenal cortex antibodies** 3. **Plasma renin activity** 4. **Hydrocortisone 100 mg IM (or IV, if available)** 5. **0.9% saline 1 L IV over 1 hour** 6. **CT/MRI of the adrenal glands** 7. **Screening for tuberculosis**   **Rationale:**  **Immediate Management:**   - **Hydrocortisone 100 mg IM/IV: Urgent glucocorticoid replacement is critical to reverse adrenal crisis. IV is preferred, but IM is acceptable if IV access is delayed.** - **0.9% saline IV: Corrects hypovolemia, hyponatremia, and pre-renal AKI.**   **Diagnostic Evaluation:**   - **ACTH level:**   **Elevated ACTH confirms primary adrenal insufficiency (Addison’s disease).**  **Low ACTH suggests secondary (pituitary/hypothalamic) insufficiency.**   - **Anti-adrenal cortex antibodies: Identifies autoimmune Addison’s disease (most common cause in adults).** - **Plasma renin activity:**   **Elevated renin with low aldosterone supports primary adrenal insufficiency.**   - **CT/MRI adrenals: Assesses for adrenal atrophy, hemorrhage, infiltration (e.g., TB, malignancy).** - **TB screening: Tuberculosis is a major infectious cause of adrenal destruction globally.**   **Excluded Options:**   - **17-OH-progesterone: Rules out congenital adrenal hyperplasia, but presentation favors acquired adrenal insufficiency.** - **Acetylcholine esterase, ferritin, pituitary hormones, dexamethasone suppression test: Not relevant here.** - **Dopamine, metoprolol: No role in adrenal crisis management.** - **MRI pituitary: Reserved if secondary adrenal insufficiency is suspected (normal TSH/FT4 and primary adrenal pattern make this unlikely).**   **Key Priorities:**   1. **Treat the crisis with hydrocortisone and fluids.** 2. **Confirm etiology (autoimmune, infectious, structural) with ACTH, antibodies, imaging, and TB testing.** 3. **Long-term management requires glucocorticoid/mineralocorticoid replacement and addressing the underlying cause (e.g., TB treatment if positive).**   **Note: A Short Synacthen test is unnecessary during an acute crisis (baseline cortisol is already diagnostic at 1.8 mcg/dL). It may be used later to confirm adrenal insufficiency once stabilized.** | **Most Appropriate Next Steps:**   1. **Immediate Management**:   **Hydrocortisone 100 mg IM/IV**: Critical to replace glucocorticoids and address adrenal crisis.  **0.9% saline 1 L IV over 1 hour**: Correct hypovolemia, hyponatremia, and improve renal perfusion.   2. **Diagnostic Evaluation of Cortisol Deficiency**:   **Serum ACTH level**: Differentiates primary (↑ ACTH) vs. secondary (↓ ACTH) adrenal insufficiency.  **Plasma renin activity**: Elevated in primary adrenal insufficiency due to aldosterone deficiency.  **Short Synacthen test (ACTH stimulation test)**: Confirms adrenal insufficiency (blunted cortisol response).  **Serum anti-adrenal cortex antibodies**: Identifies autoimmune adrenalitis (most common cause of primary adrenal insufficiency).  **CT/MRI of the adrenal glands**: Evaluates for adrenal hemorrhage, atrophy, or infiltrative disease.  **Screening for tuberculosis**: TB is a common infectious cause of adrenal insufficiency in endemic regions.   3. **Additional Hormonal Assessment (if secondary adrenal insufficiency is suspected)**:   **Serum pituitary hormone studies**: Rule out hypopituitarism (e.g., low TSH, FSH/LH, or GH).  **MRI of the pituitary gland**: If pituitary pathology (e.g., tumor, infarction) is suspected.  **Excluded Options**:   - **Serum 17-OH-progesterone**: Used for congenital adrenal hyperplasia screening, not urgent here. - **Serum acetylcholine esterase level**: Irrelevant in adrenal insufficiency. - **Serum ferritin level**: No indication of hemochromatosis or iron overload. - **Overnight dexamethasone suppression test**: Evaluates Cushing’s syndrome, not adrenal insufficiency. - **Dopamine/Metoprolol**: Not indicated for hypotension in adrenal crisis (fluids and steroids are first-line).   **Conclusion**: Prioritize **hydrocortisone**, **IV fluids**, and diagnostic tests (ACTH, renin, Synacthen test) to confirm and characterize adrenal insufficiency. Imaging and antibody testing help identify the underlying cause. | **Answer:** **• Serum ACTH level** **• Plasma renin activity** **• Hydrocortisone 100 mg IM** **• 0.9% saline 1 L IV over 1 hour** **• CT/MRI of the adrenal glands** **• Screening for tuberculosis** **• Serum anti-adrenal cortex antibodies**  **Rationale:**   1. **Immediate Management:**   **Hydrocortisone 100 mg IM**: Critical to replace glucocorticoids in adrenal crisis (IV preferred if access is available).  **0.9% saline IV**: Correct hypovolemia, hyponatremia, and prerenal AKI.   2. **Confirm Primary Adrenal Insufficiency:**   **Serum ACTH**: Elevated ACTH confirms primary adrenal insufficiency (Addison’s disease).  **Plasma renin activity**: Elevated renin with low aldosterone supports mineralocorticoid deficiency.   3. **Identify Underlying Cause:**   **Anti-adrenal cortex antibodies**: Autoimmune Addison’s is the most common cause in adults.  **CT/MRI adrenal glands**: Rule out adrenal hemorrhage, infiltrative disease (e.g., metastases, infection), or atrophy.  **Tuberculosis screening**: TB is a leading infectious cause of adrenal insufficiency globally.  **Why Other Options Are Less Relevant:**   - **Serum 17-OH-progesterone**: Used for congenital adrenal hyperplasia (not suspected here). - **Short Synacthen test**: Confirms adrenal insufficiency but unnecessary in acute crisis (treatment takes priority). - **MRI pituitary**: For secondary adrenal insufficiency (normal TSH/FT4 and electrolyte abnormalities favor primary adrenal failure). - **Dopamine/metoprolol**: No role in adrenal crisis management. - **Serum ferritin/acetylcholine esterase**: Unrelated to cortisol deficiency.   **Key:** Stabilize the patient with glucocorticoids and fluids, then investigate the cause of primary adrenal insufficiency. |
| Dx2 | **Correct Answer: • Bilateral adrenal hemorrhage following major surgery**  **Rationale:**   1. **Imaging Evidence:**   **CT scan: Confirmed bilateral subacute adrenal hemorrhage.**  **Adrenal hemorrhage is a well-documented cause of acute primary adrenal insufficiency.**   2. **Clinical Context:**   **Adrenal hemorrhage often occurs in the setting of:**  **Major surgery (physiological stress, anticoagulation).**  **Sepsis (e.g., Waterhouse-Friderichsen syndrome).**  **Trauma or coagulopathy.**  **No evidence of infection (negative TB screen) or autoimmune disease (negative antibodies).**   3. **Exclusion of Other Causes:**   **Autoimmune adrenalitis: Ruled out by negative anti-adrenal antibodies.**  **Granulomatous lesions (e.g., TB): Excluded by negative PPD and imaging.**  **Metastatic lesions/hemochromatosis: Imaging showed hemorrhage, not tumors or iron deposition.**  **Hypopituitarism: Elevated ACTH (880 pg/mL) confirms primary adrenal insufficiency.**  **Why This Is the Most Likely Cause:**  **Bilateral adrenal hemorrhage is a recognized complication of major surgery, particularly in patients exposed to anticoagulation, physiological stress, or trauma. The CT findings directly identify hemorrhage as the etiology, making this the definitive diagnosis.** | **Correct Answer**: **• Bilateral adrenal hemorrhage following major surgery**  **Rationale**: The **CT scan showing bilateral subacute adrenal hemorrhage** is the definitive finding explaining the cause of adrenal insufficiency. Key supporting evidence includes:   1. **Primary adrenal insufficiency** (↑ ACTH, ↑ renin, failed Synacthen test). 2. **Structural adrenal damage** (hemorrhage on imaging). 3. **Exclusion of other causes**:   Negative anti-adrenal antibodies (rules out autoimmune adrenalitis).  Negative TB screening (rules out granulomatous disease).  Normal ferritin (rules out hemochromatosis).  Normal pituitary hormones (rules out hypopituitarism).  **Why not other options?**   - **Autoimmune adrenalitis**: Excluded by negative anti-adrenal antibodies. - **Granulomatous/metastatic lesions**: CT shows hemorrhage, not masses or infiltrates. - **Adrenal hemochromatosis**: Normal ferritin excludes iron overload. - **Hypopituitarism**: Normal pituitary hormones and elevated ACTH (pituitary function is intact).   **Bilateral adrenal hemorrhage** is a known cause of acute adrenal insufficiency, often triggered by stressors like surgery, sepsis, trauma, or anticoagulation. The CT findings confirm this diagnosis. | **Answer:** **• Bilateral adrenal hemorrhage following major surgery**  **Rationale:**   1. **Key Diagnostic Clues**:**CT scan**: Directly identifies **bilateral subacute adrenal hemorrhage**, which destroys adrenal tissue and causes acute insufficiency.   **Biochemical evidence**:  Extremely elevated ACTH (880 pg/mL) and plasma renin activity (600 ng/mL/hour).  Flat cortisol response to Synacthen stimulation (baseline 1.4 mcg/dL, post-stimulation 1.9 mcg/dL).  **Exclusion of other causes**:  Negative anti-adrenal antibodies (rules out autoimmune adrenalitis).  Negative TB screening (rules out granulomatous disease).   2. **Why This Option Fits Best**:   Bilateral adrenal hemorrhage is a well-documented cause of **acute primary adrenal insufficiency**. It is often triggered by stressors such as:  Major surgery, trauma, sepsis, or anticoagulation (even if surgery is not explicitly mentioned here, the hemorrhage itself is the critical finding).  The CT finding of hemorrhage directly explains the rapid onset of adrenal failure, unlike chronic causes (e.g., autoimmune, metastatic, or granulomatous disease).  **Why Other Options Are Less Likely:**   - **Autoimmune adrenalitis**: Negative anti-adrenal antibodies and acute presentation (not chronic atrophy) argue against this. - **Granulomatous/metastatic lesions**: CT shows hemorrhage, not masses or granulomas. - **Hypopituitarism**: Elevated ACTH confirms primary adrenal failure (pituitary disorders cause **low ACTH**). - **Hemochromatosis**: Normal ferritin and absence of iron overload history make this unlikely.   **Conclusion**: The CT evidence of **bilateral adrenal hemorrhage** is pathognomonic for this patient’s acute adrenal insufficiency. |
| Mang | **Appropriate Management Steps:**   - **0.9% saline IV 250 mL/hr** - **Hydrocortisone 50 mg IV every 6 hours**   **Rationale:**   1. **0.9% saline IV 250 mL/hr:**   **Adrenal crisis causes severe hypovolemia and hyponatremia. Aggressive fluid resuscitation with IV saline is critical to restore circulating volume and correct electrolyte imbalances.**   2. **Hydrocortisone 50 mg IV every 6 hours:**   **High-dose IV hydrocortisone is the cornerstone of treatment for adrenal crisis. It provides both glucocorticoid and mineralocorticoid activity in the acute phase. The dose aligns with stress-dose recommendations (50–100 mg every 6–8 hours).**  **Why Other Options Are Not Appropriate:**   - **Watch and wait: Adrenal crisis is life-threatening and requires immediate intervention, not observation.** - **Bilateral adrenalectomy: Adrenal hemorrhage does not require surgical removal unless there is uncontrolled bleeding (not indicated here).** - **Desmopressin: Used for diabetes insipidus, not adrenal insufficiency.** - **Fludrocortisone: While essential for long-term mineralocorticoid replacement, it is not urgent in the acute phase because high-dose hydrocortisone provides sufficient mineralocorticoid activity. It is initiated once the patient is stabilized on oral glucocorticoids.** - **Oral hydrocortisone: IV administration is preferred during crisis due to potential hemodynamic instability and impaired gastrointestinal absorption.**   **Conclusion: Immediate priorities are fluid resuscitation and high-dose IV hydrocortisone. Fludrocortisone and oral hydrocortisone are initiated later during transition to maintenance therapy.** | **Appropriate Management Steps**:   - **0.9% saline IV 250 mL/hr** - **Hydrocortisone 50 mg IV every 6 hours** - **Fludrocortisone 50 mcg once a day orally**   **Rationale**:   1. **0.9% saline IV 250 mL/hr**:   Adrenal crisis causes severe hypovolemia, hypotension, and electrolyte imbalances (e.g., hyponatremia, hyperkalemia). Aggressive IV fluid resuscitation with **0.9% saline** is critical to restore intravascular volume.   2. **Hydrocortisone 50 mg IV every 6 hours**:   High-dose **IV hydrocortisone** is the cornerstone of treatment. It provides immediate glucocorticoid replacement and has mineralocorticoid activity to stabilize blood pressure and electrolyte imbalances.   3. **Fludrocortisone 50 mcg once daily orally**:   While hydrocortisone has partial mineralocorticoid effects, **fludrocortisone** (a pure mineralocorticoid) is essential for long-term aldosterone replacement, especially given the patient’s markedly elevated plasma renin activity (indicating severe mineralocorticoid deficiency). It should be initiated once the patient is stabilized on IV hydrocortisone.  **Why Other Options Are Incorrect**:   - **Watch and wait**: Addisonian crisis is life-threatening and requires urgent treatment, not observation. - **Bilateral adrenalectomy**: The adrenal glands are already nonfunctional due to hemorrhage; surgery is unnecessary unless there is active bleeding or infection. - **Desmopressin**: Used for diabetes insipidus, not adrenal insufficiency. - **Oral hydrocortisone**: IV administration is required during acute crisis due to unreliable gastrointestinal absorption and hemodynamic instability.   **Next Steps**:   - Transition to oral hydrocortisone (e.g., 10 mg on waking, 5 mg midday, 5 mg at 5 PM) **after stabilization**. - Investigate the **trigger** for adrenal hemorrhage (e.g., anticoagulation, sepsis, trauma). - Educate the patient on lifelong glucocorticoid/mineralocorticoid replacement and stress-dose steroids. | **Appropriate Management Steps:**   - **0.9% saline IV 250 mL/hr** - **Hydrocortisone 50 mg IV every 6 hours**   **Rationale:**   1. **0.9% Saline IV**:   Adrenal crisis causes **hypovolemia, hyponatremia, and hypotension**. Rapid IV fluid resuscitation with normal saline corrects dehydration and electrolyte imbalances.   2. **Hydrocortisone IV**:   **Stress-dose hydrocortisone** (50–100 mg IV every 6–8 hours) is critical to replace cortisol and stabilize the patient. Hydrocortisone has both glucocorticoid and partial mineralocorticoid activity, which is sufficient in the acute phase.  **Why Other Options Are Not Appropriate:**   - **Watch and wait**: Adrenal crisis is life-threatening and requires immediate intervention. - **Adrenalectomy**: Bilateral adrenal hemorrhage does not require surgical removal unless there is uncontrolled bleeding (not indicated here). - **Desmopressin**: Used for diabetes insipidus, not adrenal insufficiency. - **Fludrocortisone**: While fludrocortisone is part of **long-term management** for mineralocorticoid replacement, it is not urgent in the acute phase. High-dose IV hydrocortisone provides adequate mineralocorticoid coverage initially. - **Oral hydrocortisone**: Patients in crisis may have impaired absorption or vomiting; IV administration is necessary.   **Conclusion**: Immediate priorities are **fluid resuscitation** and **IV glucocorticoids**. Fludrocortisone and oral hydrocortisone are initiated once the patient is stabilized and transitioned to maintenance therapy. |
| Case 29 |  |  |  |
| Diff | **Differential Diagnoses to Include:**   - **Hyperthyroidism** - **Occult malignancy** - **Drug effects** - **HIV infection** - **Tuberculosis** - **Malabsorption**   **Rationale:**   1. **Hyperthyroidism:**   **Key findings: Weight loss despite increased appetite, palpitations, heat intolerance, irritability, diarrhea, exophthalmos, and tachycardia.**  **Graves' disease is the most likely etiology given the ocular symptoms and hypermetabolic state.**   2. **Occult malignancy:**   **Weight loss is a red flag for malignancy (e.g., lymphoma, gastrointestinal cancers).**  **Though hyperthyroidism explains many symptoms, malignancy must be ruled out.**   3. **Drug effects:**   **Stimulants (e.g., amphetamines) or exogenous thyroid hormone use can mimic hyperthyroidism.**  **Requires a thorough medication/substance history to exclude.**   4. **HIV infection:**   **Chronic HIV can cause weight loss, diarrhea, and fatigue.**  **Less likely without risk factors or opportunistic infections, but screening is prudent.**   5. **Tuberculosis:**   **Classic cause of weight loss and systemic symptoms.**  **Less likely without fever, night sweats, or cough, but still considered in endemic areas.**   6. **Malabsorption:**   **Conditions like celiac disease or inflammatory bowel disease may cause diarrhea and weight loss.**  **Less typical here due to preserved appetite and semi-formed (not fatty) stools, but still possible.**  **Excluded Options:**   - **Bipolar disorder: Psychiatric symptoms (irritability, restlessness) are secondary to hyperthyroidism, not primary.** - **Diabetes: No polyuria, polydipsia, or hyperglycemia symptoms.** - **Endocarditis: Absence of fever, murmurs, embolic phenomena, or cardiac findings.**   **Conclusion: While Graves' disease is the leading diagnosis, the differential must include occult malignancy, drug effects, HIV, TB, and malabsorption to ensure life-threatening or treatable conditions are not missed.** | **Differential Diagnoses to Include**:   - **Hyperthyroidism** - **Occult malignancy** - **Malabsorption** - **HIV infection** - **Tuberculosis** - **Drug effects**   **Rationale**:   1. **Hyperthyroidism** (e.g., Graves' disease):   **Weight loss** despite increased appetite, **heat intolerance**, palpitations, diarrhea, irritability, and **gritty eyes** (suggestive of Graves' ophthalmopathy). Muscle weakness and tachycardia further support this.   2. **Occult malignancy**:   Unexplained weight loss, fatigue, and systemic symptoms (e.g., night sweats not reported here) warrant ruling out malignancies (e.g., lymphoma, gastrointestinal cancers).   3. **Malabsorption** (e.g., celiac disease, Crohn’s):   Chronic diarrhea and weight loss, though the absence of steatorrhea or abdominal pain makes this less likely.   4. **HIV infection**:   Weight loss, chronic diarrhea, and systemic symptoms (e.g., fatigue) are common in advanced HIV. Risk factors should be assessed.   5. **Tuberculosis**:   Weight loss, fatigue, and night sweats (denied here), but TB can present atypically. A chest X-ray or PPD test may be needed.   6. **Drug effects**:   Medications (e.g., stimulants, thyroid hormone excess, or illicit drugs) can mimic hyperthyroidism or cause weight loss and palpitations.  **Why Other Options Are Less Likely**:   - **Bipolar disorder**: Irritability and restlessness may occur, but physical symptoms (weight loss, diarrhea, eye changes) strongly suggest an organic cause. - **Diabetes**: Weight loss occurs in uncontrolled diabetes, but the absence of polyuria/polydipsia and normal urination make this unlikely. - **Endocarditis**: No fever, heart murmurs, or other signs (e.g., embolic phenomena) to support this diagnosis.   **Next Steps**:   1. **Thyroid function tests** (TSH, free T4/T3) and **TSH receptor antibodies** (TRAb) to confirm hyperthyroidism/Graves’. 2. **HIV testing**, **chest X-ray** (for TB), and **stool studies** (for malabsorption). 3. **Cancer screening** (e.g., colonoscopy, CT imaging) if no clear cause is found. 4. **Medication review** to exclude drug-induced symptoms.   This patient’s presentation is **most consistent with Graves' disease**, but the differential must broadly address weight loss and systemic symptoms to avoid missing critical diagnoses. | **Appropriate Differential Diagnoses:**   - **Hyperthyroidism** - **Drug effects** - **Occult malignancy** - **Malabsorption**   **Rationale:**   1. **Hyperthyroidism**:   **Key Supporting Features**: Weight loss despite increased appetite, heat intolerance, palpitations, tremor, diarrhea, irritability, diffuse goiter with bruit, and ophthalmopathy (gritty eyes). Classic for **Graves' disease**, but other causes of hyperthyroidism (e.g., toxic nodular goiter, thyroiditis) should be considered.   2. **Drug Effects**:   **Key Consideration**: Exogenous thyroid hormone use, stimulants (e.g., amphetamines), or medications like amiodarone (can induce thyroid dysfunction).   3. **Occult Malignancy**:   **Key Supporting Features**: Significant unintentional weight loss. Cancers (e.g., gastrointestinal, lymphoma) or hormone-secreting tumors (e.g., pheochromocytoma, carcinoid) can mimic hypermetabolic states.   4. **Malabsorption**:   **Key Supporting Features**: Weight loss and diarrhea. Conditions like celiac disease or chronic pancreatitis may present similarly, though lack of fatty stools or abdominal pain makes this less likely.  **Why Other Options Are Less Likely:**   - **Bipolar Disorder**: Irritability and restlessness are present, but physical symptoms (weight loss, tachycardia, goiter) are not explained by a primary psychiatric disorder. - **Diabetes**: Absence of polydipsia, polyuria, or hyperglycemia makes this unlikely. - **Endocarditis**: No fever, murmurs, or embolic phenomena. - **HIV Infection**: Weight loss and diarrhea occur in advanced HIV, but hypermetabolic symptoms and goiter are atypical without opportunistic infections. - **Tuberculosis**: No fever, night sweats, or cough.   **Conclusion**: While **Graves' disease** is the leading diagnosis, the differential should include **drug-induced hyperthyroidism**, **occult malignancy**, and **malabsorption** to ensure comprehensive evaluation. |
| Diag1 | **Most Appropriate Studies to Obtain:**   1. **Serum TSH, free T4, and free T3 levels**   **Critical to confirm hyperthyroidism and assess thyroid function.**   2. **Electrocardiogram (ECG)**   **Evaluate for arrhythmias (e.g., atrial fibrillation) or signs of thyrotoxic heart disease.**   3. **Complete Blood Count (CBC)**   **Rule out anemia, infection, or hematologic malignancy contributing to systemic symptoms.**   4. **Serum electrolytes, BUN, and creatinine levels**   **Assess for electrolyte imbalances (e.g., hypokalemia, hypocalcemia) due to diarrhea/muscle cramps and evaluate renal function.**   5. **Chest X-ray**   **Screen for tuberculosis, occult malignancy (e.g., lung cancer), or cardiac enlargement.**   6. **Serum glucose level**   **Rule out diabetes mellitus (unlikely given lack of polyuria/polydipsia, but weight loss warrants confirmation).**  **Rationale for Excluded Options:**   - **CT scan of the head: Not indicated unless focal neurologic deficits or suspicion of intracranial pathology (e.g., pituitary tumor), which are absent here.** - **Serum ethanol level: No history or findings suggestive of alcohol misuse.** - **Serum troponin T level: No chest pain or signs of acute coronary syndrome; palpitations are more likely due to hyperthyroidism.** - **Overnight dexamethasone suppression test: Used to diagnose Cushing’s syndrome, which is not supported by the clinical picture.**   **Conclusion: Prioritize thyroid function tests, ECG, CBC, electrolytes, chest X-ray, and glucose to confirm hyperthyroidism, assess complications, and exclude key differentials (e.g., malignancy, TB).** | The most appropriate studies to obtain for this patient are:   1. **Serum TSH, free T4, and free T3 levels**:   **Critical** to confirm hyperthyroidism. A suppressed TSH with elevated free T4/T3 supports the diagnosis of Graves' disease.   2. **ECG**:   Assess for cardiac manifestations of hyperthyroidism (e.g., sinus tachycardia, atrial fibrillation). Palpitations and tachycardia (108 bpm) necessitate evaluation for arrhythmias.   3. **Serum electrolytes, BUN, and creatinine levels**:   Evaluate for electrolyte abnormalities (e.g., hypokalemia, hypomagnesemia) contributing to muscle cramps/weakness. BUN/creatinine assess renal function, which may influence treatment decisions.  **Rationale for Excluding Other Options:**   - **Complete blood count (CBC)**: Not urgent unless infection or anemia is suspected (not highlighted here). - **CT scan of the head**: Ophthalmopathy in Graves' disease is clinically diagnosed; imaging is reserved for atypical cases or compressive symptoms. - **Serum ethanol level**: No history or symptoms suggesting alcohol-related issues. - **Serum glucose level**: No symptoms of diabetes (e.g., polyuria, polydipsia). - **Chest x-ray**: Shortness of breath is more likely due to hyperthyroidism-induced increased metabolic demand, not cardiopulmonary pathology. - **Serum troponin T level**: No chest pain or signs of acute ischemia. - **Overnight dexamethasone suppression test**: Irrelevant here; used for Cushing’s syndrome, which is not in the differential.   **Summary:**  Prioritize **thyroid function tests** to confirm hyperthyroidism, **ECG** to evaluate cardiac involvement, and **electrolytes/BUN/creatinine** to assess metabolic contributors to muscle symptoms. These studies directly address the most likely diagnosis (Graves' disease) and its systemic effects. | **Most Appropriate Studies to Guide Diagnosis:**   - **Serum TSH, free T4, and free T3 levels** - **ECG** - **Serum electrolytes, BUN, and creatinine levels** - **Complete blood count (CBC)**   **Rationale:**   1. **Serum TSH, free T4, and free T3 levels**:   **Critical** to confirm or exclude **hyperthyroidism**. Expected findings in Graves’ disease: **↓ TSH**, ↑ free T4/T3.   2. **ECG**:   **Palpitations and tachycardia** (HR 108 bpm) suggest possible **sinus tachycardia** or arrhythmias (e.g., atrial fibrillation), which are common in hyperthyroidism.   3. **Serum electrolytes, BUN, and creatinine levels**:   **Muscle cramps and weakness** may reflect electrolyte abnormalities (e.g., hypokalemia, hypomagnesemia). Also assesses renal function, which can influence management.   4. **CBC**:   Evaluate for anemia, infection, or leukocytosis (though less likely here). Weight loss and diarrhea could rarely suggest chronic disease or malignancy.  **Why Other Options Are Less Appropriate:**   - **CT scan of the head**: No neurologic deficits or headaches to suggest intracranial pathology. - **Serum ethanol level**: No history of alcohol use or intoxication symptoms. - **Serum glucose level**: Absence of polyuria, polydipsia, or hyperglycemia symptoms. - **Chest x-ray**: No cough, fever, or respiratory signs to warrant evaluation for TB or malignancy. - **Serum troponin T level**: No chest pain or signs of acute coronary syndrome. - **Overnight dexamethasone suppression test**: Used for Cushing’s syndrome, which is not in the differential.   **Conclusion**: Prioritize **thyroid function tests** to confirm hyperthyroidism, **ECG** for cardiac evaluation, and **electrolytes/BUN/creatinine** to assess metabolic status. CBC is a reasonable adjunct for general screening. |
| Dx1 | **Answer: • Primary hyperthyroidism**  **Explanation:**  **The biochemical findings confirm primary hyperthyroidism:**   1. **Suppressed TSH (<0.01 mU/L) indicates lack of pituitary feedback due to excessive thyroid hormone production.** 2. **Elevated Free T4 (FT4) and Free T3 (FT3) confirm overactivity of the thyroid gland itself.** 3. **Sinus tachycardia on ECG and symptoms (weight loss, diarrhea, palpitations) are consistent with thyrotoxicosis.**   **Why Other Options Are Incorrect:**   - **Diabetes: Random glucose of 126 mg/dL (7 mmol/L) is borderline but not diagnostic for diabetes. Weight loss in hyperthyroidism is unrelated to glucose metabolism.** - **Drug effects: No history of medication use (e.g., amiodarone, lithium, or exogenous thyroid hormone) to suggest drug-induced hyperthyroidism.** - **Malabsorption: Normal electrolytes, CBC, and absence of anemia or fatty stools make this unlikely. Diarrhea in this case is due to hyperthyroidism.** - **Secondary hyperthyroidism: Caused by a pituitary TSH-secreting tumor, which would result in elevated TSH (not suppressed) alongside high T4/T3.**   **Final Diagnosis:**  **Graves’ disease is the most likely etiology of primary hyperthyroidism in this patient, given the absence of thyroid nodules, pain, or recent illness. Confirmatory testing (e.g., TSH receptor antibodies, thyroid ultrasound) would further support this diagnosis.** | The most likely diagnosis for this patient is:  **• Primary hyperthyroidism**  **Explanation**   - **Suppressed TSH (<0.01 mU/L)** with **elevated free T4 (4.2 ng/dL)** and **free T3 (15.5 pg/mL)** definitively confirm **primary hyperthyroidism** (thyroid gland overactivity causing autonomous hormone production). - **Graves' disease** is the most common cause of primary hyperthyroidism in this context, especially if clinical features (e.g., goiter, ophthalmopathy) are present. - Secondary hyperthyroidism (pituitary-driven) is ruled out because TSH is suppressed, not elevated. - **Diabetes**, **drug effects**, and **malabsorption** are inconsistent with the biochemical and clinical picture.   This patient’s findings are pathognomonic for **primary hyperthyroidism**. | **Answer:** **• Primary hyperthyroidism**  **Explanation:**   1. **Lab Results**:   **TSH**: <0.01 mU/L (markedly suppressed, normal 0.35-5.5 mU/L).  **Free T4**: 4.2 ng/dL (elevated, normal 0.9-1.7 ng/dL).  **Free T3**: 15.5 pg/mL (elevated, normal 2.3-4.3 pg/mL).  **Pattern**: **↓ TSH + ↑ T4/T3** confirms **primary hyperthyroidism** (thyroid gland overactivity with pituitary feedback suppression).   2. **Clinical Correlation**:   Tachycardia (HR 110 bpm on ECG), weight loss, diarrhea, and goiter (from prior context) align with hyperthyroidism.  **Graves’ disease** is the most likely etiology, given the biochemical profile and typical symptoms.   3. **Exclusion of Other Options**:   **Secondary hyperthyroidism**: Requires **elevated TSH** (e.g., TSH-secreting pituitary tumor), which is absent here.  **Diabetes**: Random glucose of 126 mg/dL is borderline, not diagnostic. No classic symptoms (polyuria, polydipsia).  **Drug effects**: No history of thyroid hormone replacement, iodine contrast, or medications (e.g., amiodarone) causing thyroid dysfunction.  **Malabsorption**: Normal electrolytes, Hb, and absence of steatorrhea/malnutrition make this unlikely.  **Conclusion**: The biochemical and clinical evidence overwhelmingly supports **primary hyperthyroidism**, most likely due to **Graves’ disease**. |
| Diag2 | **Answer:**   - **Serum TSH receptor antibodies** - **Ophthalmology consultation** - **Technetium-99m thyroid uptake scan**   **Explanation:**   1. **Serum TSH receptor antibodies (TRAb):**   **Key for diagnosing Graves’ disease, the most common cause of primary hyperthyroidism. TRAb is positive in >95% of Graves’ cases.**   2. **Ophthalmology consultation:**   **Graves’ disease is associated with thyroid eye disease (e.g., proptosis, diplopia, lid retraction). Even asymptomatic patients require baseline evaluation.**   3. **Technetium-99m thyroid uptake scan:**   **Distinguishes Graves’ disease (diffuse high uptake) from other causes like toxic multinodular goiter (patchy uptake) or thyroiditis (low/no uptake).**  **Why Other Options Are Incorrect:**   - **Anterior pituitary hormone levels:**   **Unnecessary in primary hyperthyroidism (TSH is suppressed due to thyroid autonomy). Pituitary testing is only relevant if secondary hyperthyroidism (TSH-secreting pituitary tumor) is suspected, which presents with elevated TSH (not suppressed).**   - **CT scan of the neck and thorax:**   **Not routinely indicated unless there is suspicion for malignancy (e.g., thyroid mass, compressive symptoms) or retrosternal goiter. The chest x-ray was normal, and no structural abnormalities were reported.**  **Next Steps in Context:**   - **Graves’ disease is the likely diagnosis. TRAb positivity and diffuse uptake on the thyroid scan would confirm this.** - **Ophthalmology evaluation ensures early detection/manage­ment of thyroid eye disease, which impacts treatment choices (e.g., avoiding radioiodine if severe eye disease is present).** | The most appropriate next steps to evaluate the cause of primary hyperthyroidism in this patient are:  **• Serum TSH receptor antibodies** **• Technetium-99m thyroid uptake scan** **• Ophthalmology consultation**  **Explanation**   1. **Serum TSH receptor antibodies (TRAb/TSI)**:   **Critical to confirm Graves' disease**, the most common cause of primary hyperthyroidism. TRAb/TSI are positive in 90-95% of Graves' cases.   2. **Technetium-99m thyroid uptake scan**:   Differentiates Graves' disease (diffuse increased uptake) from other causes like toxic nodular goiter (focal uptake) or thyroiditis (low/no uptake).   3. **Ophthalmology consultation**:   Graves' disease is associated with **thyroid eye disease** (proptosis, diplopia, lid retraction). Even if symptoms are absent, baseline evaluation is recommended.  **Why the others are less appropriate:**   - **Anterior pituitary hormone levels**: Not needed in **primary** hyperthyroidism (TSH is suppressed; pituitary dysfunction would cause **secondary** hyperthyroidism with elevated TSH). - **CT scan of the neck and thorax**: Unnecessary unless there is suspicion of compressive symptoms, retrosternal goiter, or malignancy (not indicated here).   These steps will confirm the etiology (e.g., Graves' disease) and guide definitive management (antithyroid drugs, radioactive iodine, or surgery). | **Answer:** The most appropriate next steps are:   - **Serum TSH receptor antibodies** - **Ophthalmology consultation** - **Technetium-99m thyroid uptake scan**   **Explanation:**   1. **Serum TSH receptor antibodies (TRAb)**:   **Purpose**: Confirm **Graves' disease**, the most common cause of primary hyperthyroidism. TRAb is positive in >90% of Graves’ cases.  **Why not other options?**: Anterior pituitary hormones (e.g., ACTH, FSH/LH) are unnecessary here because primary hyperthyroidism is confirmed (↓ TSH + ↑ T4/T3).   2. **Ophthalmology consultation**:   **Purpose**: Evaluate for **Graves’ ophthalmopathy** (e.g., proptosis, diplopia, lid retraction), which occurs in ~30% of Graves’ patients. Early referral aids in managing eye complications.   3. **Technetium-99m thyroid uptake scan**:   **Purpose**: Differentiate Graves’ disease (diffuse ↑ uptake) from toxic nodular goiter (focal ↑ uptake) or thyroiditis (↓ uptake).  **Excluded Options:**   - **Anterior pituitary hormone levels**: Not needed in primary hyperthyroidism (pituitary function is normal; TSH is suppressed due to thyroid autonomy). - **CT scan of neck and thorax**: Unnecessary unless suspecting compression (e.g., retrosternal goiter) or malignancy. Ultrasound is preferred for thyroid evaluation.   **Clinical Context**: In confirmed primary hyperthyroidism, the priority is to identify the etiology (e.g., Graves’ vs. toxic nodular disease) and screen for complications (e.g., ophthalmopathy). TRAb and uptake scan are key for diagnosis; ophthalmology referral addresses potential Graves’-specific complications. |
| Dx2 | **Answer:**   - **Graves disease**   **Explanation:**  **Graves’ disease is the most common cause of hyperthyroidism and is strongly supported by:**   1. **Positive TSH receptor antibodies (TRAb): Pathognomonic for Graves’ disease.** 2. **Thyroid eye disease: A hallmark extrathyroidal manifestation.** 3. **Diffuse increased uptake on thyroid scan: Reflects uniform hyperactivity of the gland, typical of Graves’ disease.**   **Why Other Options Are Less Likely:**   - **Toxic multinodular goiter: Causes hyperthyroidism with patchy uptake on thyroid scan, not diffuse. More common in older adults with long-standing goiter.** - **Subacute thyroiditis: Presents with painful thyroid, transient hyperthyroidism, and low/no uptake on thyroid scan.** - **Hashimoto’s thyroiditis: Typically causes hypothyroidism. A transient hyperthyroid phase (“hashitoxicosis”) is rare and not associated with TRAb or increased uptake.** - **Factitious thyrotoxicosis: Results from exogenous thyroid hormone intake. Thyroid uptake scan shows suppressed uptake (thyroid gland is inactive).** - **Iodine-induced thyrotoxicosis: Occurs in iodine-deficient regions after iodine exposure (e.g., contrast media). Uptake varies but is often low.** - **TSH-producing pituitary adenoma: Causes secondary hyperthyroidism with elevated TSH (not suppressed). Extremely rare.**   **Conclusion: The triad of TRAb positivity, thyroid eye disease, and diffuse uptake confirms Graves’ disease as the definitive diagnosis.** | **Answer:** **• Graves disease**  **Explanation**  The patient’s **positive TSH receptor antibodies (TRAb)**, **diffuse increased uptake on thyroid scan**, and **thyroid eye disease** are diagnostic of **Graves' disease**, the **most common cause of hyperthyroidism**.  **Why Other Options Are Less Likely:**   - **Factitious thyrotoxicosis**: Thyroid uptake scan would show **low/no uptake** (exogenous thyroid hormone use). - **Toxic multinodular goiter**: Thyroid scan shows **focal areas of increased uptake** (nodules), not diffuse uptake. - **Subacute thyroiditis**: Causes transient hyperthyroidism with **low uptake on scan** and often a **painful thyroid**. - **Hashimoto’s thyroiditis**: Typically causes hypothyroidism; transient hyperthyroidism (hashitoxicosis) is rare and not associated with TRAb or eye disease. - **Iodine-induced thyrotoxicosis**: Occurs in iodine-deficient regions after iodine exposure (e.g., contrast), but TRAb would be negative. - **TSH-producing pituitary adenoma**: Causes **elevated TSH** (secondary hyperthyroidism), unlike the **suppressed TSH** in primary hyperthyroidism.   Graves' disease is the **only diagnosis** consistent with **autoantibodies, diffuse thyroid hyperactivity, and extrathyroidal manifestations** (e.g., eye disease). | **Answer:** **Graves disease**  **Explanation:**  **Graves’ disease** is the **most common cause of hyperthyroidism** and is strongly supported by the following key findings in this patient:   1. **Positive TSH receptor antibodies (TRAb)**: Pathognomonic for Graves’ disease (>90% specificity). 2. **Thyroid eye disease**: A hallmark of Graves’ disease, occurring in ~30% of patients. 3. **Diffuse increased uptake on technetium-99m thyroid scan**: Indicates uniform thyroid hyperactivity, typical of Graves’ disease.   **Why Other Options Are Less Likely:**   - **Factitious thyrotoxicosis**: Excluded by elevated thyroid uptake (exogenous thyroid hormone suppresses uptake). - **Hashimoto’s thyroiditis**: Typically causes hypothyroidism; a transient hyperthyroid phase (“hashitoxicosis”) is rare and lacks TRAb or eye disease. - **Iodine-induced thyrotoxicosis**: Occurs in iodine-deficient regions or after iodine exposure (e.g., contrast/amiodarone), but uptake is usually low/normal. - **Subacute thyroiditis**: Presents with painful thyroid, transient hyperthyroidism, and **low uptake on scan**. - **Toxic multinodular goiter**: Causes focal ↑ uptake (not diffuse) and lacks TRAb/eye disease. - **TSH-producing pituitary adenoma**: Very rare; TSH would be elevated (not suppressed), conflicting with primary hyperthyroidism.   **Conclusion**: The clinical, laboratory, and imaging findings are diagnostic of **Graves’ disease**, the leading cause of hyperthyroidism. |
| Mang | **Appropriate Management Steps:**   - **Methimazole 20 mg orally twice a day** - **Propranolol 40 mg orally 3 times a day** - **Hypromellose eye drops 4 times a day and Lacrilube eye ointment at night** - **Smoking cessation program**   **Explanation:**   1. **Methimazole:**   **First-line antithyroid drug for Graves’ disease.**  **Dose of 20 mg twice daily is appropriate for moderate to severe hyperthyroidism. Methimazole is preferred over propylthiouracil (PTU) due to its better safety profile, except in specific scenarios (e.g., pregnancy).**   2. **Propranolol:**   **Beta-blockers provide rapid symptom relief for tachycardia, tremors, and anxiety associated with hyperthyroidism.**   3. **Hypromellose eye drops and Lacrilube ointment:**   **Essential for managing mild thyroid eye disease (TED) symptoms like dryness and irritation.**   4. **Smoking cessation program:**   **Smoking exacerbates TED; cessation is critical to prevent progression.**  **Why Other Options Are Not Appropriate:**   - **Propylthiouracil (PTU): Less preferred due to higher risk of hepatotoxicity. Reserved for specific cases (e.g., first-trimester pregnancy).** - **Radioactive iodine (RAI): Controversial in active TED. Requires steroid prophylaxis if used, which is not mentioned here. Typically deferred until TED is stable.** - **Emergency thyroidectomy: Reserved for life-threatening complications (e.g., airway obstruction), not mild TED.** - **Amiodarone, Potassium iodide, Levothyroxine: Exacerbate hyperthyroidism or are contraindicated.**   **Conclusion: Immediate management focuses on controlling hyperthyroidism (methimazole + propranolol), addressing eye symptoms, and mitigating TED risk factors (smoking cessation).** | **Appropriate Management Steps for Graves' Disease with Thyroid Eye Disease (TED):**   1. **Methimazole 20 mg orally twice a day**   **First-line antithyroid drug** to reduce thyroid hormone synthesis. Preferred over propylthiouracil (PTU) due to better safety profile and efficacy, except in specific scenarios (e.g., first-trimester pregnancy).   2. **Propranolol 40 mg orally 3 times a day**   **Beta-blockers** control adrenergic symptoms (e.g., tachycardia, tremors) while awaiting thyroid hormone normalization.   3. **Hypromellose eye drops 4 times a day and Lacrilube eye ointment at night**   **Symptomatic relief for mild TED** (dryness, irritation). Essential for ocular surface protection.   4. **Smoking cessation program**   **Smoking exacerbates TED progression**. Cessation is critical to prevent worsening eye disease.  **Why Other Options Are Inappropriate:**   - **Amiodarone**: Contains iodine, which may worsen hyperthyroidism. - **Propylthiouracil (PTU)**: Second-line due to hepatotoxicity risk; reserved for specific cases (e.g., thyroid storm, methimazole allergy). - **Levothyroxine**: Contraindicated in hyperthyroidism (replaces thyroid hormone, worsening symptoms). - **Potassium iodide**: Used short-term preoperatively or in thyroid storm, not for chronic management. - **Radioactive iodine (RAI)**: May worsen TED unless combined with steroids. Mild TED does not preclude RAI, but requires careful risk-benefit discussion. - **Emergency thyroidectomy**: Reserved for severe complications (e.g., airway obstruction), not indicated here.   **Key Considerations:**   - **Definitive therapy** (RAI or thyroidectomy) may be discussed later but is not urgent unless antithyroid drugs fail or are contraindicated. - **Monitor TED progression** and collaborate with ophthalmology for advanced therapies (e.g., steroids, biologics) if needed.   **Selected Options**:   - **Methimazole** - **Propranolol** - **Hypromellose/Lacrilube** - **Smoking cessation program** | **Appropriate Management Steps**:   1. **Methimazole 20 mg orally twice a day**   First-line antithyroid drug for Graves’ disease to reduce thyroid hormone production.   2. **Propranolol 40 mg orally 3 times a day**   Beta-blockers alleviate symptoms like tachycardia, tremors, and anxiety while awaiting antithyroid drug effects.   3. **Hypromellose eye drops 4 times a day and Lacrilube eye ointment at night**   Lubricants manage dryness and irritation associated with mild thyroid eye disease.   4. **Smoking cessation program**   Smoking exacerbates thyroid eye disease; cessation is critical to prevent progression.  **Incorrect Options:**   - **Amiodarone**: Contains iodine, which can worsen hyperthyroidism; contraindicated in Graves’ disease. - **Propylthiouracil (PTU)**: Less preferred than methimazole due to higher risk of hepatotoxicity; reserved for specific cases (e.g., first-trimester pregnancy). - **Levothyroxine**: Contraindicated in hyperthyroidism (replaces thyroid hormone, worsening symptoms). - **Potassium iodide**: Not used in routine management; may transiently worsen thyrotoxicosis. - **Radioactive iodine (RAI)**: Typically deferred in active thyroid eye disease (may exacerbate symptoms without steroid cover). - **Emergency thyroidectomy**: Reserved for severe complications (e.g., airway obstruction, thyroid storm).   **Conclusion**: Initial management focuses on **symptom control** (propranolol), **reducing hormone synthesis** (methimazole), **eye care**, and **smoking cessation**. Definitive therapies (RAI/surgery) are considered later. |
